# Effect of different scaling instruments on the roughness of esthetic resin composite restorations: an *in vitro* study

**DOI:** 10.3389/fdmed.2026.1894177

**Published:** 2026-08-28

**Authors:** Abdulkareem Alhumaidan, Turki Alshehri, Shahad T. Alameer, Dhai Albishri, Abdullah Al Madani, Mohammed Al Qranei, Hoda Albaqawi, Abdulrahman A. Balhaddad

**Affiliations:** 1 Department of Preventive Dental Sciences, College of Dentistry, Imam Abdulrahman Bin Faisal University, Dammam, Saudi Arabia; 2 Department of Substitutive Dental Sciences, College of Dentistry, Imam Abdulrahman Bin Faisal University, Dammam, Saudi Arabia; 3 College of Dentistry, Imam Abdulrahman Bin Faisal University, Dammam, Saudi Arabia; 4 Dental Hospital, College of Dentistry, Imam Abdulrahman Bin Faisal University, Dammam, Saudi Arabia; 5 Department of Restorative Dental Sciences, College of Dentistry, Imam Abdulrahman Bin Faisal University, Dammam, Saudi Arabia

**Keywords:** abrasion, composite, dental, roughness, scaling

## Abstract

**Objectives:**

This *in vitro* study aimed to evaluate the effect of different scaling methods on the roughness of various resin composites used in anterior teeth.

**Methods:**

Seven commercially available resin composite materials were tested: 3M™ Filtek™ Z350 XT, Beautifil II, and Beautifil II LS, G-ænial Anterior, IPS Empress Direct, Tokuyama PALFIQUE LX5, Vittra APS FKG. Disc-shaped specimens (6 mm in diameter and 2 mm in thickness) were prepared using a 3D-printed mold. A total of 30 specimens per brand were fabricated and randomly divided into three sub-groups according to the scaling technique: stainless-steel hand scaler (metal scaling), plastic hand scaler (plastic scaling), and piezoelectric ultra-sonic scaler tip (ultra-sonic scaling). Baseline surface roughness values were measured using a non-contact optical profilometer. Each specimen was then subjected to 40 scaling strokes, and post-scaling surface roughness was remeasured using the same method. The change in average surface roughness was calculated. Two and One-way ANOVA with Tukey's *post hoc* test analyzed the data (*α*= 0.05).

**Results:**

Both scaler method and resin composite type were determinant factors (*p* < 0.001) in modulating the roughness of resin composite restorations. Metal scaling consistently produced the greatest surface roughness change (*Δ*Ra, µm) across all composites, with G-ænial Anterior recording the highest *Δ*Ra value (2.23 ± 0.91 µm) and IPS Empress Direct the lowest (1.23 ± 0.82 µm). Plastic scaling resulted in the least *Δ*Ra across all materials (range: 0.00–0.10 µm), with IPS Empress Direct recording the highest change (0.10 ± 0.08 µm) and G-ænial Anterior and Tokuyama PALFIQUE LX5 revealing almost no change. Ultra-sonic scaling produced intermediate roughness change, with G-ænial Anterior again recording the highest value (1.61 ± 0.98 µm). Metal scaling produced significantly higher *Δ*Ra than both plastic and ultra-sonic methods in most resin composites (*p* < 0.05), while plastic and ultra-sonic groups were largely comparable.

**Conclusion:**

Within the limitations of the study, plastic scalers caused the least surface alteration and may be considered the most conservative option for maintaining the surface smoothness of resin composites.

## 1 Introduction

Resin composites were first introduced in the 1960s and are the most commonly used restorative material for direct dental restorations (1, 2). Over the last few decades, advancements in material quality and application techniques have significantly improved their clinical performance (3). However, the selection of restorative materials is no longer based only on these factors, as several other factors can also influence the longevity and properties of dental restorations (4). Although resin composites offer several advantages as direct restorative materials, including excellent esthetic appearance and excellent clinical performance (5), they still present some limitations. These limitations include polymerization shrinkage, a relatively high coefficient of thermal expansion, and reduced resistance to mechanical wear (6, 7). Resin composite restorations require effective finishing and polishing procedures to improve surface smoothness, minimize plaque accumulation, reduce the risk of staining and wear, and ultimately contribute to the long-term success of the restoration (8, 9).

Physical properties such as surface roughness should be considered one of the most important properties to take care of during the final finishing and polishing procedures. Resin composites are often regarded as artificial surfaces that are prone to the adhesion and accumulation of oral microorganisms (10, 11). For this reason, the restoration surface should be as smooth as possible to minimize biofilm formation. A roughened restoration surface tends to harbor plaque and bacteria, leading to the development of dental caries and the initiation of periodontal diseases (12). This can compromise the restoration's biocompatibility, color stability, and clinical longevity (13, 14). Previous studies have reported that a surface roughness value of <0.2 µm has been suggested as an acceptable threshold for clinical smoothness (11, 15). Additionally, Surface irregularities are also a concern mechanically, as they can act as starting points for microcracks and raise the risk that a restoration will fracture or fail under the repeated stress of chewing (16).

In routine dental practice, prophylaxis and periodontal maintenance visits, including scaling and root planing procedures when indicated, are essential for maintaining periodontal health in both periodontally healthy individuals and those with successfully treated periodontitis (17, 18). These procedures may be performed manually using stainless-steel hand curettes or with piezoelectric ultra-sonic scalers fitted with tips of various materials, including plastic, stainless steel, or titanium (15, 19). Regardless of the instrument used, however, scaling may alter the surface texture of dental restorations, potentially increasing surface roughness and contributing to adverse clinical outcomes (20, 21).

Previous studies have examined the effects of different scaler types on several restorative materials (15, 22), emphasizing the importance of selecting the appropriate scaler for each restoration type. The effects of scaling on restorative materials have been investigated across a range of substrates, including dental ceramics (22), 3D-printed temporary and permanent resin materials (15), glass ionomer restorations (23), and flowable resin composite (24). One study evaluating four ceramic restoration types with three scaler types found that ultra-sonic scalers revealed the greatest surface roughness changes compared with plastic and metal scalers, while plastic scalers caused the least surface alteration (22). Another study investigating 3D-printed resin materials reported that ultra-sonic scaling tips had the greatest effect on restoration surfaces, while results between metal and plastic scaler tips were inconsistent (15). Despite the range of restorative materials used in clinical practice, and to the best of our knowledge, no previous study has directly compared the effects of different scaling instruments on the surface roughness of anterior resin composites.

To address this gap, this study aim is to evaluate the effect of three scaling methods on the roughness of seven different resin composite materials. The selected resin composite brands were chosen based on their worldwide popularity among dental practitioners. Based on the available evidence, it was anticipated that both the type of scaling tip and the composition of the resin composite material could influence the resulting surface roughness. Accordingly, two null hypotheses were formulated: first, that surface roughness of resin composite restorations would not significantly be influenced by the type of scaling tip used; second, that surface roughness would not differ significantly among different anterior resin composites following scaling.

## 2 Materials and methods

### 2.1 Sample size calculation and study groups

In the present study, three different scaling methods were used to evaluate their effects on the roughness of seven dental resin composite materials. Details of the scaling tips and resin composite materials are shown in Table 1.

**Table 1:** The list and composition of the resin composite materials and scaling tips used in the study.

Scaling Tips		
1	Metal Scaler	A stainless-steel metal tip (H3/H4 Jacquette Scaler, Hu-Friedy Mfg. Co., Chicago, IL, USA).
2	Plastic Scaler	A plastic tip composed of Plasteel, a high-grade unfilled resin (Implacare II LG1/2, Hu-Friedy Mfg. Co., Chicago, IL, USA).
3	Ultra-sonic Scaler	A piezoelectric scaler tip (Piezo Scaler Tip 201, KaVo PiezoLED Ultraschall Scaler, Kaltenbach & Voigt GmbH, Biberach, Germany).

Resin Composite Materials		
1	3M™ Filtek™ Z350 XT A2E (3M, Maplewood, MN, USA)	Resins: Bis-GMA, UDMA, TEGDMA, PEGDMA and bis-EMA resins. Fillers: A combination of a non-agglomerated/non-aggregated 20 nm silica filler, a non-agglomerated/non-aggregated 4 to 11 nm zirconia filler and an aggregated zirconia/silica cluster filler (comprised of 20 nm silica and 4 to 11 nm zirconia particles). The Dentin, Enamel and Body shades have an average cluster particle size of 0.6 to 10 microns. - The inorganic filler loading is about 78.5 wt.% (63.3% by volume)
2	Beautifil II A2E (Shofu, San Marcos, CA, USA)	Bis-GMA, TEGDMA, S-PRG filler based on fluoroboro-aluminosilicate glass, Polymerization initiator, Pigments and others.
3	Beautifil II LS A2E (Shofu, San Marcos, CA, USA)	Glass powder, Urethane diacrylate, Bis-MPEPP, Bis-GMA, TEGDMA, Polymerization initiator, Pigments and others.
4	G-ænial Anterior A2 Enamel (© GC America Inc, Alsip, Illinois, USA)	Not Available
5	IPS Empress Direct enamel A2E (Ivoclar Vivadent, Schaan, Liechtenstein)	Resin: Dimethacrylates (20–21.5 wt.%, opalescent shade 17 wt.%). Fillers: fillers contain barium glass, ytterbium trifluoride, mixed oxide, silicon dioxide and copolymer (77.5–79 wt.%, opalescent shade 83 wt.%). The particle size of the inorganic fillers is between 40 nm and 3 µm with a mean particle size of 550 nm. Additional contents: additives, initiators, stabilizers and pigments (<1.0 wt.%).
6	Tokuyama PALFIQUE LX5 A2E (Tokuyama, Yamaguchi, Japan)	Resins: Bis-GMA and TGDMA. Fillers: All inorganic filler is a spherical submicron filler (mean particle size: 0.2 µm, particle size range: 0.1 to 0.3 µm) - Contains 82 wt.% (71% by volume) of silica-zirconia filler and composite filler.
7	Vittra APS FKG enamel A2E (Fort Lauderdale, FL, USA)	Resins: Methacrylate monomers mixture, photo-initiating composition (APS), co-initiators, stabilizer and silane. Fillers: Load particles of zirconia, silica and pigments. - Loading particles are composed of spheres of a zirconia complex with average load particle size of 0.2 micron, with total inorganic load of 72% to 82% in weight (52% to 60% in volume).

**Figure 1:**
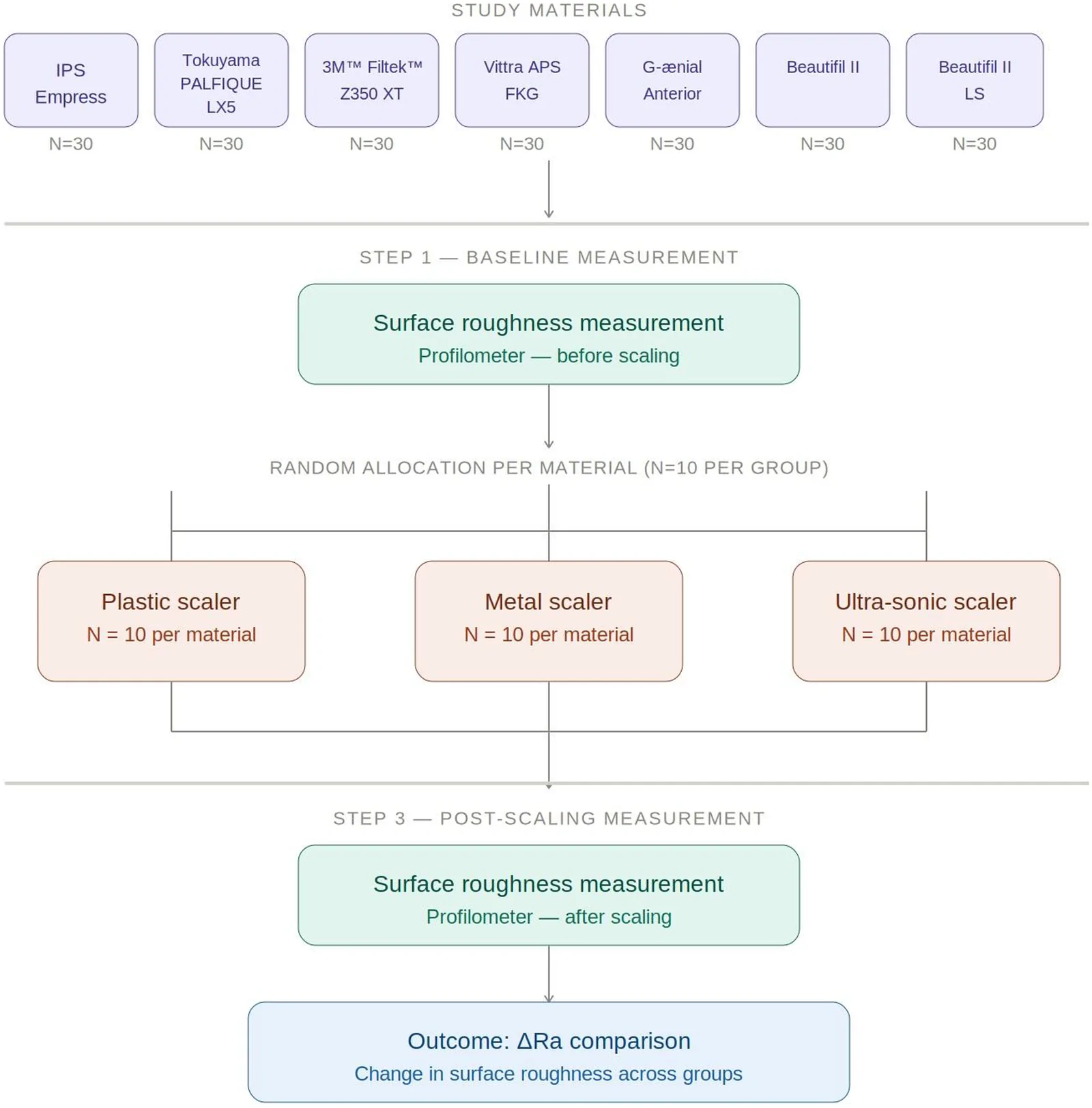
Experimental workflow of surface roughness assessment of restorative materials following instrumentation with different scalers.

Each resin composite material was tested using all three scaling techniques, with 10 specimens assigned to each method and 30 specimens prepared per material. This yielded a total of 210 specimens across 21 experimental groups, as shown in Figure 1. To determine the minimum sample size needed, *a priori* power analysis was conducted using G*Power 3, with the significance level set at *p* < 0.05 and a statistical power of 80%. The results specified that at least nine specimens per group would be sufficient to detect a large effect size (*f* = 0.4). Given that previous studies on surface roughness have typically used ten specimens per group (9, 15, 25). Accordingly, ten specimens were included in each group in the present study.

### 2.2 Sample preparation

Disc-shaped specimens (6 mm in diameter, 2 mm in thickness) were prepared using a 3D-printed mold (NextDent Model 2.0, NextDent B.V., Soesterberg, Netherlands). Each composite material was packed into the mold, after which a celluloid matrix strip was laid over the surface to achieve even distribution. A glass microscope slide was then pressed gently over the strip to hold the specimen in place and avoid any shifting during light curing. Using a halogen light-curing unit (irradiance of 800 mW), all specimens were light-cured for 40 s. Once cured, all specimens were stored in distilled water at 37 °C for 24 h prior to any surface treatment or testing. Then, the specimens of each material were assigned to three experimental groups based on the scaling method applied: ultra-sonic scaling, hand scaling, and air-powder abrasion. Assignment was performed systematically to ensure equal distribution of specimens across all groups and to maintain comparable baseline surface roughness values prior to the intervention. To make paired before-and-after comparisons of surface roughness possible, each specimen was individually labeled and kept in its own container throughout the study.

### 2.3 Surface roughness

Following specimen preparation, the average surface roughness (Ra, µm) of each specimen was examined using a non-contact optical profilometer (Contour GT-K 3D Optical Profiler, Bruker Nano GmbH, Berlin, Germany). A prefabricated mold was used to standardize the location of scanning by guiding measurements to consistent central areas of each specimen. Measurements were obtained from the top surface of each specimen, which was the surface exposed to scaling. To ensure that the same surface was consistently measured throughout the study, specimens were stored in labeled containers with the measured surface facing upward. For each specimen, three readings were recorded at a scanning speed of 0.5 mm/s, with a cutoff value of 0.8 mm, a scan area of 639 μm   ×   479 μm, a lateral resolution of 0.33 μm, a vertical resolution of <0.1 nm, and   ×  20 magnification (15). The mean of the three readings was then calculated and recorded for each specimen.

### 2.4 Scaling methods

All scaling procedures were performed by a single trained and calibrated operator to eliminate inter-operator variability. For the manual scaling groups, plastic scalers were used on 10 specimens and metal scalers on another 10. Each specimen underwent 40 scaling strokes at a 75° working angle, lasting approximately 40 s with moderate lateral pressure applied throughout (26). For the ultra-sonic group, the scaler was run with continuous water irrigation at 13 mL/min and kept at a 0° angle to the specimen surface (27). A lateral force of around 0.2 N was maintained, with the device operating at a frequency of 28–36 kHz and a stroke amplitude of 40 μm (14). All ultra-sonic settings were in line with the manufacturer's instructions for the KaVo PiezoLED Ultra-sonic Scaler (Kaltenbach & Voigt GmbH, Biberach, Germany). Strokes were directed from the base of each specimen upward through the center (27), covering a stroke length of 8 mm for a total of 40 strokes per surface. The scaling track was outlined with marks to maintain consistent alignment with the positioning mold. All scaling tips were replaced after every five specimens to control the impact of wear as a confounder. After scaling, all samples were cleaned using distilled water and sonication. Then, surface roughness was remeasured following the same protocol described above.

### 2.5 Statistical analysis

Statistical analysis was accomplished using SigmaPlot 12.0 (SYSTAT, Chicago, IL, USA). Descriptive statistics, including means and standard deviations, were used to summarize surface roughness values across groups. Differences in surface roughness among the three scaling methods and seven resin composite materials were assessed using two and one-way ANOVA followed by Tukey's *post hoc* test. The Shapiro–Wilk test was applied to verify data normality and confirm the appropriateness of parametric testing. Where normality assumptions were not met, non-parametric alternatives, the Kruskal–Wallis test was employed. Paired t-test was also used to compare between the roughness values before and after scaling at each resin composite type. Statistical significance was set at *p* < 0.05.

## 3 Results

The results of two-way ANOVA are shown in Table 2. Both scaling method and resin composite type were significantly determinants in modulating the roughness of resin composite restorations (*p* < 0.001). A significant interaction between the two factors was observed as well (*p* < 0.001).

**Table 2:** Two-way analysis of variance (ANOVA) to investigate the impact of the scaling methods, resin composite type, and their interaction on the surface roughness of resin composite restorations.

Source of Variation	df	SS	MS	*F*	p
Scaling Method	2	85.60	42.80	137.36	<0.001
Resin Composite Type	6	13.59	2.26	7.27	<0.001
Scaling Method × Resin Composite Type	12	13.37	1.11	3.57	<0.001
Residual	189	58.89	0.31		
Total	209	171.44	0.82		

**Figure 2:**
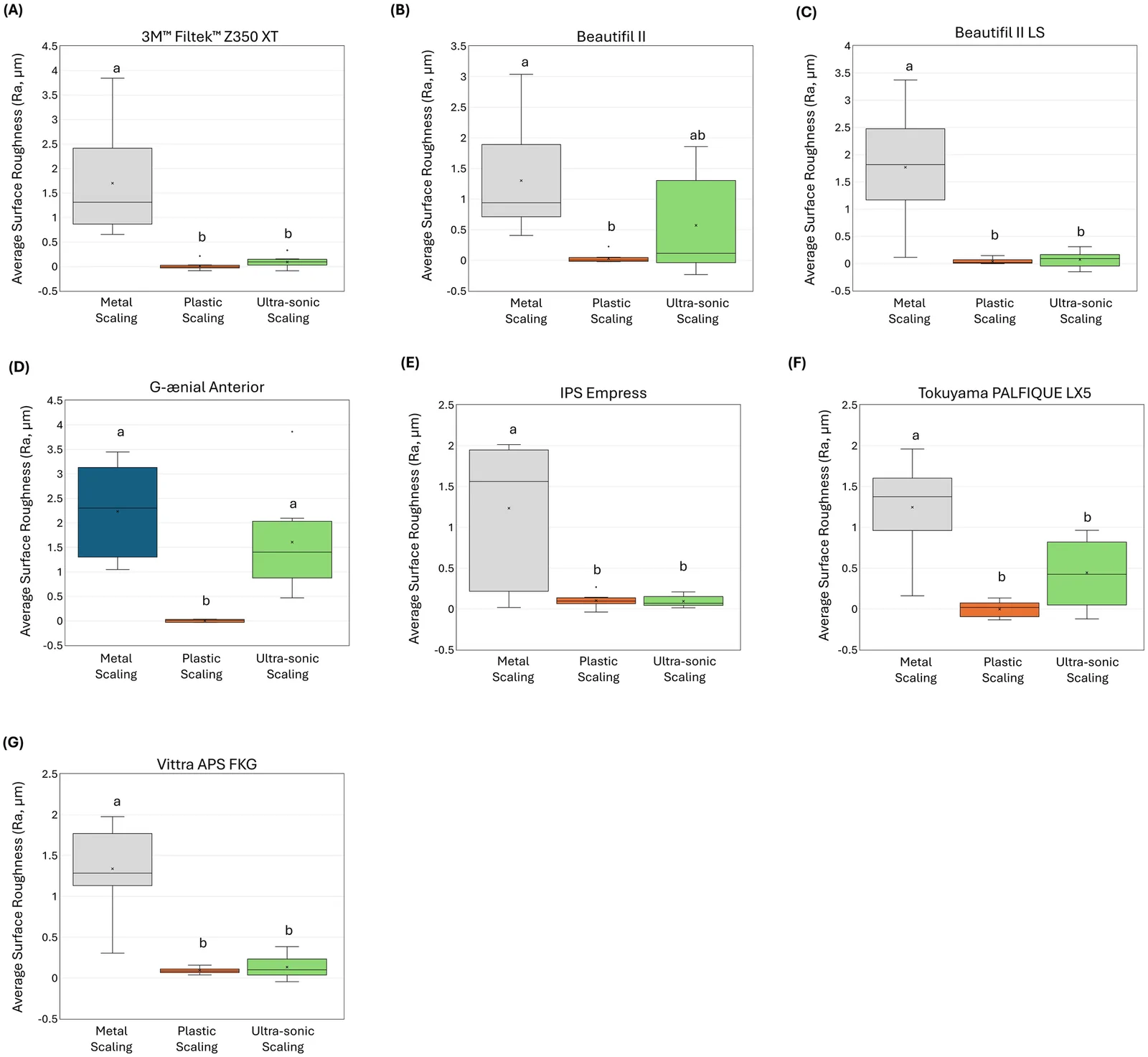
The impact of the different scaling methods on the average surface roughness change (*Δ*Ra,*μ*m) of the resin composite materials: **(A)** 3M™ filtek™ Z350 XT, **(B)** beautifil II, **(C)** beautifil II LS, **(D)** G-ænial anterior, **(E)** IPS empress direct, **(F)** tokuyama PALFIQUE LX5, and **(G)** vittra APS FKG. Different letters indicate significant differences (*p* < 0.05) among the scaling methods.

Figure 2 and Table 3 illustrate the comparison between the different scaler types in each resin composite type using one-way ANOVA. For 3M™ Filtek™ Z350 XT (Figure 2A), Tukey's *post hoc* test showed that metal scaling produced significantly (*p* < 0.05) higher surface roughness (1.70 ± 1.05 µm) than both plastic (0.01 ± 0.08 µm) and ultra-sonic (0.09 ± 0.12 µm) scaling, with no significant difference observed between the plastic and ultra-sonic groups. Similarly, Beautifil II (Figure 2B) exhibited the highest Ra values following metal scaling (1.30 ± 0.85 µm). Ultra-sonic scaling (0.57 ± 0.80 µm) yielded intermediate roughness values, whereas plastic scaling (0.03 ± 0.07 µm) resulted in significantly smoother surfaces (*p* < 0.05). For Beautifil II LS (Figure 2C), Tukey's *post hoc* test displayed that metal scaling (1.76 ± 0.92 µm) significantly increased surface roughness compared to both plastic and ultra-sonic scaling (*p* < 0.05). For G-ænial Anterior (Figure 2D), Tukey's *post hoc* test confirmed that both metal (2.23 ± 0.91 µm) and ultra-sonic (1.61 ± 0.97 µm) scaling produced similarly elevated surface roughness values, with no significant difference between them (*p* > 0.05). While plastic scaling (0.003 ± 0.03 µm), by contrast, resulted in the lowest Ra values. For IPS Empress Direct (Figure 2E), Tukey's *post hoc* test revealed that metal scaling (1.23 ± 0.82 µm) produced significantly higher surface roughness, while both plastic and ultra-sonic scaling yielded similarly low and statistically indistinguishable values (*p* > 0.05). For Tokuyama PALFIQUE LX5 (Figure 2F), Tukey's *post hoc* test showed that metal scaling (1.25 ± 0.53 µm) resulted in the highest Ra values, followed by ultra-sonic scaling (0.44 ± 0.39 µm), which showed moderate roughness; plastic scaling (0.00 ± 0.09 µm) produced the lowest surface roughness than both (*p* < 0.05). For Vittra APS FKG (Figure 2G), Tukey's *post hoc* test showed that metal scaling (1.34 ± 0.48 µm) significantly increased surface roughness, whereas both plastic and ultra-sonic scaling resulted in comparable Ra values (*p* < 0.05).

**Table 3:** One-way ANOVA of the effect of scaling method on surface roughness (Ra) within each resin composite.

Resin Composite	F value	df (between)	df (within)	*p* value
3M™ Filtek™ Z350 XT	24.112	2	27	<0.001
Beautifil II	8.856	2	27	0.001
Beautifil II LS	33.401	2	27	<0.001
G-ænial Anterior	22.201	2	27	<0.001
IPS Empress Direct	18.862	2	27	<0.001
Tokuyama PALFIQUE LX5	27.396	2	27	<0.001
Vittra APS	61.227	2	27	<0.001

One-way ANOVA (*α* = 0.05). Significance.

**Figure 3:**
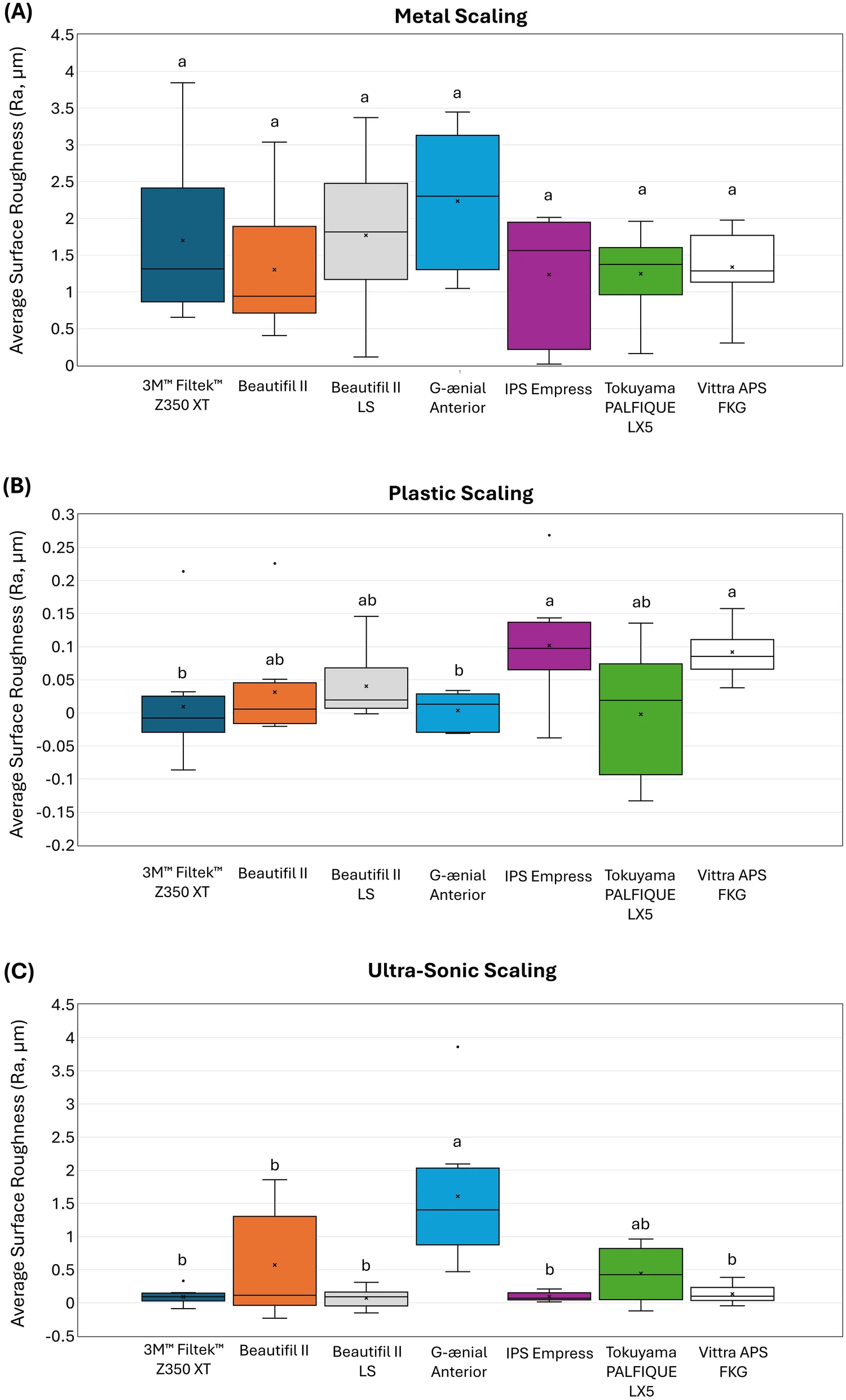
A comparison of the average surface roughness change (*Δ*Ra, μm) of the resin composite materials following each scaling method: **(A)** metal scaler, **(B)** plastic scaler, and **(C)** ultra-sonic scaler. Different letters indicate significant differences (*p* < 0.05) among the scaling methods.

Figure 3 and Table 4 compare resin composite materials within each scaling method using one-way ANOVA. Clear material-dependent differences in surface roughness were observed. Under metal scaling (Figure 3A), G-ænial Anterior (2.23 ± 0.91 µm) exhibited the highest surface roughness among all materials tested, while IPS Empress Direct (1.23 ± 0.82 µm) showed the lowest Ra values. Under plastic scaling (Figure 3B), all materials showed low surface roughness values. However, slight variation among the tested materials was observed, with IPS Empress Direct (0.10 ± 0.08 µm) and Vittra APS FKG (0.09 ± 0.04 µm) displaying relatively higher roughness values than other groups, and Tokuyama PALFIQUE LX5 revealing the lowest roughness. Under ultra-sonic scaling (Figure 3C), G-ænial (1.61 ± 0.98 µm) recorded the highest surface roughness.

**Table 4:** One-way ANOVA of the effect of resin composite type on surface roughness (Ra) within each scaling method.

Scaling Method	F value	df (between)	df (within)	*p* value	Significance
Metal Scaling	2.074	6	63	0.069	NS
Plastic Scaling	3.989	6	63	0.002	**
Ultra-sonic Scaling	11.973	6	63	<0.001	***

One-way ANOVA (*α* = 0.05). Significance:.

Table 5 presents the baseline and post-scaling surface roughness (Ra) values and the statistical analysis using paired t-test. At baseline, all seven composites exhibited comparably low roughness (Ra ≈ 0.16–0.20 µm) with no meaningful differences among groups. Metal scaling produced the most pronounced surface deterioration, significantly increasing Ra for all seven composites (*p* < 0.01), with the largest changes recorded for G-ænial Anterior and Beautifil II LS. Following ultra-sonic scaling, significant increases were observed for 3M Filtek Z350 XT, G-ænial Anterior, IPS Empress Direct, Tokuyama PALFIQUE LX5, Vittra APS, and Beautifil II (*p* < 0.05), whereas Beautifil II LS (*p* = 0.15) showed no significant change. Plastic scaling had the least impact, with no significant roughening for 3M Filtek Z350 XT, Beautifil II, G-ænial Anterior, or Tokuyama PALFIQUE LX5 (*p* > 0.05); only Beautifil II LS, IPS Empress Direct, and Vittra APS showed significant changes.

**Table 5:** The baseline and post-scaling roughness values (Ra, μm) following the scaling (mean ± SD). Asterisk indicates significant difference by paired t-test.

Brand Name	Metal Scaling		Plastic Scaling		Ultra-sonic Scaling	
	Before	After	Before	After	Before	After
3M™ Filtek™ Z350 XT	0.18 ± 0.05	1.88 ± 1.03*	0.17 ± 0.03	0.18 ± 0.08	0.18 ± 0.05	0.27 ± 0.12*
Beautifil II	0.20 ± 0.07	1.50 ± 0.83*	0.20 ± 0.05	0.23 ± 0.05	0.18 ± 0.07	0.75 ± 0.92*
Beautifil II LS	0.19 ± 0.04	1.96 ± 0.98*	0.19 ± 0.06	0.23 ± 0.08*	0.20 ± 0.06	0.27 ± 0.15
G-ænial Anterior	0.16 ± 0.05	2.40 ± 0.99*	0.17 ± 0.04	0.18 ± 0.04	0.16 ± 0.04	1.77 ± 1.05*
IPS Empress Direct	0.17 ± 0.07	1.41 ± 0.87*	0.16 ± 0.05	0.26 ± 0.07*	0.18 ± 0.04	0.27 ± 0.12*
Tokuyama PALFIQUE LX5	0.20 ± 0.06	1.45 ± 0.52*	0.17 ± 0.06	0.17 ± 0.07	0.19 ± 0.05	0.62 ± 0.50*
Vittra APS FKG	0.16 ± 0.07	1.50 ± 0.55*	0.18 ± 0.03	0.27 ± 0.04*	0.18 ± 0.06	0.32 ± 0.12*

## 4 Discussion

The aim of this study was to investigate the change in surface roughness associated with three different scaling methods (metal scaling, plastic scaling, and ultra-sonic scaling) applied to seven anterior resin composite materials. The results demonstrated that both the scaling method and the composite material significantly influenced the resulting surface roughness. Metal scaling produced the most pronounced roughening across all materials, ultra-sonic scaling caused intermediate and material-dependent effects, and plastic scaling was the most surface-conserving method, leaving several resin composites below the clinically relevant 0.2 µm threshold. Accordingly, both null hypotheses, that scaling method has no effect on surface roughness, and that material type has no effect, were rejected.

Among all tested resin composite materials, the type of scaling instrument had a clear and significant effect on surface roughness. Metal scalers consistently produced the greatest surface alteration, followed by ultra-sonic and then plastic-tip scalers, which is consistent with the findings of Checketts et al., who reported similar results on gold alloy restorations (21). The increased surface roughness associated with metal scalers can be attributed to their mechanical characteristics. Metal scaler tips are constructed from stainless steel, which has a significantly greater hardness than the organic matrix and filler particles of resin composite restorations. During scaling, direct contact between the metal tip and the composite surface generates high stress concentrations at a localized area, resulting in mechanical abrasion and resin matrix's removal (15).

On the other hand, ultra-sonic scaler tips produced less surface alteration than metal instruments, a finding also supported by Ankily et al., who demonstrated that metal scaling on enamel surfaces causes greater surface change than ultra-sonic scaling (28). Opposingly, Ismail et al. looked at manual and ultra-sonic scalers across a range of base materials and found that ultra-sonic scaling induced more surface roughness than manual scaling (29), a discrepancy that may reflect differences in tip design and power settings. In this study, ultra-sonic scaling produced intermediate levels of surface roughness, exceeding that associated with plastic-tip scalers while remaining significantly lower than that induced by metal scaling. This is in agreement with Mittal et al. who found that curette instruments left behind more surface damage to the roots of the tested teeth, while ultra-sonic scaling produced fewer scratches (30). A comparable finding was reported in a study on zirconia implant-supported crowns, where metallic scalers were associated with notably aggressive surface changes, a result that agrees with the pattern observed in the present study (31).

In this study, plastic scaler tips produced the smoothest surfaces compared to both ultra-sonic and metal scalers. This is consistent with findings from a study on four CAD/CAM ceramic materials, in which ultra-sonic scaler tips resulted in the greatest surface changes while plastic scalers produced the smoothest surfaces across all tested ceramics (22). Similarly, a study on resin nano-ceramic materials reported that plastic tips yielded the lowest mean surface roughness and caused the least surface damage (32). The minimal surface roughening observed with plastic scaling can be primarily attributed to the low hardness of the plastic instrument relative to the resin composite surface. Manual plastic scalers are mainly manufactured from high-performance polymers. These polymers' hardness is considerably lower than that of both the resin matrix and embedded inorganic filler particles that reinforce the composite. Therefore, during scaling, the plastic instrument itself tends to wear what contacts resin composites, dissipating the applied force without producing substantial surface alteration.

A previous research has shown that resin composites do not all respond similarly to the same surface treatment, highlighting that resin composite's composition may play an important role (33). The changes in surface roughness observed among the tested resin composites could be due to filler loading and particle distribution. Filler content is strongly associated with mechanical performance, as higher filler loading usually contributes to higher strength. Furthermore, resin composites containing smaller, well-distributed particles tend to exhibit smoother surfaces. Opposingly, larger or poorly distributed fillers may raise surface roughness. The observed differences in roughness among the tested materials are therefore unlikely to be explained by filler content alone, as the shape of the filler particles and how well they interact with the surrounding resin matrix probably play an equally important part (34, 35).

In general, G-ænial Anterior showed the highest surface roughness of all materials tested, followed by Beautifil II LS and 3M™ Filtek™ Z350 XT. Although its chemical composition is not fully disclosed, the greater susceptibility of G-ænial Anterior to surface roughening may be related to its hardness. Pala et al. reported lower microhardness values for it compared to two other resin composites tested under the same conditions (36). Tokuyama PALFIQUE LX5, by contrast, proved to be among the most resistant materials under metal scaling. It contains a high inorganic filler loading of 82 wt.%, composed of spherical, submicron silica-zirconia particles with a mean size of 0.2 µm. This high filler content reduces the proportion of exposed resin matrix at the surface, thereby protecting the softer matrix from wear and dislodgment during scaling. In addition, unlike irregular or angular particles, rounded spherical fillers present fewer points at which a metal cutting edge can engage and dislodge them.

Following plastic scaling, both IPS Empress Direct and Vittra APS showed increased roughness compared to the other resin composites. The roughness change illustrated in IPS Empress Direct could be attributed to its relatively soft barium glass fillers and broad particle size distribution (up to 3 µm), where abrasion force may create detectable surface irregularities (37). While Vittra APS has the lowest filler volume fraction among the tested materials (52–60 vol.%), which may expose the resin matrix and make it more vulnerable to abrasion. In ultra-sonic scaling, G-ænial Anterior demonstrated a significantly greater increase in roughness than all other materials except Tokuyama PALFIQUE LX5. The manufacturer of this brand does not disclose its full chemical composition, making it difficult to relate these findings to specific compositional features. On the other hand, materials with densely packed, well-silanized inorganic fillers of small and uniform size, such as the nanofilled Filtek Z350 XT, were found to resist abrasion from ultra-sonic scaling, suggesting that resin composite formulations with high filler loading and nano-sized particles are more capable of resisting abrasion (34, 35).

It is worth noting that a surface roughness value of around 0.2 µm is generally regarded as the upper limit of clinical acceptability (11). Once surfaces exceed this threshold, they tend to accumulate more biofilm and become more susceptible to bacterial adhesion (11). In the present study, all three scaler types altered surface roughness to some degree, which is consistent with what has been reported elsewhere in the literature (15, 21). Checketts et al. examined microbial adhesion on zirconia, lithium disilicate, and gold alloy restorations instrumented with different scalers, and found that lithium disilicate showed a notable increase in surface roughness after scaling, which was accompanied by greater bacterial adhesion compared to untreated controls (21). In this study, metal and ultra-sonic scaling raised all seven materials above the 0.2 µm threshold, with post-scaling roughness ranging from 1.41 to 2.40 µm and from 0.27 to 1.77 µm, respectively. On the other hand, plastic scaling was the most surface-conserving method, and only four composites exceeded 0.2 µm, which were Vittra APS, IPS Empress Direct, and Beautifil II and Beautifil II LS, with post-scaling roughness ranging from 0.17 to 0.27 µm. These findings reinforce the importance of thoughtful scaler selection during scaling and root planing procedures, with plastic scalers being the only method able to keep certain materials within the clinically acceptable range. Maintaining restoration surface integrity matters not only for esthetic and mechanical reasons, but also for reducing the kind of surface irregularities that give plaque somewhere to accumulate and bacteria somewhere to settle.

This study has a few limitations that should be considered. First, specimens were not repolished after scaling, which means the findings may not fully capture what occurs clinically when scaling is followed by a polishing step. Future studies would benefit from examining how different post-scaling polishing protocols influence the final surface outcome. In addition, future studies may consider investigating the cleaning effectiveness of these scalers when used against resin composite restorations. Second, manual scaling was performed by a single operator, and despite efforts to remain consistent, some variation in applied force cannot be entirely ruled out. This could be addressed in future work by incorporating mechanical rigs or standardized devices to achieve more controlled and reproducible force delivery. Third, only a selected range of commercially available resin composites was included, so the findings should not be broadly extended to all composite materials available on the market. Finally, the study measured surface roughness only and did not account for other clinically relevant outcomes, such as color stability, surface gloss, or material behavior under aging or immersion conditions. In addition, other surface roughness parameter, such as volume loss and total profile height, were not investigated. These remain important areas for future investigation, particularly surface morphology analysis using scanning electron microscopy (SEM) and evaluation of microbial adhesion patterns. Within the limitations of the study, it is concluded that both the type of scaling instrument and the composite material played a clear role in determining surface roughness. Metal scaling caused the greatest surface disruption of the three methods, plastic scaling caused the least, and ultra-sonic scaling sat somewhere in between. Not all materials responded equally as G-ænial Anterior was the most prone to roughening, while IPS Empress Direct was associated with the least roughness. In clinical practice, plastic scalers would seem to be the better option during routine maintenance of composite restorations, as they are more likely to preserve surface integrity and support long-term esthetic stability.

## Data Availability

The original contributions presented in the study are included in the article/Supplementary Material, further inquiries can be directed to the corresponding author.
