# Supplementary material for: Effect of different scaling instruments on the roughness of esthetic resin composite restorations: an in vitro study
Source: Front Dent Med. 2026 Aug 28;7:1894177. doi: 10.3389/fdmed.2026.1894177 (PMC13561887; doi:10.3389/fdmed.2026.1894177)
Supplement: Supplementary file 1 [file Datasheet1.zip › 3M_Filtek_z350xt.pdf]

3M ESPE

Filtek™ Z350 XT
Universal Restorative System

technical product profile

Filtek™

Table of Contents

INTRODUCTION **1**

PRODUCT DESCRIPTION **2**

Indications for Use 2

Composition 2

Shades 3

Fluorescence and Opalescence 4

Shade Basics 4

Color 4

Opacity Considerations 5

Opinion Leaders 6

Shade Selection Tips and Hints for Filtek™ Z350 XT Universal Restorative 6

Shade Wheel 8

BACKGROUND **10**

Fillers 10

Microfills 10

Hybrids, Microhybrids and Nanohybrids 11

Nanocomposites 12

Filler Improvements in Filtek™ Z350 XT Universal Restorative 13

Resin System 14

In Vitro Handling Evaluations 14

PHYSICAL PROPERTIES **16**

Polish Retention 16

Toothbrush Abrasion 16

Wyko Images 18

3-Body Wear 22

Fracture Toughness 23

Compressive and Diametral Tensile Strength 24

Flexural Strength and Modulus 25

Volumetric Shrinkage 26

FIELD EVALUATION **27**

Handling Acceptability 28

QUESTIONS AND ANSWERS **31**

ENDNOTES **32**

TECHNICAL DATA SUMMARY **33**

Introduction

Building upon over 40 years of innovation in the field of restorative dentistry, 3M ESPE created, at the turn of this century, a new category of dental material—the nanocomposite.

Through precise manipulation of the filler architecture at the nanoscale, 3M ESPE developed a breakthrough composite filling material, 3M™ ESPE™ Filtek™ Supreme Universal Restorative, that significantly advanced the clinical performance of universal composites. Up until the launch of this product, dentists desiring the highest esthetics in direct composite restorations chose microfills. Microfills were considered the gold standard in esthetics, however, their lack of strength, wear resistance and radiopacity limited their use to only select anterior restorations. Hybrid composites had high filler loading, but the average particle size was in the submicron range which somewhat limited their esthetic qualities. Hybrid composites provide the strength, wear resistance and radiopacity necessary for anterior and posterior use. In 2002, 3M ESPE launched Filtek Supreme restorative. This was the first product that utilized nanotechnology to provide the esthetics of a microfill and the strength of a hybrid. All of the filler particles in this novel composite are engineered nanoparticles.[1] This technology provided lasting polish, excellent handling and wear similar to enamel.[2]

Subsequently, as a result of feedback from dentists, Filtek™ Supreme XT Universal Restorative was launched in 2005. The shades were optimized to provide more vibrant, lifelike restorations as a result of increasing the value or brightness of the shades.

Since the original introduction of Filtek Supreme restorative, 3M ESPE has continued discussions with opinion leaders and general dentists regarding potential improvements desired. Based on these discussions, focus groups and other market research methods, additional improvements are realized with the introduction of Filtek™ Z350 XT Universal Restorative. The following improvements have been made to the system:

Easier to use shading system

- Color-coding by opacity
- New, easier-to-read labeling
- Extended Body shade range

Even better polish retention

Improved fluorescence

Supreme-like handling for all opacities

Improved Translucent shades

- Better Translucent shade handling
- Availability of Translucent shades in capsules
- Modified Translucent shade offering
- Translucent shades are radiopaque

Product Description

3M™ ESPE™ Filtek™ Z350 XT Universal Restorative is a visible light-activated composite designed for use in anterior and posterior restorations. All shades are radiopaque. A dental adhesive, such as those manufactured by 3M ESPE, is used to permanently bond the restoration to the tooth structure. The restorative is available in a wide variety of Dentin, Body, Enamel and Translucent shades. It is packaged in syringes and single-dose capsules.

Indications for Use

Filtek Z350 XT restorative is indicated for use in:

- Direct anterior and posterior restorations (including occlusal surfaces)
- Core build-ups
- Splinting
- Indirect restorations (including inlays, onlays and veneers)

Composition

The resin system is slightly modified from the original Filtek™ Z250 Universal Restorative and Filtek™ Supreme Universal Restorative resin. The resin contains bis-GMA, UDMA, TEGDMA, and bis-EMA(6) resins. To moderate the shrinkage, PEGDMA has been substituted for a portion of the TEGDMA resin in Filtek Supreme XT restorative.

The fillers are a combination of non-agglomerated/non-aggregated 20 nm silica filler, non-agglomerated/non-aggregated 4 to 11 nm zirconia filler, and aggregated zirconia/silica cluster filler (comprised of 20 nm silica and 4 to 11 nm zirconia particles). The Dentin, Enamel and Body (DEB)[3] shades have an average cluster particle size of 0.6 to 10 microns. The Translucent (T)[4] shades have an average cluster particle size of 0.6 to 20 microns. The inorganic filler loading is about 72.5% by weight (55.6% by volume) for the Translucent shades and 78.5% by weight (63.3% by volume) for all other shades.

Shades

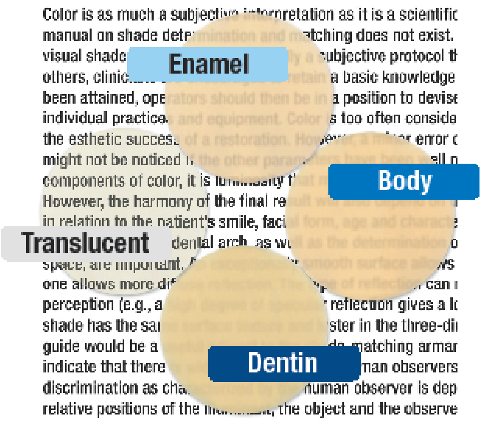

Figure 1: Opacity Choices

The system is comprised of four opacities, listed here in decreasing order of opacity: Dentin (most opaque), Body, Enamel and then Translucent (very transparent). The opacity differences are illustrated in Figure 1. The clarity of the print under the 1 mm composite discs exhibits the opacity. The Translucent shades are very clear, hence the print appears relatively unchanged from the surrounding type. The Enamel shades have opacity similar to tooth enamel. The print is slightly fuzzy but very readable through the disc. The Body shades are slightly more opaque, less translucent than the Enamel shades to enable use in single shade restorations. The print is still readable but very fuzzy. Dentin shades have the highest opacity. In multi-shade restorations, the Dentin shades are used to replace the more opaque dentin tooth structure, alter underlying dentin color and block shine-through in anterior restorations.

The shade system is based on the VITAPAN Classical shade system with the following exceptions:

For bleached teeth: White Dentin, Body and Enamel (WD, WB, WE),
Extra White Body and Enamel (XWB and XWE)

For cervical restorations: A6B and B5B

Translucent shades: Clear, Blue, Gray and Amber

Filtek™ Z350 XT Shade Offering

Dentin	Body	Enamel	Translucent
A1D	A1B	A1E	Clear
A2D	A2B	A2E	Blue
A3D	A3B	A3E	Gray
	A3.5B		Amber
A4D	A4B		
	A6B		
	B1B	B1E	
	B2B	B2E	
B3D	B3B		
	B5B		
	C1B		
	C2B		
	C3B		
C4D			
	D2B	D2E	
	D3B		
WD	WB	WE	
	XWB	XWE	

The shade offering was modified from Filtek™ Supreme XT Universal Restorative. The differences in the shade offering include a reduction in the Dentin shades (eliminated A6D, C6D, XWD). The Body shade offering was broadened by adding A6B and B5B for cervical restorations and D3B. An Enamel shade, XWE, was also added. Additionally, the Violet and Yellow Translucent shades were replaced with Blue and Amber Translucent.

The chart to the left also demonstrates the color coding used in the Filtek Z350 XT Universal Restorative system. The darker the color code, the more opaque the composite.

Fluorescence and Opalescence

Two additional esthetic properties of natural dentition are fluorescence and opalescence. It is thought that both of these properties contribute to the vitality and lifelike appearance of dentition. In natural teeth, dentin (more specifically the hydroxyapatite minerals and organic matrix) exhibits higher fluorescence than enamel. Fluorescence occurs when energy is absorbed and emitted at a longer wavelength. In teeth, this means the absorption of light in the UV region (350-365 nm) and emitting light in the visible region (~400 nm).[5] As shown in Figures 2 and 3, teeth fluoresce blue-white. Note that some materials fluoresce more than the natural tooth while others, e.g., Filtek™ Z350 XT Universal Restorative, fluoresce at a similar level and color.[6]

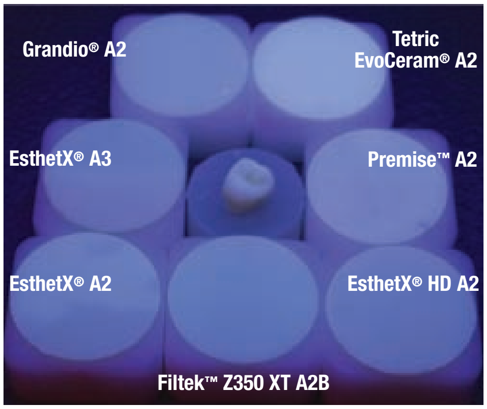

Figure 2: Dentin, Enamel or Body Shades

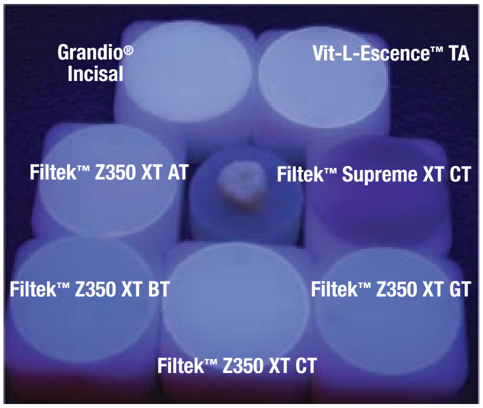

Figure 3: Translucent or Incisal Shades

Opalescence, on the other hand, is related to how a material scatters the shorter wavelengths of light. This is demonstrated by a bluish appearance under reflected light and orange/brown under transmitted light.[7] Natural enamel exhibits an opalescent effect. By changing the nanocluster used, the Translucent shades of Filtek Z350 XT restorative were specially formulated to provide the opalescence in the range of literature values for human enamel.[8]

Shade Basics

Color

- Hue—The actual color of the material. The bar below demonstrates the hues from blue to yellow.

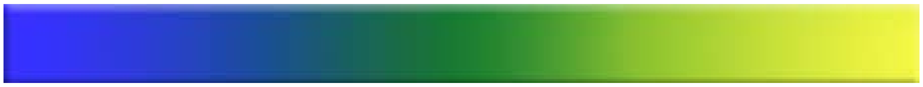

- VITAPAN Classical Shade Guide Shade Family (Hue)

A shades	Red-brown character
B shades	Red-yellow character
C shades	Gray character (lower value)
D shades	Red-gray character (lower value)

- Chroma is the intensity of the shade. The higher the number (e.g., A3 vs. A1) within a shade family, the more intense the color (A3 is more intense than A1).

- The value (amount of white or black) is higher (whiter) for the A and B shades. The C and D shades have lower value (grayer) than the A and B shades. Very generally speaking, the C shades are lower-value A shades. Value is often thought of as the most important color aspect.

Studies have indicated that tooth color, in adult teeth, is determined primarily by dentin. The enamel layer plays a very minor role in the actual tooth color.

- In young patients, teeth are brighter (higher value) and less translucent. With age, the enamel layer thins, exposing more dentin so teeth appear darker, particularly in the gingival third.

- Areas of highest intensity of color (chroma) will be in the gingival region of the tooth due to a thinner enamel layer so the dentin is more visible.

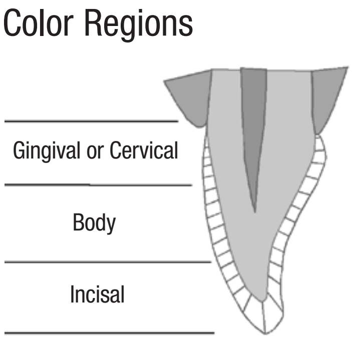

- The Body region is a combination of the dentin color and the slight contribution by the enamel layer color and surface morphology. Literature suggests that the intensity of the body is 1-2 shades lighter than the gingival area.

- The incisal area exhibits a high degree of translucency as the amount of dentin present is decreased toward the incisal edge.

Opacity Considerations

When light contacts a tooth:

- Enamel diffuses and transmits light. If the dentin layer is very thin or if there is no dentin behind the enamel layer (as in the incisal edge), some of the light is transmitted through the tooth to the oral cavity. The oral cavity can reflect light back through the enamel.

- When light encounters dentin, some of the light is absorbed and some is reflected back through the enamel.

- The light that is reflected and refracted back to the eye produces the color of the tooth.

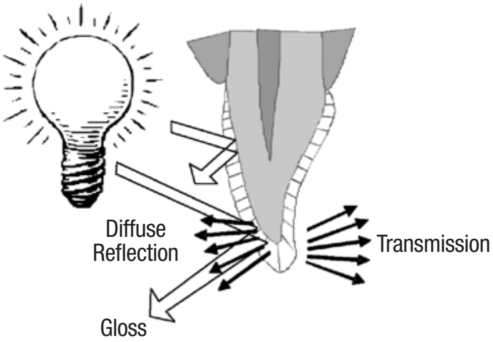

- The surface texture of a tooth plays a role in the perceived color, i.e., a smoother surface will appear whiter (or higher value) than an irregular surface.

Opinion Leaders

Eighteen opinion leaders were invited to share their views regarding case presentations (recommended treatment and technique), education methods, strengths and weaknesses of current composites, and shade guide offerings. Key findings are detailed below.

- It is important to gain agreement between patient and dentist as to the level of esthetics required.

- Shade guides can be used to start the shade selection process; however, shade mock-ups, using the composite in vivo are the best way to determine which composite shades are needed to blend with surrounding dentition.

- The acceptability of the resultant restorations can be influenced by many factors including shades selected, depth of color and re-creation of the natural tooth appearance (e.g., provided by layering translucent materials over less translucent materials or creating appropriate translucency along incisal edge), surface polish and surface morphology, and patients' and dentists' preferences.

Shade Selection Tips and Hints for Filtek™ Z350 XT Universal Restorative

1. After pumicing the surface to remove any extrinsic stains, determine the shades needed for the restoration prior to tooth preparation or rubber dam placement. A tooth that is desiccated will be lighter than normal. Therefore, a shade taken on a desiccated tooth will be lighter than the tooth upon rehydration.

2. During shade selection,

   - If one shade is to be used,

     – Select the Body shade by examining the center (body) portion of the tooth. Choose the composite shade most closely approximating the center portion of the VITAPAN classical tooth tab.

- If more than one shade is to be used to mimic actual tooth structure and increase the vitality of the final restoration, either use the Shade Wheel (next page) or identify which opacities are to be used. To determine which shade to choose in a given opacity:

  – Select the Dentin (or Body) shade by examining the exposed dentin or the gingival area of the tooth. Choose the composite shade most closely approximating the cervical portion (grinding off the neck of the tab has been recommended by some) of the VITAPAN classical tooth tab.

  – Select the Body shade by examining the center (body) portion of the tooth. Choose the composite shade most closely approximating the center portion of the VITAPAN classical tooth tab.

  – Select the Enamel shade by examining the proximal or incisal area of anterior teeth, or from the cusp tips of posterior teeth. Choose the composite shade most closely approximating the center portion of the VITAPAN classical tooth tab.

  – A Translucent shade (in the same color family) may be used to impart high translucency and increase the “depth” of the restoration.

3. Do a mock-up of the restoration prior to etching. The color of a composite will be affected by its thickness. Composites may change color upon curing. Place and cure composite material in the approximate thickness and area of the planned restoration. Obtain agreement with the patient of shade match. Remove mock-up easily by flicking it off the tooth with an explorer.

4. Evaluate shade match of the tabs and mock-up under different lighting conditions.

5. When finishing and polishing the restoration, mimic the surface morphology of adjacent teeth.

Shade Wheel

To aid in the shade selection process, 3M™ ESPE™ Filtek™ Z350 XT Universal Restorative incorporates a unique (patented) shade selector wheel. Once a shade has been selected using the VITAPAN Classical guide, the selector offers recommendations for single shade, two-shade or multi-shade restorations in supported and unsupported restorations. (Figure 4)

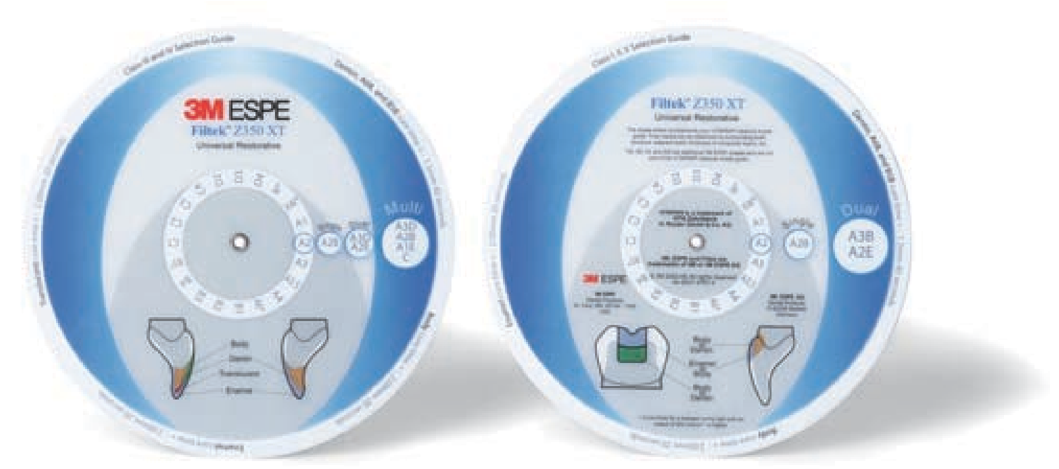

Figure 4: Shade Wheel – Unsupported and Supported Restoration

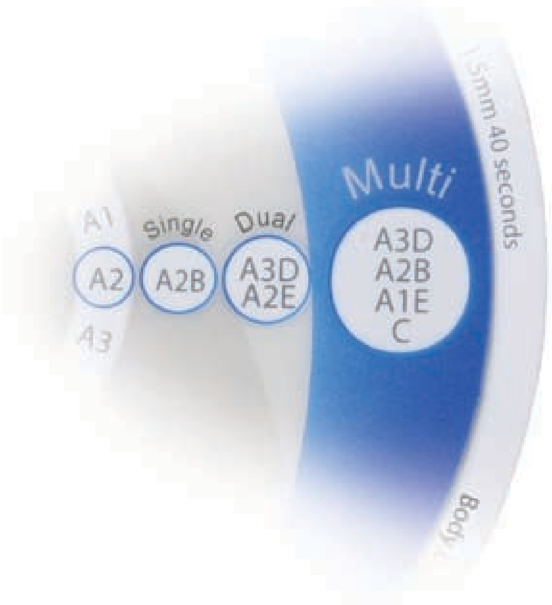

Figure 5: Shade Recommendation

Figure 5 indicates the proposed shade combinations for a Class IV and other unsupported restorations determined to be shade A2. Several options are offered, with final choice depending upon the size and esthetic requirements of the restoration.

Simpler shade recommendations are given for restorations that are supported by tooth structure. Posterior restorations are an ideal place to start exploring the esthetic options offered by the shade layering technique.

To use the wheel:

- Select VITAPAN shade: Choose the composite shade most closely approximating the center portion of the VITAPAN classical tooth tab.

- Select the appropriate wheel side that corresponds to the type of restoration – e.g., supported or unsupported. (Figure 4)

- Rotate the disc so that the VITAPAN shade is visible in the innermost circle.

- Follow the Filtek Z350 XT restorative shade combination recommendations outlined for Single, Dual or Multi shades. (Figure 5)

It should be noted that this tool is a guide only. Final results will be influenced by the thickness of composite layers, surrounding tooth structure, adjacent teeth, etc. Further, the layering diagrams depicted on the shade guide are offered as potential solutions in creating certain esthetic effects. For instance, the Translucent shade may be applied internally as indicated to create translucency at the incisal third of a Class IV restoration. Alternatively, while not diagrammed, the Translucent shade may be applied as the last facial or occlusal increment to create depth. As using the Translucent shade in this manner may tend to decrease the overall value of the restoration, choosing a shade one step lighter for the increment immediately below the Translucent shade may moderate this effect.

Background

Fillers

Microfills

Traditional microfills are made from fumed silica, prepared by a pyrogenic process, with an average particle size of 0.04um. Typically, the primary particles tend to aggregate (the degree of aggregation varies, depending on the filler used in the microfill product). Breakdown of any aggregated particles into smaller entities is difficult, if not impossible, to achieve. The structure of these aggregates results in relatively low filler loading.

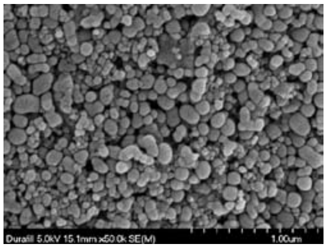

Figure 6: Durafill® VS

In the SEM (Figure 6), courtesy of Dr. Jorge Perdigao,[9] the surrounding resin matrix was removed with a solvent.[10] The field of view of this SEM did not include any of the prepolymerized filler, but focused instead on the individual silica aggregates. Note that the particles appear to be in the 0.1um range, significantly larger than 0.04 microns as a result of the aggregation. Most microfill manufacturers add prepolymerized filled resin particles to increase filler loading. Prepolymerized filler is made by adding the fumed silica filler to resin. The mixture is polymerized and then ground to form particles. These ground particles are added to more resin and fumed silica filler. Even by using this process, microfills still have a substantially lower filler loading than hybrids resulting in lower strength and wear resistance.

Residual methacrylate groups bind the prepolymerized particles to the resin matrix. The effectiveness of this bond is impacted by the amount of residual double bonds on the surface of these particles. During the polymerization of the prepolymerized filler the reaction is driven to near completion. Hence, the bond of the prepolymerized filler particles to the resin is weaker than desired and breakdown frequently occurs at this interface. Additionally, traditional microfills containing only silica filler are not radiopaque. These properties have limited the usefulness of microfills, particularly in the posterior area.

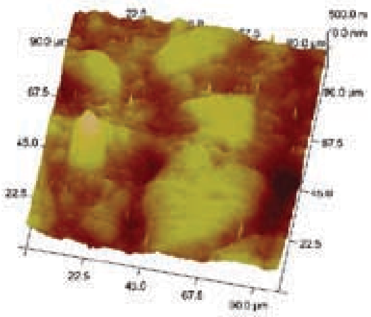

Figure 7: Durafill® VS

The AFM[11] (Figure 7) is a 3-D image of the surface of a microfill after 6000 cycles of toothbrush abrasion. Microfills have proven to retain their polish (surface reflectivity) over time. The prepolymerized filler particles are marginally more wear resistant than the surrounding matrix, resulting in small surface irregularities.

Hybrids, Microhybrids and Nanohybrids

Hybrids, microhybrids and nanohybrids contain a broad distribution of particle sizes. A wide distribution of particle sizes can lead to high filler loading with resultant high strength and wear resistance. While they contain a small fraction of filler particles in the nanoparticle size range (less than 0.1µ or 100 nm), they also contain a range of substantially larger filler particles which influences the optical properties of these composites and detracts from polish retention. (Figure 8) The average particle size of hybrids, microhybrids and nanohybrids is typically below 1 micron, but above 0.2 microns. The larger particle sizes can extend to well over 1 micron. They are typically manufactured by grinding or milling large fillers into smaller particles. The nanohybrids have some particles in the nanofiller size range less than 100 nm (0.1um), but they also contain particles in the submicron range (0.2 to 1µ).

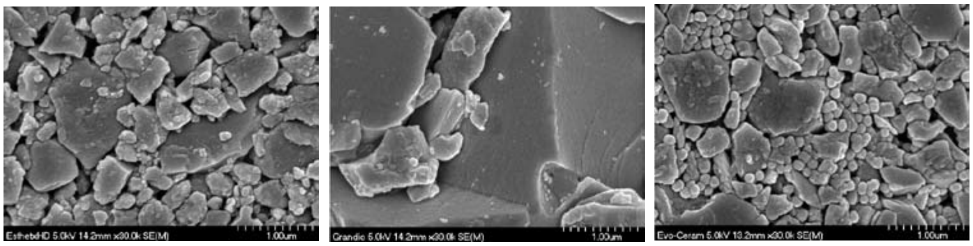

Figure 8: EsthetX® HD, Grandio®, Tetric EvoCeram® (L to R)

When any of these materials are subjected to abrasion, the resin between and around the particles is lost, leading to protruding filler particles (bumps). Eventually the entire filler particle is plucked from the surface, resulting in craters. These bumps and craters create a roughened surface, resulting in loss of reflectivity (loss of polish retention) of the composite surface. The AFM images[12] (Figure 9) show the influence of the large- to small-particle ratios and the number of sizes of the particles after the surface has been toothbrush abraded. The material shown in the far right contains prepolymerized fillers which are typically larger than the typical inorganic fillers. Note the roughness is clearly shown by the many peaks and valleys. The materials in the SEMs shown above correspond to the materials in the AFM images shown below.

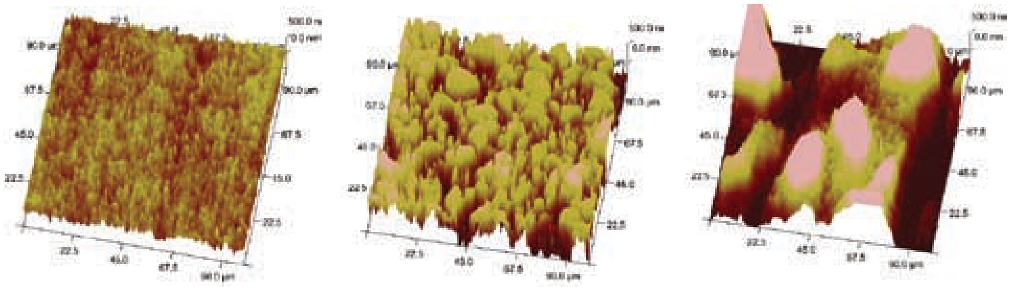

Figure 9: EsthetX® HD, Grandio®, Tetric EvoCeram® (L to R)

Nanocomposites

3M ESPE manufactures many of its fillers using a sol gel process. The sol gel process is a route wherein fillers are made from liquid precursors, or a “sol.” These liquids are chemically and mechanically processed to produce particles. One aspect of this process results in sintering, which effectively coalesces primary particles together to form larger filler particles. Sintering can be viewed as a type of melting process whereby the particles are softened, creating a surface which can attach to neighboring particles resulting in a particle-to-particle bond. The sintering process can produce fillers that are highly densified or compacted, as found in Z100™ Restorative and Filtek™ Z250 Universal Restorative. (Figure 10)

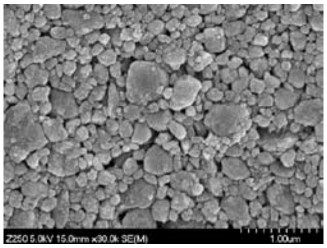

Figure 10: Filtek™ Z250 Restorative

In 2002, 3M ESPE discovered a way to modify the sintering process to produce loosely agglomerated nanoparticles, i.e., nanoclusters. Although structurally different from densified particles, these nanoclusters behaved similarly to the densified particles found in other composites in terms of providing high filler loading. This resulted in a material with the strength and wear of hybrids with significantly improved polish retention and optical properties. This technology advance was used in 3M™ ESPE™ Filtek™ Supreme Universal Restorative.

Filtek Supreme restorative was formulated using both engineered nanoparticle and nanocluster fillers. The nanocluster filler particles consist of loosely bound aggregates of engineered nanofiller particles. The addition of engineered nanoparticles to formulations containing nanoclusters reduces the interstitial spacing of the filler particles leading to higher filler loadings. The filled matrix (resin plus engineered nanoparticles) is harder and more wear resistant than resin alone. The increased filler loading results in better physical properties and wear resistance. The DEB shade fillers (Figure 11) of Filtek Supreme restorative were compositionally different than the T shade fillers. (Figure 11) The nanoclusters in the DEB shades were zirconia/silica (thereby producing a radiopaque material), while the T shades contained silica clusters (hence, no radiopacity). The ratio of nanoclusters to engineered nanoparticles was different for the DEB shades than for the T shade in Filtek Supreme restorative. Nanoclusters comprised about 90% of the filler in the DEB shades, but only 50% of the filler in the T shades. The AFM images[13] show the surfaces after they were toothbrush abraded. The Z scale is smaller in these images than in the AFM images shown previously, which effectively increases the magnification of the surface. During abrasion, the wear rate and wear pattern of the clusters is closer to the wear rate of the surrounding filled matrix, particularly in the Filtek™ Supreme XT

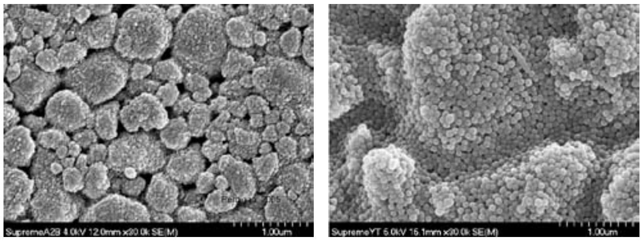

Figure 11: Filtek™ Supreme XT (DEB shades)
Filtek™ Supreme XT (T shades)
(L to R)

Universal Restorative Translucent shades. (Figure 12) This increases the polish retention of the cured composite when compared to traditional hybrid composites.

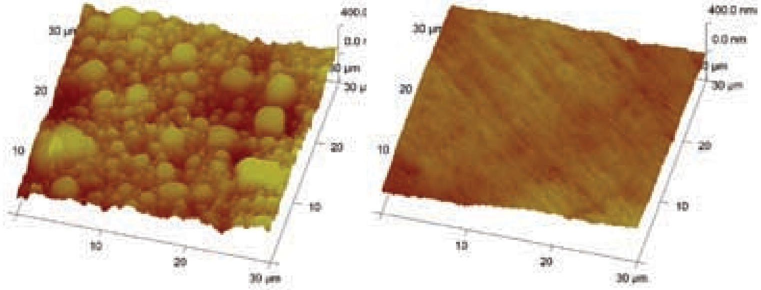

Figure 12: After Abrasion Filtek™ Supreme XT (DEB shades)

Filtek™ Supreme XT (T shades)

(L to R)

Filler Improvements in Filtek™ Z350 XT Universal Restorative

This filler technology was improved again. The manufacturing process, where the clusters are formed, was modified to produce less sintering. Once again, the nanoclusters are produced in a broad range of sizes enabling a high filler loading. As the particles are not as strongly sintered, the cluster size range could be broadened (vs. Filtek Supreme XT restorative) without affecting properties such as polish retention. These nanoclusters still have the structural integrity to provide strength, fracture and wear resistance. In the SEMs (Figure 13),[14] note the shape of the primary nanoparticles are still evident in the clusters. Both materials (DEB and T shades) contain zirconia/silica clusters (Figure 14), silica nanoparticles and zirconia nanoparticles. The ratio of nanoclusters to nanoparticles is similar in both formulations. Compositionally both clusters are the same. In order to achieve the high degree of transparency and opalescence required for the T shades, the manufacturing process is slightly different. Both the DEB shades and the T shades are radiopaque. During abrasion, their wear rate and pattern are more similar to the nanofilled matrix surrounding the clusters than Filtek Supreme XT restorative DEB and T shades. Note that in the AFM 3-D image (Figure15), the Z-scale is different than previous AFM images, resulting in a greater magnification of these surfaces. This greater magnification exacerbates the very slight irregularities in the surfaces.

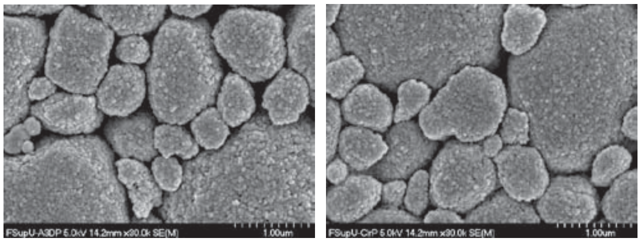

Figure 13: Nanoclusters at 30,000x Filtek™ Z350 XT (DEB shades)

Filtek™ Z350 XT (T shades)

(L to R)

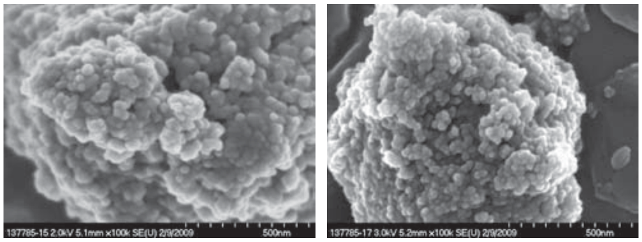

Figure 14: Nanoclusters at 100,000x Filtek™ Z350 XT (DEB shades)

Filtek™ Z350 XT (T shades)

(L to R)

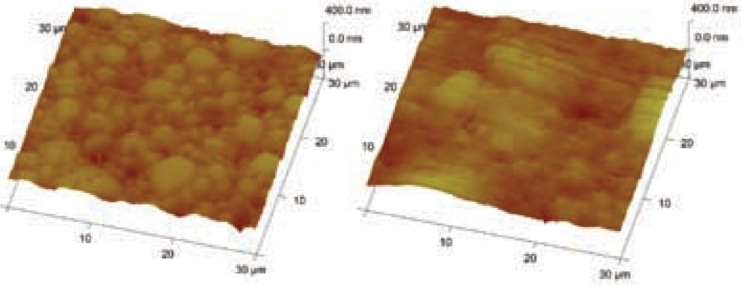

Figure 15: After Abrasion Filtek™ Z350 XT (DEB shades)

Filtek™ Z350 XT (T shades)

(L to R)

Resin System

The resin system introduced with 3M™ ESPE™ Filtek™ Z250 Universal Restorative and used in Filtek™ Supreme XT Universal Restorative comprises the majority of the Filtek™ Z350 XT Universal Restorative resin system. The resin consists of three major components. The majority of TEGDMA (in the Z100™ Restorative system) was replaced with a blend of UDMA (urethane dimethacrylate) and Bis-EMA(6) (Bisphenol A polyethethylene glycol diether dimethacrylate). UDMA and Bis-EMA(6) resins are of higher molecular weight than TEGDMA and therefore have fewer double bonds per unit of weight. The high molecular weight materials also impact the measurable viscosity. However, the higher molecular weight of the resin results in less shrinkage, improved aging and a slightly softer resin. TEGDMA and PEGDMA are used in minor amounts to adjust the viscosity. PEGDMA was used to replace part of the TEGDMA component to moderate shrinkage in Filtek Z350 XT restorative.

In Vitro Handling Evaluations

During the years Filtek™ Supreme Universal Restorative has been on the market, dentists have commented favorably on the handling of the Dentin, Enamel and Body shades. As such, when dentists and opinion leaders were asked to identify features to improve in this iteration, they indicated that the handling acceptability needed to be maintained. At the same time, they indicated that the handling of the Translucent shades needed to be improved.

Handling of composites is influenced by both resin and filler. While the filler composition of Filtek Z350 XT restorative Dentin, Enamel and Body shades is similar to its predecessor, the morphology of the clusters is different. Both the filler composition and morphology were modified to produce the new Translucent shade formulation.

Hence, numerous handling evaluations were conducted throughout the development process with dentists. Blind evaluations were done with anterior and posterior restoration in heated typodonts. Dentists were asked a series of questions about the handling of each paste. There were over 500 evaluations of this type conducted. Filtek Supreme XT restorative was included as a blind control in these evaluations. Overall, the handling of Filtek Z350 XT restorative met or exceeded the acceptability of the handling of Filtek Supreme XT restorative DEB shades. The handling acceptability of the T shades of Filtek Z350 XT restorative exceeded that of Filtek Supreme XT restorative T shades.

In one in vitro study, dentists were asked to evaluate a series of pastes that included different lots of Filtek Z350 XT restorative representing potential handling targets and two lots of Filtek Supreme XT restorative. (Figure 16) About one-third of the evaluators were current Filtek Supreme XT restorative users. The materials were evaluated in random order, restoring a Class II and Class IV preparation on heated typodonts. The handling acceptance of the DEB shade pastes is shown in Figure 16. Greater than 70% of the dentists evaluating these materials liked the handling, and consequently identified the handling specification range. In addition to the overall acceptability, the following handling attributes were also rated: viscosity, stickiness to instrument, flow, ability to hold shape or resist slump, ease of veneering (for anterior restorations), cavity and marginal adaptation, and packability (for posterior restorations only). In all cases, the Filtek Z350 XT restorative materials were rated as the same or better than the Filtek Supreme XT restorative materials.

An evaluation comparing the two Translucent shade formulations yielded even better results. A similar method was followed but with three Filtek Z350 XT restorative Translucent shade lots representing different potential handling targets and one Filtek Supreme XT restorative Translucent shade lot. Nominally, twice as many dentists liked the handling of Filtek Z350 XT restorative T shade lots as Filtek Supreme XT restorative T shades. (Figure 16) In addition, significant improvements were also noted in the individual handling attributes: viscosity, stickiness to instrument, flow, ability to hold shape or resist slump, and ease of veneering.

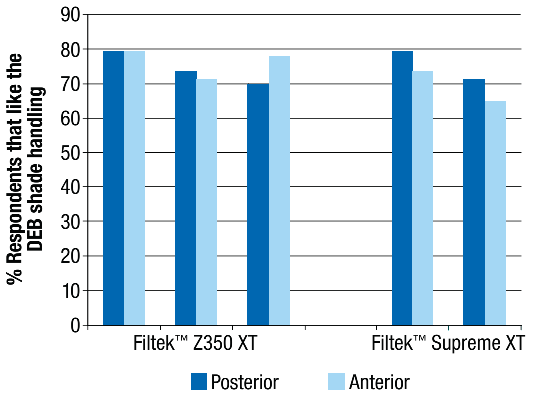

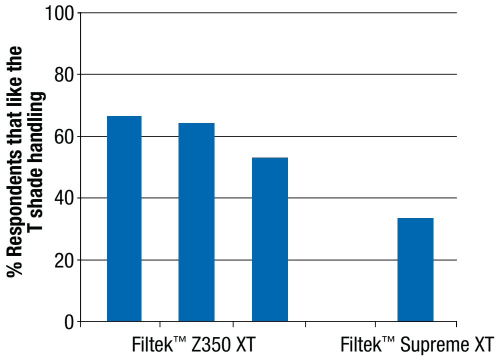

Figure 16: Handling Acceptability

Source: 3M ESPE internal data

Physical Properties

Polish Retention

Toothbrush Abrasion

Composite materials were shaped into tiles and thoroughly cured. The surfaces were polished wet using a Beuhler variable-speed grinder-polisher to remove the air-inhibited layer and to ensure a uniform surface. They were stored in water at 37ºC for 24 hours. Gloss was measured. The samples were brushed with toothpaste and a toothbrush that was mounted on an Automatic Toothbrush Machine. Gloss measurements were taken after 500 cycles and then every 1000 cycles. The test was terminated after 6000 toothbrush strokes.

Figure 17: Polish Retention Versus Microfills

Source: 3M ESPE internal data

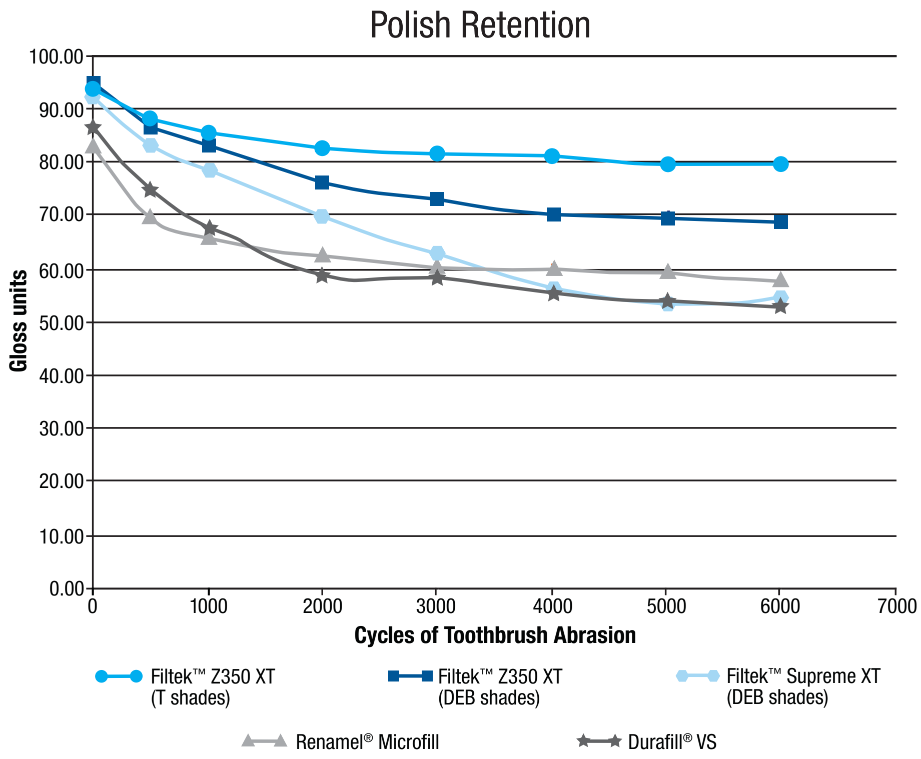

- In this test, even after only 500 cycles of toothbrush abrasion, the polish retention (gloss) of Filtek™ Z350 XT Universal Restorative DEB shades and T shades was statistically significantly higher than the microfill products Durafill VS and Renamel Microfill and the hybrid composites CeramX, Estelite Sigma Quick, EsthetX HD, Gradia Direct X, Grandio, Herculite XRV Ultra, Premise, Tetric EvoCeram, TPH3 and Venus.

- Both compositions of Filtek Z350 XT restorative were better in gloss than Filtek™ Supreme XT Universal Restorative after 2000 cycles of toothbrush abrasion.

- After 6000 cycles, the gloss of Filtek Z350 XT restorative T shades was better than Durafill VS, Renamel Microfill, CeramX, Estelite Sigma Quick, EsthetX HD, Gradia Direct X, Grandio, Herculite XRV Ultra, Premise, Tetric EvoCeram, TPH3 and Venus.

- After 6000 cycles, the gloss of Filtek Z350 XT restorative DEB shades was statistically better than Durafill VS, Renamel Microfill, CeramX, EsthetX HD, Gradia Direct X, Grandio, Herculite XRV Ultra, Premise, Tetric EvoCeram, TPH3 and Venus.

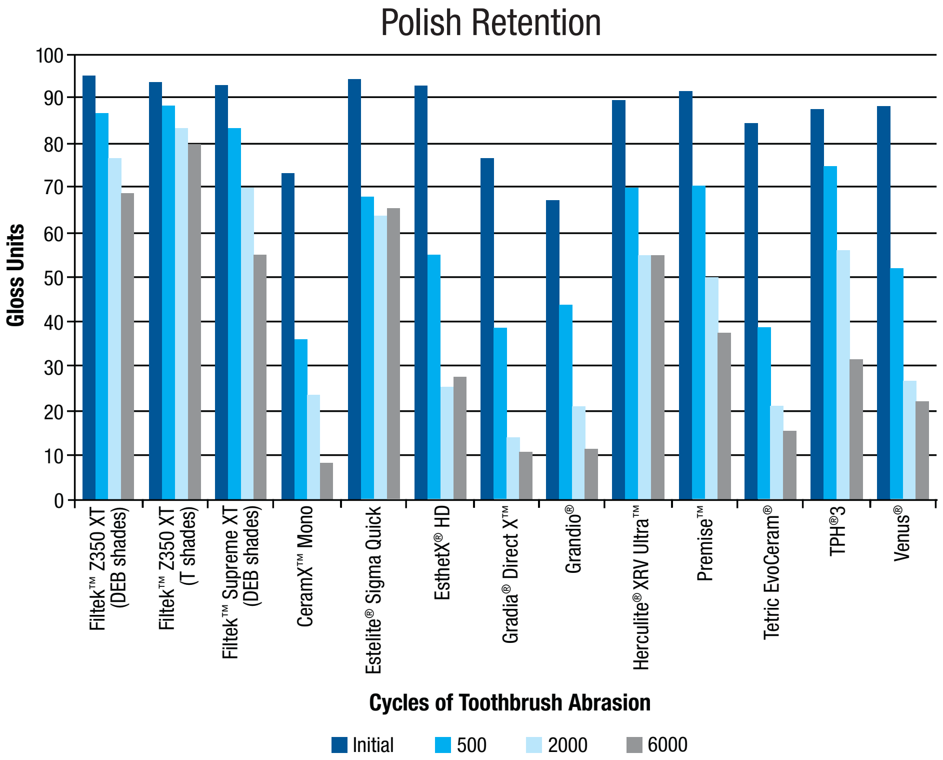

Figure 18: Polish Retention Versus Other Universal Restoratives

Source: 3M ESPE internal data

Wyko Images

These images were generated using a Wyko Optical profiler. This method provides a wider field of view than the previous AFM images. The bar to the right of the scanned image identifies the color coding key of the images.

The surface roughness is shown by the color differences. Blue indicates pits, and red indicates protrusions from the plane of the sample. Shades of green indicate roughness with a smaller peak-to-valley range.

Polished:
Filtek™ Z350 XT
Universal Restorative

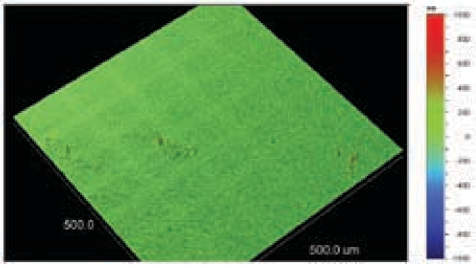

DEB shades

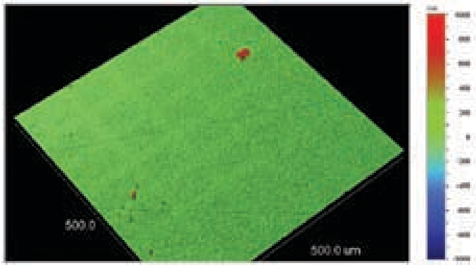

T shades

Polished:
Filtek™ Supreme XT
Universal Restorative

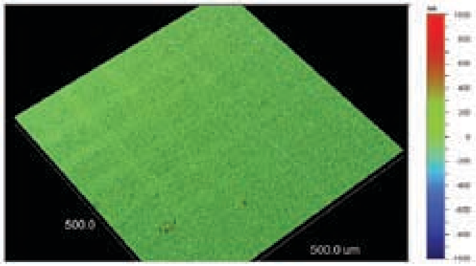

DEB shades

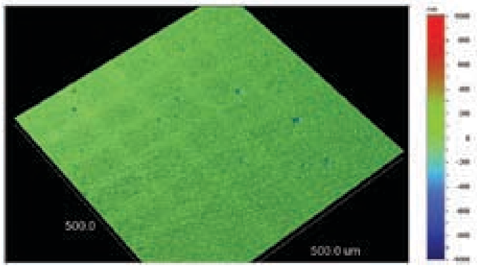

T shades

Note the smoothness of the surfaces after polish. In all of the images above, there is little color variation indicating little roughness. (Ra[15]<20 nm)

Toothbrush abraded: Filtek™ Z350 XT Universal Restorative

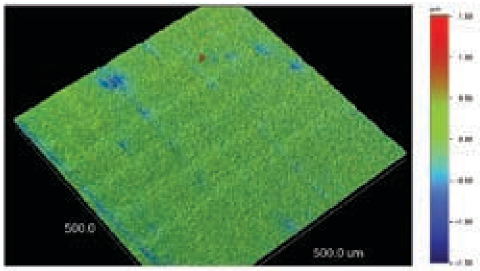

DEB shades

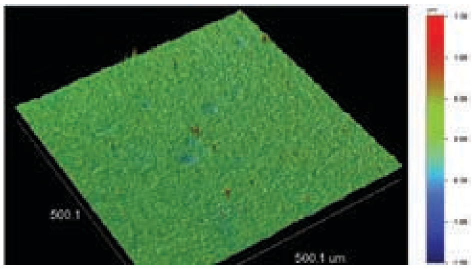

T shades

After 6000 cycles of toothbrush abrasion Wyko images were taken and the scale was changed to a slightly smaller magnification. The surface of the Filtek™ Z350 XT Universal Restorative DEB (Ra~129 nm) and T shade (Ra~70 nm) samples exhibits very minor color variation across the samples.

The images below show the original Filtek™ Supreme XT Universal Restorative DEB and T shades (Ra~148 nm) after toothbrush abrasion. Note the few deeper blue areas where larger masses were removed, probably clusters during toothbrush abrasion.

Toothbrush abraded: Filtek™ Supreme XT Universal Restorative

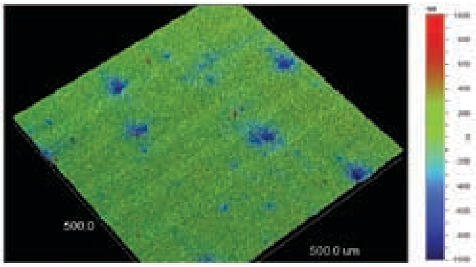

DEB shades

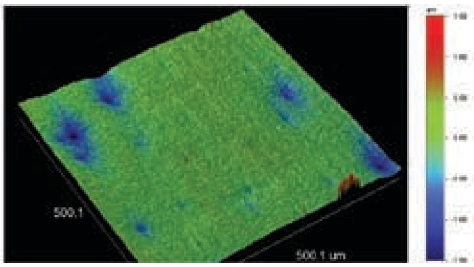

T shades

Below are images generated of two microfill restoratives after toothbrush abrasion. Note in both samples the higher areas due to the prepolymerized filler particles abrading at a different rate than the surrounding resin matrix. (Ra~135 nm) Note the deeper blue areas where larger masses were removed, which is similar to Filtek™ Supreme XT Universal Restorative materials.

Toothbrush abraded: Microfills

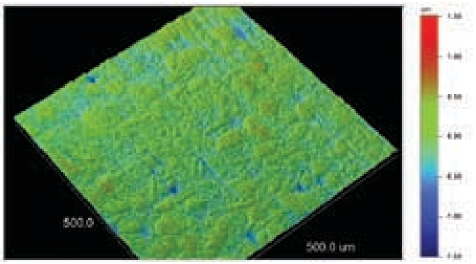

Durafill® VS

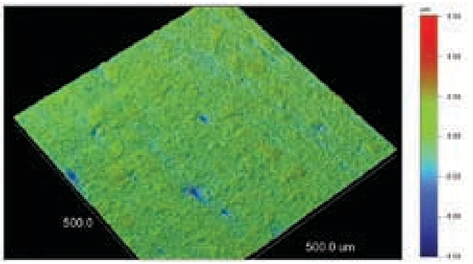

Renamel® Microfill

In the CeramX image above there is a significant roughness indicated due to the ragged appearing edges of the image and the yellow-orange hue of the surface. (Ra~240 nm) The EsthetX HD image exhibits a less uniform orange surface hue. (Ra~187 nm) This indicates broader and larger concentrations of raised areas. Both have measurably greater surface roughness than Filtek™ Z350 XT Universal Restorative materials.

Toothbrush abraded: Universals

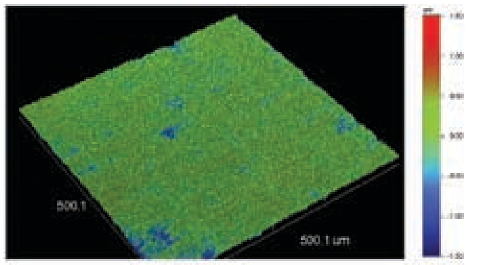

CeramX™ Mono

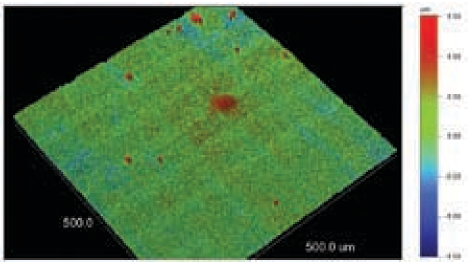

EsthetX® HD

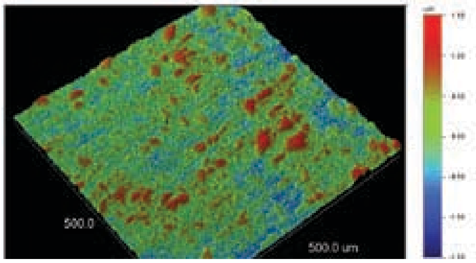

Gradia® Direct X™

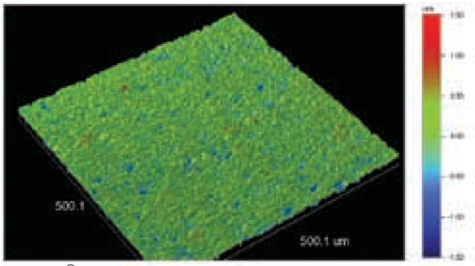

Grandio®

Gradia Direct X exhibits a very rough surface after toothbrush abrasions. (Ra~287 nm) The Wyko image shows orange protrusions and deep blue valleys in the surface. The Grandio surface shows a smaller peak-to-valley ratio (Ra~226 nm) than Gradia Direct X, but is still very rough (the color across the sample is not uniform).

Toothbrush abraded: Universals

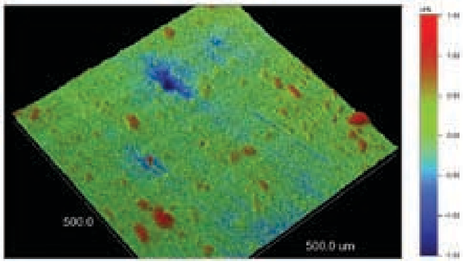

Herculite® XRV Ultra™

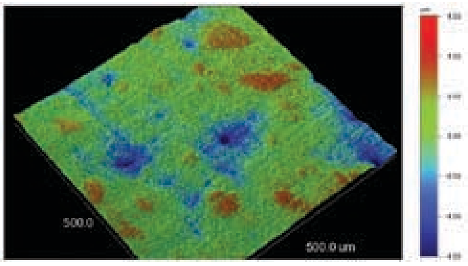

Premise™

In both of the materials shown above there are a significant number of bumps on the surface (peaks), which may be from filler particles protruding as the surrounding resin matrix was worn away. (Herculite XRV Ultra Ra~280 nm, Premise Ra~266 nm) In addition, Premise has several large valleys where a mass material was lost.

The prepolymerized filler particles (orange protrusions)in the Tetric EvoCeram become readily apparent in this surface analytic technique. (Ra~542 nm) The color variation across the Tetric EvoCeram sample covers the entire range of this surface roughness analytic technique (+ 1.5um). TPH3, on the other hand, exhibits deep valleys (large particle loss) but more diffuse peak areas (orange hues). (Ra~348 nm)

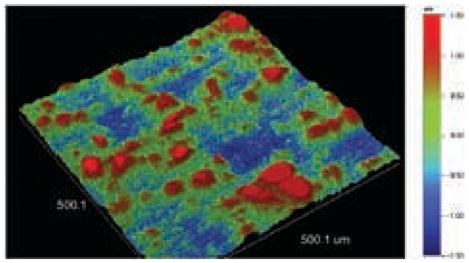

Tetric EvoCeram®

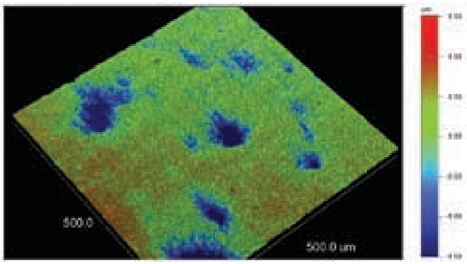

TPH®3

The abrasion pattern of the Venus sample is similar to that of CeramX. There is an obvious orange cast to the sample, indicating many peaks rising from the surface. (Ra~147 nm)
In addition, there are several deep blue striations that were created from the toothbrush abrasion test.

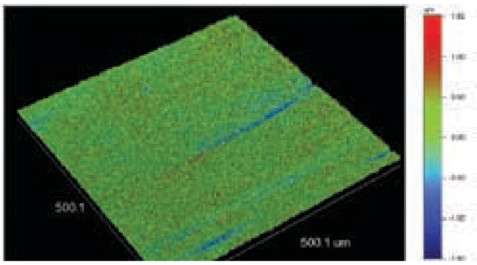

Venus®

3-Body Wear

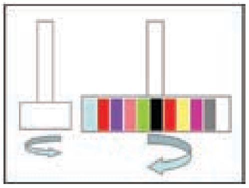

The wear rate was determined by an in vitro 3-body wear test. In this test, composite (1st body) is loaded onto a wheel, which contacts another wheel, which acts as an "antagonistic cusp" (2nd body). The two wheels counter-rotate against one another, dragging abrasive slurry (3rd body) between them. Dimensional loss during 156,000 cycles is determined by profilometry at regular intervals (i.e., after every 39,000 cycles). As the wear in this method typically follows a linear pattern, the data is plotted using linear regression. The wear rates, i.e., the slope of the lines, are determined. The comparison of rates reduces some of the variability in the test due to sample preparation and can be predictive of anticipated wear beyond the length of the actual test.

Wear Rate

Source: 3M ESPE internal data

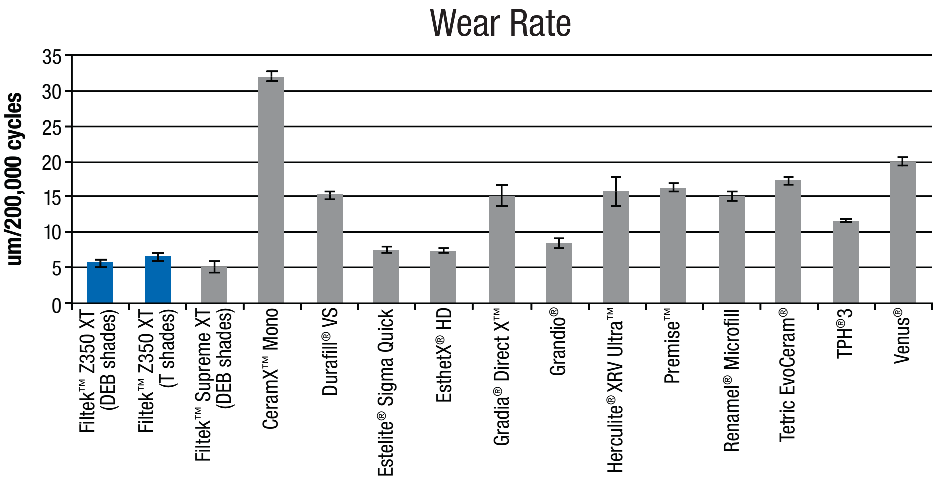

The lower the wear rate, the better the wear resistance. The wear rate of Filtek™ Z350 XT Universal Restorative DEB shades and T shades is comparable to the 3-body wear of Filtek™ Supreme XT Universal Restorative. The 3-body wear rate is statistically significantly lower (more wear resistant) than the microfills Durafill VS and Renamel Microfill. In addition, it is statistically lower than CeramX Mono, Estelite Sigma Quick, EsthetX HD, Gradia Direct X, Grandio, Herculite XRV Ultra, Premise, Tetric EvoCeram, TPH3 and Venus universal restoratives.

Fracture Toughness

The values reported for fracture toughness (K1c) are related to the energy required to propagate a crack. In this test, a short rod of material is cured. A notch is cut into the cylinder. The cylinder is placed on a fixture that supports either end and the stylus is positioned above the notch. This is similar to 3-point bend (similar to the fixture that provides flexural strength and modulus data).

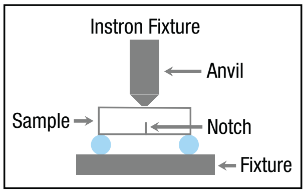

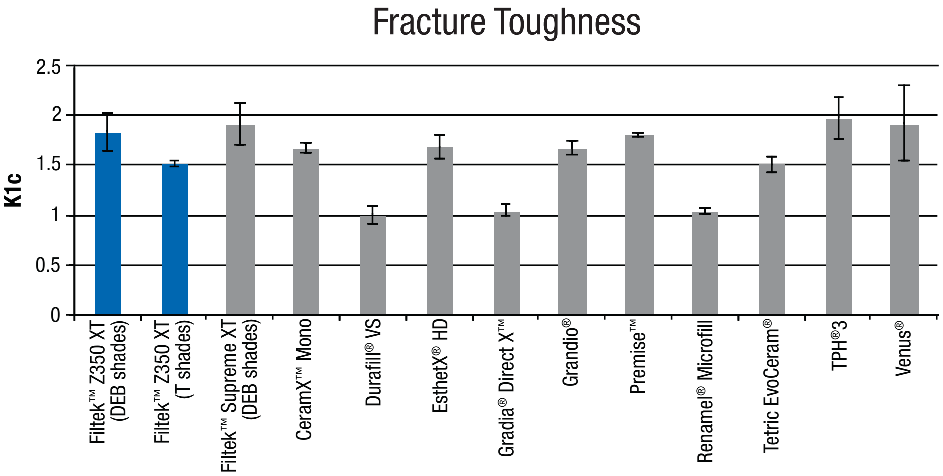

Fracture Toughness

Source: 3M ESPE internal data

The fracture toughness of Filtek Z350 XT restorative (DEB shades) is comparable to Filtek Supreme XT restorative. Filtek Z350 XT restorative has statistically significantly higher fracture toughness than the microfills Durafill VS and Renamel Microfill. The fracture toughness of Filtek Z350 XT restorative is also statistically significantly higher than Gradia Direct X and Tetric EvoCeram.

Compressive and Diametral Tensile Strength

Compressive strength is particularly important because of chewing forces. Rods are made of the material and simultaneous forces are applied to the opposite ends of the sample length. The sample failure is a result of shear and tensile forces.

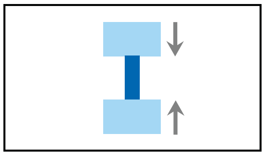

The compressive strength of Filtek™ Z350 XT Universal Restorative (DEB shades) is comparable to the T shades and Filtek™ Supreme XT Universal Restorative. Filtek Z350 XT restorative has statistically significantly higher compressive strength than Gradia Direct X.

Compressive Strength

Source: 3M ESPE internal data

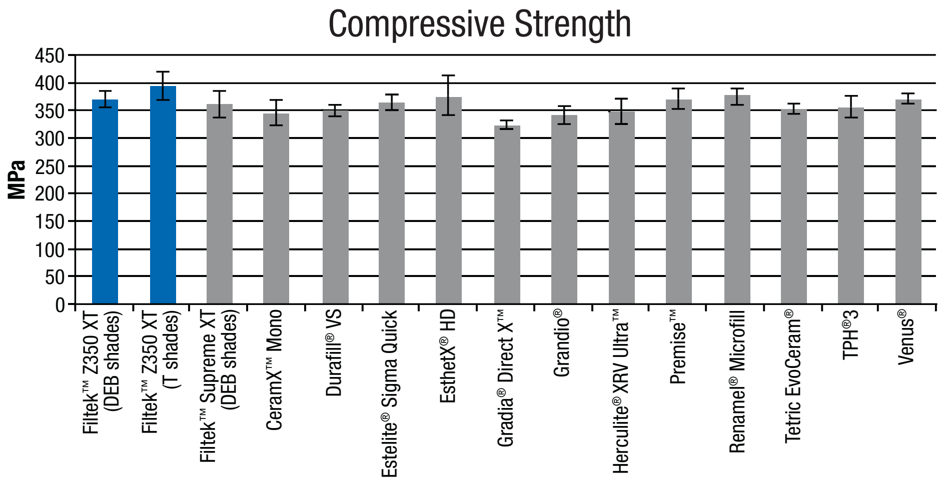

Diametral tensile strength is measured using a similar apparatus. Compressive forces are applied to the sides of the sample, not the ends, until fracture occurs.

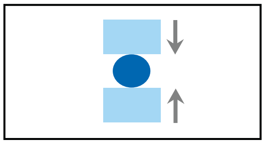

The diametral tensile strength of Filtek Z350 XT restorative is comparable to Filtek Supreme XT restorative. The diametral tensile strength is statistically significantly higher than the microfills Durafill VS and Renamel Microfill. In addition, it is statistically higher than CeramX Mono, Estelite Sigma Quick, EsthetX HD, Gradia Direct X, Premise, Tetric EvoCeram, TPH3 and Venus universal restoratives.

Diametral Tensile Strength

Source: 3M ESPE internal data

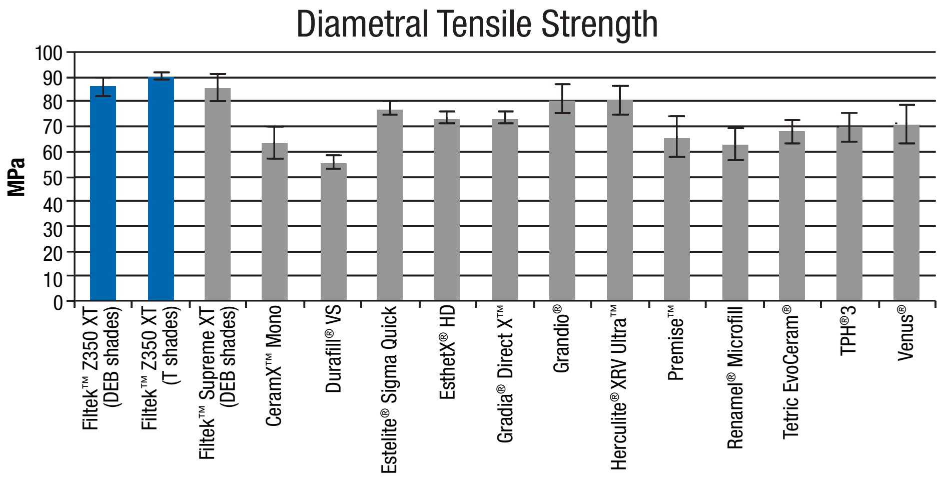

Flexural Strength and Modulus

Flexural strength is determined in the same test as flexural modulus. Flexural strength is the value obtained when the sample breaks. This test combines the forces found in compression and tension.

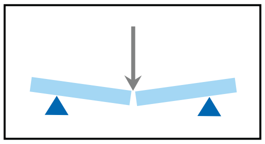

The flexural strength of the DEB shades of Filtek Z350 XT restorative is comparable to the T shades and Filtek Supreme XT restorative. The flexural strength of Filtek Z350 XT restorative is significantly higher than the microfills Durafill VS and Renamel Microfill. It is also higher than the universal restoratives CeramX Mono, Estelite Sigma Quick, EsthetX HD, Gradia Direct X, Grandio, Herculite XRV Ultra, Premise, Tetric EvoCeram and Venus.

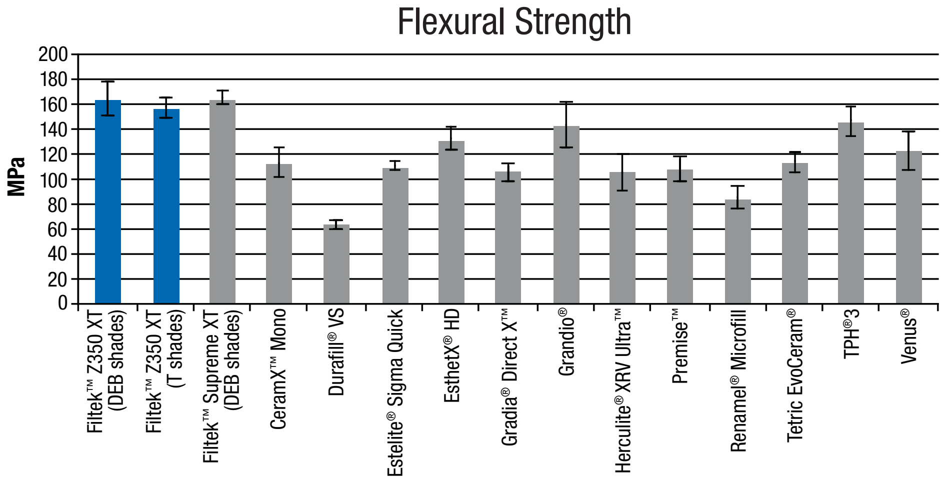

Flexural Strength

Source: 3M ESPE internal data

Flexural modulus is a method of defining a material's stiffness. A low modulus indicates a flexible material. The flexural modulus is measured by applying a load to a material specimen that is supported at each end.

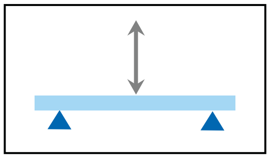

The flexural modulus of the DEB shades of Filtek Z350 XT restorative is statistically different than the T shades, CeramX Mono, Durafill VS, Estelite Sigma Quick, EsthetX HD, Gradia Direct X, Grandio, Herculite XRV Ultra, Premise, Renamel Microfill, Tetric EvoCeram, TPH3 and Venus restoratives. It is the same as Filtek Supreme XT restorative.

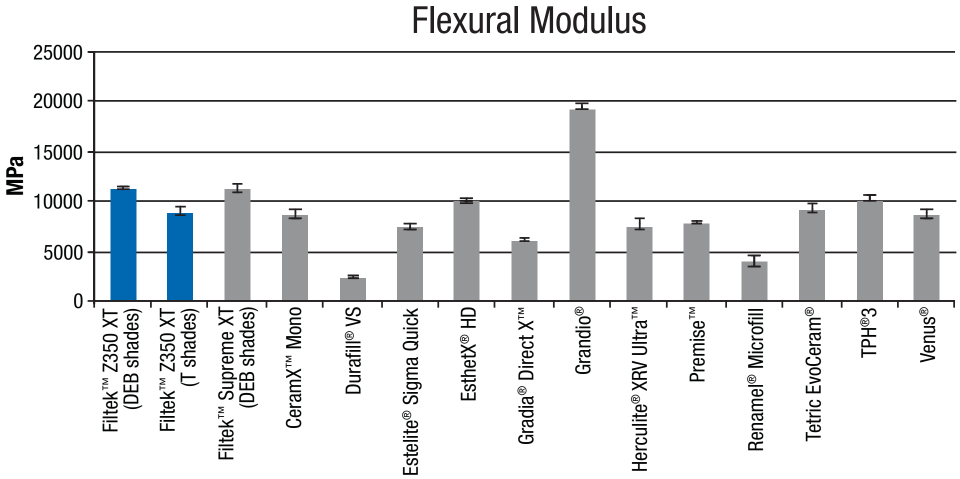

Flexural Modulas

Source: 3M ESPE internal data

Volumetric Shrinkage

A method for determining polymerization shrinkage was described by Watts and Cash (Meas. Sci. Technol. 2(1991) 788-794). In this method, a disc-shaped test specimen and uncured paste is sandwiched between two glass plates and light cured through the lower rigid plate. The flexible upper plate is deflected during the polymerization of the test specimen. The less the flexible plate bends, the lower the shrinkage. Deflection is measured and recorded as a function of time. Although this process actually measures linear shrinkage, volumetric shrinkage was closely approximated due to the fact that the dimensional changes were limited to the thickness dimension. The lower the value, the less the shrinkage.

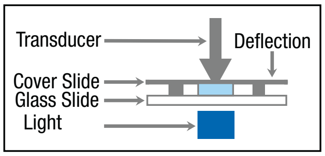

In this test, samples were exposed for 60 seconds to a 3M™ ESPE™ Visilux™ 2 Visible Light Curing Unit. The final shrinkage was recorded 4 minutes after the end of light exposure.

Shrinkage

Source: 3M ESPE internal data

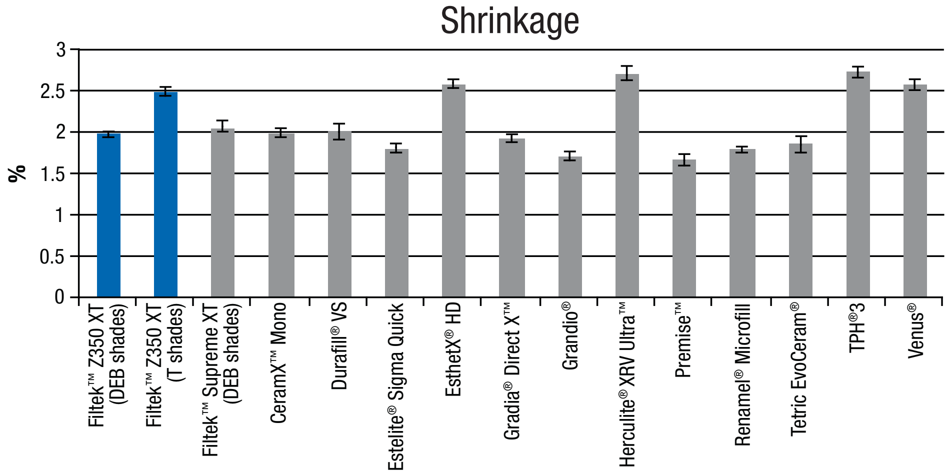

The volumetric shrinkage of Filtek™ Z350 XT Universal Restorative DEB shades and T shades is statistically different. The DEB shades of Filtek Z350 XT restorative are statistically lower than EsthetX HD, Herculite XRV Ultra, TPH3 and Venus.

Field Evaluation

A field evaluation was conducted with 256 dentists in three countries (U.S., Germany and Italy) to confirm the in vitro handling and assess the esthetic clinical performance. Recruited dentists were sent either capsules or syringes, depending on their delivery preference. In addition, they were screened on their typical composite shade technique. Dentists that were primarily single-shade users in a restoration were sent a selection of the Body shades. Dentists that typically layer multiple shades in a single restoration received a selection of all opacities.

Eighty-three dentists currently use Filtek™ Supreme XT Universal Restorative as their most frequently used composite. An additional 74 use it in their practice. One hundred and two were assessed to be primarily single-shade users and 154 were multi-shade users (in a single restoration).

There were 25,858 placements: 12,606 in anterior restorations and 13,252 in the posterior.

Anterior Single Shade – 8,905

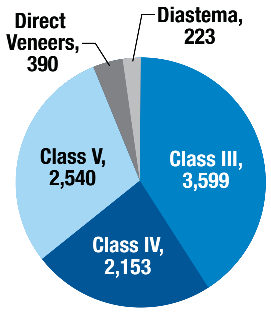

Anterior Multiple Shade – 3,701

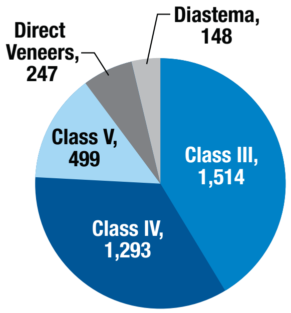

Posterior Single Shade – 11,026

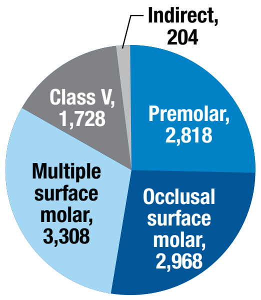

Posterior Multiple Shade – 2,226

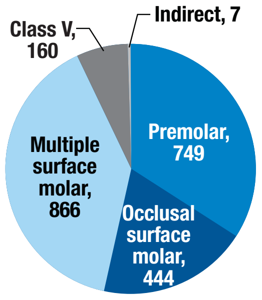

Source: 3M ESPE internal data

Handling Acceptability

The handling ratings of Filtek™ Z350 XT Universal Restorative were compared to the handling of their most frequently used composite. The data for current Filtek™ Supreme XT Universal Restorative users (labeled Filtek Supreme XT Universal Restorative) was separated from dentists using any other composite more frequently (labeled Competitive product).

Dentists were asked to rate handling attributes on a 7-point scale. For Viscosity, Stickiness, Flow and Ability to hold shape, a rating of 4 was ideal. For ease of shaping and veneering, ability to use a brush and adaptation, a rating of 7 was best.

4 is Ideal

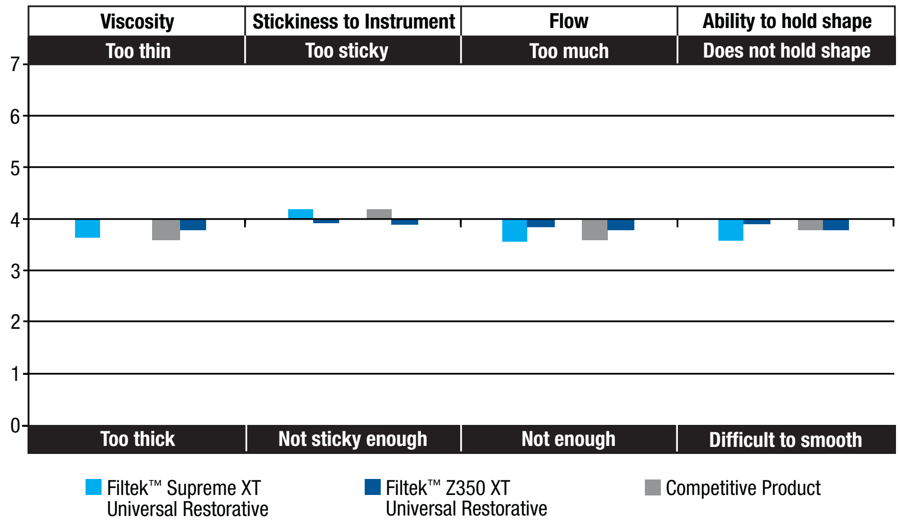

7 is Best

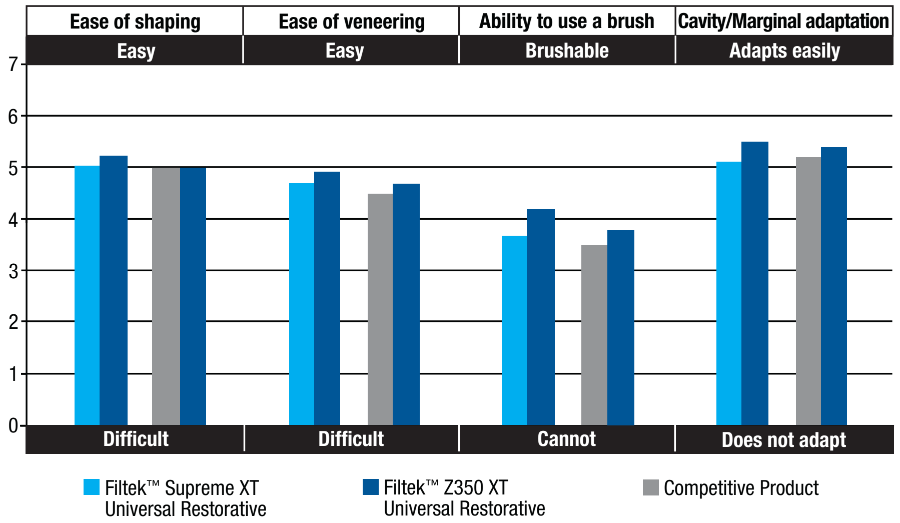

Filtek Supreme XT restorative users rated the Viscosity, Stickiness, Flow and Ability to hold shape of Filtek Z350 XT restorative as ideal. In addition, the Viscosity, Stickiness, Flow, Ability to hold shape, and the Ability to use a brush was rated statistically higher for Filtek Z350 XT restorative than their current product.

Competitive product users rated the Stickiness of Filtek Z350 XT restorative as ideal. In addition, the Stickiness, Flow and the Ability to use a brush were rated statistically higher for Filtek Z350 XT restorative than their current product.

Dentists were also specifically asked to compare Filtek Z350 XT restorative to their current product on a scale of 1 to 7. A rating of 1-3 indicated Filtek Z350 XT restorative was Much Worse to Worse than their current product. Ratings of 5-7 indicated Filtek Z350 XT restorative was Better to Much Better than their current product. A rating of 4 indicated Filtek Z350 XT restorative was performing similarly to their current product (for the purpose of this report, the rating of 4 is not shown. It can be calculated by subtracting the Worse and Better % Respondents from 100).

Supreme Users

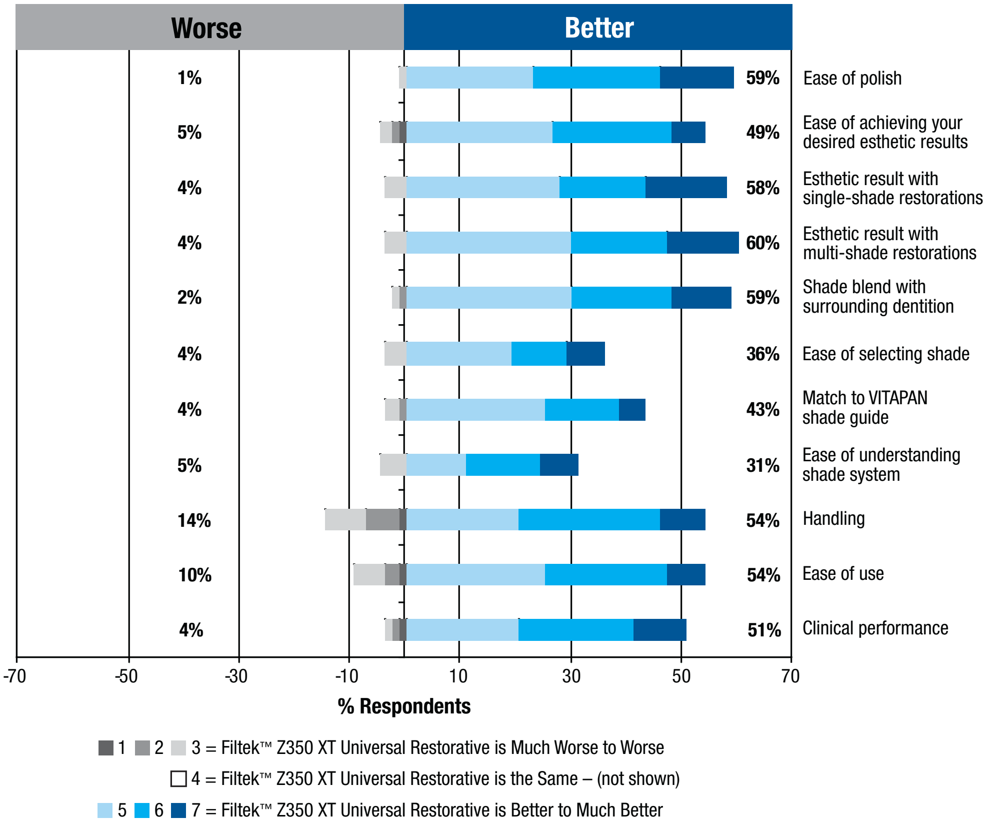

This chart shows the response of the current Filtek Supreme XT restorative users. Over 50% of the current Filtek Supreme XT restorative users felt Filtek Z350 XT restorative exhibited improved:

- Ease of Polish
- Results with single- and multi-shade
- Shade blend with surrounding dentition
- Handling
- Ease of use
- Clinical performance

In all aspects, except handling, more than 90% agreed the performance of Filtek Z350 XT restorative was the same or better than Filtek Supreme XT restorative. More than 85% rated the handling of Filtek Z350 XT restorative as the same or better than Filtek Supreme XT restorative.

Competitive Users

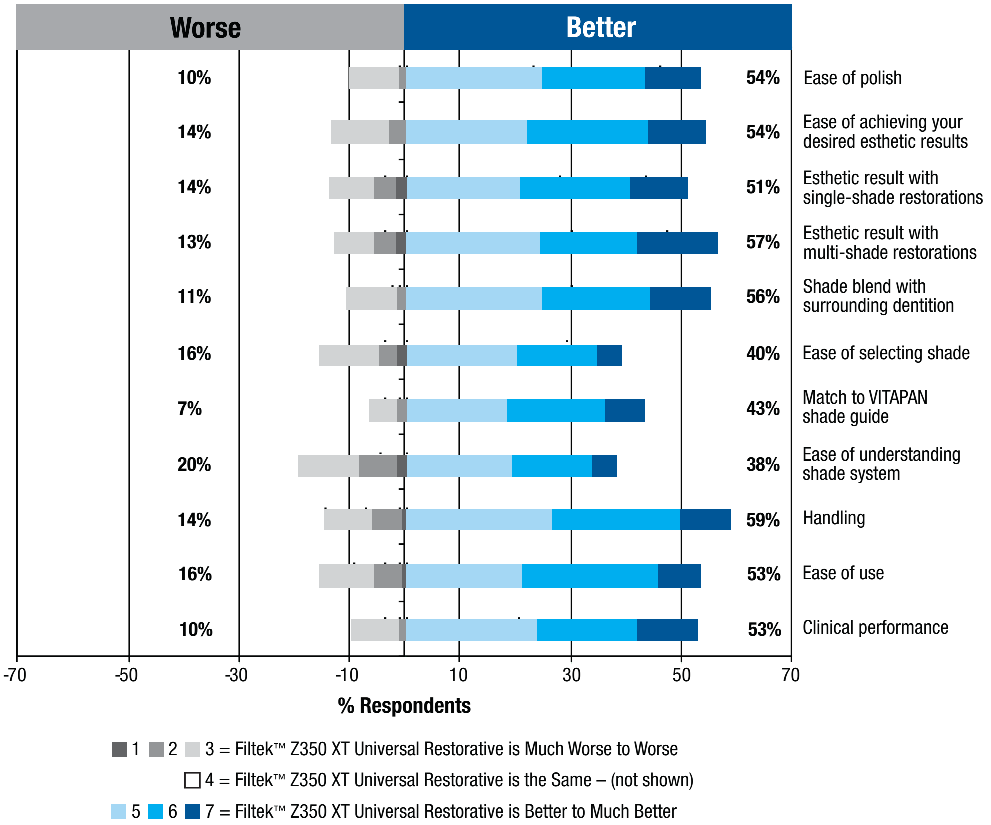

This chart shows the response of the current Competitive product users. Over 50% of the current competitive product users felt Filtek™ Z350 XT Universal Restorative exhibited improved:

- Ease of Polish
- Ease of achieving your desired esthetic result
- Results with single- and multi-shade
- Shade blend with surrounding dentition
- Handling
- Ease of use
- Clinical performance

In all aspects, except ease of selecting shade, Ease of understanding the shade system, and Ease of use, more than 85% agreed the performance of Filtek Z350 XT restorative was the same or better than their current product. Many of these dentists were not familiar with Filtek™ Supreme XT Universal Restorative. Dentists in this study did not receive any Shade Reference Card or Shade Wheel to assist in shade identification. Even with this handicap, these three attributes were rated the same or better than competitive products by 80% of the dentists.

Questions and Answers

- **This system is too complicated for me. Most of the time I use only one shade in my restorations.**

  The Filtek™ Z350 XT Universal Restorative system is designed to be flexible to meet the needs of every dentist. While there are four opacities available, dentists using only one shade in a restoration can use the Body shades. Use of all opacities in this system is not required but optional.

- **The Translucent shades are very transparent. Tooth structure is not. Where can I use this type of material?**

  The Translucent shades can be used either internally or externally in a restoration. These materials can be used to accentuate the recreated dentin mamelons and maximize translucency of an incisal edge. In addition, they can be used as a very thin layer over the surface of the restoration to take advantage of their excellent polish retention.

- **What is the difference between Filtek™ Supreme XT Universal Restorative and Filtek Z350 XT restorative?**

  There have been improvements made in the filler processing and pigments to provide better polish retention, improved fluorescence and Filtek Supreme XT-like handling for all opacities, including translucent.

- **Can I use my old shade wheel (from Filtek Supreme XT restorative) or recipes to create multi-shaded restorations?**

  Yes. The color targets for the shades remain the same. However, due to the modified shade offering, a new wheel (and recipes) was created to take advantage of this enhancement.

1. Mitra SB, Wu D, Holmes BN. JADA (2003) 134, 1382-1390.

2. Katholieke Universiteit Leuven clinical study.

3. Dentin, Enamel and Body shades will be referred to as DEB shades throughout this document.

4. Translucent shades will be referred to as T shades throughout this document.

5. Takahashi MK, Viera S, Rached RN, Almeida JB, Aguiar M, Souza EM. Operative Dentistry (2008), 33-2, 189-195.

6. Kobussen GA, Craig BD, Halvorson RH, Doruff MC, Bigham WS. Optical Properties of Highly Aesthetic Composite Restoratives, J Dent Res 88 (Spec Iss A):1508, 2009.

7. Lee YK. Measurement of Opalescence of Resin Composites. Dental Materials (2005) 21, 1068-1074.

8. Kobussen GA.

9. Dr. Jorge Perdigao, University of Minnesota, Division of Operative Dentistry, Department of Restorative Science.

10. Uncured paste was dissolved in acetone, and then centrifuged. The supernatant liquid was removed and the residue dissolved in acetone, then centrifuged.

11. AFM – Atomic Force Microscopy in a 3-D surface plot – Scanned area is about $100um^2$. The Tapping Mode AFM uses a single crystal silica probe with a force constant of ~40N/m to determine the surface profile. The darker the color – the deeper the gouge, the lighter the color – the higher the peak (the pink color indicates the maximum instrument capability).

12. Ibid

13. Ibid

14. Dr. Jorge Perdigao.

15. Ra is the arithmetic average of the absolute values of the surface height deviations from the mean surface plane calculated from the $500x500\mu^2$ Wyko maps.

	Level	Filtek™ Z350 XT Universal Restorative (DEB shades)	Filtek™ Z350 XT Universal Restorative (T shades)	Filtek™ Supreme XT Universal Restorative (DEB shades)	CeramX™ Mono	Durafill® VS	Estelite® Sigma Quick	EsthetX® HD	Gradia® Direct	Grandio®	Herculite® XRV Ultra™	Premise™
**Compressive Strength**	MPa	370.56	394.01	361.37	346.80	349.86	364.19	376.83	323.40	341.84	349.10	370.81
	StDev	15.13	25.05	23.78	22.96	10.40	14.03	35.41	7.92	16.04	23.51	18.83
**Diametral Tensile Strength**	MPa	86.12	90.64	85.53	63.31	55.89	77.56	73.64	52.82	81.28	80.65	65.89
	StDev	3.91	1.40	5.47	6.49	2.87	2.98	2.38	5.89	5.63	5.76	8.18
**Flexural Strength**	MPa	165.14	157.98	165.90	113.68	64.50	111.08	132.90	106.07	144.03	106.48	108.64
	StDev	13.59	8.16	5.40	11.52	3.62	3.94	8.65	6.77	17.54	14.34	9.64
**Flexural Modulus**	MPa	11348.00	9180.00	11436.00	8830.00	2613.00	7552.00	10128.00	6299.00	19437.00	7679.00	7839.00
	StDev	271.00	431.00	442.00	379.00	66.00	202.00	146.00	185.00	299.00	541.00	183.00
**Fracture Toughness**	K1c	1.84	1.51	1.92	1.69	1.01	—	1.70	1.05	1.68	—	1.81
	StDev	0.19	0.03	0.21	0.05	0.09	—	0.12	0.06	0.07	—	0.03
**Shrinkage**	%	1.97	2.48	2.06	1.97	2.00	1.80	2.58	1.92	1.69	2.70	1.66
	StDev	0.03	0.06	0.06	0.05	0.08	0.05	0.05	0.04	0.04	0.07	0.06
**Polish Retention**												
**Initial**	Mean	94.83	93.83	92.81	72.90	86.33	93.93	92.45	76.17	67.27	89.67	91.60
	StDev	1.03	1.39	2.35	—	0.15	0.68	2.33	0.32	1.71	2.17	0.96
**500 cycles**	Mean	86.82	88.04	83.09	36.03	74.82	67.62	54.75	37.98	43.47	69.63	70.36
	StDev	5.77	6.01	6.08	7.27	4.85	7.45	3.86	10.27	4.82	9.21	5.97
**1000 cycles**	Mean	83.32	85.72	78.73	25.50	68.08	64.14	27.65	21.58	35.31	60.83	63.11
	StDev	5.96	5.60	7.69	6.39	5.67	3.75	1.03	12.86	6.34	7.29	5.81
**2000 cycles**	Mean	76.55	82.83	69.74	23.18	59.03	63.55	25.05	13.53	20.79	54.89	49.35
	StDev	6.43	5.12	8.57	2.74	6.15	3.88	2.64	5.00	3.29	6.85	8.48
**3000 cycles**	Mean	73.19	82.01	62.89	10.45	58.70	64.29	29.28	13.00	17.26	52.57	44.12
	StDev	5.99	5.96	8.69	1.37	3.38	9.89	2.59	0.81	2.81	11.34	4.93
**4000 cycles**	Mean	70.33	81.23	56.63	9.80	55.67	62.35	26.78	10.47	13.13	53.71	39.29
	StDev	5.52	4.15	7.28	1.23	6.57	3.66	6.12	0.89	1.33	5.48	6.97
**5000 cycles**	Mean	69.66	79.80	53.48	9.55	54.02	63.30	28.68	11.77	12.16	52.84	39.26
	StDev	5.36	6.05	8.19	1.00	3.57	9.53	0.65	1.16	0.96	11.58	3.12
**6000 cycles**	Mean	68.62	79.72	54.73	7.98	53.21	65.01	27.65	10.55	11.48	54.88	37.18
	StDev	4.77	4.42	7.75	0.71	6.32	3.33	1.01	1.22	0.98	4.57	5.00
**3-Body Wear Rate**	um lost	5.61	6.54	5.07	32.04	15.22	7.50	7.38	15.17	8.49	15.78	16.27
	StDev	0.63	0.50	0.80	0.68	0.55	0.46	0.31	1.43	0.64	2.13	0.55

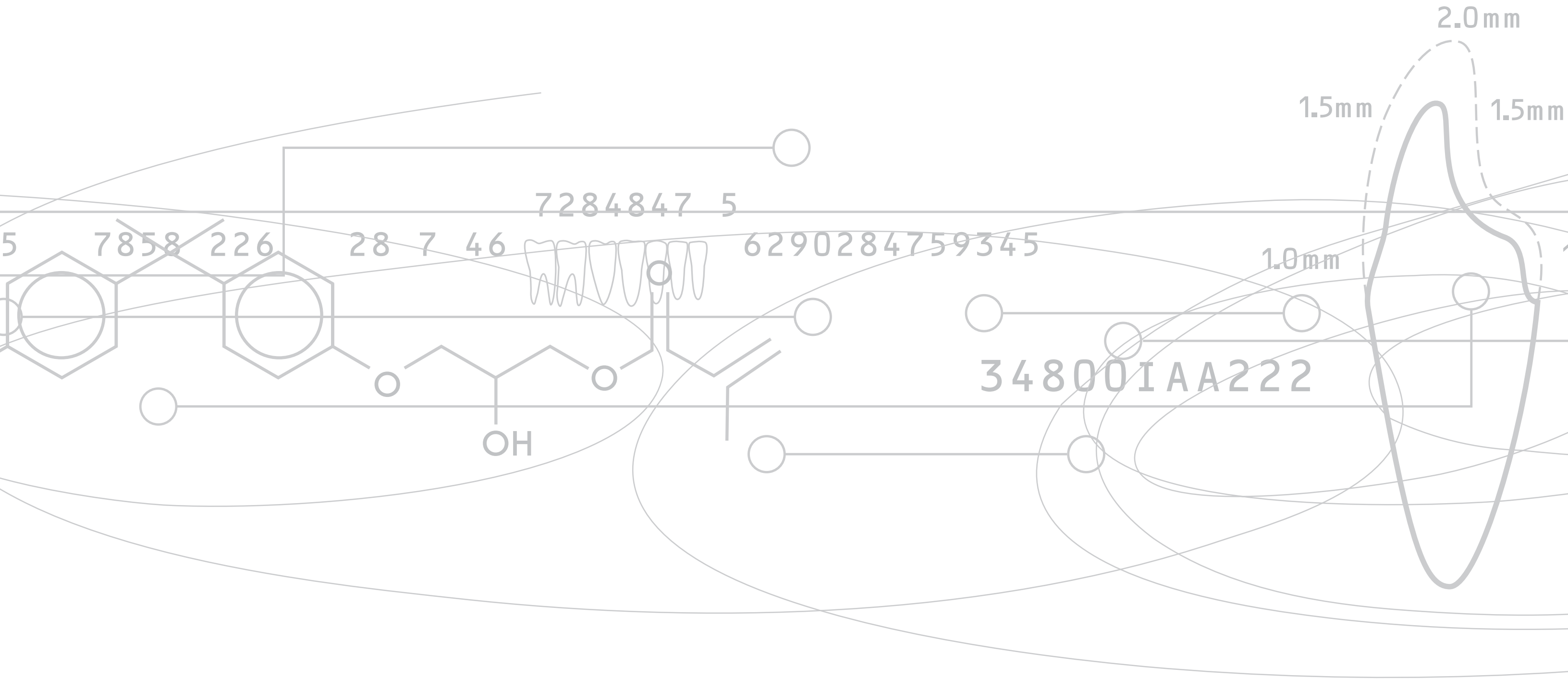

3M ESPE

Name

Vskd Askikfgajer
Fayhin siekit Hainety
Askik Siekit Hainety
Country

Name

Vskd Askikfgajer
Fayhin siekit Hainety
Askik Siekit Hainety
Country

Please recycle. Printed in (Country).
© 3M 2010. All rights reserved.

3M, ESPE, Filtek, Visilux and Z100 are trademarks of 3M or 3M ESPE AG. CeramX, Durafill, Estelite, EsthetX, Gradia Direct X, Grandio, Herculite XRV Ultra, Premise, Renamel, Tetric EvoCeram, TPH3, Venus, VITAPAN and Vit-L-Escence are not trademarks of 3M.
