# Supplementary material for: Effect of different scaling instruments on the roughness of esthetic resin composite restorations: an in vitro study
Source: Front Dent Med. 2026 Aug 28;7:1894177. doi: 10.3389/fdmed.2026.1894177 (PMC13561887; doi:10.3389/fdmed.2026.1894177)
Supplement: Supplementary file 1 [file Datasheet1.zip › Beautifil-II LS.pdf]

en

READ CAREFULLY BEFORE USE

Light-cured Dental restorative material

BEAUTIFIL II LS is a light-cured (400-500 nm), radiopaque restorative material indicated for anterior and posterior restorations including occlusal surfaces.

COMPOSITION

Glass powder, Urethane diacrylate, Bis-MPEPP, Bis-GMA, TEGDMA, *Polymerization initiator*, Pigments and others

INDICATIONS

- ➢ Anterior and posterior restorations
- ➢ Repair of restorations and prostheses

PRECAUTIONS

1. Do not use this product on patients with known allergies to this material and/or methacrylate monomer.
2. Operators with known allergies to this material *and/or* methacrylate monomer should exercise caution while using this product.
3. *If any inflammation* or other allergic reactions occur on either patient or operator, immediately discontinue use and seek medical advice.
4. Use medical (dental) gloves and protective glasses to avoid sensitization to this product. In case of accidental contact with oral soft tissue or skin, immediately blot with alcohol moistened cotton ball, and rinse with plenty of water. Medical (dental) gloves provide some protection against direct contact with this material; however some monomers can penetrate through the gloves very quickly to reach the skin. Therefore, should this *material contact* even the gloves, immediately remove them and wash hands with plenty of water.
5. In case of contact with eyes, immediately flush the eyes with plenty of water and seek medical advice.
6. Do not re-use tips.
7. This product is intended for use by dental professionals only.

DIRECTIONS FOR USE

A. Anterior and posterior restorations

A-1. Cleaning of tooth surface

Thoroughly clean the tooth surface to remove plaque.

A-2. Shade selection

Select appropriate shade using a shade guide while the tooth is still moist.

A-3. Cavity preparation

Prepare the cavity following the standard method.

A-4. Isolation

Use a rubber dam etc to isolate the treatment area from contamination.

A-5. Placement of matrix

Place a matrix band in case the cavity involves proximal area. Follow the matrix manufacturer's instructions for use.

A-6. Pulp protection

In case of pulp exposure or deep cavity with close proximity to the pulp, cap the pulp with calcium hydroxide etc.

A-7. Adhesive application

Apply dentin adhesive following the manufacturer's instructions for use. Either two step adhesive such as FL-BOND II or one step adhesive such as BeautiBond can be used

Scanned with CamScanner
