# Supplementary material for: Effect of different scaling instruments on the roughness of esthetic resin composite restorations: an in vitro study
Source: Front Dent Med. 2026 Aug 28;7:1894177. doi: 10.3389/fdmed.2026.1894177 (PMC13561887; doi:10.3389/fdmed.2026.1894177)
Supplement: Supplementary file 1 [file Datasheet1.zip › Beautifil-II.pdf]

BEAUTIFIL II

EN Instructions for use · ES Instrucciones de uso · RO Instrucțiuni de utilizare · DE Gebrauchsanleitung · IT Istruzioni per l'uso · SV Bruksanvisning · FR Mode d'emploi · RU Инструкция по применению · EL Οδηγίες χρήσης · NL Gebruiksaanwijzing · PL Instrukcja obsługi

06:2019-03
71428

CE 0123

Manufacturer
SHOFU INC.
11 Kamitakamatsu-cho, Fukuine,
Higashiyama-ku, Kyoto 605-0983, Japan
www.shofu.com

EC REP
SHOFU DENTAL GmbH
Am Brüll 17, 40878 Ratingen, Germany

SHOFU DENTAL CORPORATION
1225 Stone Drive, San Marcos,
California 92078-4059, USA

SHOFU DENTAL ASIA-PACIFIC PTE.LTD.
10 Science Park Road, #03-12,
The Alpha, Science Park II, Singapore 117684

EN READ CAREFULLY BEFORE USE

Fluoride Releasing Restorative Material

BEAUTIFIL II is a light-cured radiopaque restorative material indicated for anterior and posterior restoration.

SHADES
Syringe: A1, A2, A3, A3.5, A4, B1, B2, B3, C2, C3, A2O, A3O, Inc(Incisal), BW(Bleaching White)
Tips: A1, A2, A3, A3.5, A4, B1, B2, C2, A2O, A3O, Inc, BW

COMPOSITION
Bis-GMA, TEGDMA, S-PRG filler based on fluoroboro-aluminosilicate glass, Polymerization initiator, Pigments and others

INDICATIONS
- Anterior and posterior restorations
- Direct laminate veneer
- Repair of restorations and prostheses
- Core-build up

PRECAUTIONS
1. Do not use this product on patients with known allergies to this material and/or methacrylate monomer.
2. Operators with known allergies to this material and/or methacrylate monomer must not use this product.
3. If any inflammation or other allergic reactions occur on either the patient or operator, immediately discontinue use and seek medical advice.
4. Do not re-use tips.
5. Use medical (dental) gloves and protective glasses to avoid sensitization to this product. In case of accidental contact with oral soft tissue or skin, immediately blot with alcohol moistened cotton ball, and rinse with plenty of water. Medical (dental) gloves provide some protection against direct contact with this material; however some monomers can penetrate through the gloves very quickly to reach the skin. Therefore, should this material contact even the gloves, immediately remove them and wash hands with plenty of water.
6. In case of contact with eyes, immediately flush the eyes with plenty of water and seek medical advice.
7. This product is intended for use by dental professionals only.

DIRECTIONS FOR USE
1. Cleaning of tooth surface
Thoroughly clean the tooth surface to remove plaque.
2. Shade selection
Select appropriate shade using a shade guide while the tooth is still moist.
3. Cavity preparation
Prepare the cavity following the conventional method.
4. Isolation
Isolate with a rubber dam.
5. Pulp protection
In the case where the pulp is exposed or the affected area is close to the pulp, cap the pulp with calcium hydroxide etc.
- Do not use eugenol containing materials since they might inhibit polymerization or adhesion of the material.
6. Conditioning and application of adhesive
Apply dentin adhesive following the manufacturer's instructions for use.
7. Placement
Dispense adequate amount of BEAUTIFIL II onto a paper pad from the syringe. Apply the dispensed material into the cavity using a suitable instrument and create the shape desired.
When using a tip, insert the tip into the dispenser to apply the material directly into the cavity. The tip material may also be dispensed onto a paper pad to be applied into the cavity with a suitable instrument.
- When storing these materials in a refrigerator, allow them to reach room temperature before use.
- When applying the material directly into the cavity, sterilize the dispenser before and after use.
- Tips should be used for only one patient and then discarded at the end of the procedure to avoid cross contamination.
- Grip the dispenser gently to dispense the material slowly. Excessive pressure may cause Tips to rupture or to eject from the dispenser.
- The paste dispensed onto the paper pad should be shielded from ambient light by light-shielding cover to avoid unintended polymerization of the material.
- Tightly close the cap after each use.
8. Light-curing
Light-cure using a dental light-curing unit.
- When the cavity is deep, incremental filling and light-curing not exceeding 2 mm is recommended.
- When the cavity surface is large, light-cure in segments.
- Dental light-curing unit and light-curing time

Light-curing unit	Halogen	LED
Light-curing time	20 sec.	10 sec.

- Halogen light-curing unit
Wavelength: 400-500 nm,
Light intensity: ≥ 500 mW/cm^2
- LED light-curing unit
Wavelength: 440-490 nm,
Light intensity: ≥ 1000 mW/cm^2
- Use a dental light-curing unit having specifications equivalent to those described above. When using the one having performance other than these recommendations, follow the instructions of each unit to light-cure BEAUTIFIL II properly.
- Operators must use protective glasses or light shielding plate to avoid looking directly at the curing light. Operators must also protect patients' eyes from direct exposure to curing light.
- Disinfect the syringe with alcohol after each use to avoid cross-contamination. Then, tightly close the cap. Use caution to avoid including alcohol into the paste.
- Disinfect the light-curing unit with alcohol after each use to avoid cross-contamination.
9. Finishing and polishing
After light-curing, contour, finish and polish the restoration following the conventional method.

NOTE ON USE
1. Follow the instructions for use of each dental material or instrument to be used in conjunction with this product.
2. Do not use this product for any purposes other than specifically outlined in the INDICATIONS in these instructions for use.
3. Use this product within the expiration date indicated on the package and container.
(Example ⌛ YYYY-MM-DD → Year-Month-Date of the expiration date)

STORAGE
Store at room temperature (1-30 °C/34-86 °F). Avoid high temperatures and high humidity. Keep away from direct sunlight and any source of ignition.

CAUTION: US Federal law restricts this device to sale by or on the order of a dental professional.

DE VOR GEBRAUCH SORGFÄLTIG DURCHLESEN

Fluoridfreisetzendes Füllungsmaterial

BEAUTIFIL II ist ein lichthärtendes, röntgenopakes, für Front- und Seitenzähne indiziertes Füllungsmaterial.

FARBEN
Spritze: A1, A2, A3, A3.5, A4, B1, B2, B3, C2, C3, A2O, A3O, Inc (inzisal), BW (gebleicht weiß)
Tips: A1, A2, A3, A3.5, A4, B1, B2, C2, A2O, A3O, Inc, BW

ZUSAMMENSETZUNG
Bis-GMA, TEGDMA, S-PRG Füller auf der Basis von Fluoroboroaluminiumsilikatglas, Polymerisationsinitiator, Pigmente u. a.

INDIKATIONEN
- Front- und Seitenzahnfüllungen
- Direkte Verblendschalen
- Reparatur von Restaurationen und Prothesen
- Stumpfaufbau

VORSICHTSMASSNAHMEN
1. Das Produkt nicht bei Patienten mit bekannten Allergien gegen dieses Material und/oder Methacrylat-Monomere verwenden.
2. Behandler mit bekannten Allergien gegen dieses Material und/oder Methacrylat-Monomere dürfen das Produkt nicht benutzen.
3. Falls bei Patient oder Behandler Entzündungen oder andere allergische Reaktionen auftreten, sofort den Gebrauch einstellen und ärztlichen Rat einholen.
4. Tips nicht wieder verwenden.
5. Zur Vermeidung einer Sensibilisierung gegen das Produkt (zahn-)medizinische Schutzhandschuhe und Schutzbrille tragen. Bei versehentlichem Kontakt mit oralen Weichgeweben oder Haut sofort mit einem mit Alkohol befeuchteten Wattebausch abtupfen und mit reichlich Wasser spülen. (Zahn-)medizinische Handschuhe bieten zwar einen gewissen Schutz vor direktem Kontakt mit diesem Material, doch einige Monomere können sehr schnell durch die Handschuhe auf die Haut dringen. Sollte das Material also auf die Schutzhandschuhe gelangen, diese sofort ausziehen und die Hände mit reichlich Wasser waschen.
6. Bei Kontakt mit den Augen diese sofort mit reichlich Wasser spülen und ärztlichen Rat einholen.
7. Dieses Produkt ist nur für den zahnärztlichen Gebrauch vorgesehen.

GEBRAUCHSANLEITUNG
1. Reinigung der Zahnoberfläche
Die Zahnoberfläche gründlich von Plaque reinigen.
2. Farbauswahl
Mit einem Farbschlüssel die geeignete Farbe auswählen, solange der Zahn noch feucht ist.
3. Kavitätenpräparation
Die Kavität wie gewohnt präparieren.
4. Isolierung
Mit Kofferdam isolieren.
5. Pulpaschutz
Bei freiliegender Pulpa oder pulpanahen Kavitäten die Pulpa mit Calciumhydroxid usw. überkappen.
- Keine eugenolhaltigen Materialien verwenden, da diese die Polymerisation oder Adhäsion des Produkts beeinträchtigen können.
6. Konditionierung und Adhäsivauftragung
Ein Dentinadhäsiv nach der Anleitung des Herstellers applizieren.
7. Ausbringen
Eine ausreichende Menge BEAUTIFIL II aus der Spritze auf einen Mischblock geben. Das entnommene Material mit einem geeigneten Instrument in die Kavität applizieren und wie gewünscht modellieren.
Bei Verwendung eines Tips diesen in den Dispenser einsetzen und das Material direkt in die Kavität applizieren. Das Tip-Material kann aber auch auf einen Mischblock gegeben und mit einem geeigneten Instrument in die Kavität appliziert werden.
- Im Kühlschrank gelagertes Material erst verarbei-ten, wenn es sich auf Zimmertemperatur erwärmt hat.
- Bei der direkten Applikation des Materials in die Kavität den Dispenser vor und nach dem Gebrauch sterilisieren.
- Tips sollten nur bei einem Patienten verwendet und am Ende der Behandlung entsorgt werden, um Kreuzkontaminationen zu vermeiden.
- Den Dispenser vorsichtig greifen, um das Material langsam aufzutragen. Bei übermäßigem Druck können die Tips reißen oder aus dem Dispenser gedrückt werden.
- Das auf den Mischblock ausgebrachte Material sollte mit einer Lichtschutzfolie vor Umgebungslicht geschützt werden, damit es nicht zu einer unbeab-sichtigten Polymerisation des Materials kommt.
- Nach jedem Gebrauch die Verschlusskappe wieder sicher befestigen.
8. Lichthärtung
Mit einer dentalen Polymerisationslampe lichthärten.
- Bei tiefen Kavitäten ist eine inkrementelle Füllung und Lichthärtung mit maximal 2 mm Schichtstärke zu empfehlen.
- Großflächige Füllungen in Segmenten lichthärten.
- Polymerisationslampe und Polymerisationszeit

Polymerisationslampe	Halogen	LED
Polymerisationszeit	20 Sek.	10 Sek.

- Halogen-Polymerisationslampe
Wellenlänge: 400-500 nm,
Lichtleistung: ≥ 500 mW/cm^2
- LED-Polymerisationslampe
Wellenlänge: 440-490 nm,
Lichtleistung: ≥ 1000 mW/cm^2
- Eine dentale Polymerisationslampe mit den obigen technischen Merkmalen benutzen. Bei Verwendung von Modellen mit anderen Leistungsmerkmalen die Gebrauchsanleitung der jeweiligen Lampe befolgen, damit BEAUTIFIL II korrekt lichtgehärtet wird.
- Der Behandler muss eine Schutzbrille tragen oder ein Lichtschutzschild verwenden, um nicht unmittelbar in das Licht der Polymerisationslampe zu blicken. Der Behandler muss auch die Augen des Patienten vor direkter Einwirkung des Lichts der Polymerisationslampe schützen.
- Die Spritze nach jedem Gebrauch mit Alkohol desinfizieren, um Kreuzkontaminationen zu vermeiden. Anschließend die Verschlusskappe fest wieder anbringen. Achtgeben, dass kein Alkohol in die Paste gelangt.
- Die Polymerisationslampe nach jedem Gebrauch mit Alkohol desinfizieren, um Kreuzkontaminationen zu vermeiden.
9. Finieren und Polieren
Nach der Lichthärtung die Füllung wie gewohnt konturieren, finieren und polieren.

HINWEISE ZUM GEBRAUCH
1. Bei allen zusammen mit diesem Produkt verwendeten zahnärztlichen Materialien oder Instrumenten die Gebrauchsanleitung beachten.
2. Das Produkt ausschließlich für die im Abschnitt INDIKATIONEN dieses Beipackzettels genannten Anwendungen verwenden.
3. Das Produkt nur bis zu dem auf Packung und Behälter angegebenen Verfallsdatum verwenden.
(Beispiel ⌛ JJJJ-MM-TT → Jahr-Monat-Tag des Verfallsdatums)

LAGERUNG
Bei Zimmertemperatur lagern (1-30°C). Hohe Temperaturen und hohe Luftfeuchtigkeit vermeiden. Vor direkter Sonneneinstrahlung schützen und von Zündquellen fernhalten.

FR A LIRE ATTENTIVEMENT AVANT USAGE

Matériau de restauration à libération de fluor

BEAUTIFIL II est un matériau de restauration radio opaque photopolymérisable destiné aux restaurations antérieures et postérieures.

TEINTES
Seringue: A1, A2, A3, A3.5, A4, B1, B2, B3, C2, C3, A2O, A3O, Inc (Incisal), BW (Bleaching White)
Carpule: A1, A2, A3, A3.5, A4, B1, B2, C2, A2O, A3O, Inc, BW

COMPOSITION
Bis-GMA, TEGDMA, Charge S-PRG à base de verre fluoroboroaluminosilicate, initiateur de polymérisation, pigments et divers

INDICATIONS
- Restaurations antérieures et postérieures
- Facette directe
- Réparation de restaurations et prothèses
- Reconstitution

PRECAUTIONS
1. Ne pas utiliser ce produit pour des patients allergiques à ce matériau ou aux monomères acryliques.
2. De même le personnel allergique à ces produits ne devra pas les manipuler.
3. Si, lors de l'utilisation du produit, le patient ou l'opérateur présente une inflammation ou autre réaction allergique, interrompre l'opération et consulter un médecin.
4. Ne jamais réutiliser les carpules.
5. Porter des gants et des lunettes de protection afin d'éviter toute sensibilisation. En cas de contact accidentel avec les tissus mous, la peau ou un vêtement, essuyer avec un coton imbibé d'alcool et rincer à l'eau courante. Bien que les gants médicaux protègent du contact direct, certains monomères peuvent les traverser. Si c'est le cas, ôter immédiatement les gants, se laver les mains à l'eau courante.
6. En cas de contact oculaire, rincer à l'eau et consulter un médecin.
7. Ce produit est exclusivement réservé à un usage dentaire professionnel.

MODE D'EMPLOI
1. Nettoyage de la surface de la dent
Procéder soigneusement pour ôter toute trace de plaque.
2. Teinte
Sélectionner la teinte appropriée du matériau de restauration à l'aide d'un teintier pendant que la dent reste humide.
3. Préparation de la cavité
Préparer la cavité de façon conventionnelle.
4. Isolation
Isoler avec une digue.
5. Protection de la pulpe
En cas de cavité profonde, proche de la pulpe, la protéger en l'enduisant d'un hydroxyde de calcium.
- Ne pas utiliser de produit contenant de l'eugenol car cela nuirait à l'adhérence et à la polymérisation du matériau.
6. Conditionnement et application de l'adhésif
Passer un adhésif dentinaire en suivant les indications du fabricant.
7. Placement
A l'aide de la seringue, déposer la quantité désirée de BEAUTIFIL II sur un bloc de mélange. Appliquer le produit dans la cavité à l'aide d'un instrument adéquat et sculpter la forme souhaitée.
Si l'on utilise les carpules, en insérer une dans le pistolet distributeur pour une application directe dans la cavité. Avec la carpule on peut aussi déposer le produit sur un bloc de mélange.
- S'il est stocké au réfrigérateur, laisser le matériau atteindre la température ambiante avant usage.
- En cas d'application directe dans la cavité, stériliser le pistolet distributeur avant et après utilisation.
- Les carpules sont à usage unique et donc à jeter après chaque intervention, pour éviter la contamination croisée.
- Appuyer doucement sur le distributeur pour distribuer lentement le matériau. Une pression excessive peut provoquer la rupture des pointes ou leur éjection du distributeur.
- La pâte sur le bloc de mélange doit être protégée de la lumière ambiante pour éviter un début de polymérisation indésirable.
- Reboucher tout soigneusement après chaque utilisation.
8. Photopolymérisation
Photopolymériser avec une lampe de cabinet
- En cas de cavité profonde, procéder en plusieurs étapes (application/polymérisation) sans excéder 2 mm d'épaisseur à chaque fois.
- Pour les cavités de grande surface, photopolymériser par segments.
- Lampe à photopolymériser et temps d'insolation

Lampe à photopolymériser	Halogène	LED
Temps d'insolation	20 s	10 s

- Lampe halogène
longueur d'onde : 400-500 nm,
intensité lumineuse : ≥ 500 mW/cm^2
- Lampe à LED
longueur d'onde : 440-490 nm,
intensité lumineuse : ≥ 1000 mW/cm^2
- Utiliser une lampe de cabinet aux spécifications identiques à celles indiquées plus haut. Si une lampe présente des performances différentes de celles décrites, suivre son mode d'emploi pour polymériser correctement BEAUTIFIL II.
- Utiliser des lunettes de protection et éviter de regarder directement la lumière de la lampe à polymériser. Les opérateurs doivent aussi protéger les yeux du patient d'une exposition directe à la lumière de la lampe à polymériser.
- Désinfecter à l'alcool la seringue après chaque patient pour éviter la contamination croisée. Puis reboucher soigneusement. User de prudence pour éviter de mettre de l'alcool dans la pâte.
- Désinfecter à l'alcool la lampe à polymériser après chaque patient pour éviter la contamination croisée.
9. Finition et polissage
Après polymérisation, procéder au modelage, à la finition et au polissage de manière conventionnelle.

REMARQUES
1. Suivre les instructions du fabricant pour chaque matériau ou instrument utilisé durant la mise en œuvre de ce produit.
2. Ne pas utiliser ce produit à d'autres fins que celles mentionnées au chapitre INDICATIONS du présent mode d'emploi.
3. Utiliser ce produit avant la date d'expiration indiquée sur l'étiquette et l'emballage.
(Exemple ⌛ AAAA-MM-JJ → Année-Mois-Jour de la date d'expiration)

STOCKAGE
Conserver à température ambiante (1-30 °C). Eviter toute température ou humidité élevée. Ne pas approcher d'une source lumineuse intense ou d'une flamme.

NL LEES ZORGVULDIG VOOR GEBRUIK

Fluoride afgevend restauratiemateriaal

BEAUTIFIL II is een lichtuithardend radiopaak restauratiemateriaal, geïndiceerd voor anterieure en posterieure restauraties.

KLEUREN
Syringe: A1, A2, A3, A3.5, A4, B1, B2, B3, C2, C3, A2O, A3O, Inc (snijtanden), BW (bleekwit)
Tips: A1, A2, A3, A3.5, A4, B1, B2, C2, A2O, A3O, Inc, BW

SAMENSTELLING
Bis-GMA, TEGDMA, S-PRG vulstof op basis van fluoroboroaluminosilicaatglas, polymerisatie-initiator, pigmenten en andere

INDICATIES
- Anterieure en posterieure restauraties
- Direct laminaat veneer
- Reparatie van restauraties en prothesen
- Kernopbouw

VOORZORGSMAATREGELEN
1. Gebruik dit product niet bij patiënten met een bekende allergie voor dit materiaal en/of methacrylaatmonomeer.
2. Gebruikers met een bekende allergie voor dit materiaal en/of methacrylaatmonomeer mogen dit product niet gebruiken.
3. Als een ontsteking of een andere allergische reactie ontstaat bij de patiënt of de gebruiker dient het gebruik onmiddellijk te worden gestopt en moet medisch advies worden ingewonnen.
4. Gebruik de tips niet een tweede maal.
5. Gebruik medische (dentale) handschoenen en een beschermbril om de ontwikkeling van gevoeligheid voor dit product te voorkomen. Bij accidenteel contact met orale zachte weefsels of met de huid moet onmiddellijk met een met alcohol bevochtigde wattenpellet worden gedept en met ruim water worden gespoeld. Medische (dentale) handschoenen bieden enige bescherming tegen direct contact met dit materiaal; sommige monomeren kunnen echter zeer snel door de handschoenen penetreren en de huid bereiken. Derhalve moeten, mocht het materiaal in aanraking komen met de handschoenen, deze onmiddellijk worden uitgetrokken en moeten de handen met ruim water worden gewassen.
6. Bij contact met de ogen moeten deze onmiddellijk met ruim water worden gespoeld en moet medisch advies worden ingewonnen.
7. Dit product is bedoeld om uitsluitend door tandartsen te worden gebruikt.

GEBRUIKSAANWIJZING
1. Reinigen van het tandoppervlak
Reinig het oppervlak van het element grondig om de plak te verwijderen.
2. Kleurselectie
Kies de juiste kleur aan de hand van een kleurenring wanneer het element nog vochtig is.
3. Voorbereiden van de caviteit
Prepareer de caviteit op conventionele wijze.
4. Isolatie
Isoleer met een rubberdam.
5. Bescherming van de pulpa
Overkap de pulpa met calciumhydroxide etc. wanneer deze is geëxposeerd of als het aangetaste gebied dicht bij de pulpa ligt.
- Gebruik geen eugenol bevattende materialen daar deze de polymerisatie of adhesie van het materiaal nadelig kunnen beïnvloeden.
6. Conditionering en toepassing van het adhesief
Applicer dentineadhesief volgens de gebruiksaanwijzing van de fabrikant.
7. Plaatsing
Dispenseer vanuit de syringe een adequate hoeveelheid BEAUTIFIL II op een papieren mengblok. Plaats het gedispenseerde materiaal met een geschikt instrument in de caviteit en geef er de gewenste vorm aan.
Als een tip wordt gebruikt, plaats deze dan in de dispenser om het materiaal direct in de caviteit te brengen. Het tipmateriaal kan ook op een papieren mengblok worden geplaatst om met een geschikt instrument naar de caviteit te worden gebracht.
- Laat, wanneer deze materialen in de koelkast worden bewaard, ze afkoemen tot ze gebruiken op kamertemperatuur komen.
- Steriliseer de dispenser voor en na de behandeling wanneer het materiaal direct in de caviteit wordt geplaatst.
- Gebruik de tip voor slechts een enkele patiënt om kruisbesmetting te voorkomen en gooi hem weg na de behandeling.
- Hanteer de dispenser rustig om het materiaal langzaam te dispenseren. Overmatige druk kan oorzaak zijn dat de tip breekt of loskomt.
- Het op het mengblok gedispenseerde materiaal moet met een lichtafdekking tegen omgevingslicht worden afgeschermd om voortijdige polymerisatie te voorkomen.
- Draai na elk gebruik de dop stevig vast.
8. Uitharden onder licht
Hard uit onder licht met een dentale polymerisatielamp.
- Bij een diepe caviteit wordt aanbevolen lagen van niet meer dan 2 mm te maken en uit te harden.
- Aanbevolen wordt bij een caviteit met een groot oppervlak in segmenten uit te harden.
- Dentale polymerisatielamp en -tijd

Polymerisatielamp	Halogeen	LED
Polymerisatietijd	20 sec.	10 sec.

- Halogeen polymerisatielamp
Golflengte: 400-500 nm,
Lichtintensiteit: ≥ 500 mW/cm^2
- LED-polymerisatielamp
Golflengte: 440-490 nm,
Lichtintensiteit: ≥ 1000 mW/cm^2
- Gebruik een polymerisatielamp waarvan de specificaties overeenkomen met de hierboven genoemde waarden. Volg bij gebruik van een polymerisatielamp met afwijkende specificaties de gebruiksaanwijzing om BEAUTIFIL II correct uit te harden.
- De gebruiker moet een beschermbril of een lichtafdekking gebruiken om te voorkomen dat direct in het licht van de polymerisatielamp gekeken kan worden. De gebruiker dient ook de ogen van de patiënt te beschermen tegen directe blootstelling aan het licht van de polymerisatielamp.
- Desinfecteer de doseerspuit na elk gebruik met alcohol om kruisbesmetting te voorkomen. Sluit vervolgens de dop stevig. Wees voorzichtig en voorkom dat er alcohol in het materiaal terechtkomt.
- Desinfecteer de polymerisatielamp na elk gebruik met alcohol om kruisbesmetting te voorkomen.
9. Afwerken en polijsten
Contoureer, finish en polijst de restauratie na het uitharden op conventionele wijze.

AANWIJZINGEN BIJ HET GEBRUIK
1. Volg voor elk materiaal of instrument dat in combinatie met dit product wordt gebruikt de betreffende gebruiksaanwijzing.
2. Gebruik dit product niet voor enig ander doel dan specifiek vermeld onder INDICATIES in deze gebruiksaanwijzing.
3. Gebruik dit product voor de vervaldatum die is aangegeven op de verpakking en de houder.
(Bijvoorbeeld ⌛ JJJJ-MM-DD → Jaar-Maand-Dag van de uiterste gebruiksdatum)

OPSLAG
Te bewaren op kamertemperatuur (1-30 °C). Vermijd hoge temperaturen en grote vochtigheid. Houd verwijderd van direct zonlicht en van enige ontstekingsbron.

ES LEER CUIDADOSAMENTE ANTE DE USARLO

Material de restauración con liberación de flúor

BEAUTIFIL II es un material de restauración fotopolimerizable y radiopaco indicado para restauraciones anteriores y posteriores.

TONOS
Jeringa: A1, A2, A3, A3.5, A4, B1, B2, B3, C2, C3, A2O, A3O, Inc (Incisal), BW (Blanqueador)
Puntas: A1, A2, A3, A3.5, A4, B1, B2, C2, A2O, A3O, Inc, BW

COMPOSICIÓN
Bis-GMA, TEGDMA, relleno S-PRG a base de vidrio de fluoroboroaluminosilicato, iniciador de la polimerización, pigmentos y otros

INDICACIONES
- Restauraciones anteriores y posteriores
- Carillas laminadas directas
- Reparación de restauraciones y prótesis
- Reconstrucción de muñones

PRECAUCIONES
1. No usar este producto en pacientes con alergias conocidas a este material y/o al monómero de metacrilato.
2. Los profesionales con alergias conocidas a este material y/o al monómero de metacrilato no deben usar este producto.
3. Si apareciera inflamación o cualquier otra reacción alérgica en el paciente o el profesional mientras se está utilizando este producto, parar su uso inmediatamente y buscar ayuda médica.
4. No reutilizar las puntas.
5. Usar guantes médicos (dentales) y gafas protectoras para evitar la sensibilización a este producto. En caso de contacto accidental con el tejido blando oral o la piel, limpiar inmediatamente con una bolita de algodón humedecida en alcohol y lavar con gran cantidad de agua. Los guantes médicos (dentales) proporcionan algo de protección contra el contacto directo con este material; sin embargo algunos monómeros pueden penetrar a través de los guantes muy rápidamente hasta la piel. Por tanto, si el material contactara con los guantes, quitárselos inmediatamente y lavarse las manos con gran cantidad de agua.
6. En caso de contacto con los ojos, lavar inmediatamente los ojos con gran cantidad de agua y buscar ayuda médica.
7. Este producto está fabricado para uso solo por profesionales dentales.

INSTRUCCIONES DE USO
1. Limpieza de la superficie del diente
Limpiar completamente la superficie del diente para eliminar la placa.
2. Selección del tono
Seleccionar el tono apropiado con la guía de tonos mientras el diente aún está húmedo.
3. Preparación de la cavidad
Preparar la cavidad según el método convencional.
4. Aislamiento
Aislar con un dique de goma.
5. Protección de la pulpa
Recubrir la pulpa con hidróxido de calcio cuando la cavidad sea profunda o se exponga la pulpa.
- No usar materiales que contengan eugenol, ya que pueden inhibir la polimerización o la adhesión del material.
6. Acondicionamiento y aplicación del adhesivo
Aplicar el adhesivo dentinario según las instrucciones de uso del fabricante.
7. Colocación
Dispensar de la jeringa una cantidad adecuada de BEAUTIFIL II en un papel de mezcla. Aplicar el material dispensado en la cavidad con un instrumento apropiado y crear la forma deseada.
Si usamos la punta, insertarla en el dispensador para aplicar el material directamente en la cavidad. También se puede dispensar el material con la punta en un papel de mezcla para ser aplicado dentro de la cavidad con un instrumento adecuado.
- Cuando el material está guardado en la nevera, esperar a que alcance la temperatura ambiente antes de usarlo.
- Cuando se aplique el material directamente en la cavidad, esterilizar el dispensador antes y después de su uso.
- La punta debe ser usada solo en un paciente y luego tirarla al final del tratamiento para evitar la contaminación cruzada.
- Agarrar el dispensador con suavidad para dispensar el material lentamente. Si se ejerce una presión excesiva, las puntas pueden romperse o salir expulsadas del dispensador.
- La pasta dispensada en el papel de mezcla debe protegerse de la luz ambiente con una cubierta de protección de la luz para evitar la polimerización del material.
- Cerrar correctamente el tapón después de cada uso.
8. Fotopolimerización
Fotopolimerizar con una lámpara dental de fotopolimerización.
- Si la cavidad es profunda, se recomienda realizar un relleno incremental y una fotopolimerización no superior a 2 mm.
- Si la superficie de la cavidad es grande, fotopolimerizar por segmentos.
- Lámpara dental de fotopolimerización y tiempo de fotopolimerización

Unidad de fotopolimerización	Halógena	LED
Tiempo de fotopolimerización	20 segundos	10 segundos

- Unidad halógena de fotopolimerización
Longitud de onda: 400-500 nm,
Intensidad lumínica: ≥ 500 mW/cm^2
- Unidad LED de fotopolimerización
Longitud de onda: 440-490 nm,
Intensidad lumínica: ≥ 1.000 mW/cm^2
- Usar una lámpara dental de fotopolimerización con las mismas especificaciones que aquellas descritas anteriormente. Si se usa otro tipo de lámpara de fotopolimerización, seguir las instrucciones de cada una para fotopolimerizar correctamente el BEAUTIFIL II.
- Los operadores deben usar gafas protectoras o plástico protector para evitar mirar directamente a la luz de fotopolimerización. Los operadores también deben proteger los ojos del paciente para evitar su exposición directa a la luz de fotopolimerización.
- Desinfectar la jeringa con alcohol después de cada uso para evitar la contaminación cruzada. A continuación, cerrar correctamente el tapón. Proceder con precaución para evitar que penetre alcohol en la pasta.
- Desinfectar la lámpara de fotopolimerización con alcohol después de cada uso para evitar la contaminación cruzada.
9. Acabado y pulido
Después de la fotopolimerización, contornear, terminar y pulir la restauración siguiendo la técnica convencional.

NOTA DE USO
1. Seguir las instrucciones de uso de cada material dental o instrumento que se va a usar junto con este producto.
2. No usar este producto para otras indicaciones que no sean las especificadas en las INDICACIONES de estas instrucciones de uso.
3. Usar este producto dentro de la fecha de caducidad indicada en envase y el embalaje.
(Ejemplo ⌛ AAAA-MM-DD → Año-Mes-Día de la fecha de caducidad)

ALMACENAJE
Almacenar a temperatura ambiente (1-30 °C). Evitar temperatura y humedad altas. Mantenerlo alejado de la luz directa del sol y de cualquier fuente de calor.

IT LEGGERE ATTENTAMENTE PRIMA DELL'USO

Materiale ricostruttivo a rilascio di fluoro

BEAUTIFIL II è un materiale ricostruttivo radiopaco fotopolimerizzato indicato per il restauro anteriore e posteriore.

COLORI
Siringa: A1, A2, A3, A3.5, A4, B1, B2, B3, C2, C3, A2O, A3O, Inc(Incisal), BW(Bleaching White)
Puntali: A1, A2, A3, A3.5, A4, B1, B2, C2, A2O, A3O, Inc, BW

COMPOSIZIONE
Bis-GMA, TEGDMA, riempitivo S-PRG a base di vetro silicato di alluminio fluoroborico, iniziatore della polime-rizzazione, pigmenti e altro

INDICAZIONI
- Restauri anteriori e posteriori
- Faccetta dentale rivestita diretta
- Riparazione di restauri e protesi
- Realizzazione monconi protesici

MISURE DI SICUREZZA
1. Non usare questo prodotto su pazienti con allergie note al materiale e/o al monomero metacrilato.
2. Il prodotto non deve essere usato da operatori con allergie note al materiale e/o al monomero metacrilato.
3. Se un paziente o un operatore dovesse presentare infiammazioni o altre reazioni allergiche, interrompere immediatamente l'uso del prodotto e consultare un medico.
4. Non riutilizzare i puntali.
5. Indossare guanti protettivi medicali e occhiali protettivi per evitare la sensibilizzazione a questo prodotto. In caso di contatto accidentale con i tessuti molli della cavità orale o con la pelle, pulire immediatamente con un batuffolo di cotone imbevuto d'alcol e risciacquare abbondantemente con acqua. I guanti protettivi medicali forniscono protezione dal contatto diretto con il prodotto, ma alcuni monomeri possono penetrare molto velocemente attraverso il guanto e arrivare alla pelle. Qualora il prodotto entrasse a contatto con i guanti, toglierli immediatamente e lavare le mani con abbondante acqua.
6. In caso di contatto con gli occhi, risciacquare immediatamente con abbondante acqua e consultare un medico.
7. Questo prodotto è destinato esclusivamente all'uso odontoiatrico professionale.

ISTRUZIONI PER L'USO
1. Pulizia della superficie dentaria
Pulire accuratamente le superfici dentarie dalla placca.
2. Scelta del colore
Scegliere il colore adatto avvalendosi di una scala colori, finché il dente è ancora umido.
3. Preparazione della cavità
Preparare la cavità come di consueto.
4. Isolamento
Isolare con una diga di gomma.
5. Protezione della polpa
In caso di esposizione pulpare o qualora l'area interessata si trovi vicino alla polpa, eseguire un incappucciamento con idrossido di calcio, ecc.
- Non usare materiali contenenti eugenolo, poiché potrebbero inibire la polimerizzazione o l'adesione del materiale.
6. Condizionamento e applicazione di adesivo
Applicare un adesivo dentinale secondo le istruzioni del produttore.
7. Posizionamento
Erogare una quantità sufficiente di BEAUTIFIL II dalla siringa sul blocchetto di carta. Applicare il materiale erogato nella cavità utilizzando uno strumento adatto e modellare come desiderato.
Nell'utilizzo di un puntale, introdurlo nel dispenser per applicare il materiale direttamente nella cavità. Anche il materiale del puntale può essere erogato su un blocchetto di carta e applicato nella cavità con uno strumento adatto.
- Utilizzare il materiale conservato nel frigorifero solo quando ha raggiunto la temperatura ambiente.
- Nell'applicazione diretta del materiale nella cavità sterilizzare il dispenser prima e dopo l'uso.
- Per evitare contaminazioni crociate, i puntali devono essere usati per un solo paziente e smaltiti al termine del trattamento.
- Impugnare il dispenser con delicatezza per erogare il materiale gradualmente. Un'eccessiva pressione potrebbe causare la rottura dei puntali o la loro espulsione dal dispenser.
- Il materiale erogato sul blocco di carta deve essere protetto dalla luce circostante con un apposito schermo fotoprotettivo in modo da evitarne la polimerizzazione indesiderata.
- Richiudere bene il cappuccio dopo ogni uso.
8. Fotopolimerizzazione
Fotopolimerizzare utilizzando un'unità fotopolimerizzante dentale.
- Se la cavità è profonda, si consiglia un incremento del riempimento e della fotopolimerizzazione non superiore a 2 mm.
- Quando la superficie della cavità è di grandi dimensioni, eseguire la fotopolimerizzazione in segmenti.
- Unità fotopolimerizzante dentale e tempo di fotopolimerizzazione

Unità fotopolimerizzante	Alogena	LED
Tempo di fotopolimerizzazione	20 s	10 s

- Unità fotopolimerizzante alogena
lunghezza d'onda: 400-500 nm,
intensità luminosa: ≥ 500 mW/cm^2
- Unità fotopolimerizzante LED
lunghezza d'onda: 440-490 nm,
intensità luminosa: ≥ 1000 mW/cm^2
- Usare un'unità fotopolimerizzante dentale con le caratteristiche tecniche sopra descritte. Per la corretta fotopolimerizzazione di BEAUTIFIL II con unità aventi caratteristiche diverse da quelle indicate, attenersi alle rispettive istruzioni.
- Gli operatori devono indossare occhiali protettivi o usare uno schermo fotoprotettivo per evitare di guardare direttamente il fotopolimerizzatore. Gli operatori sono inoltre tenuti a proteggere gli occhi dei pazienti dall'esposizione diretta al fotopolimerizzatore.
- Per evitare contaminazione crociata, disinfettare la siringa con alcol dopo ogni uso. Quindi richiudere bene il cappuccio. Fare attenzione affinché l'alcol non venga introdotto nel materiale.
- Per evitare contaminazione crociata, disinfettare l'unità fotopolimerizzante con alcol dopo ogni uso.
9. Rifinitura e lucidatura
Dopo la fotopolimerizzazione, il restauro viene sagomato, rifinito e lucidato come di consueto.

NOTE SULL'USO
1. Per ogni materiale o strumento dentale utilizzato in combinazione con questo prodotto seguire le istruzioni del produttore.
2. Usare il prodotto esclusivamente per lo scopo indicato nella sezione INDICAZIONI di queste istruzioni.
3. Utilizzare il prodotto entro la data di scadenza indicata sulla confezione e sul contenitore.
(Esempio ⌛ AAAA-MM-GG → Anno-Mese-Giorno della data di scadenza)

CONSERVAZIONE
Conservare a temperatura ambiente (1-30 °C). Evitare umidità e temperature elevate. Proteggere da irradiazione solare diretta e tenere lontano da fonti d'accensione.

RU ВНИМАТЕЛЬНО ПРОЧЕСТЬ ПЕРЕД ПРИМЕНЕНИЕМ

Выделяющий фтор реставрационный материал

BEAUTIFIL II –это светоотверждаемый рентгеноконтрастный реставрационный материал, предназначенный для реставраций фронтальных и боковых зубов.

ОТТЕНКИ
Шприц: A1, A2, A3, A3.5, A4, B1, B2, B3, C2, C3, A2O, A3O, Inc (Incisal/режущий край), BW (Bleaching White/отбеленные зубы)
Капсулы: A1, A2, A3, A3.5, A4, B1, B2, C2, A2O, A3O, Inc, BW

СОСТАВ
Bis-GMA/бисфенол А-глицидил-метакрилат, TEGDMA/триэтиленгликольдиметакрилат, наполнитель S-PRG на основе фторборалюминийсиликатного стекла, инициатор полимеризации, пигменты и др.

ПОКАЗАНИЯ
- Реставрация фронтальных и боковых зубов
- Прямые многослойные виниры
- Ремонт реставраций и протезов
- Восстановление культи зуба (Core-build up)

МЕРЫ ПРЕДОСТОРОЖНОСТИ
1. Не следует применять данный продукт для пациентов, у которых ранее наблюдались аллергические реакции на этот материал и/или метакрилатные мономеры.
2. Персонал, у которого ранее наблюдались аллергические реакции на этот материал и/или метакрилатные мономеры, не должен использовать этот продукт.
3. При возникновении у персонала или пациента воспалительных или других аллергических реакций следует немедленно прекратить применение данного продукта и обратиться за консультацией к врачу.
4. Капсулы нельзя использовать повторно.
5. Для предотвращения сенсибилизации на продукт следует использовать защитные медицинские (стоматологические) перчатки и очки. При случайном контакте с мягкими тканями полости рта или с кожей следует немедленно промокнуть ватным тампоном, пропитанным спиртом, и прополоскать большим количеством воды. Медицинские (стоматологические) перчатки обеспечивают определенную защиту от прямого контакта с этим материалом, однако некоторые мономеры могут очень быстро проникать через перчатки на кожу. Это значит, что даже в случае попадания материала на защитные перчатки их следует немедленно снять и вымыть руки большим количеством воды.
6. При контакте с глазами необходимо немедленно промыть их большим количеством воды и обратиться за консультацией к врачу.
7. Этот продукт предназначен только для применения специалистами в стоматологии.

ИНСТРУКЦИЯ ПО ПРИМЕНЕНИЮ
1. Очистка поверхности зуба
Тщательно очистить поверхность зуба от отложений.
2. Выбор оттенка
С помощью шкалы оттенков выбрать необходимый оттенок, пока зуб еще влажный.
3. Препарирование кариозной полости
Препарировать полость обычным способом.
4. Изолированность
Изолировать с помощью резинового изолятора слюны.
5. Защита пульпы
В случае когда пульпа открыта или пораженная зона расположена близко к пульпе, закрыть пульпу гидроокисью кальция и др.
   - Не использовать материалы, содержащие эвгенол, поскольку они влияют на качество полимеризации или адгезии материала.
6. Обработка и нанесение адгезива
Нанести адгезив для дентина согласно инструкции производителя.
7. Нанесение
На блок для замешивания дозировать из шприца достаточное количество BEAUTIFIL II. Дозированный материал нанести соответствующим инструментом в полость и моделировать, как это необходимо.
При использовании капсулы установить ее в диспенсер и наносить материал непосредственно в полость. Однако капсульный материал может быть также дозирован на блок для замешивания и нанесен в полость соответствующим инструментом.
   - Хранящийся в холодильнике материал перед применением нагреть до комнатной температуры.
   - При прямом нанесении материала в полость стерилизовать диспенсер до и после использования.
   - Капсулы должны использоваться только для одного пациента и утилизироваться после окончания процедуры во избежание перекрестной инфекции.
   - Держать диспенсер осторожно, чтобы материал наносился медленно. При излишнем давлении капсула может разорваться или выпасть из диспенсера.
   - Дозированную на блок для замешивания пасту защищать от окружающего света светозащитной пленкой, чтобы избежать случайной полимеризации материала.
   - Плотно закрывать крышку после каждого использования.
8. Светополимеризация
Полимеризация с помощью дентальной светополимеризационной лампы.
   - В случае глубокой полости рекомендуется нанесение материала дозами и светополимеризация слоя не более 2 мм.
   - В случае большой полости рекомендуется проводить светополимеризацию сегментами.
   - Дентальная светополимеризационная лампа и время светополимеризации

Дентальная светополимеризационная лампа	Галогеновый	Светодиодный
Время светополимеризации	20 с	10 с

   - Галогенная светополимеризационная лампа длина волн: 400–500 нм, интенсивность света: ≥ 500 мВт/см^2
   - Светодиодная светополимеризационная лампа длина волн: 440–490 нм, интенсивность света: ≥ 1000 мВт/см^2
   - Использовать дентальную светополимеризационную лампу с указанными выше техническими характеристиками. При использовании моделей с другими параметрами мощности следовать инструкции по эксплуатации конкретной лампы, чтобы выполнить надлежащую светополимеризацию BEAUTIFIL II.
   - Персонал должен пользоваться защитными очками или защитным щитком, чтобы не смотреть непосредственно на свет светополимеризационной лампы. Персонал должен также защитить глаза пациента от прямого попадания полимеризационного света.
   - Дезинфицировать шприц спиртом после каждого использования во избежание перекрестного заражения. Затем плотно закрыть колпачок. Следует быть осторожным, чтобы избежать попадания спирта в пасту.
   - Дезинфицировать дентальную светополимеризационную лампу спиртом после каждого использования во избежание перекрестного заражения.
9. Финирование и полировка
После светополимеризации контурировать, финировать и полировать реставрацию обычным способом.

РЕКОМЕНДАЦИИ ПО ПРИМЕНЕНИЮ
1. Следовать инструкциям по применению стоматологических материалов или инструментов, используемых с этим продуктом.
2. Применять продукт исключительно для целей, указанных в разделе ПОКАЗАНИЯ этой инструкции по применению.
3. Использовать данный продукт только до истечения указанного на упаковке и емкости срока годности.
(Пример ⌛ ГГГГ-ММ-ДД → год-месяц-дата окончания срока годности)

ХРАНЕНИЕ
Хранить при комнатной температуре (1–30°C). Избегать воздействия высоких температур и высокой влажности. Защищать от прямых солнечных лучей, хранить вдали от источников возгорания.

PL NALEŻY DOKŁADNIE PRZECZYTAĆ PRZED UŻYCIEM

Uwalniający fluor materiał wypełnieniowy

BEAUTIFIL II to światłoutwardzalny, radionieprzezierny materiał wypełnieniowy przeznaczony do odbudowy zębów przednich i tylnych.

ODCIENIE
Strzykawki: A1, A2, A3, A3.5, A4, B1, B2, B3, C2, C3, A2O, A3O, Inc (do brzegów siecznych), BW (do zębów wybielanych)
Końcówka: A1, A2, A3, A3.5, A4, B1, B2, C2, A2O, A3O, Inc, BW

SKŁAD
Bis-GMA, TEGDMA wypełniacz S-PRG na bazie szkła fluoro-boro-glinowo-krzemowego, inicjator polimeryzacji, pigmenty i inne

WSKAZANIA
- Wypełnienia zębów przednich i tylnych
- Licówki wykonywane bezpośrednio
- Naprawa wypełnień i protez
- Odbudowa zrębu zębów

ŚRODKI OSTROŻNOŚCI
1. Nie należy używać tego produktu u pacjentów ze zdiagnozowaną alergią na ten materiał i/lub monomer metakrylu.
2. Niniejszego produktu nie powinni stosować operatorzy z rozpoznaną alergią na ten materiał i/lub monomer metakrylu.
3. W przypadku wystąpienia u pacjenta stanu zapalnego lub innego rodzaju reakcji alergicznej należy niezwłocznie zaprzestać stosowania i zasięgnąć porady lekarza.
4. Nie należy powtórnie używać końcówki.
5. Aby uniknąć uczulenia na produkt, należy używać rękawiczek medycznych (dentystycznych) i okularów ochronnych. W sytuacji przypadkowego kontaktu z miękkimi tkankami jamy ustnej lub skórą należy natychmiast przetrzeć skórę kłębkiem waty zwilżonej alkoholem i spłukać dużą ilością wody. Rękawiczki medyczne (dentystyczne) zapewniają pewną ochronę przeciwko bezpośredniemu kontaktowi z materiałem, jednakże niektóre monomery mogą szybko penetrować przez rękawiczki i dosięgnąć skóry. Jeśli materiał wszedł w kontakt z rękawiczkami, należy natychmiast je zdjąć, a ręce umyć dużą ilością wody.
6. W sytuacji kontaktu z oczami należy natychmiast przemyć je dużą ilością wody i zasięgnąć porady lekarza.
7. Niniejszy produkt jest przeznaczony wyłącznie do użytku przez lekarzy dentystów.

INSTRUKCJA STOSOWANIA
1. Oczyszczenie powierzchni zęba
Należy dokładnie oczyścić powierzchnię zęba z płytki nazębnej.
2. Wybór odcienia
Należy wybrać odcień za pomocą kolornika, gdy ząb jest jeszcze mokry.
3. Opracowanie ubytku
Należy opracować ubytek w konwencjonalny sposób.
4. Odizolowanie zęba
Należy odizolować ząb przy użyciu ślinochronu.
5. Zabezpieczenie miazgi
W przypadku odkrycia miazgi lub bliskiej odległości ubytku od miazgi należy pokryć miazgę za pomocą wodorotlenku wapnia itd.
   - Nie stosować materiałów zawierających eugonol, ponieważ może hamować polimeryzację i adhezję materiału.
6. Dopasowywanie i nakładanie systemu wiążącego
Należy nałożyć system wiążący zgodnie z zaleceniami producenta.
7. Nanoszenie
Odmierzyć ze strzykawki odpowiednią ilość BEAUTIFIL II na papierek. Nałożyć odmierzoną ilość materiału do ubytku za pomocą odpowiednich narzędzi i stworzyć pożądany kształt.
Podczas używania końcówek należy umieścić końcówkę w pistolecie, aby nałożyć materiał bezpośrednio do ubytku. Materiał z końcówki może być także wyciśnięty na papierek i nałożony do ubytku odpowiednimi narzędziami.
   - Jeśli produkt jest przechowywany w lodówce, należy pozwolić, aby produkt osiągnął temperaturę pokojową przed użyciem.
   - Jeśli materiał jest nakładany bezpośrednio do ubytku, należy sterylizować pistolet przed i po użyciu.
   - Końcówka powinna być używana tylko u jednego pacjenta i wyrzucona po zakończonym zabiegu, aby uniknąć zakażeń krzyżowych.
   - Należy delikatnie chwycić pistolet, aby powoli nanieść materiał. Nadmierny nacisk może spowodować pęknięcie lub wysunięcie końcówki z pistoletu.
   - Materiał odmierzony na papierku powinien być zasłonięty przed działaniem naturalnego światła pokrywką, aby uniknąć niezamierzonego spolimeryzowania materiału.
   - Należy szczelnie zamknąć zakrętkę po każdym użyciu.
8. Utwardzanie światłem
Należy utwardzać światłem przy użyciu lampy polimeryzacyjnej.
   - W przypadku głębokich ubytków zalecane jest stopniowe nanoszenie kolejnych warstw i utwardzanie w odległości nieprzekraczającej 2 mm.
   - W przypadku dużej powierzchni ubytku należy utwardzać w odcinkach.
   - Lampa polimeryzacyjna i czas utwardzania światłem

Lampa polimeryzacyjna	Halogen	LED
Czas utwardzania światłem	20 s	10 s

   - Lampa halogenowa długość fali: 400-500 nm, intensywność światła: ≥ 500 mW/cm^2
   - Lampa LED długość fali: 440-490 nm, intensywność światła: ≥ 1 000 mW/cm^2
   - Należy stosować lampy polimeryzacyjne, których specyfikacja jest zgodna z powyższą specyfikacją. Jeśli stosuje się inny rodzaj lampy, należy stosować się do danych dotyczących danej lampy polimeryzacyjnej podczas utwardzania BEAUTIFIL II.
   - Należy stosować okulary ochronne lub osłonę, aby uniknąć patrzenia bezpośrednio na utwardzaną światłem powierzchnię. Operatorzy muszą również chronić oczy pacjentów przed bezpośrednim narażeniem na światło polimeryzacyjne.
   - Należy zdezynfekować strzykawkę alkoholem po każdym użyciu w celu uniknięcia zakażeń krzyżowych. Następnie szczelnie zamknąć zakrętkę. Zachować ostrożność, aby uniknąć przedostania się alkoholu do materiału.
   - Należy zdezynfekować lampę polimeryzacyjną alkoholem po każdym użyciu w celu uniknięcia zakażeń krzyżowych.
9. Wykończenie i polerowanie
Po polimeryzacji wypełnienie należy opracować, wykończyć i wypolerować w tradycyjny sposób.

UWAGI DOTYCZĄCE UŻYTKOWANIA
1. Należy przestrzegać instrukcji każdego materiału i narzędzi używanych w połączeniu z tym produktem.
2. Nie należy używać tego produktu do celów innych niż w zaleceniach zawartych w punkcie WSKAZANIA w niniejszej instrukcji obsługi.
3. Stosować produkt zgodnie z datą ważności podaną na opakowaniu i pojemniku.
(Przykład ⌛ RRRR-MM-DD → Rok-Miesiąc-Dzień terminu ważności)

PRZECHOWYWANIE
Należy przechowywać w temperaturze pokojowej (1-30°C). Unikać dużej wilgotności i wysokiej temperatury. Trzymać z dala od bezpośredniego nasłonecznienia i źródeł zapłonu.

RO A SE CITI CU ATENŢIE ÎNAINTE DE UTILIZARE

Material de restaurare cu degajare de fluor

BEAUTIFIL II este un material de restaurare radioopac fotopolimerizabil pentru obturaţii anterioare şi posterioare.

CULORI
Seringă: A1, A2, A3, A3.5, A4, B1, B2, B3, C2, C3, A2O, A3O, Inc(Incisal), BW(Bleaching White)
Vârf: A1, A2, A3, A3.5, A4, B1, B2, C2, A2O, A3O, Inc, BW

COMPOZIŢIE
Material de restaurare Bis-GMA, TEGDMA, S-PRG pe bază de sticlă de fluoroboroaluminosilicat, promotor de polimerizare, pigmenţi şi altele

INDICAŢII
- Obturaţii anterioare şi posterioare
- Faţete laminate directe
- Repararea obturaţiilor şi a protezelor
- Acumularea miezului

PRECAUŢII
1. Nu utilizaţi acest produs la pacienţii cu alergii cunoscute la acest material şi/sau monomer de metacrilat.
2. Produsul nu trebuie utilizat de către personalul cabinetului stomatologic cu alergii cunoscute la acest material şi/sau monomer de metacrilat.
3. În cazul inflamaţiei sau apariţiei oricăror reacţii alergice la pacient sau la personalul cabinetului stomatologic, se va opri imediat utilizarea şi se va consulta medicul.
4. Nu reutilizaţi vârfurile.
5. Pentru a evita sensibilizarea la acest produs, utilizaţi mănuşi medicale (stomatologice) şi ochelari de protecţie. În cazul contactului accidental cu ţesuturi moi orale sau cu pielea, ştergeţi imediat cu tampoane de bumbac îmbibate în alcool şi clătiţi cu apă din abundenţă. Mănuşile medicale (stomatologice) oferă un grad de protecţie contra contactului direct cu acest material; cu toate acestea, unii monomeri pot pătrunde foarte rapid prin mănuşi, ajungând la piele. Prin urmare, dacă acest material intră în contact chiar şi numai cu mănuşile, schimbaţi-le imediat şi spălaţi mâinile cu apă din abundenţă.
6. În cazul contactului cu ochii, spălaţi imediat ochii cu apă din abundenţă şi consultaţi un medic.
7. Acest produs este destinat exclusiv utilizării de către specialiştii în medicină şi tehnică dentară.

INSTRUCŢIUNI DE UTILIZARE
1. Curăţarea suprafeţei dintelui
Curăţaţi temeinic suprafaţa dintelui pentru a îndepărta placa.
2. Selecţia culorii
Selectaţi o cheie adecvată cu ajutorul ghidului aferent cât dintele este încă umed.
3. Pregătirea cavităţii
Pregătiţi cavitatea pe baza metodei convenţionale.
4. Izolaţia
Izolaţi cu o digă de cauciuc.
5. Protecţia pulpei
În cazul în care pulpa este expusă sau zona afectată se află în apropierea pulpei, protejaţi pulpa cu hidroxid de calciu etc.
   - Nu utilizaţi materiale ce conţin eugenol deoarece acestea pot inhiba polimerizarea sau adeziunea materialului.
6. Condiţionarea şi aplicarea adezivului
Aplicaţi adeziv pentru dentină urmând instrucţiunile de utilizare furnizate de producător.
7. Amplasarea
Dozaţi o cantitate adecvată de BEAUTIFIL II din seringă pe un bloc de hârtie. Folosind un instrument corespunzător, aplicaţi materialul dozat în cavitate şi conferiţi-i forma dorită.
Dacă utilizaţi un vârf, introduceţi-l în dozator pentru a aplica materialul direct în cavitate. Materialul din vârf poate fi de asemenea dozat pe un bloc de hârtie şi aplicat în cavitate cu un instrument corespunzător.
   - În cazul depozitării acestor materiale la frigider, lăsaţi-le să atingă temperatura camerei înainte de utilizare.
   - Când aplicaţi materialul direct în cavitate, sterilizaţi dozatorul înainte şi după utilizare.
   - Vârfurile trebuie utilizate numai la un singur pacient şi trebuie apoi aruncate la finalul procedurii pentru a se evita contaminarea între pacienţi.
   - Apucaţi dozatorul delicat pentru a doza materialul lent. Presiunea excesivă poate determina ruperea vârfurilor sau ieşirea din dozator.
   - Pasta dozată pe blocul de hârtie trebuie să fie protejată de lumina ambiantă prin acoperirea cu scut de protecţie pentru a evita polimerizarea neprevăzută a materialului.
   - Strângeţi ferm capacul după fiecare utilizare.
8. Fotopolimerizarea
Fotopolimerizaţi folosind o unitate lampa de fotopolimerizare stomatologică.
   - În cazul în care cavitatea este adâncă, se recomandă umplerea şi fotopolimerizarea treptată, fără a depăşi 2 mm.
   - Când suprafaţa cavităţii este mare, fotopolimerizaţi pe segmente.
   - Lampa de fotopolimerizare stomatologică şi timpul de fotopolimerizare

Lampa de fotopolimerizare stomatologică	Halogen	LED
Durata fotopolimerizării	20 s	10 s

   - Lampa de fotopolimerizare cu halogen lungime de undă: 400-500 nm, intensitate luminoasă: ≥ 500 mW/cm^2
   - Unitate de fotopolimerizare cu led lungime de undă: 440-490 nm, intensitate luminoasă: ≥ 1000 mW/cm^2
   - Utilizaţi o lampa de fotopolimerizare cu specificaţii echivalente celor descrise mai sus. Când se utilizează o lampă de fotopolimerizare cu performanţe diferite de cele recomandate aici, urmaţi instrucţiunile aferente fiecărei lămpi pentru fotopolimerizarea adecvată a materialului BEAUTIFIL II.
   - Personalul trebuie să poarte ochelari de protecţie sau un scut de protecţie pentru a nu privi direct în lumina de fotopolimerizare. Personalul din cabinetul stomatologic trebuie să protejeze şi ochii pacienţilor împotriva expunerii directe la lumina de fotopolimerizare.
   - Dezinfectaţi seringa cu alcool după fiecare utilizare pentru a evita contaminarea între pacienţi. Apoi închideţi bine capacul. Procedaţi cu atenţie pentru a evita pătrunderea alcoolului în pastă.
   - Dezinfectaţi lampa de fotopolimerizare cu alcool după fiecare utilizare pentru a evita contaminarea între pacienţi.
9. Finisare şi lustruire
După fotopolimerizare, conturaţi, finisaţi şi lustruiţi preparaţia pe baza metodei convenţionale.

OBSERVAŢII PRIVIND UTILIZAREA
1. Urmaţi instrucţiunile de utilizare ale fiecărui material sau instrument de stomatologie care se utilizează în combinaţie cu acest produs.
2. Evitaţi utilizarea produsului în oricare alte scopuri decât cele specificate în INDICAŢIILE din cadrul acestor instrucţiuni de utilizare.
3. Utilizaţi produsul înainte de data expirării indicată pe ambalaj şi recipient.
(Exemplu ⌛ AAAA-LL-ZZ → Anul-Luna-Ziua a termenului de valabilitate)

DEPOZITAREA
Depozitaţi la temperatura camerei (1-30 °C). Evitaţi temperaturile înalte şi umiditatea ridicată. Nu expuneţi la lumina solară directă şi la nicio sursă de aprindere.

SV LÄS NOGA FÖRE ANVÄNDNING

Fluoravgivande kompositmaterial

BEAUTIFIL II är ett ljuspolymeriserande röntgenkontrasterande kompositmaterial för anteriora och posteriora fyllningar.

FÄRGER
Spruta: A1, A2, A3, A3.5, A4, B1, B2, B3, C2, C3, A2O, A3O, Inc (incisal), BW (vitt för blekning)
Ampull: A1, A2, A3, A3.5, A4, B1, B2, C2, A2O, A3O, Inc, BW

SAMMANSÄTTNING
Bis-GMA, TEGDMA, S-PRG filler baserad på fluoroboroaluminiumsilikat glas, polymeriseringsaktivator och andra

INDIKATIONER
- Anteriora och posteriora restaurationer
- Direkt laminatfernissa
- Reparation av restaurationer och protester
- Uppbyggnad av kärna

FÖRSIKTIGHETSÅTGÄRDER
1. Denna produkt får inte användas på patienter med kända allergier mot detta material och/eller metakrylat monomer.
2. Operatörer med kända allergier mot detta material och/eller metakrylat monomer ska inte använda denna produkt.
3. Om någon inflammation eller annan allergisk reaktion uppstår på antingen patient eller operatör ska användningen av produkten avbrytas omedelbart och medicinsk expertis konsulteras.
4. Ampullerna är enbart avsedda för engångsbruk och skall ej återanvändas.
5. Använd medicinska (dental-) handskar och skyddsglasögon för att undvika överkänslighet mot denna produkt. I händelse av kontakt med oral mjukvävnad eller hud, läska omedelbart med en bomullspellet fuktad med alkohol, och skölj med rikligt med vatten. Medicinska (dental-) handskar ger ett visst skydd mot direkt kontakt med detta material; vissa monomer kan dock penetrera mycket snabbt genom handskarna och nå huden. Skulle detta material ens komma i kontakt med handskarna så ska de omedelbart tas av och händerna ska sköljas med rikligt med vatten.
6. I händelse av kontakt med ögonen, skölj omedelbart med rikligt med vatten och uppsök medicinsk expertis.
7. Denna produkt är enbart avsedd för bruk av auktoriserad tandvårdspersonal.

BRUKSANVISNING
1. Rengöring av tandytan
Rengör tandytan noggrant för avlägsnande av plack.
2. Val av nyans
Välj lämplig färg med hjälp av en färgskala medan tanden fortfarande är fuktig.
3. Preparationer för kavitet
Preparera kaviteten på konventionellt sätt.
4. Isolering
Isolera med gummidamm.
5. Skydd av pulpa
Vid pulpaexponering eller om den angripna ytan är nära pulpan ska pulpan skyddas med kalciumhydroxid etc.
   - Använd inte material som innehåller eugenol då de kan inhibera polymeriseringen eller bindningen av dessa material.
6. Konditionering och applicering av adhesiv
Applicera dentin adhesiv i enlighet med respektive tillverkares bruksanvisningar.
7. Placering
Dosera lämplig mängd BEAUTIFIL II kompositmaterial på ett pappersblock från sprutan. Applicera den doserade mängden material i kaviteten med ett lämpligt instrument och skapa den önskade formen.
Vid användning av ampuller för kompositmaterialet ska ampullen appliceras i ampullpistolen för ändamålet så att materialet kan appliceras direkt i kaviteten. Kompositmaterial från ampullerna kan också doseras på ett pappersblock för att kunna appliceras i kaviteten med ett lämpligt instrument.
   - Vid förvaring av dessa material i kylskåp ska de rumstempereras före användning.
   - Vid applicering av kompositmaterialet direkt i kaviteten ska kompositsprutan steriliseras före och efter användningen.
   - Kanyler ska enbart användas på en patient och sedan kasseras efter den färdiga användningsproceduren för att undvika korskontaminering.
   - Greppa dispensern försiktigt för att fördela materialet långsamt. Överdrivet tryck kan göra att kanylerna bryts eller utmatning från dispensern.
   - Kompositmaterial som är upplagt på ett pappersblock ska skyddas från omgivande ljus med ett ljusskyddslock för att förhindra en för tidig och oavsiktlig polymerisering av materialet.
   - Förslut hättan noggrant efter varje användning.
8. Ljuspolymerisering
Ljuspolymerisera med en ljuspolymeriseringslampa för dentalt bruk.
   - Vid djupa kaviteter rekommenderas fyllningar i skikt och ljuspolymerisering som inte överskrider 2 mm.
   - Vid stora kavitetsytor, ljuspolymerisera i skikt.
   - Dental ljuspolymeriseringslampa och ljuspolymeriseringstid

Dental ljuspolymeriseringslampa	Halogen	LED
Ljuspolymeriseringstid	20 sek.	10 sek.

   - Halogen ljuspolymeriseringslampa våglängd: 400–500 nm, ljusintensitet: ≥ 500 mW/cm^2
   - LED ljuspolymeriseringslampa våglängd: 440–490 nm, ljusintensitet: ≥ 1000 mW/cm^2
   - Använd en ljuspolymeringslampa för dentalt bruk som har specifikationer som överensstämmer med de ovan beskrivna. Vid användning av ljuspolymeriseringslampor med andra specifikationer än de ovan angivna, ska instruktionerna för ljuspolymerisering av BEAUTIFIL II följas noggrant.
   - Tandvårdspersonalen måste använda skyddsglasögon eller ljusspatel för att undvika att se direkt på ljuspolymeriseringsljuset. Tandvårdspersonalen måste också skydda patientens ögon från direkt exponering för ljuset från polymeriseringslampan.
   - Desinficera kanylen med alkohol efter varje användning för att undvika korskontaminering. Dra sedan åt hättan noggrant. Iakttag försiktighet för att undvika att alkohol blandas i pastan.
   - Desinficera ljuspolymeriseringslampan med alkohol efter varje användning för att undvika korskontaminering.
9. Finishering och polering
Efter ljuspolymerisering, konturera, finishera och polera materialet på konventionellt sätt.

ATT NOTERA VID ANVÄNDNING
1. Följ bruksanvisningen för varje dental material eller instrument som ska användas i samband med denna produkt.
2. Denna produkt får inte användas för något annat ändamål än vad som specificeras i INDIKATIONER i denna bruksanvisning.
3. Använd denna produkt inom det utgångsdatum som specificeras på förpackningen och behållaren.
(T.ex. ⌛ ÅÅÅÅ-MM-DD → år-månad-dag för utgångsdatumet)

FÖRVARING
Förvaras i rumstemperatur (1–30 °C). Undvik höga temperaturer och hög luftfuktighet. Skyddas från direkt solljus och all form av gnistbildning.

EL ΔΙΑΒΑΣΤΕ ΠΡΟΣΕΚΤΙΚΑ ΠΡΙΝ ΑΠΟ ΤΗ ΧΡΗΣΗ

Υλικό αποκατάστασης με απελευθέρωση φθορίου

Το BEAUTIFIL II είναι ένα φωτοπολυμεριζόμενο ακτινοσκιερό υλικό αποκατάστασης που ενδείκνυται για αποκαταστάσεις πρόσθιων-οπισθίων δοντιών.

ΑΠΟΧΡΩΣΕΙΣ
Σύριγγα: A1, A2, A3, A3.5, A4, B1, B2, B3, C2, C3, A2O, A3O, Inc(Κοπτήρων), BW(Αστραφτερό λευκό)
Ρύγχος: A1, A2, A3, A3.5, A4, B1, B2, C2, A2O, A3O, Inc, BW

ΣΥΝΘΕΣΗ
Bis-GMA, TEGDMA, S-PRG ενισχυτικά σωματίδια με βάση φθορίο-βοριο-αργυλοπυριτικής ύάλου, ενεργοποιητής πολυμερισμού, χρωστικές ουσίες και άλλα

ΕΝΔΕΙΞΕΙΣ
- Αποκαταστάσεις πρόσθιων και οπισθίων δοντιών
- Άμεσες όψεις
- Επιδιόρθωση αποκαταστάσεων και προσθετικών εργασιών
- Ανασύσταση κολοβώματος

ΠΡΟΦΥΛΑΞΕΙΣ
1. Μη χρησιμοποιείτε αυτό το προϊόν σε ασθενείς με γνωστή αλλεργία στο υλικό αυτό και/ή σε μεθακρυλικό μονομερές.
2. Οι χειριστές που γνωρίζουν πως έχουν αλλεργία στο υλικό αυτό και/ή στο μεθακρυλικό μονομερές να μη χρησιμοποιούν το προϊόν αυτό.
3. Εάν εμφανιστεί φλεγμονή ή άλλη αλλεργική αντίδραση είτε στον ασθενή είτε στον χειριστή, θα πρέπει αμέσως να διακοπεί η χρήση του προϊόντος και να ζητηθεί ιατρική συμβουλή.
4. Μη ξαναχρησιμοποιείτε τα ρύγχη.
5. Χρησιμοποιήστε ιατρικά (οδοντιατρικά) γάντια και προστατευτικά γυαλιά για να αποφύγετε την ευαισθητοποίηση σε αυτό το προϊόν. Εάν κατά λάθος έρθει το προϊόν σε επαφή με τον μαλακό ιστό του στόματος ή του δέρματος, σκουπίστε αμέσως με βαμβάκι εμποτισμένο σε οινόπνευμα και ξεπλύνετε με άφθονο νερό. Τα ιατρικά (οδοντιατρικά) γάντια παρέχουν κάποια προστασία ενάντια στην απευθείας επαφή με αυτό το υλικό εντούτοις μερικά μονομερή μπορούν να διαπεράσουν μέσω των γαντιών και πολύ γρήγορα να φθάσουν στο δέρμα. Επομένως, εάν αυτό το υλικό έρθει σε επαφή ακόμη και με τα γάντια, πρέπει να τα αφαιρέσετε αμέσως και να πλύνετε τα χέρια σας με άφθονο νερό.
6. Σε περίπτωση επαφής με τα μάτια, ρίξτε άφθονο νερό και αναζητήστε ιατρική συμβουλή.
7. Αυτό το προϊόν προορίζεται για χρήση από επαγγελματίες της οδοντιατρικής μόνο.

ΟΔΗΓΙΕΣ ΧΡΗΣΗΣ
1. Καθαρισμός της επιφάνειας των δοντιών
Καθαρίστε λεπτομερώς την επιφάνεια των δοντιών για να αφαιρέσετε την πλάκα.
2. Επιλογή απόχρωσης
Επιλέξτε την κατάλληλη απόχρωση χρησιμοποιώντας το χρωματολόγιο ενώ το δόντι είναι ακόμα υγρό.
3. Προετοιμασία κοιλότητας
Προετοιμάστε την κοιλότητα ακολουθώντας τη συμβατική μέθοδο.
4. Απομόνωση
Απομονώστε με ελαστικό απομονωτήρα.
5. Προστασία πολφού
Στην περίπτωση που εκτίθεται ο πολφός ή η πλησιέστερη περιοχή είναι κοντά στον πολφό, καλύψτε τον πολφό με υδροξείδιο ασβεστίου κ.λπ.
   - Μη χρησιμοποιήστε υλικά που περιέχουν ευγενόλη δεδομένου ότι μπορεί να εμποδίσουν τον πολυμερισμό ή την προσκόλληση του υλικού.
6. Προπαρασκευή και εφαρμογή του συγκολλητικού παράγοντα
Εφαρμόστε τον συγκολλητικό παράγοντα οδοντίνης ακολουθώντας τις οδηγίες του κατασκευαστή για τη χρήση.
7. Τοποθέτηση
Βάλτε αρκετή ποσότητα BEAUTIFIL II πάνω σε χάρτινο μπλοκ από τη σύριγγα. Τοποθετείστε το υλικό στην κοιλότητα χρησιμοποιώντας κατάλληλο εργαλείο και δημιουργήστε το σχήμα που επιθυμείτε.
Εάν χρησιμοποιείτε ρύγχος, εισάγετε το ρύγχος στον διανομέα για να εφαρμόσετε το υλικό απευθείας στην κοιλότητα. Μπορείτε να βάλετε το υλικό και σε χάρτινο μπλοκ για να το εφαρμόσετε στην κοιλότητα με κατάλληλο εργαλείο.
   - Όταν αποθηκεύετε αυτά τα υλικά σε ψυγείο, αφήστε τα να φθάσουν σε θερμοκρασία δωματίου πριν από τη χρήση.
   - Όταν εφαρμόζετε το υλικό απευθείας στην κοιλότητα, αποστειρώστε τον διανομέα πριν και μετά τη χρήση.
   - Το ρύγχος πρέπει να χρησιμοποιηθεί μόνο για έναν ασθενή και να απορριφθεί μετά το τέλος της διαδικασίας για να αποφευχθεί η επιμόλυνση.
   - Σφίξτε το πιστόλι ελαφρά για να εξαχθεί το υλικό αργά. Υπερβολική πίεση μπορεί να προκαλέσει θραύση της φύσιγγας ή εκτόπιση της από το πιστόλι.
   - Η πάστα που εξάγεται στο χάρτινο μπλοκ πρέπει να προστατεύεται από το φως του περιβάλλοντος με προστατευτικό κάλυμμα ώστε να αποφευχθεί ο πολυμερισμός του υλικού.
   - Κλείστε σφικτά το καπάκι μετά από κάθε χρήση.
8. Φωτοπολυμερισμός
Φωτοπολυμερίστε με οδοντιατρική συσκευή φωτοπολυμερισμού.
   - Όταν η κοιλότητα είναι βαθειά συνιστάται διαστρωματική τοποθέτηση και φωτοπολυμερισμός με πάχος στρώματος που δεν υπερβαίνει τα 2 mm.
   - Όταν η επιφάνεια της κοιλότητας είναι μεγάλη τότε ο φωτοπολυμερισμός γίνεται σε τμήματα.
   - Οδοντιατρική μονάδα φωτοπολυμερισμού και χρόνος φωτοπολυμερισμού

Οδοντιατρική μονάδα φωτοπολυμερισμού	Αλογόνου	LED
Χρόνος φωτοπολυμερισμού	20 δευτ.	10 δευτ.

   - Μονάδα φωτοπολυμερισμού με αλογόνο Μήκος κύματος: 400-500 nm, Ένταση φωτός: ≥ 500 mW/cm^2
   - Μονάδα φωτοπολυμερισμού LED Μήκος κύματος: 440-490 nm, Ένταση φωτός: ≥ 1000 mW/cm^2
   - Χρησιμοποιήστε μια οδοντική μονάδα φωτοπολυμερισμού που έχει προδιαγραφές αντίστοιχες με αυτές που περιγράφονται ανωτέρω. Εάν χρησιμοποιείτε κάποια που έχει απόδοση άλλη από αυτές τις συστάσεις, ακολουθήστε κατάλληλα τις οδηγίες κάθε μονάδας για τον φωτοπολυμερισμό του BEAUTIFIL II.
   - Οι χειριστές πρέπει να χρησιμοποιήσουν τα προστατευτικά γυαλιά ή την πλακέτα προστασίας από το φως για να αποφύγουν να κοιτάξουν απευθείας στο φως του φωτοπολυμερισμού. Οι χειριστές πρέπει να προστατεύσουν επίσης τα μάτια των ασθενών από απευθείας έκθεση στο φως του φωτοπολυμερισμού.
   - Απολυμάνετε τη σύριγγα με αλκοόλη μετά από κάθε χρήση για την αποφυγή επιμόλυνσης. Έπειτα, κλείστε καλά το καπάκι. Προσέχετε για την αποφυγή συμπερίληψης αλκοόλης στην πάστα.
   - Απολυμάνετε τη μονάδα φωτοπολυμερισμού με αλκοόλη μετά από κάθε χρήση για την αποφυγή επιμόλυνσης.
9. Ολοκλήρωση και γυάλισμα
Μετά τον φωτοπολυμερισμό, ακολουθήστε τη συμβατική μέθοδο για διαμόρφωση, ολοκλήρωση και γυάλισμα της αποκατάστασης.

ΣΗΜΕΙΩΣΗ ΓΙΑ ΤΗ ΧΡΗΣΗ
1. Ακολουθήστε τις οδηγίες για τη χρήση κάθε οδοντικού υλικού ή οργάνου που χρησιμοποιείται από κοινού με αυτό το προϊόν.
2. Μη χρησιμοποιήστε αυτό το προϊόν για οποιουσδήποτε άλλους λόγους εκτός από αυτούς που συγκεκριμένα περιγράφονται στις ΕΝΔΕΙΞΕΙΣ σε αυτές τις οδηγίες για τη χρήση.
3. Χρησιμοποιήστε αυτό το προϊόν εντός της ημερομηνίας λήξης που υποδεικνύεται στη συσκευασία και το κιβώτιο.
(Παράδειγμα ⌛ ΕΕΕΕ-ΜΜ-ΗΗ → Έτος-Μήνας-Ημέρα της ημερομηνίας λήξης)

ΑΠΟΘΗΚΕΥΣΗ
Αποθηκεύστε σε θερμοκρασία δωματίου (1-30 °C). Αποφύγετε τις υψηλές
θερμοκρασίες και την υψηλή υγρασία. Κρατήστε μακρυά από το άμεσο φως του ήλιου και οποιαδήποτε πηγή ανάφλεξης.
