# Supplementary material for: Effect of different scaling instruments on the roughness of esthetic resin composite restorations: an in vitro study
Source: Front Dent Med. 2026 Aug 28;7:1894177. doi: 10.3389/fdmed.2026.1894177 (PMC13561887; doi:10.3389/fdmed.2026.1894177)
Supplement: Supplementary file 1 [file Datasheet1.zip › IPSEmpressDirectIFU.pdf]

English

Description
IPS Empress® Direct is a light-curing, radiopaque, nano-hybrid composite based on state-of-the-art technology, which is suitable for the fabrication of restorations that fulfill the highest demands. IPS Empress Direct cures with light in the wavelength range of 400-500 nm (blue light).

Shades
IPS Empress Direct is offered in 32 different shades and 5 different degrees of translucency. The following shades are available:
Enamel shades
A1, A2, A3, A3.5, A4, B1, B2, B3, B4, C1, C2, C3, D2, D3, Bleach-L, Bleach-XL
Dentin shades
A1, A2, A3, A3.5, A4, IVA5, IVA6, B1, B2, C3, D2, Bleach-L, Bleach-XL
Translucent shades
Trans 20, Trans 30, Opal*

*Not radiopaque due to its high transparency

Composition
The monomer matrix is composed of dimethacrylates (20-21.5 wt%, opalescent shade 17 wt%). The fillers contain barium glass, ytterbium trifluoride, mixed oxide, silicon dioxide and copolymer (77.5-79 wt%, opalescent shade 83 wt%). Additional contents: additives, catalysts, stabilizers and pigments (<1.0 wt%). The total content of inorganic fillers is 75-79 wt% or 52-59 vol% (opalescent shade 60.5 wt% or 45 vol%). The particle size of the inorganic fillers is between 40 nm and 3000 nm with a mean particle size of 550 nm.

Indication
– Anterior restorations (Class III, IV)
– Restorations in the posterior region (Classes I and II)
– Tooth neck restorations (Class V, e.g. in the case of cervical caries, root erosion, wedge-shaped defects)
– Corrections of tooth positions and tooth shape (e.g. diastema closure, closure of interdental "black triangles", lengthening of the incisal edge)
– Direct veneers

Contraindication
Placement of IPS Empress Direct restorations is contraindicated
– if a dry working field cannot be established, or if the stipulated technique cannot be applied
– if a patient is known to be allergic to any of the ingredients in IPS Empress Direct

Side effects
In rare cases, components of IPS Empress Direct may lead to sensitization. IPS Empress Direct should not be used in such cases. To avoid possible irritation of the pulp, areas close to the pulp should be protected with a suitable pulp/dentin protector (selectively apply a calcium hydroxide-based preparation in areas close to the pulp and coat with a suitable liner).

Interactions
Substances containing eugenol/oil of cloves may inhibit the polymerization of composite materials. Consequently, the application of such materials together with IPS Empress Direct must be avoided. Discoloration may occur in combination with cationic mouthwashes, plaque disclosing agents and chlorhexidine.

IPS Empress® Direct

EN Instructions for Use
– Light-curing resin-based dental restorative material

DE Gebrauchsinformation
– Lichthärtendes zahnärztliches Füllungscomposite

FR Mode d'emploi
– Composite de restauration photopolymérisable

IT Istruzioni d'uso
– Materiale dentale da otturazione fotoindurente

ES Instrucciones de uso
– Material de restauración dental en base a resina fotopolimerizable

PT Instruções de Uso
– Material de restauração, baseado em resina e fotopolimerizável

DA Brugsanvisning
– Lyshærdende dentalt plastfyldningsmateriale

FI Käyttöohjeet
– Valokovetteinen resiinipohjainen täytemateriaali hammaslääketieteelliseen käyttöön

NO Bruksanvisning
– Lysherdende odontologisk fyllingsmateriale

NL Gebruiksaanwijzing
– Lichtuithardend composietvulmateriaal voor tandheelkundig gebruik

EL Οδηγίες Χρήσεως
– Οδοντιατρικό αποκαταστατικό υλικό, φωτοπολυμεριζόμενο, με ακρυλική βάση

IPS Empress® Direct

SL Navodilo za uporabo
– Kompozit za plombiranje, ki se strjuje s halogensko lučjo

HR Upute za upotrebu
– Kompozit za ispune, otvrdnjava halogenim svjetlom

CS Návod k použití
– Světlem vytvrzovaný zubařský výplňový kompozit

SK Návod na používanie
– Svetlom vytvrdzovaný zubný rekonštrukčný materiál na báze živice

HU Használati utasítás
– Fényre keményedő fogorvosi tömőkompozitum

PL Instrukcja stosowania
– Światłoutwardzalny materiał

BG Инструкции за употреба
– Фотополимеризиращ зъболекарски композит за пълнежи

SQ Udhëzime përdorimi
– Material dentar rrëshinor restaurues fotopolimerizues

RO Instrucţiuni de utilizare
– Material compozit dentar restaurativ şi fotopolimerizabil

RU Инструкция
– Стоматологический композитный пломбировочный материал световой полимеризации

UK Інструкція з використання
– Рентгеноконтрастний композит світлового отвердження для пломбування зубів

Application

1. Shade determination

Clean the teeth prior to shade determination. The enamel shade is selected with the tooth still moist. The dentin shade can be taken either by placing the tab next to the prepared tooth or next to the cervical area of the canine.

2. Isolation

Appropriate isolation is required, best with a rubber dam (e.g. OptraDam® Plus).

3. Cavity preparation

Cavity preparation is carried out according to the requirements of the adhesive technique, i.e. protecting the tooth structure. Enamel edges in the anterior region are bevelled. In the posterior region, only sharp enamel edges should be rounded (finishing diamonds, 25-40 µm). Caries-free cervical defects are not prepared, only cleaned with pumice or other suitable cleaning pastes with the help of rubber cups or rotary brushes. In case of sclerotic dentin, superficial reconditioning with a rose bur can improve the adhesive bond. Subsequently, all residue in the cavity is removed with water spray. The cavity is dried with water- and oil-free air.

4. Pulp protection / Base

Do not apply a base material when using an enamel/dentin bonding agent. Only cover very deep areas close to the pulp with a calcium hydroxide material (e.g. ApexCal®) and subsequently use pressure-resistant cement (e.g. glass ionomer cement, such as Vivaglass® Liner). Do not cover other cavity walls, since they can be used to support the bond with an enamel/dentin adhesive.

5. Placement of a matrix / interdental wedge

Use a wrap-around matrix for cavities affecting the promixal area or a sectional matrix and wedge.

6. Conditioning / Application of the bonding agent

Condition the tooth structure and apply the bonding agent according to the Instructions for Use of the product in use. We recommend using Syntac® or ExciTE® F (with phosphoric acid etching) or AdheSE® or AdheSE® One F self-etching adhesive.

7. Application of IPS Empress Direct

- Since a large number of IPS Empress Direct Dentin and Enamel shades in different translucencies for the creation of anatomical detail are available, any layering technique can be creatively used to achieve highly esthetic restorations. For an optimum result, apply IPS Empress Direct in layers of max. 2 mm (or 1.5 mm Dentin shades) thickness and adapt with e.g. OptraSculpt®/ OptraSculpt® Pad.
- Sufficient exposure to the curing light prevents incomplete polymerization. Recommended curing times (exposure time) and light intensities are listed in Table 1. Hold the light emission window as closely as possible to the surface of the restorative material.
- When using a metal matrix, additionally polymerize the restoration from the buccal or lingual/palatal aspect after removing the matrix, if no Bluephase® curing light was used or if the light probe could not be optimally positioned (e.g. far away from the material or with divergent radiation).
- In many cases, a flowable composite is now used for the initial layer in order to create an even cavity floor and to simplify the adaptation of the subsequently used restorative material. The use of a flowable composite (e.g. Tetric EvoFlow®) as an initial thin layer is optional. This layer must be light-cured in a separate step (the instructions of the respective Instructions for Use must be observed).

8. Finishing / Checking the occlusion / Polishing

Remove excess material with suitable finishers (e.g. Astropol® F) or fine diamonds after polymerization. Remove proximal excess with diamond finishers, tungsten carbide finishers, finishing strips, or flexible finishing discs. Check the occlusion and articulation and apply appropriate grinding corrections to prevent premature contacts or undesired articulation paths on the surface of the restorations. Use silicone polishers (e.g. Astropol® P, Astropol® HP, Astrobrush or OptraPol® Next Generation) as well as polishing discs and polishing strips to polish the restorations to a durable high gloss.

Additional information

1. If necessary, additional IPS Empress Direct material can be directly applied to polymerized material. If the IPS Empress Direct restoration has already been polished, it must first be roughened and wetted with Heliobond® before fresh IPS Empress Direct is applied.
2. IPS Empress Direct should be at room temperature when it is applied. Cool temperatures render the material difficult to extrude.
3. For single use only. If IPS Empress Direct is applied from the Cavifil directly in the mouth of the patient, the Cavifil must be discarded after use to prevent cross-contamination between patients.
4. Syringes or Cavifils should not be disinfected with oxidizing disinfection agents.

Warning

Unpolymerized IPS Empress Direct should not come in contact with the skin, mucous membrane, or eyes. Unpolymerized IPS Empress Direct may have a slight irritating effect and may lead to a sensitization against methacrylates. Commercial medical gloves do not provide protection against sensitivity to methacrylates.

Storage

- Storage temperature 2–28 °C / 36–82 °F.
- Close syringes/Cavifils immediately after use. Exposure to light causes premature polymerization.
- Do not use IPS Empress Direct after the indicated date of expiration.
- Shelf life: see information on Cavifils, syringes and packages.

Keep material out of children's reach.
For use in dentistry only.

The material has been developed solely for use in dentistry. Processing should be carried out strictly according to the Instructions for Use. Liability cannot be accepted for damages resulting from failure to observe the Instructions or the stipulated area of application. The user is responsible for testing the products for their suitability and use for any purpose not explicitly stated in the Instructions. Descriptions and data constitute no warranty of attributes and are not binding.

Deutsch

Beschreibung

IPS Empress® Direct ist ein auf moderner Technologie basierendes lichthärtendes, röntgenopakes Nanohybrid-Composite für Restaurationen höchster Ansprüche. IPS Empress Direct härtet mit Licht der Wellenlänge im Bereich von 400-500 nm (Blaulicht) aus.

Farben
Wir bieten 32 Farben in 5 verschiedenen Transluzenzstufen.
IPS Empress Direct ist in folgenden Farben lieferbar:
Schmelzfarben
A1, A2, A3, A3.5, A4, B1, B2, B3, B4, C1, C2, C3, D2, D3, Bleach-L, Bleach-XL
Dentinfarben
A1, A2, A3, A3.5, A4, IVA5, IVA6, B1, B2, C3, D2, Bleach-L, Bleach-XL
Transluzente Farben
Trans 20, Trans 30, Opal*

*Aufgrund der hohen Transparenz nicht röntgenopak

Zusammensetzung
Die Monomermatrix besteht aus Dimethacrylaten (20 - 21.5 Gew-%, opaleszente Farbe 17 Gew-%). Die Füllstoffe bestehen aus Bariumglas, Ytterbiumtrifluorid, Mischoxid, Siliziumdioxid und Copolymer (77.5 - 79 Gew-%, opaleszente Farbe 83 Gew-%). Zusätzlich enthalten sind Additive, Katalysatoren, Stabilisatoren und Pigmente (< 1.0 Gew-%). Der Gesamtgehalt an anorganischem Füller beträgt 75 - 79 Gew-% bzw. 52 - 59 Vol.-% (opaleszente Farbe 60.5 Gew-% bzw. 45 Vol.-%). Die Primärpartikelgrösse der anorganischen Füllstoffe liegt zwischen 40 nm und 3000 nm bei einer mittleren Korngrösse von 550 nm.

Indikation
– Frontzahnfüllungen (Klasse III, IV)
– Seitenzahnfüllungen (Klasse I und II)
– Zahnhalsfüllungen (Klasse V, z.B. bei Zahnhalskaries, Wurzelerosionen, keilförmigen Defekten)
– Zahnstellungs- und Zahnform-Korrekturen (z.B. Diastemaschluss, Schluss von Interdentaldreiecken, Verlängerung der Inzisalkanten)
– Direkte Veneers

Kontraindikation
Das Legen von IPS Empress Direct Füllungen ist kontraindiziert
– wenn eine ausreichende Trockenlegung oder die vorgeschriebene Anwendungstechnik nicht möglich ist
– bei erwiesener Allergie gegen Bestandteile von IPS Empress Direct

Nebenwirkungen
Bestandteile von IPS Empress Direct können in seltenen Fällen zu einer Sensibilisierung führen. In diesen Fällen ist auf die weitere Verwendung zu verzichten. Um Irritationen der Pulpa auszuschliessen, sind pulpanahe Areale mit einem geeigneten Pulpa-/Dentinschutz zu versorgen (pulpanah punktförmig ein kalziumhydroxidhaltiges Präparat aufbringen und mit einer geeigneten Unterfüllung abdecken).

Wechselwirkungen
Eugenol- / nelkenölhaltige Werkstoffe inhibieren die Aushärtung von Composites. Auf die Verwendung solcher Materialien zusammen mit IPS Empress Direct ist zu verzichten. In Kontakt mit kationischen Mundwässern sowie bei Plaquerelevatoren und Chlorhexidin können Verfärbungen auftreten.

Anwendung
1. Farbbestimmung
Vor der Farbbestimmung die Zähne reinigen. Die Schmelzfarbe wird am noch feuchten Zahn bestimmt. Die Dentinfarbe kann am präparierten Zahn direkt oder zervikal am Eckzahn bestimmt werden.

SV Bruksanvisning
– Ljushärdande resinbaserat dentalt fyllnadsmaterial

TR Kullanma Talimatları
– Işıkla sertleşen dental restoratif kompozit

złożony przeznaczony do wypełniania ubytków

SR Упутство за употребу
– Композит за испуне који се стврдњава на светлу

MK Упатство за употреба
– Стоматолошки композит што се стврднува на светло за ресторативната терапија со полнења

ET Kasutamisjuhend
– Valguskõvastuv täidismaterjal hambaraviks

LV Lietošanas instrukcija
– Gaismā cietējošs zobārstniecības kompozīts plombām

LT Naudojimo informacija
– Kietėjanti šviesoje, taki plombuojamoji medžiaga odontologijai

CE 0123

Rx ONLY

Date information prepared
2013-06-25/Rev. 2
627408/WW

Manufacturer
Ivoclar Vivadent AG
9494 Schaan/Liechtenstein
www.ivoclarvivadent.com

2. Trockenlegung
Ausreichende Trockenlegung, am besten mit Kofferdam (z. B. OptraDam® Plus), ist erforderlich.

3. Kavitätenpräparation
Die Kavitätenpräparation erfolgt nach den Regeln der Adhäsivtechnik, d. h. unter Schonung der Zahnhartsubstanz. Im Frontzahnbereich die Schmelzränder anschrägen, im Bereich der Seitenzähne nur die scharfen Schmelzkanten leicht brechen oder abrunden (Finierdiamant, 25–40 µm). Kariesfreie Zahnhalsdefekte werden nicht präpariert sondern nur mit Bims bzw. einer geeigneten Reinigungspaste sowie einem Gummikelch oder einem rotierenden Bürstchen gesäubert. Bei sklerotischem Dentin kann ein oberflächliches Anfrischen mit einem Rosenbohrer den adhäsiven Verbund verbessern. Anschliessend Entfernung aller Rückstände in der Kavität mit Wasserspray und Trocknen der Kavität mit wasser- und ölfreier Luft.

4. Pulpenschutz / Unterfüllung
Bei Verwendung eines Schmelz-Dentin-Haftvermittlers soll auf eine Unterfüllung verzichtet werden. Nur bei sehr tiefen, pulpanahen Kavitäten diesen Bereich punktförmig mit einem Kalziumhydroxidpräparat (z. B. ApexCal®) abdecken und anschliessend mit einem druckstabilen Zement überschichten (z. B. Glasionomerzement wie Vivaglass® Liner). Die restlichen Kavitätenwände nicht abdecken, damit sie für die Haftvermittlung mit einem Schmelz-Dentin-Adhäsiv nutzbar bleiben.

5. Matrize / Interdentalkeil anbringen
Bei Kavitäten mit approximalem Anteil entweder eine Zirkulärmatrize oder eine Teilmatrize verwenden und verkeilen.

6. Konditionierung / Applikation des Haftvermittlers
Konditionieren und Applikation des Haftvermittlers entsprechend der Gebrauchsanleitung des verwendeten Produktes. Ivoclar Vivadent empfiehlt die Haftvermittler Syntac® oder ExciTE® F (in Verbindung mit Phosphorsäureätzung) oder die selbstätzenden Adhäsive AdheSE® bzw. AdheSE® One F zu verwenden.

7. Applikation von IPS Empress Direct
– Da bei IPS Empress Direct eine grosse Anzahl von Dentin- und Schmelzfarben in unterschiedlichen Transparenzen sowie transluzente Farben zur Gestaltung anatomischer Fein- und Besonderheiten zur Verfügung stehen, kann damit jede Schichttechnik kreativ eingesetzt werden, um eine hochästhetische Füllung zu legen. Mit z. B. OptraSculpt®/OptraSculpt® Pad kann die Füllung adaptiert werden. IPS Empress Direct darf in Schichtstärken von max. 2 mm bzw. 1,5 mm (Dentinfarben) appliziert werden.
– Eine ausreichende Belichtung verhindert eine unvollständige Polymerisation. Empfehlungen zur Belichtungszeit (Exposure Time) und Lichtintensität (Light Intensity) siehe Table 1. Das Lichtaustrittsfenster möglichst nahe an das Füllungsmaterial halten.
– Bei Anwendung einer Metallmatrize muss nach dem Entfernen die Füllung zusätzlich von bukkal und lingual/palatinal belichtet werden, wenn kein Bluephase®-Polymerisationsgerät eingesetzt wurde oder der Lichtleiter nicht ideal positioniert werden konnte, zum Beispiel bei Abstand zum Composite oder bei divergierendem Abstrahlwinkel.
– In vielen Fällen wird heutzutage als erste Schicht ein fliessfähiges Composite verwendet, um einen gleichmässigen Kavitätenboden zu schaffen und gleichzeitig die Adaptation des anschliessend verwendeten Füllungsmaterials zu erleichtern. Die Verwendung eines fliessfähigen Composites (z.B. Tetric EvoFlow®) in einer dünnen ersten Schicht ist fakultativ möglich. Diese Schicht muss separat ausgehärtet werden (die entsprechenden Vorgaben der jeweiligen Gebrauchsinformation sind zu beachten).

8. Ausarbeiten / Okklusionskontrolle / Politur
Nach der Polymerisation die Überschüsse mit geeigneten Finierern (z.B. Astropol® F) oder feinkörnigen Diamanten entfernen. Approximale Überschüsse mit Diamant-, Hartmetallfinierern oder mit Finierstreifen bzw. flexiblen Finierscheibchen entfernen. Okklusion und Artikulation überprüfen und einschleifen, so dass keine Frühkontakte oder unerwünschte Artikulationsbahnen auf der Füllungsoberfläche verbleiben. Die Hochglanzpolitur erfolgt mit Silikonpolierern (z.B. Astropol P, Astropol HP bzw. Astrobrush oder OptraPol® Next Generation) sowie Polierscheiben und Polierstreifen.

Besondere Hinweise
1. Bei Korrekturen kann frisches IPS Empress Direct Material direkt auf schon polymerisiertes Material aufgebracht werden. Ist die IPS Empress Direct Füllung schon poliert, muss sie zuerst aufgeraut und mit Heliobond benetzt werden, bevor neues IPS Empress Direct aufgebracht wird.
2. IPS Empress Direct soll bei Umgebungstemperatur verwendet werden. Bei Kühlschranktemperatur kann das Auspressen erschwert sein.
3. Nur zum Einmalgebrauch. Wird IPS Empress Direct aus dem Cavifil direkt im Mund des Patienten appliziert, so ist das Cavifil aus hygienischen Gründen nur für einen Patienten angezeigt (Vermeidung von Kreuzinfektionen zwischen Patienten).
4. Keine Desinfektion von Spritzen oder Cavifils mit oxidierenden Desinfektionsmitteln.

Warnhinweis
Kontakt von unausgehärtetem IPS Empress Direct mit Haut / Schleimhaut und Augen vermeiden. IPS Empress Direct kann in unausgehärtetem Zustand leicht reizend wirken und zu einer Sensibilisierung auf Methacrylate führen. Handelsübliche medizinische Handschuhe schützen nicht vor Sensibilisierung auf Methacrylate

Lager- und Aufbewahrungshinweise
– Lagertemperatur 2–28 °C.
– Spritzen/Cavifils nach Gebrauch sofort verschliessen. Lichtzutritt führt zu vorzeitiger Polymerisation.
– IPS Empress Direct nach Ablauf des Verfalldatums nicht mehr verwenden.
– Ablaufdatum: siehe Hinweis auf Cavifil, Spritze bzw. Verpackung.

Für Kinder unzugänglich aufbewahren!
Nur für zahnärztlichen Gebrauch!

Das Produkt wurde für den Einsatz im Dentalbereich entwickelt und muss gemäss Gebrauchsinformation angewendet werden. Für Schäden, die sich aus anderweitiger Verwendung oder nicht sachgemässer Anwendung ergeben, übernimmt der Hersteller keine Haftung. Darüber hinaus ist der Verwender verpflichtet, das Produkt eigenverantwortlich vor dessen Einsatz auf Eignung und Verwendungsmöglichkeit für die vorgesehenen Zwecke zu prüfen, zumal wenn diese Zwecke nicht in der Gebrauchsinformation aufgeführt sind.

Description
IPS Empress® Direct est un composite nano-hybride, photopolymérisable radio-opaque basé sur une technologie de pointe, approprié pour les restaurations répondant aux exigences les plus élevées. IPS Empress Direct se polymérise avec une lumière d'une longueur d'onde comprise entre 400 et 500 nm (partie bleue du spectre de la lumière).

Teintes
IPS Empress Direct offre 32 teintes différentes et 5 degrés de translucidité. IPS Empress Direct est disponible dans les teintes suivantes :
Teintes Email
A1, A2, A3, A3.5, A4, B1, B2, B3, B4, C1, C2, C3, D2, D3, Bleach-L, Bleach-XL
Teintes Dentine
A1, A2, A3, A3.5, A4, IVA5, IVA6, B1, B2, C3, D2, Bleach-L, Bleach-XL
Teintes translucides
Trans 20, Trans 30, Opal* *Non radio-opaque en raison de sa transparence élevée

Composition
La matrice monomère est composée de diméthacrylates (20 -21.5 % poids, teinte opalescente 17% poids). La charge minérale se compose de verre de baryum, du trifluoride d'ytterbium, d'oxyde mixte, dioxide de silicium et de copolymères (77.5-79 % poids, teinte opalescente 83 % poids). Sont aussi contenus : additifs, catalyseurs, stabilisateurs et pigments (<1.0 % poids). La teneur totale en charge minérale est de 75-79 % poids ou 52-59 % (teinte opalescente 60.5 % poids ou 45% volume). La taille des particules est comprise entre 40 nm et 3 000 nm, avec une taille moyenne de 550 nm.

Indications
– Restaurations antérieures (Classes III, IV)
– Restaurations postérieures (Classes I et II)
– Restaurations de la zone cervicale (Classe V, ex. en cas de caries cervicales, érosion cervicale, défauts cunéiformes)
– Corrections de la position et de la forme des dents (ex. fermeture de diastèmes, fermeture de "triangles noirs" interdentaires, allongement du bord incisif)
– Facettes en méthode directe

Contre-indications
L'utilisation d'IPS Empress Direct n'est pas indiquée :
– s'il n'est pas possible d'isoler le champ opératoire, ou de respecter les prescriptions du mode d'emploi,
– en cas d'allergie connue à l'un de ses composants.

Effets secondaires
Les composants d'IPS Empress Direct peuvent, dans certains cas, conduire à des sensibilités. Dans ces cas, ne plus utiliser le matériau.
Afin d'éviter une irritation pulpaire, il est conseillé d'utiliser une protection dentinaire/pulpaire sur les zones proches de la pulpe (application d'une préparation à l'hydroxyde de calcium et d'un fond de cavité adapté).

Interactions
Les substances à base d'eugénol/essence de girofle inhibent la polymérisation des composites. Par conséquent, l'application de ces matériaux en combinaison avec IPS Empress Direct doit être évitée.
Le contact avec des solutions cationiques de rinçage buccal, des révélateurs de plaque et la chlorhexidine peuvent conduire à des colorations.

Mise en oeuvre
1. Détermination de la teinte
Nettoyer les dents avant de procéder au choix de la teinte.
Celui-ci doit s'effectuer sur dent humide. La teinte est relevée en plaçant le nuancier à côté de la dent préparée ou près de la zone cervicale de la canine.
2. Isolation du champ opératoire
Il est conseillé de bien isoler le champ opératoire, de préférence à l'aide d'une digue. (OptraDam® Plus)
3. Préparation de la cavité
Préparer la cavité suivant les principes de la restauration adhésive, c'est-à-dire. de façon à préserver le plus possible la structure dure de la dent. Les bords de l'émail sont bisautés dans la région antérieure. Dans les zones postérieures, les arêtes vives de l'émail sont préparées avec un instrument diamanté à finir (25-40 µm). Les défauts cervicaux exempts de carie, sont nettoyés à la ponce ou avec une pâte de nettoyage adaptée, puis à l'aide d'un disque caoutchouté ou d'une brossette rotative. En cas de sclérose de la dentine, reconditionner de manière superficielle à l'aide d'une fraise ronde peut améliorer l'adhésion. Nettoyer ensuite la cavité au spray et la sécher à l'air exempt d'eau et d'huile.
4. Protection pulpaire / Fond de cavité
L'application d'un adhésif amélo-dentinaire permet de ne pas utiliser de fond de cavité. Appliquer une préparation à base d'hydroxyde de calcium (ex. ApexCal®) seulement dans les cas de cavités très profondes, proches de la pulpe, et recouvrir ensuite avec un ciment résistant à la compression (ex. un verre ionomère). Ne pas appliquer de ciment sur les autres parois de la cavité, afin de pouvoir y appliquer un adhésif amélo-dentinaire.
5. Matrice / Coin interdentaire
Pour les cavités ayant une face proximale, utiliser une matrice transparente (ex. OptraMatrix, à zone sélective plus fine) ou une matrice partielle et la fixer.
6. Conditionnement / Application de l'adhésif
Conditionner et appliquer l'adhésif selon le mode d'emploi du produit utilisé. Les matériaux Ivoclar Vivadent étant adaptés les uns aux autres, utiliser de préférence Syntac® ou ExciTE® F (après un mordançage préalable à l'acide phosphorique) ou l'adhésif automordançant (AdheSE® ou AdheSE® One F).
7. Application d'IPS Empress Direct
– Un grand nombre de teintes IPS Empress Direct sont disponibles selon différents degrés de translucidité. De fait, l'obtention de résultats hautement esthétiques s'en trouve considérablement simplifiée. Pour un résultat optimal, appliquer IPS Empress Direct en une épaisseur de couche maximale de 2 mm (ou 1,5 mm pour les teintes Dentine). Mettre en forme IPS Empress Direct à l'aide d'un instrument adapté comme, par exemple, Optra-Sculpt®/OptraSculpt® Pad.
– Une exposition insuffisante à la lampe à polymériser empêche une polymérisation totale. Pour les recommandations concernant le temps d'exposition (Exposure Time) et l'intensité lumineuse (Light Intensity), voir le tableau 1. Maintenir l'embout lumineux de la lampe le plus près possible du matériau d'obturation.
– Si vous avez utilisé une matrice métallique, il est recommandé de procé-

der à une photopolymérisation supplémentaire sur les faces vestibulaire et linguale/palatine après retrait de la matrice, si vous n'avez pas utilisé de lampe à photopolymériser Bluephase® ou si l'embout lumineux n'a pas pu être positionné de manière optimale (par exemple, loin du matériau ou avec une radiation divergente).
– Dans de nombreux cas on utilise maintenant un composite fluide en couche initiale afin de créer un fond de cavité régulier et pour simplifier l'adaptation du matériau de restauration qui sera utilisé ensuite. L'utilisation d'un composite fluide (ex. Tetric EvoFlow®) comme couche initiale est optionnelle. Cette couche doit être photopolymérisée séparément (respecter le mode d'emploi correspondant).

8. Finition / Contrôle de l'occlusion / Polissage
Après la photopolymérisation, éliminer les excédents de matériau à l'aide de pointes abrasives (ex. Astropol F) ou des instruments diamantés à fine granulométrie. Eliminer les excédents proximaux à l'aide d'instruments diamantés, de polissoirs au carbure de tungstène, de strips abrasifs ou de disques de finition flexibles. Contrôler l'occlusion et l'articulé. Il convient de veiller à la réalisation d'un bon profil occlusal, afin d'éviter un contact antagoniste prématuré pouvant provoquer des fractures. Le polissage au brillant s'obtient grâce aux pointes siliconées vertes Astropol® P, Astropol® HP, Astrobrush ou OptraPol® Next Generation ainsi qu'aux disques ou strips de polissage.

Remarques particulières
1. Un ajout d'IPS Empress Direct peut être appliqué, si nécessaire, sur du matériau déjà polymérisé. Si l'obturation en IPS Empress Direct est déjà polie, il convient de rendre préalablement la surface rugueuse puis appliquer un film d'Heliobond avant d'appliquer de nouveau IPS Empress Direct.
2. IPS Empress Direct doit être utilisé à température ambiante. A la température du réfrigérateur, l'extrusion du matériau peut s'avérer plus difficile.
3. ⊗ Usage unique. Pour des raisons d'hygiène, si IPS Empress Direct est appliqué directement en bouche à l'aide d'un Cavifil, n'utiliser ce Cavifil qu'une seule fois (prévention des contaminations entre patients).
4. Ne pas désinfecter les seringues ou Cavifils à l'aide d'agents oxydants.

Recommandations
Eviter le contact d'IPS Empress Direct non polymérisé avec la peau, les muqueuses et les yeux.
IPS Empress Direct pouvant être à ce stade légèrement irritant et conduire à une sensibilisation au méthacrylate.
Les gants médicaux du commerce ne procurent pas une protection suffisante contre les sensibilités aux méthacrylates.

Conditions de stockage
– Conserver le produit à une température comprise entre 2 et 28 °C.
– Refermer les seringues / Cavifils immédiatement après prélèvement du matériau, une exposition à la lumière pouvant provoquer une polymérisation prématurée.
– Ne pas utiliser IPS Empress Direct au-delà de la date de péremption.
– Délai de conservation : se référer aux indications figurant sur les Cavifils, les seringues ou l'emballage.

Ne pas laisser à la portée des enfants !
Réservé à l'usage exclusif du Chirurgien-Dentiste.

Ce produit a été développé en vue d'une utilisation dans le domaine dentaire et doit être mis en œuvre selon le mode d'emploi. Les dommages résultant du non-respect de ces prescriptions ou d'une utilisation à d'autres fins que celles indiquées n'engagent pas la responsabilité du fabricant. L'utilisateur est tenu de vérifier sous sa propre responsabilité l'appropriation du produit à l'utilisation prévue, et ce d'autant plus si celle-ci n'est pas citée dans le mode d'emploi.

Italiano

Descrizione
IPS Empress® Direct è un composito nanoibrido, radiopaco, fotoindurente basato sulla moderna tecnologia dei compositi per la realizzazione di restauri di massime esigenze. IPS Empress Direct è fotopolimerizzabile con luce nella lunghezza d'onda nel campo di 400–500 nm (luce blu).

Colori
Sono disponibili 32 colori e 5 diverse gradazioni di traslucenza.
IPS Empress Direct é disponibile nei seguenti colori:
Colori smalto
A1, A2, A3, A3.5, A4, B1, B2, B3, B4, C1, C2, C3, D2, D3, Bleach-L, Bleach-XL
Colori dentina
A1, A2, A3, A3.5, A4, IVA5, IVA6, B1, B2, C3, D2, Bleach-L, Bleach-XL
Colori traslucenti
Trans 20, Trans 30, Opal* *Non radiopaco in seguito all'elevata trasparenza

Composizione
La matrice del monomero è composta da dimetacrilati (20 – 21,5% in peso, colore opalescente 17% in peso). I riempitivi sono composti da vetro di bario, trifluoruro di itterbio, ossido misto, biossido di silicio e copolimero (77,5 - 79% in peso, colore opalescente 83% in peso). Inoltre sono contenuti additivi, catalizzatori, stabilizzatori e pigmenti (< 1,0% in peso). Il contenuto totale di riempitivi inorganici è di 75 – 79% in peso, rispettiv. 52 - 59 % in volume (colore opalescente 60,5% in peso rispettiv. 45% in volume).
La grandezza delle particelle dei riempitivi inorganici è compresa tra 40 nm e 3000 nm, con una dimensione media di 550 nm.

Indicazioni
– Restauri di denti anteriori (Classe III, IV)
– Restauri di denti latero-posteriori (Classe I e II)
– Restauri cervicali (Classe V, p.es. carie in zona cervicale, erosioni radicolari, difetti cuneiformi)
– Splintaggio e correzioni di forma (p.es. chiusura del diastema, chiusura di triangoli interdentali, prolungamento dei bordi incisali)
– Faccette dirette

Controindicazioni
La realizzazione di restauri diretti in IPS Empress Direct è controindicata in caso di:
– impossibilità di realizzare un campo operatorio asciutto oppure non si possano seguire le tecniche d'applicazione prescritte.
– in caso di accertata allergia ad uno dei componenti di IPS Empress Direct.

Effetti collaterali
In rari casi, componenti di IPS Empress Direct possono condurre ad una sensibilizzazione. In tal caso sospendere l'utilizzo del prodotto. Per evitare eventuali reazioni pulpari, si consiglia di ricoprire le zone in prossimità pulpare con prodotti indicati per la protezione pulpo/dentinale (nelle zone attigue alla polpa applicare in modo puntiforme un preparato a base d'idrossido di calcio e ricoprire con un idoneo sottofondo).

Interazioni
Le sostanze a base di eugenolo o olio di garofano possono inibire la polimerizzazione dei compositi. Pertanto se ne sconsiglia l'utilizzo in concomitanza con IPS Empress Direct. Il contatto con collutori cationici, con rilevatori di placca o con clorexidina può causare discromie.

Modalità d'uso
1. Determinazione del colore
Detergere i denti prima di procedere con la scelta del colore. Il colore deve essere determinato con i denti ancora umidi. Il colore dentinale può essere determinato direttamente sul dente preparato oppure cervicalmente sul canino.
2. Isolamento
E' necessario un appropriato isolamento del campo operatorio, preferibilmente con diga (p. es. OptraDam® Plus).
3. Preparazione cavitaria
Preparare la cavità secondo i principi della tecnica adesiva, cercando di preservare i tessuti duri del dente. Per le cavità dei settori anteriori si consiglia la bisellatura dello smalto, mentre per quelle dei settori posteriori si consiglia di smussare o arrotondare gli spigoli vivi dello smalto con fresa di rifinitura diamantata a granulometria fine (25–40 µm). I difetti cervicali non cariosi non devono essere preparati, ma solo detersi con pomice o pasta detergente atta allo scopo, servendosi di coppette di gomma o spazzolini rotanti. In caso di dentina sclerotica, un leggero irruvidimento superficiale con fresa a rosetta può migliorare il legame adesivo. Infine, si eliminano tutti gli eventuali residui da preparazione con spray d'acqua e si asciuga la cavità con soffio d'aria privo d'olio.
4. Sottofondo / Protezione pulpare
Nel caso si utilizzi un adesivo smalto-dentinale, è preferibile rinunciare ad un sottofondo. Solamente in cavità molto profonde, proteggere l'area in prossimità pulpare con un materiale a base di idrossido di calcio (p.es. ApexCal®), applicato in modo puntiforme. Il tutto è poi ricoperto da un cemento resistente alla pressione (p. es. un cemento vetroionomerico come Vivaglass® Liner). Non ricoprire le restanti pareti cavitarie, che dovranno servire per l'adesione con un adesivo smalto-dentinale.
5. Applicazione della matrice / cuneo interdentale
Nelle cavità con interessamento prossimale si consiglia l'uso di una matrice circolare o una matrice parziale, che possono essere fissate e stabilizzate con cunei.
6. Condizionamento / applicazione dell'adesivo
Condizionamento ed applicazione dell'adesivo secondo le istruzioni d'uso del prodotto utilizzato. Ivoclar Vivadent consiglia l'uso dell'adesivo Syntac® oppure ExciTE® F (in combinazione alla mordenzatura con acido ortofosforico) oppure degli adesivi automordenzanti quali AdheSE® oppure AdheSE® One F.
7. Applicazione di IPS Empress Direct
– Poiché per IPS Empress Direct è disponibile un ampio numero di colori dentinali ed incisali in diverse trasparenze e traslucenze, per la realizzazione di particolari e dettagli anatomici, qualsiasi tecnica di stratificazione è utilizzabile in modo creativo e consente di ottenere restauri altamente estetici. Utilizzando p.es. OptraSculpt®/OptraSculpt® Pad il restauro può essere modellato.IPS Empress Direct deve essere stratificato in spessori di max. 2 mm rispettiv. 1,5 mm (colori dentinali).
– Una fotopolimerizzazione accurata con lampada ad intensità adeguata garantisce una polimerizzazione del composito in profondità. Per il tempo di esposizione (Exposure Time) e l'intensità luminosa (Light Intensity), vedi tabella 1. Tenere il conduttore ottico il più vicino possibile al composito.
– Se si usa una matrice metallica, dopo averla rimossa, fotopolimerizzare ulteriormente dal lato vestibolare o linguo-palatale, qualora non è stata utilizzata una lampada fotopolimerizzante Bluephase®, oppure se non è stato possibile posizionare il conduttore ottico in modo ideale, p.es. in caso di distanza dal composito oppure in caso di angolazione di irradiazione divergente.
– Oggigiorno, in molti casi, si applica un primo strato di composito fluido per creare un fondo cavitario uniforme e contemporaneamente facilitare l'adattamento del materiale da restauro utilizzato. L'uso di un composito fluido (p.es. Tetric EvoFlow®) in un primo strato sottile è facoltativamente possibile. Questo strato deve essere indurito separatamente (attenersi alle relative istruzioni d'uso).
8. Rifinitura / Controllo dell'occlusione / Lucidatura
Dopo la polimerizzazione rimuovere le eccedenze del materiale con appositi strumenti per rifinitura (p.es. Astropol® F) o frese diamantate a granulometria fine. Rimuovere le eccedenze interprossimali con frese diamantate, frese per rifinitura al carburo di tungsteno, con strisce o dischi flessibili di rifinitura. Controllare ed eventualmente ritoccare l'occlusione e l'articolazione al fine di evitare precontatti o indesiderati decorsi articolari a livello della superficie del restauro. La lucidatura a specchio viene realizzata con gommini in silicone (p.es. Astropol P, Astropol HP rispettiv. Astrobrush oppure OptraPol® Next Generation), con dischi e con strisce di lucidatura.

Avvertenze particolari
1. In caso di correzioni IPS Empress Direct può essere applicato anche direttamente su materiale polimerizzato. Se il restauro in IPS Empress Direct è già stato lucidato, prima di apportare del nuovo materiale composito IPS Empress Direct, si deve irruvidire la zona interessata ed umettarla con Heliobond.
2. IPS Empress Direct deve essere utilizzato a temperatura ambiente. A temperatura di frigorifero l'estrusione del materiale può risultare difficile.
3. Monouso. Se IPS Empress Direct viene applicato direttamente in cavità con il Cavifil, per motivi igienici si consiglia l'utilizzo del Cavifil una volta sola (prevenzione di infezioni crociate tra pazienti).
4. Non disinfettare siringhe o Cavifil con disinfettanti ossidanti.

Avvertenza
Evitare il contatto diretto di IPS Empress Direct non polimerizzato con cute, mucose ed occhi.
IPS Empress Direct allo stato non polimerizzato può avere un leggero effetto irritante e indurre una sensibilizzazione ai metacrilati.
I normali guanti ad uso medico non proteggono da sensibilizzazione ai metacrilati.

Conservazione
– Temperatura di conservazione 2–28 °C.
– Chiudere le siringhe/Cavifil immediatamente dopo l'uso. L'esposizione alla luce causa una polimerizzazione prematura del materiale.
– Non utilizzare IPS Empress Direct dopo la data di scadenza.
– Scadenza: vedi avvertenze su Cavifil, siringa, confezionamento.

Tenere lontano dalla portata dei bambini!
Solo per uso odontoiatrico!

Il presente prodotto è destinato all'uso in campo odontoiatrico e deve essere utilizzato in conformità con le relative istruzioni per l'uso. Il produttore declina qualsiasi responsabilità per eventuali danni che possano derivare dall'utilizzo improprio o da utilizzi diversi dalla prevista finalità d'uso. Inoltre, l'utilizzatore è tenuto a verificare prima dell'uso l'idoneità e la possibilità di utilizzo del prodotto per il previsto scopo d'uso, tanto più se tale scopo d'uso non è menzionato nelle corrispondenti istruzioni per l'uso.

Español

Descripción
IPS Empress Direct es un composite nanohíbrido, fotopolimerizable y radiopaco que está basado en tecnología punta, indicado para la realización de restauraciones que cumplen con las más altas exigencias. IPS Empress Direct polimeriza con luz en la longitud de onda de 400-500 nm (luz azul).

Colores
IPS Empress Direct se ofrece en 32 colores distintos y 5 grados de translucidez diferentes.
Se dispone de los siguientes colores:
Colores esmaltes
A1, A2, A3, A3.5, A4, B1, B2, B3, B4, C1, C2, C3, D2, D3, Bleach L, Bleach XL
Colores dentinas
A1, A2, A3, A3.5, A4, IVA5, IVA6, B1, B2, C3, D2, Bleach L, Bleach XL
Colores translúcidos
Trans 20, Trans 30, Opal*

*No radiopaco debido a su alta transparencia

Composición
La matriz de monómero se compone de dimetacrilatos (20-21,5 % en peso, color opalescente 17 % en peso). Los rellenos contienen vidrio de bario, trifluoruro de iterbio, óxidos mixtos, dióxido de silicio y copolímeros (77,5-79 % en peso, color opalescente 83 % en peso). Componentes adicionales: aditivos, catalizadores, estabilizadores y pigmentos (<1,0 % en peso). El contenido total de rellenos inorgánicos es de 75-79 % en peso o 52-59 % en volumen (color opalescente 60,5 % en peso o 45 % en volumen). El tamaño de partícula de los rellenos inorgánicos oscila entre 40 nm y 3000 nm con un tamaño de partícula medio de 550 nm.

Indicaciones
– Restauraciones anteriores (Clases III, IV).
– Restauraciones en la zona posterior (Clase I y II).
– Restauraciones de cuellos (Clase V, ej. en caso de caries cervical, erosiones radiculares, defectos cuneiformes).
– Correcciones de posición y formas de dientes (ej. cierre de diastemas, cierre de 'espacios negros', alargamiento de bordes incisales).
– Carillas directas.

Contraindicaciones
La realización de restauraciones con IPS Empress Direct está contraindicada si:
– no se puede establecer un aislamiento seco del campo de trabajo o aplicar la técnica estipulada
– el paciente presenta alergia conocida a cualquiera de los componentes de IPS Empress Direct

Efectos secundarios
En casos aislados, los componentes de IPS Empress Direct pueden provocar sensibilización, en cuyo caso no debe aplicarse IPS Empress Direct.
Para evitar una posible irritación de la pulpa, las áreas próximas a la misma deben protegerse con un protector pulpar-dentinario apropiado (aplicar selectivamente una preparación con base de hidróxido de calcio en las zonas próximas a la pulpa y cubrir con una adecuada obturación de base).

Interacciones
Los productos que contienen eugenol/esencia de clavo pueden inhibir la polimerización de los materiales de composite. Por consiguiente, debe evitarse la aplicación conjunta de dichos materiales con IPS Empress Direct. En combinación con colutorios catiónicos, agentes reveladores de placa y clorhexidina se pueden producir decoloraciones.

Aplicación
1. Toma de color
Limpiar los dientes antes de la toma de color.
El color de esmalte se toma con el diente aún húmedo. El color de la dentina se puede tomar bien colocando la muestra próxima al diente preparado o en la zona cervical del canino.
2. Aislamiento
Se requiere un aislamiento apropiado con dique de goma (ej. OptraDam® Plus).
3. Preparación de la cavidad
La preparación de la cavidad se realiza de acuerdo con los requisitos de la técnica adhesiva, es decir protegiendo la estructura del diente. Los bordes del esmalte de la región anterior se biselan y, en la zona posterior, sólo deben redondearse los bordes adamantinos cortantes (diamantes de acabado, 25-40 µm). Los defectos cervicales sin caries no se preparan y solo se limpian con piedra pómez u otras pastas de limpieza apropiada con copas de goma o cepillos rotatorios. En el caso de la dentina esclerótica, un reacondicionamiento superficial con una fresa redonda puede mejorar la adherencia. Seguidamente, se eliminan los residuos de la cavidad con agua pulverizada. La cavidad se seca con aire libre de agua y grasa.
4. Protección pulpar/base
No aplicar un material de base cuando se aplique un agente adhesivo esmalte-dentinario. Cubrir únicamente las zonas muy profundas próximas a pulpa con un material de hidróxido de calcio (ej. ApexCal®) y seguidamente aplicar un cemento resistente a la presión (ej. cemento de ionómero de vidrio, como por ejemplo Vivaglass® Liner). No cubrir otras paredes cavitarias, ya que se pueden utilizar como soporte de la adhesión con el adhesivo esmalte/dentinario.
5. Colocación de la matriz / cuña interdental
Utilizar una matriz envolvente para las cavidades que afecten al área proximal o una matriz transversal y una cuña.
6. Acondicionamiento / aplicación de agente adhesivo
Acondicionar la estructura dental y aplicar el agente adhesivo de acuerdo a las instrucciones de uso del producto utilizado. Nosotros recomendamos la aplicación de Syntac® o ExciTE® F (con grabado de ácido fosfórico) o AdheSE® o AdheSE® One F, adhesivos autograbantes.
7. Aplicación de IPS Empress Direct
– Gracias a la disponibilidad del gran número de colores IPS Empress Direct Dentina y Esmalte, con diferentes niveles de translucidez para la creación de detalles anatómicos, se puede aplicar de manera creativa cualquier

técnica de estratificación para obtener restauraciones altamente estéticas. Para un resultado óptimo, IPS Empress Direct se aplica en capas de un grosor máximo de 2 mm (o 1,5 mm de colores dentinarios) y se adaptan por ejemplo con OptraSculpt®/OptraSculpt® Pad.
– La suficiente exposición a la lámpara de polimerización previene de la polimerización incompleta. Para las recomendaciones en cuanto al tiempo de exposición (Exposure time) e intensidad de luz (Light Intensity), ver tabla 1. La ventana de emisión de luz se debe mantener lo más cerca posible de la superficie del material de restauración.
– Cuando utilice una matriz metálica, la restauración se debe polimerizar además desde el aspecto bucal o lingual/palatal después de retirar la matriz, si no se ha empleado la luz polimerizadora Bluephase® o si la sonda de luz no pudo posicionarse de forma óptima (por ejemplo lejos del material o sin radiación divergente).
– En muchos casos, actualmente se emplea un composite fluido para la capa inicial para crear una oquedad homogénea y para simplificar la adaptación del material de restauración que se emplea posteriormente. El uso de un composite fluido (por ejemplo Tetric EvoFlow®) como capa fina inicial es opcional. Esta capa debe fotopolimerizarse independientemente (deben observarse las instrucciones de uso respectivas).

8. Acabado / Revisión de la oclusión / Pulido

Eliminar el exceso de material con puntas de acabado apropiadas (ej. Astropol® F) o diamantes finos después de la polimerización. Eliminar el exceso proximal con puntas de diamante, puntas de acabado de carburo de tungsteno, tiras o discos flexibles de acabado. Se revisa la oclusión y articulación y se aplican las correspondientes correcciones con fresado para evitar contactos prematuros o pistas de articulación indeseadas sobre la superficie de las restauraciones. Utilizar pulidores de silicona (e.g. Astropol® P, Astropol® HP, Astrobrush o OptraPol® Next Generation), así como discos y tiras de pulido para pulir las restauraciones a alto brillo duradero.

Información adicional

1. Si fuera necesario, se puede aplicar directamente material IPS Empress Direct adicional sobre material polimerizado. Si la restauración de IPS Empress Direct ya ha sido pulida, primero se crean rugosidades y se humectan con Heliobond antes de aplicar IPS Empress Direct fresco.
2. IPS Empress Direct debe tener temperatura ambiente cuando se aplique. Las temperaturas frias hacen que el material sea difícil de extraer del envase.
3. Diseñado para un solo uso. Si se aplica IPS Empress Direct desde el Cavifil directamente en la boca del paciente, el Cavifil debe desecharse después del uso para evitar la contaminación cruzada entre pacientes.
4. Las jeringas o Cavifils no deben desinfectarse con agentes de desinfección oxidantes.

Avisos

IPS Empress Direct sin polimerizar no debe entrar en contacto con la piel, las membranas ni los ojos. IPS Empress Direct sin polimerizar puede tener un ligero efecto irritante y puede producir sensibilización a los metacrilatos. Los guantes médicos comerciales no proporcionan protección a la sensibilización a los metacrilatos.

Almacenamiento

– Temperatura de almacenamiento 2-28º C.
– Cerrar las jeringas/Cavifils inmediatamente después de su uso. La exposición a la luz causa polimerización prematura.
– No utilizar IPS Empress Direct después de la fecha de caducidad.
– Caducidad: ver información en Cavifils, jeringas y envases.

Conservar el material fuera del alcance de los niños.
Sólo para uso odontológico.

El material ha sido fabricado para su uso dentaly debe manipularse según instrucciones de uso. El fabricante no se hace responsable de los daños ocasionados por otros usos o una manipulación indebida. Además, el usuario está obligado a comprobar, bájo su propia responsabilidad, antes de su uso, si el material es apto para los fines previstos, sobre todo si éstos no figuran en las instrucciones de uso.

Português

Descrição

IPS Empress® Direct é um compósito nano-híbrido, radiopaco, fotopolimerizável, baseado na mais recente tecnologia, adequado para a confecção de restaurações que satisfazem às mais altas exigências. IPS Empress Direct polimeriza com luz de comprimento de onda de 400-500 nm (lâmpada azul).

Cores

IPS Empress Direct é oferecido em 32 diferentes cores e em 5 diferentes níveis de translucidez.
As seguintes cores estão disponíveis:

Cores de esmalte
A1, A2, A3, A3.5, A4, B1, B2, B3, B4, C1, C2, C3, D2, D3, Bleach-L, Bleach-XL
Cores de dentina
A1, A2, A3, A3.5, A4, IVA5, IVA6, B1, B2, C3, D2, Bleach-L, Bleach-XL
Cores translúcidas
Trans 20, Trans 30, Opal*

*Não radiopaco devido à sua alta transparência.

Composição

A matriz de monômero é composta de dimetacrilatos (20-21,5 % em peso, cor opalescente 17 % em peso). As partículas são constituídas por vidro de bário, trifluoreto de itérbio, óxidos mistos, dióxido de silício e copolímero (77,5-79 % em peso, cor opalescente 83 % em peso). Aditivos, catalisadores, estabilizadores e pigmentos (< 1,0 % em peso) são componentes adicionais. O conteúdo total de partículas inorgânicas é de 75-79 % em peso ou 52-59 % em volume (cor opalescente 60,5% em peso ou 45 % em volume). O tamanho das partículas inorgânicas está situado entre 40 nm e 3000 nm, com um tamanho médio de partícula de 550 nm.

Indicação

– Restaurações anteriores (Classes III e IV).
– Restaurações posteriores (Classes I e II).
– Restaurações de Classe V (p.ex., cáries cervicais, erosões radiculares, defeitos cuneiformes).
– Correções de forma e de posicionamento dos dentes (p.ex., fechamento de diastemas e de "triângulos pretos" interdentais, alongamentos da margem incisal).
– Facetas diretas.

Contra-indicação

A confecção de restaurações de IPS Empress Direct está contra-indicada
– Quando um campo operatório seco não puder ser estabelecido ou a téc-

nica de trabalho estipulada não puder ser aplicada.
– Quando o paciente for alérgico a qualquer um dos ingredientes do IPS Empress Direct.

Efeitos colaterais
Em casos raros, os componentes de IPS Empress Direct podem levar à sensibilização. IPS Empress Direct não deve ser usado em tais casos.
Para evitar possível irritação pulpar, as áreas em contato direto com a polpa devem ser protegidas com adequada proteção dentino-pulpar (aplicar hidróxido de cálcio nas áreas em contato direto com a polpa e recobrir com um forramento apropriado).

Interações
Os materiais que contêm eugenol ou óleo de cravo podem inibir a polimerização de compósitos. Assim, a aplicação destes materiais com IPS Empress Direct deve ser evitada. O uso de colutórios catiônicos, agentes para eliminação de placas bacterianas ou clorexidina pode promover descolorações.

Aplicação
1. Escolha da cor
Efetuar a limpeza do dente, antes da escolha da cor. A cor de esmalte deve ser selecionada com o dente úmido. A cor de dentina pode ser tomada posicionando o amostra da escala de cores próxima do dente preparado ou próximo à área cervical do canino.
2. Isolamento
Isolamento apropriado, de preferência efetuado com um dique de borracha (p.ex., OptraDam® Plus).
3. Preparo da cavidade
O preparo da cavidade deve ser conduzido de acordo com as exigências da técnica adesiva, isto é, protegendo a estrutura dental. As margens de esmalte, na região anterior, devem ser biseladas. Na região posterior, apenas as margens de esmalte devem ser arredondadas (pontas diamantadas finas, 25-40 µm). Os defeitos cervicais, sem cáries, não devem ser preparados, mas apenas limpos com pedra-pomes ou outras adequadas pastas de limpeza, com o auxílio de escovas ou de taças de borracha. Em casos de dentina esclerótica, a ativação da superfície com uma broca esférica pode melhorar a resistência adesiva. A seguir, remover todos os resíduos da cavidade com spray de água. A secagem da cavidade deverá ser feita com ar, isento de água e óleo.
4. Proteção pulpar / Forramento
Não aplicar material de base (forramento) quando for usado um agente de união para esmalte/dentina. Apenas cobrir as áreas profundas, próximas da polpa, com um material de hidróxido de cálcio (p.ex., ApexCal®) e, em seguida, recobrir com um cimento resistente à pressão (p.ex., cimento de ionômero de vidro, como Vivaglass® Liner). Não recobrir as outras paredes da cavidade, para que elas possam ser usadas para suportar a união com um adesivo para esmalte/dentina.
5. Colocação de matriz / cunha interdental
Use uma matriz circunferencial para cavidades que envolvem a área proximal ou matriz parcial e cunha.
6. Condicionamento / Aplicação do agente de união
Condicionar a estrutura dental e aplicar um agente de união, de acordo com as Instruções de Uso do produto que vai ser utilizado. Nós recomendamos o adesivos como Syntac®, ExciTE® F (com condicionamento de ácido fosfórico), ou adesivos autocondicionantes como AdheSE® ou AdheSE® One F.
7. Aplicação de IPS Empress Direct
– Devido ao grande número de cores de esmalte e de dentina, com diferentes níveis de translucidez, apresentados pelo IPS Empress Direct e que estão disponíveis para a criação de detalhes anatômicos, qualquer técnica de estratificação pode ser empregada para a fabricação de restaurações altamente estéticas. Para excelentes resultados, aplicar IPS Empress Direct em camadas de no máximo 2 mm (ou 1,5 mm para cores de dentina) de espessura e adaptar com um adequado instrumento (p.ex., OptraSculpt®/OptraSculpt® Pad).
– A insuficiente exposição de luz de cura evita a completa polimerização. Para as recomendações relacionadas com tempo de exposição (Exposure Time) e intensidade de luz (Light Intensity), consultar a Tabela 1. Mantenha a janela de emissão de luz o mais próximo possível da superfície do material restaurador.
– Quando for usar uma matriz metálica, polimerize também a restauração por vestibular ou lingual/palatal após remover a matriz, se não for usada uma lâmpada Bluephase® ou se a ponteira de luz não puder ser posicionada idealmente (por ex. longe do material ou com radiação divergente).
– Em muitos casos, uma resina de baixa viscosidade atualmente é usada na camada inicial para criar um assoalho cavitário regular e simplificar a adaptação do material restaurador usado subsequentemente. Usar uma resina de baixa viscosidade (por ex. Tetric EvoFlow®) como uma camada inicial fina é opcional. Esta camada deve ser fotoativada em um passo separado (as orientações das Instruções de Uso devem ser observadas).
8. Acabamento / Verificação da oclusão / Polimento
Após a polimerização, remover o excesso de material com pontas de acabamento adequadas (p.ex., Astropol® F) ou com pontas diamantadas finas. Remover os excessos proximais com pontas diamantadas de acabamento, instrumentos de acabamento de carboneto de tungstênio, tiras de acabamento ou discos finos flexíveis. Verificar a oclusão, a articulação e efetuar os desgastes de correção necessários para evitar contatos prematuros e evitar trajetórias indesejáveis da articulação sobre a superfície das restaurações. Utilizar polidores de silicone (p.ex., Astropol®, Astropol® HP, Astrobrush ou OptraPol® Next Generation), tiras de polimento e discos de polimento para polir as restaurações e obter um alto e durável brilho.

Informações adicionais
1. Quando necessário, o IPS Empress Direct pode ser aplicado diretamente sobre material já polimerizado. Se a restauração de IPS Empress Direct já foi polida, este material deverá ser, em primeiro lugar, asperizado e umedecido com Heliobond, antes da aplicação do novo IPS Empress Direct.
2. IPS Empress Direct deve estar à temperatura ambiente quando for aplicado. Temperaturas frias dificultam a sua extrusão.
3. Apenas para uso único. Quando, a partir do Cavifil, o IPS Empress Direct for aplicado diretamente na boca, o Cavifil deve ser usado apenas uma única vez por razões higiênicas (prevenção de contaminação cruzada entre pacientes).
4. Não usar desinfetantes oxidantes para a desinfecção de seringas e Cavifils.

Advertências
Evitar o contato do IPS Empress Direct não polimerizado com a pele, mucosa e olhos. O IPS Empress Direct não polimerizado pode causar ligeira irritação e pode promover sensibilização aos metacrilatos. As luvas médicas comerciais

não promovem proteção contra o efeito de sensibilização dos metacrilatos.

Armazenagem
– Temperatura de armazenagem: 2-28 ºC.
– Após o uso, fechar imediatamente seringas e Cavifils. A exposição à luz pode promover polimerização prematura.
– Não usar IPS Empress Direct com prazo de validade vencido
– Vida útil: ver informações em Cavifils, seringas e nas embalagens.

Manter fora do alcance das crianças.
Somente para uso odontológico.

Este material foi fabricado somente para uso dental e deve ser manipulado de acordo com as Instruções de Uso. O fabricante não é responsável pelos danos causados por outros usos ou por manipulação incorreta. Além disto, o usuário está obrigado a comprovar, antes do emprego e sob sua responsabilidade, se este material é compatível com a utilização desejada, principalmente quando esta utilização não está indicada nas Instruções de Uso.

Svenska

Beskrivning
IPS Empress® Direct är en ljushärdande, radiopak nanohybridkomposit, baserad på den modernaste teknologin. Materialet är lämpligt att använda till framställning av restaurationer som tillfredsställer de högsta krav. IPS Empress Direct härdas med ljus i våglängdsområdet 400-500 nm (blått ljus).

Färger
IPS Empress Direct erbjuds i 32 olika färger och 5 olika grader av translucens. Följande färger finns tillgängliga:
Emaljfärger
A1, A2, A3, A3.5, A4, B1, B2, B3, B4, C1, C2, C3, D2, D3, Bleach-L, Bleach-XL
Dentinfärger
A1, A2, A3, A3.5, A4, IVA5, IVA6, B1, B2, C3, D2, Bleach-L, Bleach-XL
Translucenta färger
Trans 20, Trans 30, Opal*

*Hög transparens och därför inte röntgentät

Sammansättning
Monomermatrixen består av dimetakrylater (20-21,5 vikts%, opalescent färg 17 vikts%). Fillers består av bariumglas, ytterbiumtrifluorid, blandoxid, kiseldioxid och copolymer (77,5-79 vikts%, opalescent färg 83 vikts%). Ytterligare innehåll: tillsatsämnen, katalysatorer, stabilisatorer och pigment (< 1,0 vikts%). Total andel oorganisk filler är 75-79 vikts% eller 52-59 vol% (opalescent färg 60,5 vikts% eller 45 vol%). Partikelstorleken på den oorganiska fillern ligger mellan 40 nm och 3000 nm med en medelstorlek av 550 nm.

Indikationer
– Anteriora restaurationer (Klass III, IV)
– Restaurationer i posteriora områden (Klass I och II)
– Tandhalsfyllningar (Klass V, t.ex. vid cervikal karies, roterosioner, kilformiga defekter)
– Korrektion av tandposition och tandform (t.ex. förslutning av diastema, förslutning av interdentala "svarta trianglar", förlängning av incisalkanten)
– Framställning av direkta fasader

Kontraindikationer
Fyllningar med IPS Empress Direct är kontraindicerade
– om ett torrt arbetsfält inte kan erhållas, eller om den beskrivna arbetstekniken inte kan användas
– om en patient har känd allergi mot något av innehållet i IPS Empress Direct.

Biverkningar
I sällsynta fall kan komponenter i IPS Empress Direct leda till sensibilisering. I dessa fall ska IPS Empress Direct inte användas. För att undvika eventuell irritation på pulpan, ska områden nära pulpan täckas med ett lämpligt pulpa/dentinskydd (applicera selektivt ett kalciumhydroxid-preparat på områden nära pulpan och täck med en passande liner).

Interaktioner
Material som innehåller eugenol/nejlikeolja kan förhindra polymerisationen av kompositmaterial. Därför ska material som innehåller eugenol/nejlikeolja inte användas tillsammans med IPS Empress Direct. Missfärgning kan uppstå vid användandet av katjoniska munsköljningar, plackpåvisande medel samt klorhexidin.

Användning
1. Färgval
Rengör tänderna före färgvalet. Emaljfärgen väljs på fuktig tand. Dentinfärgen kan antingen väljas med färgprovet intill den preparerade tanden eller intill cervikalområdet på hörntanden.
2. Torrläggning
Torrlägg ordentligt, helst med kofferdam (t.ex. OptraDam® Plus).
3. Kavitetspreparation
Kavitetspreparation utförs enligt riktlinjerna för adhesiv teknik, d.v.s. sparande av tandsubstans. Emaljkanter i anteriort område ska kantskäras (bevel). I posteriort område ska endast skarpa emaljkanter rundas av (finisheringsdiamant, 25-40 µm). Kariesfria cervikala defekter ska inte prepareras, utan endast rengöras med pimpsten eller annan lämplig rengöringspasta med hjälp av gummikopp eller roterande borste. Vid fall av sklerotiskt dentin kan ytlig rekonditionering med en rosenborr förbättra vidhäftningen. Kaviteten rengörs sedan med vattenbläster. Kaviteten torkas med vatten- och oljefri luft.
4. Pulpaskydd/ Underfyllning
Applicera inget basmaterial när du använder ett emalj/dentin-bondingmaterial. Täck endast mycket djupa områden nära pulpan med ett kalciumhydroxidmaterial (t.ex. ApexCal®) och applicera sedan ett tryckresistent cement (t.ex. glasjonomercement som Vivaglass® Liner). Täck inte övriga kavitetsväggar då dessa kan användas som stöd för emalj/dentin-adhesivet.
5. Applicering av matris/interdentalkil
Lägg ett matrisband runt tanden vid approximala fyllningar (alternativt en sektionsmatris) och placera kilar.
6. Konditionering/ Applicering av bonding
Konditionera tanden och applicera bondingmedlet enligt bruksanvisningen för det medel som används. Vi rekommenderar Syntac® eller ExciTE® F (med fosforsyre-etsning) eller AdheSE® eller AdheSE® One F självetsande adhesiv.
7. Applicering av IPS Empress Direct
– Då IPS Empress Direct omfattar ett stort antal dentin- och emaljfärger i olika translucenser för framställning av anatomiska detaljer, kan olika skiktningstekniker användas kreativt för att skapa högestetiska restaurationer. För att erhålla ett optimalt resultat appliceras IPS Empress Direct i skikt på max 2 mm eller 1,5 mm (dentinfärger) och bearbetas med ett lämpligt instrument (t.ex. OptraSculpt®/OptraSculpt® Pad).

– Otillräckligt med ljus från ljusledaren kan skapa en partiell härdning. För rekommendationer gällande exponeringstid (Exposure Time) och ljusintensitet (Light Intensity) se tabell 1. Håll ljusledarens fönster så nära ytan på restaurationsmaterialet som möjligt.
– När en metallmatris används ska man ljushärda även från buckalt eller lingualt/palatinalt håll efter att matrisen har tagits bort. Rekommenderas också om annan härdlampa än Bluephase® använts eller ljusledaren inte kunnat placeras på ett optimalt sätt (t.ex. långt från restaurationsmaterialet eller med divergerande strålning).
– I många fall används nu en flytande komposit för det initiala skiktet för att skapa ett jämnt kavitetsgolv och förenkla appliceringen av det påföljande restaurationsmaterialet. Användning av en flytande komposit (t.ex. Tetric EvoFlow®) som initialt tunt skikt är valfritt. Detta lager måste ljushärdas i ett separat steg (instruktionerna i respektive bruksanvisning måste följas).

8. Finishering/ Ocklusionskontroll/ Polering
Avlägsna överskottsmaterial med lämplig finisherare (t.ex. Astropol® F) eller fina diamanter efter ljushärdningen. Avlägsna approximalt överskott med diamantfinisherare, hårdmetallfinisherare, finisheringsstrips eller böjliga finisheringsdiskar. Kontrollera ocklusion och artikulation och justera ytan så att prekontakter eller oönskat artikulationsmönster på restaurationens yta undviks. Använd silikonpolerare (t.ex. Astropol® P, Astropol® HP, Astrobrush eller OptraPol® Next Generation) samt polerdiskar och polerstrips för att polera restaurationen till bestående högglans.

Ytterligare information
1. Vid behov kan ytterligare IPS Empress Direct-material appliceras direkt på det polymeriserade materialet. Om IPS Empress Direct-restaurationen redan har polerats, måste ytan ruggas upp först och vätas med Heliobond® innan nytt IPS Empress Direct appliceras.
2. IPS Empress Direct ska ha rumstemperatur när det appliceras. För kallt material kan vara svårt att trycka ut.
3. Endast för engångsbruk. Om IPS Empress Direct appliceras från en cavifil direkt i patientens mun, får samma cavifil av hygieniska skäl (för att förhindra korskontaminering mellan patienter) inte användas på annan patient, utan ska kasseras.
4. Sprutor och cavifiller ska inte desinfekteras med oxiderande desinfektionsmedel.

Varning
Opolymeriserat IPS Empress Direct ska inte komma i kontakt med hud, slemhinna eller ögon. Opolymeriserat IPS Empress Direct kan vara lätt irriterande och leda till sensibilisering för metakrylater. Kommersiella medicinska skyddshandskar skyddar inte mot sensibilisering för metakrylater.

Förvaring
– Förvaringstemperatur: 2–28 °C.
– Förslut sprutor/cavifiller omedelbart efter användning. Exponering för ljus gör att materialet härdar i förtid.
– Använd inte IPS Empress Direct efter utgångsdatum.
– Hållbarhet: se information på cavifiller, sprutor och förpackning.

Förvaras oåtkomligt för barn
Endast för dentalt bruk

Detta material har utvecklats speciellt för dentalt bruk. Bearbetningen ska noga följa bruksanvisningen. Tillverkaren påtager sig inget ansvar för skador som uppkommer genom underlåtande att följa givna föreskrifter eller användning utöver de fastställda indikationsområdena. Användaren är ansvarig för kontrollen av produktens lämplighet för annat ändamål än vad som finns direkt uttryckt i bruksanvisningen. Beskrivningar och uppgifter utgör ingen garanti för egenskaper och är inte bindande.

Dansk

Beskrivelse
IPS Empress® Direct er et lyshærdende, radiopakt nano-hybridkomposit til restaureringer af højeste kvalitet og baseret på moderne teknologi. IPS Empress Direct hærder ved lys i et bølgelængdeområde på 400–500 nm (blåt lys).

Farver
Vi tilbyder 32 farver i 5 forskellige translucens-niveauer.
Fås i følgende farver:
Emaljefarver
A1, A2, A3, A3.5, A4, B1, B2, B3, B4, C1, C2, C3, D2, D3, Bleach-L, Bleach-XL
Dentinfarver
A1, A2, A3, A3.5, A4, IVA5, IVA6, B1, B2,C3, D2, Bleach-L, Bleach-XL
Translucente farver
Trans 20, Trans 30, Opal*

*Ikke radiopakt på grund af sin høje transparens

Sammensætning
Monomermatrixen består af dimethacrylater (20-21,5 vægt-%, opalescente farver 17 vægt-%). Fyldstofferne (fillers) består af bariumglas, ytterbiumtrifluorid, blandingsoxid, siliciumdioxid og copolymer (77,5-79 vægt-%, opalescente farver 83 vægt-%). Indeholder desuden additiver, katalysatorer, stabilisatorer og pigmenter (<1,0 vægt%). Det samlede indhold af uorganisk filler udgør 75-79 vægt-% eller 52-59 vol.-% (opalescente farver 60,5 vægt-% eller 45 vol.-%). Partikelstørrelsen ligger mellem 40 nm og 3000 nm med en middel partikelstørrelse på 550 nm.

Indikationer
– Fortandsfyldninger (klasse III, IV)
– Kindtandsfyldninger (klasse I og II)
– Tandhalsfyldninger (Klasse V, f.eks. ved cervical caries, rod-erosioner, kile formede defekter)
– Tandstillings- og tandforms-korrektioner (fx diastema-lukning, lukning af interdental-trekanter, forlængelse af incisalkanter)
– Direkte veneers

Kontraindikationer
Anvendelse af IPS Empress Direct-fyldninger er kontraindiceret
– hvis tilstrækkelig tørlægning eller den foreskrevne anvendelsesteknik ikke er mulig.
– ved kendt allergi mod bestanddele som indgår i IPS Empress Direct

Bivirkninger
Bestanddelene i IPS Empress Direct kan i sjældne tilfælde føre til sensibilisering. I sådanne tilfælde må produktet ikke anvendes. For at udelukke pulpale irritationer skal pulpanære områder beskyttes med en egnet pulpa-/dentinbeskyttelse (et calciumhydroxidholdigt præparat appliceres punktvis omkring pulpa og dækkes med en egnet bunddækning).

Interaktioner
Eugenol-/nellikeolieholdige produkter inhiberer afbindingen af kompositter. Sådanne materialer bør ikke anvendes sammen med IPS Empress Direct.

Misfarvning kan forekomme i kombination med kationiske mundskyllemidler, plakindfarvningsmidler og chlorhexidin.

Applicering

1. Farveprøve
Før valg af farve rengøres tænderne. Farven bestemmes, mens tænderne stadig er fugtige. Dentinfarven kan bestemmes direkte på den præparerede tand eller cervicalt på hjørnetanden.

2. Tørlægning
Grundig tørlægning, helst med kofferdam (f.eks. OptraDam® Plus), er påkrævet.

3. Kavitetspræparation
Kaviteten præpareres jf. reglerne for adhæsiv teknik dvs. substansbevarende. I anteriore områder skrånes emaljerandene. I posteriore områder må kun de skarpe emaljekanter brydes eller afrundes let (finkornet diamant 25–40 µm). Kariesfri tandhalsdefekter skal ikke præpareres, men kun rengøres med pimpsten hhv. en egnet pudsepasta og en pudsekop eller en roterende børste. Ved sklerotisk dentin kan superficiel bearbejdning med et rosenbor forbedre adhæsivbindingen. Efterfølgende fjernes alle rester i kaviteten med vandspray, og kaviteten tørres med vand- og oliefri luft.

4. Beskyttelse af pulpa / bunddækning
Ved anvendelse af et emalje-dentin-bondingmiddel bør en bunddækning ikke appliceres. Kun ved meget dybe, pulpanære områder skal disse arealer dækkes punktvis med et calciumhydroxid præparat (f.eks. ApexCal®). Herefter dækkes de pulpanære områder med en trykstabil cement (f.eks. glasionomercement som Vivaglass® Liner). De øvrige kavitetsvægge skal forblive udækkede således at de er tilgængelige for et emalje-dentin-adhæsiv.

5. Anlæg af matrice/interdentalkile
Til kaviteter med approksimal involvering anvendes enten en omsluttende matrice eller en sektionsmatrice. Interdentalkile anbringes.

6. Konditionering / Applicering af adhæsiv
Konditionering og applicering af bondingmidlet foretages i overensstemmelse med brugsanvisningen til det anvendte produkt. Ivoclar Vivadent anbefaler anvendelse af adhæsivsystemet Syntac® eller ExciTE® F (i forbindelse med fosforsyreætsning) eller det selvætsende adhæsiv AdheSE®/AdheSE® One F.

7. Applicering af IPS Empress Direct
- Da en stor del af dentin- og emaljefarverne i IPS Empress Direct står til rådighed i forskellige transparente og translucente farver med henblik på udformning af anatomiske og specifikke detaljer, kan enhver lag-teknik anvendes kreativt til opnåelse af yderst æstetiske restaureringer.
  Med henblik på et optimalt resultat skal IPS Empress Direct appliceres i lagtykkelser på maks. 2 mm eller 1,5 mm (dentinfarver) og adapteres med f.eks. OptraSculpt®/OptraSculpt® Pad.
- En tilstrækkelig belysning forhindrer en ufuldstændig polymerisation. For anbefalinger vedrørende eksponeringstid (Exposure Time) og lysintensitet (Light Intensity), se Tabel 1. Lysvinduet anbringes så tæt på fyldningsmaterialet som muligt.
- Ved anvendelse af metalmatricer skal restaureringen efter fjernelsen af matricen desuden belyses buccalt eller lingualt/palatinalt, hvis der ikke er anvendt Bluephase®-hærdningslys, eller hvis lyssonden ikke kan placeres optimalt (f.eks. ved for stor afstand til materialet eller ved en divergerende stråling).
- I mange tilfælde bliver der i dag anvendt en flowable (flydende) komposit som første lag for at opnå en ensartet kavitetsbund og samtidig lette adaptationen af det efterfølgende fyldningsmateriale. Anvendelse af en flowable komposit (f.eks. Tetric EvoFlow®) i et tyndt første lag er fakultativt muligt. Dette lag skal lyshærdes i et separat trin (anvisningerne i de respektive brugsanvisninger skal følges).

8. Beslibning / okklusionskontrol / polering
Efter polymerisering fjernes overskuddet med egnede finerere (f.eks. Astropol® F) eller et finkornet diamantbor. Approksimalt overskud fjernes med diamant-, hårdmetalfinerbor, pudseskiver eller finerstrips hhv. fleksible pudseskiver. Okklusion og artikulation kontrolleres og tilpasses, således at suprakontakter og uønskede artikulations-baner på fyldningen fjernes. Højglanspolering foretages med silikonepolerere (f.eks. Astropol® P, Astropol® HP, Astrobrush eller OptraPol® Next Generation) samt pudseskiver og -strips.

Bemærk

1. I forbindelse med korrektioner kan nyt IPS Empress Direct materiale appliceres direkte på allerede polymeriseret materiale. Hvis IPS Empress Direct restaureringen allerede er poleret, skal den først gøres ru og befugtes med Heliobond®, inden der appliceres nyt IPS Empress Direct.
2. IPS Empress Direct skal mindst have stuetemperatur ved anvendelsen. Ved køleskabstemperatur kan udpresning være vanskelig.
3. Kun til engangsbrug. Hvis IPS Empress Direct appliceres direkte fra Cavifil'en ind i patientens mund, må Cavifil'en af hygiejniske grunde kun anvendes til én patient for at undgå krydsinfektioner mellem patienter.
4. Sprøjter og Cavifils må ikke desinficeres med oxiderende desinfektionsmidler.

Advarsel

Ikke afbundet IPS Empress Direct bør ikke komme i kontakt med hud, slimhinder og øjne. IPS Empress Direct kan i uafbundet tilstand virke let lokalirriterende og kan føre til en sensibilisering mod methacrylater. De gængse medicinske handsker yder ikke beskyttelse mod sensibilitet over for methacrylater.

Opbevaring

- Opbevaringstemperatur: 2–28 °C
- Luk sprøjter/Cavifils straks efter brug. Eksponering over for lys fører til præmatur polymerisering.
- IPS Empress Direct må ikke anvendes efter udløb af holdbarhedsdato.
- Holdbarhedsdato: se udløbsdato på Cavifil, sprøjte eller emballagen.

Opbevares utilgængeligt for børn.
Kun til dentalt brug.

Materialet er blevet udviklet udelukkende til anvendelse i tandlægeindustrien. Bearbejdning skal udføres i nøje overensstemmelse med brugsanvisningen. For skader, der er opstået som følge af manglende overholdelse af anvisningerne eller det stipulerede anvendelsesområde, påtager producenten sig intet erstatningsansvar. Brugeren er forpligtet til at teste produkterne for deres egnethed og anvendelse til formål, der ikke er udtrykkeligt anført i brugsanvisningen. Beskrivelserne og data udgør ingen garanti for egenskaber og er ikke bindende.

Suomi

Kuvaus

IPS Empress® Direct on kokonaan uudenlainen valokovetteinen, radio-opaakki nanohybridiyhdistelmämuovi käytettäväksi paikkaushoidoissa. IPS Empress Direct kovettuu valolla aallonpituuden ollessa 400–500 nm (sinivalo).

Sävyt
Tuotevalikoimaan kuuluu 32 eri sävyä ja 5 eri läpikuultavuusastetta.
IPS Empress Direct on saatavissa seuraavina sävyinä:
Kiillesävyt
A1, A2, A3, A3.5, A4, B1, B2, B3, B4, C1, C2, C3, D2, D3, Bleach-L, Bleach-XL
Dentiinisävyt
A1, A2, A3, A3.5, A4, IVA5, IVA6, B1, B2, C3, D2, Bleach-L, Bleach-XL
Läpikuultavat sävyt
Trans 20, Trans 30, Opal* *Eivät ole läpikuultavuutensa vuoksi radio-opaakkeja

Koostumus
Monomeerimatriisi koostuu dimetakrylaateista (20–21,5 paino-%, opalisoiva sävy 17 paino-%). Fillereinä ovat bariumlasi, ytterbiumtrifluoridi, sekoitettu oksidi, piidioksidi ja kopolymeeri (77,5–79 paino-%, opalisoiva sävy 83 paino-%). Lisäksi materiaali sisältää lisäaineita, katalyyttejä, stabilisaattoreita ja pigmenttejä (<1,0 paino-%). Epäorgaanisten fillereiden kokonaismäärä on 75–79 paino-% ja 52–59 tilavuus-% (opalisoiva sävy 60,5 paino-% tai 45 tilavuus-%). Epäorgaanisten fillereiden ensisijainen hiukkaskoko on 40–3000 nm keskimääräisen hiukkaskoon ollessa 550 nm.

Indikaatio
– Etuhampaiden paikkaushoito (luokat III ja IV)
– Poskihampaiden paikkaushoito (luokat I ja II)
– Hampaan kaulojen paikkaushoito (luokka V, esim. hampaan kaulan karies, juurieroosio, kiilamaiset vauriot)
– Hampaiden asennon ja muodon korjaus (esim. diasteeman sulku, hampaiden välisten "kolmioiden" sulku, puremareunojen pidennys)
– Suorat kruunut

Vasta-aihe
IPS Empress Direct -täytteet ovat kontraindikoituja
– mikäli ei voida olla varmoja riittävästä kosteussulusta tai ei voida noudattaa ohjeiden mukaista käyttötekniikkaa
– mikäli potilaan tiedetään olevan allerginen jollekin IPS Empress Direct -materiaalin aineosalle.

Haittavaikutukset
Harvinaisissa tapauksissa IPS Empress Direct -sidosaineen aineosat saattavat aiheuttaa herkistymistä. Materiaalia ei tällöin tule käyttää. Pulpaärsytyksen välttämiseksi alueet, jotka ovat aivan pulpan läheisyydessä, tulee suojata sopivalla pulpa/dentiinisuoja-aineella (käytä kalsiumhydroksidipohjaista valmistetta aivan pulpan läheisyydessä ja peitä sopivalla eristemateriaalilla).

Yhteisvaikutukset
Eugenoli-/neilikkaöljypitoiset vaikuttavat aineet saattavat inhiboida yhdistelmämuovin kovettumisen. Tällaisia materiaaleja ei tule käyttää IPS Empress Directin kanssa. Kationiset suunhuuhteluaineet, plakkivärjäysaineet ja klooriheksidiini saattavat aiheuttaa materiaalin värjäytymistä.

Käyttö
1. Sävyn määrittely
Puhdista hampaat ennen sävyn määrittelyä. Valitse sävy hampaan ollessa vielä kostea. Dentiinisävy voidaan valita suoraan esivalmistellussa hampaassa tai kervikaalisesti kulmahampaassa.
2. Eristys
Eristä työskentelyalue kosteudelta mieluiten kofferdam-kumilla (esim. OptraDam® Plus).
3. Kaviteetin valmistelu
Kaviteetin preparointi suoritetaan adhesiivisen paikkaustekniikan sääntöjen mukaisesti eli kudosta säästäen. Viistä etuhampaiden kiillereunat. Takahampaiden alueella ainoastaan terävät kiillereunat pyöristetään (viimeistelytimantti, 25–40 µm). Karieksettomia kervikaalivaurioita ei preparoida, vaan ne puhdistetaan ainoastaan hohkakivellä tai sopivalla puhdistuspastalla käyttäen kumikuppeja tai pyöriviä harjoja. Skleroottisen dentiinin tapauksessa pinnallinen käsittely ruusuporalla voi edistää kiinnittymistä. Lopuksi kaikki jäänteet poistetaan kaviteetista vesisuihkeella, ja kaviteetti kuivataan vedettömällä ja öljyttömällä ilmalla.
4. Pulpan suojaus / eriste
Älä käytä eristemateriaalia käyttäessäsi kiille-/dentiinisidosainetta. Vain erittäin syvissä kaviteeteissa pulpaa lähellä olevat alueet peitetään kalsiumhydroksidipohjaisella materiaalilla (esim. ApexCal®), ja sen jälkeen paineen kestävällä sementillä (esim. lasi-ionomeerisementti, kuten Vivaglass® Liner). Älä peitä kaviteetin muita seinämiä, koska niitä käytetään kiille-/ dentiinisidosaineen sidospintoina.
5. Matriisin / kiilan kiinnittäminen
Käytä hampaan ympärille kierrettävää matriisia tai sektionaalista matriisia ja kiilaa kaviteeteissa, jotka ulottuvat proksimaalialueelle.
6. Esikäsittely / sidosaineen applikointi
Esikäsittele hammaskudos ja käytä sidosainetta tuotteen käyttöohjeiden mukaisesti. Suosittelemme Syntac®- tai ExciTE® F -sidosaineen (molemmat vaativat fosforihappoetsauksen) tai itse-etsaavan AdheSE®- tai AdheSE® One F -sidosaineen käyttöä.
7. IPS Empress Directin käyttö
– Koska IPS Empress Direct on saatavana useana eri dentiini- ja kiillesävynä sekä kirkkaus- ja läpikuultavuusasteena anatomisten erikoispiirteiden ja yksityiskohtien huomioinnin mahdollistamiseksi, kaikkia kerrostekniikoita voidaan käyttää luovalla tavalla hyvin esteettisen paikan valmistamiseksi. Optimaalisen tuloksen aikaansaamiseksi annostele IPS Empress Direct maks. 2 mm:n tai 1,5 mm:n (dentiinisävyt) kerroksina ja mukauta sopivalla instrumentilla (esim. OptraSculpt® / OptraSculpt® Pad).
– Riittävä valotus estää epätäydellisen polymeroitumisen. Kovetusaikaa (Exposure Time) ja valotehoa (Light Intensity) koskevat suositukset on annettu taulukossa 1. Pidä valokärki mahdollisimman lähellä täytemateriaalia.
– Metallimatriisia käytettäessä täytemateriaali on lisäksi polymeroitava bukkaalipuolelta tai linguaali-/palatinaalipuolelta matriisin poistamisen jälkeen, jos ei käytetä Bluephase®-polymerointivaloa tai jos valokärkeä ei voida asemoida ihanteellisesti (esim. liian suuri etäisyys täytemateriaaliin tai hajaantuva sirokulma).
– Nykyään käytetään useissa tapauksissa juoksevaa yhdistelmämuovia, jolla voidaan luoda kaviteettiin tasainen pohja ja joka helpottaa sen jälkeen käytettävän täytemateriaalin muokkausta. Juoksevan yhdistelmämuovin (esim. Tetric EvoFlow®) käyttö ohuena kerroksena on valinnaista. Tämä kerros on valokovetettava erikseen (noudata tuotteen käyttöohjeita).
8. Viimeistely / purennan tarkistus / kiillotus
Poista ylimääräinen materiaali sopivilla viimeistelijöillä (esim. Astropol® F) tai

hienoilla timanteilla polymeroinnin jälkeen. Poista proksimaaliset ylijäämät timantti- tai volframikarbidiviimeistelijöillä, viimeistelystripseillä tai taipuisilla viimeistelykiekoilla. Tarkasta purenta ja sivuliikkeet, ja hio tarvittavat korjaukset estääksesi varhaiskontaktit tai korotukset sivuliikkeissä täytteen pinnalla. Kiillota täyte silikonikiillottimilla (esim. Astropol® P, Astropol® HP, Astrobrush tai OptraPol® Next Generation) sekä kiillotuskiekoilla ja kiillotusstripseillä.

Lisätietoja
1. Mikäli välttämätöntä, IPS Empress Direct -materiaalia voidaan lisätä suoraan polymeroidun materiaalin päälle. Jos IPS Empress Direct -täyte on jo kiillotettu, pinta tulee ensin karhentaa ja kostuttaa Heliobond®-valmisteella ennen tuoreen IPS Empress Directin käyttöä.
2. IPS Empress Directin tulee olla huoneenlämpöistä sitä käytettäessä. Jäähtynyttä materiaalia on vaikea pursottaa.
3. Ⓧ Vain kertakäyttöön. Jos IPS Empress Direct annostellaan Cavifil-kärjestä suoraan potilaan suuhun, suosittelemme käyttämään Cavifil-kärkiä kertakäyttöisinä hygieenisistä syistä (potilaiden välisen ristikkäiskontaminaation välttäminen).
4. Älä desinfioi ruiskuja ja Cavifil-kärkiä hapettavilla desinfiointiaineilla.

Varoitus
Vältä kovettumattoman IPS Empress Direct -materiaalin joutumista kosketukseen ihon, limakalvojen ja silmien kanssa. Polymeroimaton IPS Empress Direct voi aiheuttaa lievää ärsytystä ja johtaa metakrylaattiyliherkkyyteen. Kaupallisesti saatavat, lääketieteelliseen käyttöön tarkoitetut käsineet eivät suojaa metakrylaattien herkistävältä vaikutukselta.

Säilytys
– Säilytyslämpötila: 2–28 °C.
– Sulje ruiskut/Cavifil-kärjet välittömästi käytön jälkeen. Altistuminen valolle aiheuttaa ennenaikaisen polymeroinnin.
– Älä käytä IPS Empress Directiä viimeisen käyttöpäivän jälkeen.
– Säilyvyysaika: Katso Cavifil-kärjessä, ruiskussa ja pakkauksessa olevat tiedot.

Säilytä materiaali poissa lasten ulottuvilta!
Vain hammaslääketieteelliseen käyttöön!

Tämä materiaali on tarkoitettu ainoastaan hammaslääketieteelliseen käyttöön. Materiaalia tulee käsitellä tarkasti käyttöohjeita noudattaen. Valmistaja ei vastaa vahingoista, jotka johtuvat siitä, että käyttöohjeita tai ohjeidenmukaista soveltamisalaa ei noudateta. Tuotteiden soveltuvuuden testaaminen tai käyttäminen muuhun kuin ohjeissa mainittuun tarkoitukseen on käyttäjän vastuulla. Kuvaukset ja tiedot eivät ole takuu ominaisuuksista eivätkä ole sitovia.

Norsk

Beskrivelse
IPS Empress® Direct er en lysherdende, røntgengjennomskinnelig, nanohybrid kompositt basert på toppmoderne teknologi, som er egnet til å lage restaureringer som oppfyller de høyeste kravene. IPS Empress Direct herdes med lys i bølgelengdeområdet 400–500 nm (blått lys).

Farger
IPS Empress Direct tilbys i 32 forskjellige farger og 5 forskjellige grader av gjennomskinnelighet.
Følgende farger er tilgjengelige:
Emaljefarger
A1, A2, A3, A3.5, A4, B1, B2, B3, B4, C1, C2, C3, D2, D3, Bleach-L, Bleach-XL
Dentinfarger
A1, A2, A3, A3.5, A4, IVA5, IVA6, B1, B2, C3, D2, Bleach-L, Bleach-XL
Gjennomskinnelige farger
Trans 20, Trans 30, Opal* *Ikke radiogjennomskinnelig på grunn av den høye gjennomsiktigheten

Sammensetning
Monomermatrisen består av dimetakrylater (20–21,5 vekt-%, opaliserende farge 17 vekt-%). Fyllstoffene inneholder bariumglass, ytterbiumtrifluorid, blandingsoksid, silikondioksid og kopolymer (77,5–79 vekt-%, opaliserende farge 83 vekt-%). I tillegg inneholder de additiver, stabilisatorer og pigmenter (< 1,0 vekt-%). Det totale innholdet av uorganiske fyllstoff er 75–79 vekt-% eller 52–79 vol-% (opaliserende farge 60,5 vekt-% eller 45 vol-%). Partikkelstørrelsen for de uorganiske fyllstoffene ligger mellom 40 nm og 3000 nm med en snittstørrelse på 550 nm.

Indikasjon
– Fortannsfyllinger (klasse III, IV)
– Fyllinger i posteriorområdet (klasse I og II)
– Tannhalsfyllinger (klasse V, f.eks. i tilfelle tannhalskaries, roterosjoner, kileformede defekter)
– Justering av tannstilling og tannform (for eksempel lukking av diastema, lukking av interdentale "svarte trekanter", forlengelse av incisalkanter)
– Direkte skallfasetter

Kontraindikasjon
Plassering av IPS Empress Direct-fyllinger er kontraindisert
– hvis tilstrekkelig tørrlegging eller den foreskrevne anvendelsesteknikken ikke er mulig
– ved påvist allergi mot bestanddeler i IPS Empress Direct

Bivirkninger
Bestanddeler i IPS Empress Direct kan i sjeldne tilfeller føre til sensibilisering. I slike tilfeller må videre bruk av IPS Empress Direct unngås. For å unngå mulig irritasjon av pulpa skal pulpanære områder forsynes med en egnet pulpa-/dentinbeskyttelse (pulpanært påføres selektivt et kalsiumhydroksidholdig preparat som dekkes med en egnet kavitetsfôring).

Interaksjoner
Materialer som inneholder eugenol-/nellikolje, hemmer polymeriseringen av komposittmaterialer. Slike materialer skal derfor ikke brukes sammen med IPS Empress Direct. I kontakt med kationisk munnvann samt ved plakkindikatorer og klorheksidin kan det oppstå misfarginger.

Bruksområde
1. Fargebestemmelse
Før bestemmelse av farge skal tennene renses. Emaljefargen skal bestemmes mens tannen er fuktig. Dentinfargen kan bestemmes enten ved å plassere fliken ved siden av den preparerte tannen eller ved siden av det cervikale området på hjørnetannen.
2. Tørrlegging
Tilstrekkelig tørrlegging, helst med kofferdam (f.eks. OptraDam® Plus), er nødvendig.
3. Forberedelse av kaviteten

Preparering av kaviteten skal gjøres etter reglene for adhesivteknikken, dvs. slik at tannsubstansen skånes. Skrå emaljekantene i fortannsområdet. I posteriorområdet skal bare de skarpe emaljekantene avrundes (pussediamanter, 25–40 µm). Kariesfrie tannhalsdefekter skal ikke prepareres, bare renses med pimpstein eller en passende pussepasta samt gummikopp eller en roterende børste. I tilfeller av sklerotisk dentin kan overfladisk rekondisjonering med rosefres forbedre heftforankringen. Deretter fjernes alle rester i kaviteten med vannspray. Kaviteten tørkes med vann- og oljefri luft.

4. Pulpabeskyttelse/fôring

Ved bruk av emalje-dentin-forankring skal det ikke brukes fôring. Bare ved svært dype, pulpanære kaviteter skal dette området tildekkes med et kalsiumhydroksidmateriale (f.eks. ApexCal®) og deretter legges et lag med trykkstabil sement over (f.eks. glassionomersement som Vivaglass® Liner). De andre kavitetsveggene skal ikke dekkes til, slik at de kan brukes til forankring med et emalje-dentin-klebemiddel.

5. Plassering av matrise/interdentalkile

Ved kaviteter med approksimal andel skal det enten brukes en sirkulær matrise eller en delmatrise og deretter kiler.

6. Klargjøring/påføring av forankringsmiddel

Klargjør tannstrukturen, og påfør forankringsmiddelet i samsvar med bruksanvisningen til produktet som brukes. Vi anbefaler bruk av Syntac® eller ExciTE® F (med fosforsyreetsing) eller AdheSE® eller AdheSE® One F selvetsende klebemiddel.

7. Påføring av IPS Empress Direct

– Ettersom et stort utvalg av dentin og emaljefarger med forskjellige gjennomskinneligheter er tilgjengelige ved bruk av IPS Empress Direct til utforming av anatomiske detaljer, kan en hvilken som helst lagbyggende teknikk brukes kreativt for å oppnå svært estetiske restaureringer.
  For å få et optimalt resultat skal IPS Empress Direct påføres i lagtykkelser på maks 2 mm (eller 1,5 mm for dentinfarger) og tilpasses med f.eks. OptraSculpt®/OptraSculpt® Pad.
– Tilstrekkelig belysning forhindrer ufullstendig polymerisering. Anbefalinger for belysningsvarighet (Exposure Time) og lysintensitet (Light Intensity), se tabell 1. Hold vinduet som utstråler lys, så nært overflaten til restaureringsmaterialet som mulig.
– Ved bruk av en metallmatrise må det, etter at matrisen er fjernet, i tillegg utføres bukkal henholdsvis lingual/palatinal belysning hvis det ikke ble brukt Bluephase®-herdelys eller hvis lysproben ikke kunne plasseres på en ideell måte (f.eks. langt unna materialet eller med divergerende stråling).
– I mange tilfeller blir det nå brukt en flytende kompositt for det første sjiktet for å skape en jevn kavitetsbunn og samtidig gjøre det lettere å tilpasse restaureringsmaterialet som brukes etterpå. Bruken av flytende kompositt (f.eks. Tetric EvoFlow®) som et tynt første lag, er valgfritt. Dette laget må lysherdes i et eget trinn (instruksjonene fra de respektive bruksanvisningene må følges).

8. Overflatebehandling/okklusjonskontroll/polering

Etter polymeriseringen skal overskuddet fjernes med egnet overflatebehandlingsutstyr (f.eks. Astropol® F) eller finkornede diamanter etter polymerisering. Approksimale overskudd fjernes med diamant-, hardmetall-finerbor, finerstrips eller fleksible finerskiver. Kontroller okklusjon og artikulasjon og utfør egnet slipekorreksjoner for å hindre for tidlig kontakt og uønskede artikulasjonsveier på overflaten av fyllingene. Høyglanspolering av fyllinger gjøres med silikonpolerere (f.eks. Astropol® P, Astropol® HP, Astrobrush eller OptraPol® Next Generation) samt polerskiver og polerstrips.

Tilleggsinformasjon

1. Ekstra IPS Empress Direct-materiale kan påføres direkte på det polymeriserte materialet om nødvendig. Hvis IPS Empress Direct-fyllingen allerede er polert, må den først gjøres ru og fuktes med Heliobond® før ny IPS Empress Direct kan påføres.
2. IPS Empress Direct skal ha romtemperatur når det påføres. Ved kjøleskapstemperatur kan den være vanskelig å presse ut.
3. Kun til engangsbruk. Hvis IPS Empress Direct påføres fra Cavifil direkte inn i munnen på pasienten, må Cavifil kastes etter bruk for å forhindre krysskontaminering mellom pasienter.
4. Sprøyter eller Cavifils skal ikke desinfiseres med oksiderende desinfeksjonsmidler.

Advarsel

Unngå kontakt mellom uherdet IPS Empress Direct og huden/slimhinnene og øynene. IPS Empress Direct i uherdet tilstand kan virke irriterende og kan føre til overfølsomhet mot metakrylater. Vanlige medisinske hansker gir ingen beskyttelse mot sensitivitet mot metakrylater.

Oppbevaring

– Oppbevaringstemperatur 2–28 °C.
– Sprøyter/Cavifils skal lukkes umiddelbart etter bruk. Lys fører til for tidlig polymerisering.
– Bruk ikke IPS Empress Direct etter utløpsdato.
– Holdbarhet: se dato på Cavifil, sprøyte eller emballasje.

Oppbevar materialet utilgjengelig for barn!
Bare til dental bruk.

Materialet er utviklet til bruk kun på det odontologiske området. Arbeid med produktet skal utføres strengt i samsvar med bruksanvisningen. Produsenten påtar seg intet ansvar for skader som oppstår på grunn av annen bruk eller ufagmessig bearbeiding. I tillegg er brukeren forpliktet til på forhånd og på eget ansvar å undersøke om produktene egner seg og kan brukes til de tiltenkte formål, særlig dersom disse formålene ikke er oppført i bruksanvisningen. Beskrivelser og data utgjør ingen garanti for egenskaper og er ikke bindende.

Nederlands

Omschrijving

IPS Empress® Direct is een lichtuithardend, röntgenopaak, nanohybride composietmateriaal voor restauraties die aan de hoogste eisen moeten voldoen. Het materiaal is ontwikkeld volgens de modernste technologische inzichten. IPS Empress Direct hardt uit onder invloed van licht met een golflengte van 400 à 500 nm (blauw licht).

Kleurtinten

IPS Empress Direct is verkrijgbaar in 32 verschillende kleurtinten en in 5 verschillende translucentiegradaties.
De volgende kleurtinten zijn verkrijgbaar:

Glazuurkleuren

A1, A2, A3, A3.5, A4, B1, B2, B3, B4, C1, C2, C3, D2, D3, Bleach-L, Bleach-XL

Dentinekleuren

A1, A2, A3, A3.5, A4, IVA5, IVA6, B1, B2, C3, D2, Bleach-L, Bleach-XL

Translucente kleurtinten

Trans 20, Trans 30, Opal* *Door de sterke translucentie niet röntgenopaak

Samenstelling
De monomeermatrix bestaat uit dimethacrylaten (20-21,5 gewichtsprocent, opalescente kleurtint 17 gewichtsprocent). De vulstoffen zijn bariumglas, ytterbiumtrifluoride, mengoxide, siliciumdioxide en copolymeer (77,5-79 gewichtsprocent, opalescente kleurtint 83 gewichtsprocent). Verdere bestanddelen zijn additieven, katalysatoren, stabilisatoren en pigmenten (< 1,0 gewichtsprocent). Het totale gehalte aan anorganische vulstoffen is 75-79 gewichtsprocent of 52-59 volumeprocent (opalescente kleurtint 60,5 gewichtsprocent of 45 volumeprocent). De deeltjesgrootte van de anorganische vulstoffen ligt tussen de 40 nm en 3000 nm, met een gemiddelde deeltjesgrootte van 550 nm.

Indicatie
– Restauraties in het anteriorgebied (klasse III, IV)
– Restauraties in het posteriorgebied (klasse I en II)
– Tandhalsvullingen (klasse V, bijv. bij tandhalscariës, wortelerosie, wigvormige defecten)
– Correctie van de positie of vorm van gebitselementen (bijv. sluiten van diastemen, sluiten van interdentale driehoeken, verlenging van de incisale randen)
– Directe veneers

Contra-indicaties
Het aanbrengen van restauraties van IPS Empress Direct is gecontra-indiceerd
– bij onvoldoende drooglegging van het werkgebied, of wanneer de voorgeschreven toepassingstechniek niet mogelijk is
– bij bekende allergie van de patiënt voor een van de bestanddelen van IPS Empress Direct

Bijwerkingen
In uitzonderlijke gevallen kunnen bestanddelen van IPS Empress Direct tot overgevoeligheid leiden. In die gevallen mag IPS Empress Direct niet meer worden gebruikt. Om mogelijke irritatie van de pulpa tegen te gaan, moeten gebieden in de buurt van de pulpa worden behandeld met een geschikt pulpa-/dentinebeschermingsmateriaal (breng in de buurt van de pulpa selectief een calciumhydroxidehoudend preparaat aan en dek de preparatie af met een geschikte onderlaag).

Wisselwerkingen
Materialen die eugenol-/kruidnagelolie bevatten, kunnen de polymerisatie van composietmaterialen afremmen. Daarom moet van applicatie van dergelijke materialen in combinatie met IPS Empress Direct worden afgezien. Kationische mondspoelmiddelen, plaquekleurstoffen en chloorhexidine kunnen bij contact met het materiaal tot verkleuringen leiden.

Toepassing
1. Kleurbepaling
Reinig het gebit voor u de kleur bepaalt. Voer de kleurbepaling van de glazuurtint uit aan de hand van de kleur van het nog vochtige gebit. De dentinekleurtint kan direct aan de hand van het geprepareerde element worden bepaald of cervicaal bij de hoektand.
2. Isolatie
Zorg voor goede isolatie van het werkgebied, liefst met een cofferdam (bijv. OptraDam® Plus).
3. Voorbereiding van de caviteit
Prepareer de caviteit volgens de regels van de adhesieftechniek. Dat wil zeggen dat het gebitsweefsel zoveel mogelijk ongemoeid wordt gelaten. Glazuurranden in het anteriorgebied moeten schuin worden afgeslepen. In het posteriorgebied moeten alleen de scherpe glazuurranden worden afgerond (fineerdiamanten, 25-40 µm). Cariësvrije defecten van de tandhals worden niet geprepareerd, maar slechts gereinigd met een reinigingspasta zoals puimsteen, een rubbercupje of een roterend borsteltje. In het geval van sclerotisch dentine kan oppervlakkig herconditioneren met een rozenboor de hechting verbeteren. Spoel de caviteit vervolgens schoon met water om alle restanten te verwijderen. Blaas de caviteit droog met water- en olievrije lucht.
4. Pulpabescherming/onderlaag
Pas geen onderlaag toe als er een glazuur-dentinehechtmiddel wordt gebruikt. Dek zeer diepe gebieden dichtbij de pulpa selectief af met een calciumhydroxidemateriaal (bijv. ApexCal®) en gebruik vervolgens een drukbestendig cement (bijv. glasionomeercement, zoals Vivaglass® Liner). Dek de overige wanden van de caviteit niet af, zodat ze nog te gebruiken zijn voor het toepassen van een glazuur-dentineadhesief.
5. Aanbrengen van een matrixband/interdentale wig
Breng bij (gedeeltelijk) approximale caviteiten een circulaire matrixband of een partiële matrixband aan en zet deze vast met wiggen.
6. Conditionering/aanbrengen van het hechtmiddel
Conditioneer het gebitsweefsel en breng het hechtmiddel aan zoals beschreven staat in de gebruiksaanwijzing van het toegepaste product. Ivoclar Vivadent raadt aan de adhesieven Syntac® of ExciTE® F (met fosforzuuretsen) te gebruiken of de zelfetsende adhesieven AdheSE® of AdheSE® One F.
7. Aanbrengen van IPS Empress Direct
– Aangezien er bij IPS Empress Direct sprake is van een groot aantal verkrijgbare dentine- en glazuurkleurtinten in verschillende transparantiegradaties en er bovendien translucente kleurtinten beschikbaar zijn voor het maken van anatomische details, kan met iedere laagtechniek creatief worden toegewerkt naar zeer esthetische restauraties. Voor een optimaal resultaat moet IPS Empress Direct worden aangebracht in laagdikten van max. 2 mm resp. 1,5 mm (dentinekleurtinten) en vervolgens worden geadapteerd met bijv. OptraSculpt®/OptraSculpt® Pad.
– Door voldoende belichting wordt onvolledige polymerisatie voorkomen. Zie voor adviezen over de belichtingstijd (exposure time) en lichtintensiteit (light intensity) tabel 1. Zorg dat u het lichtemissievenster van de polymerisatielamp zo dicht mogelijk op het restauratiemateriaal houdt.
– Belicht de restauratie na het verwijderen van de eventuele metalen matrixband eveneens van buccaal resp. linguaal/palatinaal. Dit is vooral noodzakelijk als er geen Bluephase®-polymerisatielamp wordt gebruikt of als de lichtgeleider niet in een ideale positie kan worden gebracht (qua afstand tot de composiet of de hoek voor de juiste lichtspreiding).
– In veel gevallen wordt er nu een vloeibare composiet gebruikt als eerste laag. Daarmee wordt de bodem van de caviteit vlak en kan het verder gebruikte restauratiemateriaal gemakkelijker worden geadapteerd. Er kan als eerste, dunne laag een vloeibaar composietmateriaal (bijv. Tetric EvoFlow®) worden gebruikt. Deze laag moet met licht worden uitgehard in een aparte stap (raadpleeg de instructies in de betreffende gebruiksaanwijzing).
8. Afwerking/occlusiecontrole/polijsten

Verwijder overtollig materiaal na polymerisatie met behulp van de juiste fineerinstrumenten (bijv. Astropol® F) of fijne diamanten. Verwijder proximaal overtollig materiaal met fineerinstrumenten van diamant of wolfraamcarbide, fineerstrips of flexibele fineerschijfjes. Controleer de occlusie en articulatie en slijp deze zo in dat er geen premature contacten of ongewenste articulatiebanen aan het oppervlak van de restauratie achterblijven. Polijst de restauraties blijvend op hoogglans met behulp van siliconenpolijstinstrumenten (bijv. Astropol® P, Astropol® HP, Astrobrush of OptraPol® Next Generation) en polijstschijfjes en polijststrips.

Overige informatie
1. Indien nodig kan aanvullend IPS Empress Direct-materiaal direct worden aangebracht op reeds gepolymeriseerd materiaal. Als de restauratie van IPS Empress Direct reeds is gepolijst, moet hij eerst worden opgeruwd en vervolgens worden bevochtigd met Heliobond®, voor er een nieuwe laag IPS Empress Direct kan worden aangebracht.
2. IPS Empress Direct moet bij verwerking op kamertemperatuur zijn. Wanneer het materiaal nog gekoeld is, kan het doseren moeilijk zijn.
3. Alleen voor éénmalig gebruik. Als IPS Empress Direct direct vanuit de Cavifil in de mond van de patiënt wordt geapplicееrd, moet de Cavifil na gebruik worden weggegooid, om kruisbesmetting tussen patiënten te voorkomen.
4. Gebruik geen oxiderende desinfectiemiddelen om spuiten of Cavifils te ontsmetten.

Waarschuwing
Voorkom contact van niet uitgehard IPS Empress Direct met de huid, de slijmvliezen of de ogen. Niet-gepolymeriseerd IPS Empress Direct kan een licht irriterende werking hebben en kan tot overgevoeligheid voor methacrylaten leiden. In de handel verkrijgbare medische handschoenen bieden geen bescherming tegen overgevoeligheid voor methacrylaten.

Bewaren
– Bewaartemperatuur: 2–28°C.
– Sluit spuiten/Cavifils direct af na gebruik. Blootstelling aan licht kan tot voortijdige polymerisatie leiden.
– Gebruik IPS Empress Direct niet na afloop van de vervaldatum.
– Vervaldatum: zie informatie op de Cavifils, spuiten en verpakkingen.

Bewaar het materiaal buiten bereik van kinderen.
Alleen voor tandheelkundig gebruik.

Het materiaal is uitsluitend ontwikkeld voor tandheelkundig gebruik. Het moet precies volgens de gebruiksaanwijzing worden verwerkt. Indien er schade optreedt door toepassing voor andere doeleinden of door verkeerd gebruik, kan de fabrikant daarvoor niet aansprakelijk worden gesteld. De gebruiker is bovendien verplicht om vóór gebruik na te gaan of de producten voor de beoogde toepassing geschikt zijn, vooral als deze toepassing niet expliciet in de gebruiksaanwijzing vermeld staat. De beschrijvingen en gegevens houden geen garantie in ten aanzien van de eigenschappen en zijn niet bindend.

Ελληνικά

Περιγραφή
Το IPS Empress® Direct είναι μια φωτοπολυμεριζόμενη, ακτινοσκιερή, νανο-υβριδική σύνθετη ρητίνη βασισμένη στην τελευταία λέξη της τεχνολογίας, η οποία είναι κατάλληλη για την κατασκευή αποκαταστάσεων που ικανοποιούν και τις πιο υψηλές απαιτήσεις. Το IPS Empress Direct πολυμερίζεται με φως μήκους κύματος 400–500 nm (μπλε φως).

Αποχρώσεις
Το IPS Empress Direct διατίθεται σε 32 διαφορετικές αποχρώσεις και 5 διαφορετικούς βαθμούς φωτοδιαπερατότητας. Διατίθενται οι ακόλουθες αποχρώσεις:
Αποχρώσεις αδαμαντίνης
A1, A2, A3, A3.5, A4, B1, B2, B3, B4, C1, C2, C3, D2, D3, Bleach-L, Bleach-XL
Αποχρώσεις οδοντίνης
A1, A2, A3, A3.5, A4, IVA5, IVA6, B1, B2, C3, D2, Bleach-L, Bleach-XL
Αποχρώσεις με υψηλή φωτοδιαπερατότητα
Trans 20, Trans 30, Opal*

*Μη ακτινοσκιερό λόγω της υψηλής του διαφάνειας

Σύνθεση
Η μονομερής μήτρα αποτελείται από διμεθακρυλικά (20–21,5 % κ.β., οπαλίζουσα απόχρωση 17 % κ.β.). Οι ανόργανες ενισχυτικές ουσίες περιλαμβάνουν βαριούχο ύαλο, τριφθορίδιο του υττερβίου, αναμεμιγμένα οξείδια, διοξείδιο του πυριτίου και συμπολυμερή (77,5–79 % κ.β., οπαλίζουσα απόχρωση 83 % κ.β.). Επιπλέον περιέχει πρόσθετα, καταλύτες, σταθεροποιητές και χρωστικές (<1,0 % κ.β.). Η συνολική περιεκτικότητα σε ανόργανες ενισχυτικές ουσίες είναι 75–79 % κ.β. ή 52–59 % κ.ό. (οπαλίζουσα απόχρωση 60,5 % κ.β. ή 45 % κ.ό.). Το μέγεθος των ενισχυτικών κόκκων κυμαίνεται από 40 nm έως 3.000 nm με μέσο μέγεθος κόκκων 550 nm.

Ενδείξεις
– Αποκαταστάσεις προσθίων (Ομάδες III, IV)
– Αποκαταστάσεις οπισθίων (Ομάδες I και II)
– Αυχενικές αποκαταστάσεις (Ομάδα V, π.χ. στην περίπτωση αυχενικών τερηδόνων, διάβρωσης ρίζας, σφηνοειδών βλαβών)
– Διορθώσεις θέσης και σχήματος δοντιών (π.χ. κλείσιμο διαστήματος, κλείσιμο μεσοδόντιων "μαύρων τριγώνων", επιμήκυνση του κοπτικού άκρου)
– Άμεσες όψεις

Αντενδείξεις
Η εφαρμογή του IPS Empress Direct αντενδείκνυται όταν:
– δεν είναι δυνατόν να εξασφαλιστεί στεγνό εγχειρητικό πεδίο ή εάν δεν μπορεί να εφαρμοστεί η ενδεδειγμένη τεχνική εργασίας
– είναι γνωστό ότι ο ασθενής είναι αλλεργικός σε κάποιο από τα συστατικά του IPS Empress Direct

Παρενέργειες
Σε σπάνιες περιπτώσεις, συστατικά του IPS Empress Direct είναι δυνατόν να προκαλέσουν ευαισθησίες. Σε τέτοιες περιπτώσεις, δεν πρέπει να χρησιμοποιείται το IPS Empress Direct. Για την αποφυγή πιθανών αντιδράσεων του πολφού, καλύπτουμε περιοχές κοντά σε αυτόν με κατάλληλους παράγοντες προστασίας πολφού/οδοντίνης (επιλεκτικά τοποθετήστε σκεύασμα υδροξειδίου του ασβεστίου σε επιφάνειες κοντά στον πολφό και επικαλύψτε με κατάλληλο liner).

Αλληλεπιδράσεις
Σκευάσματα που περιέχουν ευγενόλη/γαρυφαλέλαιο είναι δυνατόν να αναχαιτίσουν τον πολυμερισμό των σύνθετων ρητινών. Κατά συνέπεια, θα πρέπει να αποφεύγεται η χρήση τέτοιων σκευασμάτων με το IPS Empress Direct. Επαφή με κατιονικά στοματοπλύματα, με παράγοντες αποκάλυψης πλάκας και με χλωρεξιδίνη είναι δυνατόν να προκαλέσει δυσχρωμίες.

Εφαρμογή
1. Επιλογή απόχρωσης
Καθαρίστε τα δόντια πριν από την επιλογή της απόχρωσης. Για την επιλογή της απόχρωσης της αδαμαντίνης, τα δόντια θα πρέπει να παραμείνουν υγρά. Η απόχρωση της οδοντίνης μπορεί να καθοριστεί τοποθετώντας το δείγμα δίπλα είτε στο παρασκευασμένο δόντι είτε στην αυχενική περιοχή του κυνόδοντα.
2. Απομόνωση
Απαιτείται η κατάλληλη απομόνωση. Η χρήση ελαστικού απομονωτήρα είναι απαραίτητη (π.χ. OptraDam® Plus).
3. Παρασκευή κοιλότητας
Παρασκευάζετε την κοιλότητα σύμφωνα με τις αρχές της τεχνικής της συγκόλλησης, π.χ. διαφύλαξη υγιών οδοντικών ιστών. Λοξοτομήστε τις ακμές της αδαμαντίνης στην πρόσθια περιοχή. Στην περιοχή των οπισθίων, στρογγυλέψτε μόνο τις οξύαιχμες ακμές της αδαμαντίνης (με διαμάντια φινιρίσματος, 25–40 µm). Οι αυχενικές βλάβες χωρίς τερηδόνα δεν παρασκευάζονται. Καθαρίστε μόνο με ελαφρόπετρα ή άλλη κατάλληλη πάστα καθαρισμού με τη βοήθεια ελαστικών κυπελλοειδών ή περιστροφικών βουρτσών. Στην περίπτωση σκληρωτικής οδοντίνης, η επιφανειακή επαναπροετοιμασία με φρέζα μπορεί να βελτιώσει το συγκολλητικό δεσμό. Στη συνέχεια, απομακρύνετε όλες τις περίσσειες από την κοιλότητα με καταιονισμό νερού. Στεγνώστε την κοιλότητα με ξηρό αέρα, ελεύθερο από νερό και ελαιώδεις ουσίες.
4. Προστασία του πολφού / Ουδέτερο στρώμα
Μην τοποθετείτε ουδέτερο στρώμα όταν χρησιμοποιείτε συγκολλητικό παράγοντα αδαμανίνης/οδοντίνης. Μόνο σε πολύ βαθιές κοιλότητες, καλύψτε τις επιφάνειες που βρίσκονται κοντά στον πολφό με λεπτό στρώμα υδροξειδίου του ασβεστίου (π.χ. ApexCal®) και έπειτα καλύψτε με στρώμα κονίας για απορρόφηση των δυνάμεων πίεσης (π.χ. υαλοϊονομερή κονία όπως η Vivaglass® Liner). Μην καλύψετε τις υπόλοιπες επιφάνειες της κοιλότητας, γιατί θα χρησιμοποιηθούν στη δημιουργία δεσμού με τον συγκολλητικό παράγοντα οδοντίνης και αδαμαντίνης.
5. Τοποθέτηση τεχνητού τοιχώματος / μεσοδόντιας σφήνας
Σε κοιλότητες που περιλαμβάνουν όμορες περιοχές, χρησιμοποιήστε τεχνητό τοίχωμα που τυλίγεται γύρω από το δόντι ή τμηματικό τεχνητό τοίχωμα. Στη συνέχεια, τοποθετήστε σφήνα.
6. Τροποποίηση αδαμαντίνης και οδοντίνης /
Εφαρμογή του συγκολλητικού παράγοντα
Τροποποιήστε τη δομή του δοντιού και τοποθετήστε συγκολλητικό παράγοντα σύμφωνα με τις οδηγίες του κατασκευαστή του υλικού. Συστήνουμε τη χρήση του συγκολλητικού Syntac® ή ExciTE® F (με αδροποίηση ορθοφωσφορικού οξέος) ή του συγκολλητικού παράγοντα με ενεργοποιητές αδροποίησης AdheSE® ή AdheSE® One F (self-etching).
7. Τοποθέτηση του IPS Empress Direct
– Δεδομένου ότι διατίθεται ένας μεγάλος αριθμός αποχρώσεων IPS Empress Direct Dentin (οδοντίνης) και Enamel (αδαμαντίνης) σε διαφορετικούς βαθμούς φωτοδιαπερατότητας για τη δημιουργία ανατομικής λεπτομέρειας, μπορεί να χρησιμοποιηθεί δημιουργικά οποιαδήποτε τεχνική με στρώματα για την επίτευξη αισθητικών αποκαταστάσεων. Για ιδανικά αποτελέσματα, τοποθετήστε το IPS Empress Direct σε στρώματα πάχους μέχρι 2 χιλ. (ή 1,5 χιλ. για αποχρώσεις οδοντίνης) και προσαρμόστε με χρήση κατάλληλου εργαλείου (π.χ. OptraSculpt®/OptraSculpt® Pad.
– Επαρκής έκθεση στο φως πολυμερισμού αποτρέπει τον ελλιπή πολυμερισμό. Για συστάσεις σχετικά με το χρόνο έκθεσης (Exposure Time) και τη φωτεινή ένταση (Light Intensity), βλ. Πίνακα 1. Κρατάτε το παράθυρο μετάδοσης φωτός όσο το δυνατόν πιο κοντά στην επιφάνεια του αποκαταστατικού υλικού.
– Εάν έχετε χρησιμοποιήσει μεταλλικό τεχνητό τοίχωμα, πολυμερίστε επιπροσθέτως την αποκατάσταση από την παρειακή ή γλωσσική/υπερώια πλευρά μόλις αφαιρέσετε το τοίχωμα, εάν δεν χρησιμοποιήθηκε λυχνία πολυμερισμού Bluephase® ή εάν το ρύγχος φωτός δεν ήταν δυνατόν να τοποθετηθεί σε ιδανική θέση (π.χ. μακριά από το υλικό ή με παρεκκλίνουσα ακτινοβολία).
– Σε πολλές περιπτώσεις, χρησιμοποιείται σήμερα μια λεπτόρρευστη σύνθετη ρητίνη για το αρχικό στρώμα προκειμένου να δημιουργηθεί μια ομοιόμορφη κοίτη κοιλότητας και να απλουστευθεί η προσαρμογή του υλικού αποκατάστασης που χρησιμοποιείται στη συνέχεια. Η χρήση μιας λεπτόρρευστης σύνθετης ρητίνης (π.χ. Tetric EvoFlow®) ως αρχικό λεπτό στρώμα είναι προαιρετική. Αυτό το στρώμα πρέπει να φωτοπολυμερίζεται σε ένα ξεχωριστό βήμα (πρέπει να τηρούνται οι οδηγίες που δίνονται στις αντίστοιχες Οδηγίες χρήσης).
8. Φινίρισμα / Έλεγχος της σύγκλεισης / Στίλβωση
Μετά τον πολυμερισμό, απομακρύνετε τις περίσσειες με τα κατάλληλα λαστιχάκια (π.χ. Astropol® F) ή με λεπτόκοκκο διαμάντι. Απομακρύνετε όμορες περίσσειες με διαμάντι, φρέζες καρβιδίου βολφραμίου, ταινίες λείανσης ή εύκαμπτους δίσκους λείανσης. Ελέγξτε τη σύγκλειση και την άρθρωση και διορθώστε με τρόχισμα εάν χρειάζεται για να αποφύγετε πρόωρες επαφές ή ανεπιθύμητες συγκλεισιακές παρεμβολές στην επιφάνεια των αποκαταστάσεων. Χρησιμοποιήστε σιλικονούχα λαστιχάκια (π.χ. Astropol® P, Astropol® HP, Astrobrush ή OptraPol® Next Generation) καθώς και δίσκους και ταινίες στίλβωσης για να αποδώσετε στίλβωση υψηλού βαθμού.

Πρόσθετες πληροφορίες
1. Μπορείτε να τοποθετήσετε νέο στρώμα IPS Empress Direct σε ήδη πολυμερισμένο, εάν χρειαστεί. Εάν η αποκατάσταση IPS Empress Direct είναι ήδη στιλβωμένη, θα πρέπει πρώτα να τροχίσετε την επιφάνεια και να την διαβρέξετε με Heliobond® πριν τοποθετήσετε τη νέα ποσότητα IPS Empress Direct.
2. Το IPS Empress Direct πρέπει να χρησιμοποιείται σε θερμοκρασία δωματίου. Κρύο υλικό ίσως είναι δύσκολο να εξαχθεί από τη σύριγγα.
3. Για μία χρήση μόνο. Εάν το IPS Empress Direct εφαρμόζεται με μικροσύριγγες Cavifil απευθείας μέσα στο στόμα του ασθενή, συστήνουμε, για λόγους υγιεινής, τη χρήση των Cavifil μία μόνο φορά (πρόληψη της μετάδοσης μόλυνσης μεταξύ των ασθενών).
4. Οι σύριγγες ή τα Cavifil δεν πρέπει να απολυμαίνονται με οξειδωτικούς παράγοντες απολύμανσης.

Προειδοποίηση
Απολυμέριστο IPS Empress Direct δεν πρέπει να έρθει σε επαφή με δέρμα, βλεννογόνους και μάτια. Απολυμέριστο IPS Empress Direct μπορεί να προκαλέσει ερεθισμούς ελαφράς μορφής, με αποτέλεσμα ευαισθησία στα μεθακρυλικά. Τα ιατρικά γάντια του εμπορίου δεν παρέχουν προστασία από την ευαισθησία στα μεθακρυλικά.

Αποθήκευση
– Θερμοκρασία αποθήκευσης 2–28 °C.
– Κλείνετε τις σύριγγες/Cavifil αμέσως μετά τη χρήση. Έκθεση στο φως προκαλεί πρόωρο πολυμερισμό.
– Μη χρησιμοποιείτε το IPS Empress Direct μετά την ημερομηνία λήξης.

– Διάρκεια ζωής: βλ. πληροφορίες επάνω στα Cavifil, στις σύριγγες και στις συσκευασίες.

Φυλάσσετε τα υλικά μακριά από παιδιά.
Μόνο για οδοντιατρική χρήση.

Το υλικό αυτό κατασκευάστηκε αποκλειστικά για οδοντιατρική χρήση. Οι διαδικασίες πρέπει να ακολουθούνται σχολαστικά και ακολουθώντας αυστηρά τις οδηγίες χρήσης. Απαιτήσεις για βλάβες που μπορεί να προκληθούν από μη ορθή ακολουθία των οδηγιών ή από χρήση σε μη ρητώς ενδεικνυόμενη περιοχή, είναι απαράδεκτες. Επιπλέον ο χρήστης είναι υποχρεωμένος να ελέγχει με δική του ευθύνη την καταλληλότητα και τη δυνατότητα χρήσης του προϊόντος για τον προβλεπόμενο σκοπό πριν την εφαρμογή, ιδιαίτερα εάν ο συγκεκριμένος σκοπός δεν αναφέρεται στις πληροφορίες χρήσης. Περιγραφές και στοιχεία δεν αποτελούν εγγύηση των ιδιοτήτων και δεν είναι δεσμευτικά.

Türkçe

Tanım
IPS Empress® Direct, en üst seviyede beklentileri karşılayabilecek restorasyonların yapımına uygun, teknoloji harikası,ışıkla sertleşen, radyopak,nanohibrit kompozit restoratif diş dolgu malzemesidir. IPS Empress Direct 400-500 nm arası ışık dalga boyunda polimerize olur (mavi ışık).

Renkler
IPS Empress Direct 32 farklı renk ve 5 farklı derecede translusenside mevcuttur.
Aşağıdaki renk tonlarında temin edilebilir:
Mine renkleri
A1, A2, A3, A3.5, A4, B1, B2, B3, B4, C1, C2, C3, D2, D3, Bleach-L, Bleach-XL
Dentin renkleri
A1, A2, A3, A3.5, A4, IVA5, IVA6, B1, B2, C3, D2, Bleach-L, Bleach-XL
Translusent renkler
Trans 20, Trans 30, Opal* *Yüksek şeffaflığı nedeniyle radyopak değildir

Bileşimi
Monomer matriks dimetakrilatlardan oluşur (%20-21,5 ağırlık olarak, opalesen renk %17). Doldurucular baryum glass, iterbiyum triflorid, karma oksit, silikon dioksit ve kopolimerler şeklindedir (%77,5-79 ağırlık olarak, opalesen renk %83). İlave katkılar: katkı maddeleri, katalizörler, stabilize edici ve pigmentlerdir (<1,0% ağırlık olarak). İnorganik doldurucuların toplam oranı ağırlık olarak %75-79 veya hacimsel olarak %52-59 (opalesen renk % 60,5 ağırlık olarak veya %45 hacimsel). İnorganik doldurucuların partikül büyüklüğü 40 nm ve 3000 nm arasında olup ortalama partikül büyüklüğü 550nm'dir.

Endikasyon
– Anterior restorasyonlar (Sınıf III, IV)
– Posterior bölgedeki restorasyonlar (Sınıf I ve II)
– Dişlerin boyun bölgelerindeki restorasyonlar (servikal çürükler, kök erozyonları, kama defektleri benzeri Sınıf V restorasyonlar)
– Diş pozisyonlarının ve şekillerinin düzeltilmesi (diastema kapatılması, interdental "siyah üçgenlerin" kapatılması, insizal ucun uzatılması benzeri işlemler)
– Direkt lamina vener uygulamaları

Kontrendikasyonları
IPS Empress Direct restorasyonların yapımı şu durumlarda kontrendikedir:
– Kuru bir çalışma ortamının sağlanamadığı veya belirtilen uygulama tekniğine uyulamadığı durumlarda
– Hastanın IPS Empress Direct'in içeriğindeki herhangi bir maddeye alerjisi biliniyorsa

Yan etkiler
Kişisel olgularda, IPS Empress Direct'in bileşenleri nadiren duyarlılığa sebep olabilir. Böyle bir durumda IPS Empress Direct kullanılmamalıdır. Pulpaya yakın bölgelerde, muhtemel bir iritasyona karşı pulpa kalsiyum hidroksit benzeri uygun bir pulpa/dentin koruyucusuyla korunmalıdır(kalsiyum hidroksit esaslı preparasyon sadece pulpaya yakın bölgeye uygulanmalı ve sadece o bölgede uygun bir kaide ile üstü kapatılmalıdır).

Etkileşimler
Öjenol veya karanfil yağı içerikli maddeler kompozit materyallerin polimerizasyonunu engelleyebilir. Dolayısıyla, bu gibi materyallerin IPS Empress Direct ile birlikte uygulanmasından kaçınılmalıdır. Katyonik ağız çalkalayıcılar, plak çözücü ajanlar ve klorheksidin renk değişikliğine neden olabilir.

Uygulama
1. Renk tespiti
Renk tespitinden önce dişleri temizleyin. Mine rengi alınırken diş yüzeyi nemli olmalıdır. Dentin rengi, prepare edilen dişten ya da kaninin servikal bölgesinden referans alarak belirlenebilir.
2. Yalıtım
Uygun bir izolasyon, tercihen OptraDam® Plus gibi bir rubber-dam kullanımı, önerilir.
3. Kavite preparasyonu
Kavite preparasyonu sağlam diş yapısının korunması gibi adeziv tekniğin gereksinimlerini karşılayacak şekilde yapılır. Anterior bölgede mine kenarlarına bizotaj yapılır. Posterior bölgede sadece keskin mine kenarları 25-40µm'lik elmas bitirme frezleri ile yuvarlatılır. Çürüksüz servikal defektlerde preparasyona gerek yoktur ancak yüzey pomza veya başka bir uygun temizleme pastasıyla lastik veya fırça kullanarak temizlenir. Sklerotik dentin durumunda, rose frez ile yapılan yüzeyel hazırlık adeziv bağın daha iyi olmasını sağlayabilir. Bunu takiben kavitedeki tüm artıklar su spreyi ile temizlenir. Kavite, yağsız ve susuz hava ile kurutulur.
4. Pulpa koruması / Kaide
Mine/dentin bonding ajanı kullanılıyorsa kaideye gerek yoktur. Sadece pulpaya yakın derin bölgelerde ApexCal® benzeri kalsiyum hidroksit esaslı bir pulpa koruyucu, üstüne de Vivaglass® Liner benzeri basınca dayanıklı bir cam ionomer kaide materyali konulmalıdır. Diğer kavite duvarları mine/dentin adezivinin bağlantısını kuvvetlendirmek için açık bırakılmalıdır.
5. Matriks yerleştirilmesi / interdental kama
Proksimal alanı etkileyen kaviteler için sarıcı bir matriks veya bölümlü bir matriks kullanın ve kamalayın.
6. Koşullandırma / Bağlayıcı ajan uygulaması
Pürüzlendirme ve bonding ajanının uygulanması işlemi üretici firmanın direktifleri doğrultusunda yapılmalıdır. Biz optimum sonuçlar için, Syntac® veya ExciTE® F (fosforik asit pürüzlendirmesiyle) veya kendiliğinden asitleyici etkisi olan(self-etch) AdheSE® veya AdheSE® One F kullanımını tavsiye ediyoruz.
7. IPS Empress Direct'in uygulanması
– Anatomik detayların yaratılması için çok sayıda mine ve dentin renkleri farklı traslusenside mevcuttur, mükemmel estetik restorasyonların yapımı için tüm tabakalama tekniklerinden yaratıcı şekilde yararlanılabilir. En iyi sonuç için, IPS Empress Direct, 2 mm veya dentin rengi için

1,5 mm'lik tabakalar halinde OptraSculpt®/OptraSculpt® Pad ile kaviteye adapte edilmelidir.
– Yeterli bir ışınlama süresi polimerizasyonun eksik gerçekleşmesini önler. Işınlama süresi (Exposure Time) ve ışın yoğunluğu (Light Intensity) ile ilgili tavsiyeler için Tablo 1'e bakın. Işık emisyonu penceresini, restoratif malzemenin yüzeyine mümkün olduğunca yakın tutun.
– Metal matrix kullanıldığında, eğer Bluephase® sertleştirme ışığı kullanılmadıysa ya da ışık probu optimum konumda kullanılamadıysa (ör. materyalden uzakta ya da ıraksak ışınım ile), matriks çıkarıldıktan sonra bukkal ve lingual/palatinal'den ilave polimerizasyon yapılmalıdır.
– Çoğu durumda artık düzgün bir kavite zemini oluşturmak ve daha sonra kullanılacak restoratif malzemenin adaptasyonunu kolaylaştırmak amacıyla ilk tabaka olarak bir akışkan kompozit kullanılmaktadır. İlk ince katman olarak bir akışkan kompozitin (ör. Tetric EvoFlow®) kullanılması isteğe bağlıdır. Bu tabaka, ayrı bir adımda ışıkla sertleştirilmelidir (ilgili Kullanım Talimatları'nda verilen talimatlara uyulmalıdır).

8. Bitirme / Oklüzyon kontrolü / Polisaj
Polimerizasyon sonrası fazlalıklar Astropol® F veya ince elmas bitirme frezleri ile uzaklaştırılır. Proksimal fazlalıklar elmas bitirme frezleri, tungsten karbid bitirme frezleri, ara yüz bitirme bantları veya esnek bitirme diskleri ile uzaklaştırılır. Okluzyon ve artikülasyon kontrol edilip, restorasyon üzerindeki prematür kontaklar ve istenmeyen artikülasyon yolları uygun şekilde düzeltilir. Restorasyona kalıcı ve parlak bir görünüm verebilmek için silikon parlatıcılar (örn. Astropol® P, Astropol® HP, Astrobrush ya da OptraPol® Next Generation), cila diskleri ve bantları kullanılmalıdır.

İlave bilgi
1. Gerekirse, ilave IPS Empress Direct polimerize olmuş materyalin üzerine direkt olarak uygulanabilir. Eğer IPS Empress Direct restorasyon cilalanmışsa, yeni bir tabaka IPS Empress Direct uygulamadan önce yüzey önce pürüzlendirilip sonra da Heliobond® ile ıslatılmalıdır.
2. IPS Empress Direct uygulanırken oda ısısında olmalıdır. Dolaptan çıkar çıkmaz kullanılırsa materyali sıkmak ve uygulamak güçleşebilir.
3. Sadece tek kullanımlıktır. Eğer IPS Empress Direct'in hasta ağzına uygulanmasında kavifil uygulama ucu tercih edilmiş ise, hijyenik nedenlerden ötürü bu kavifillerin tek kez kullanımı önerilir (hastalar arasında çapraz kontaminasyonu önlemek için).
4. şırınga ve kavifiller okside edici dezenfeksiyon ajanları ile dezenfekte edilmemelidir.

Uyarı
Polimerize olmamış halde IPS Empress Direct cilt, mukoz membranlar ve göze temas etmemelidir. Polimerize olmamış halde IPS Empress Direct hafif bir iritasyona ve metakrilatlara karşı duyarlılığa sebep olabilir. Ticari medikal eldivenler metakrilatların duyarlılık geliştirebilen etkilerine karşı koruyucu değildir.

Saklama koşulları
– Saklama sıcaklığı 2-28 °C.
– Ürünü kullandıktan sonra şırıngaları/Cavifil'leri derhal kapatın. Işığa maruz kalması zamanından önce polimerize olmasına neden olur.
– IPS Empress Direct belirtilen son kullanma tarihinden sonra kullanılmamalıdır.
– Raf ömrü: şırınga, kavifil ve paketlerin üzerine bakınız.

Materyali çocukların ulaşamayacağı bir yerde saklayın.
Sadece diş hekimliği kullanımı içindir.

Bu materyal sadece diş hekimliğinde kullanılmak üzere hazırlanmıştır. İşleme koyma sırasında Kullanma Talimatına katı olarak riayet edilmelidir. Belirlenen kullanım alanı ve Kullanım Talimatının izlenmediği durumlarda oluşacak hasarlardan sorumluluk kabul edilmeyecektir. Ürünleri Talimatta açıkça belirtilmemiş herhangi bir amaç için kullanım ve uygunluk açısından test etmekten kullanıcı sorumludur. Tanımlama ve bilgiler özellikler için garanti oluşturmayacağı gibi bir bağlayıcılığı da yoktur.

Slovensko

Opis
IPS Empress® Direct je svetlobno strjujoč, radionepreprusten, nanohibriden kompozit na osnovi vrhunske tehnologije, primeren za izdelovanje restavracij, ki izpolnjujejo najvišje zahteve. IPS Empress Direct se strjuje s svetlobo z valovno dolžino 400–500 nm (modra svetloba).

Odtenki
IPS Empress Direct je na voljo v 32 različnih odtenkih in 5 različnih stopnjah prosojnosti. Na voljo so naslednji odtenki:
Odtenki sklenine
A1, A2, A3, A3.5, A4, B1, B2, B3, B4, C1, C2, C3, D2, D3, Bleach-L, Bleach-XL
Odtenki zobovine
A1, A2, A3, A3.5, A4, IVA5, IVA6, B1, B2, C3, D2, Bleach-L, Bleach-XL
Prosojni odtenki
Trans 20, Trans 30, Opal*

*Ni radionepreprusten zaradi visoke prosojnosti.

Sestava
Monomerna matrica sestoji iz dimetakrilatov (20–21,5 ut. %, opalescentni odtenek 17 ut. %). Polnila vsebujejo barijevo steklo, iterbijev trifluorid, mešani oksid, silicijev dioksid in kopolimer (77,5–79 ut. %, opalescentni odtenek 83 ut. %). Dodatno vsebuje: aditive, katalizatorje, stabilizatorje in pigmente (< 1,0 ut. %). Skupna vsebnost anorganskih polnil je 75–79 ut. % ali 52–59 vol% (opalescentni odtenek 60,5 ut. % ali 45 vol%). Velikost delcev anorganskih polnil je med 40 nm in 3000 nm, povprečna velikost delcev pa je 550 nm.

Indikacije
– anteriorne restavracije (razreda III, IV)
– restavracije v posteriornem območju (razreda I in II)
– restavracije zobnih vratov (razreda V, na primer v primeru cervikalnega kariesa, erozije korenine, okvar v obliki zagozd)
– popravki položajev zob in oblik zob (npr. zaprtje diasteme, zaprtje medzobnih »črnih trikotnikov«, podaljšanje incizivnega robu)
– neposredne fasete

Kontraindikacije
Namestitev restavracij IPS Empress Direct je kontraindicirana:
– če ni mogoče zagotoviti suhega delovnega območja ali ni mogoče uporabiti predpisane tehnike,
– če je znano, da je bolnik alergičen na katero koli sestavino kompozita IPS Empress Direct.

Neželeni učinki
V redkih primerih lahko sestavine kompozita IPS Empress Direct povzročijo senzibilizacijo. V teh primerih kompozita IPS Empress Direct ne smete uporabljati. Da bi se izognili morebitnemu vnetju pulpe, je treba območja blizu

pulpe zaščititi z ustrezno zaščito za pulpo/zobovino (selektivno nanesite preparacijo na osnovi kalcijevega hidroksida na območja, ki so blizu pulpe, in prevlecite z ustrezno podlago).

Medsebojno delovanje
Snovi, ki vsebujejo evgenol/nageljnovo olje, lahko zavirajo polimerizacijo kompozitnih materialov. Zato se je treba izogibati uporabi takšnih materialov skupaj s kompozitom IPS Empress Direct. V kombinaciji s kationskimi ustnimi vodicami, sredstvi za obarvanje zobnih oblog in klorheksidinom lahko pride do razbarvanja.

Uporaba
1. Določitev odtenka
Preden določite odtenek, zobe očistite. Odtenek sklenine izberite, ko so zobje še vlažni. Odtenek zobovine lahko določite tako, da zavihek postavite poleg pripravljenega zoba ali poleg cervikalnega predela podočnika.
2. Izolacija
Potrebna je ustrezna izolacija, najbolje s koferdamom (npr. OptraDam® Plus).
3. Preparacija kavitete
Kaviteto pripravite v skladu z zahtevami adhezivne tehnike, tj. tako da zaščitite zobno strukturo. Robovi sklenine v anteriornem območju so poševni. V posteriornem območju je treba zgladiti samo ostre robove sklenine (diamanti za končno obdelavo, 25–40 µm). Cervikalnih okvar brez kariesa ne pripravite, ampak jih samo očistite s plovcem ali drugo ustrezno pasto za čiščenje s pomočjo gumijastih čašic ali rotacijskih ščetk. V primeru sklerotične zobovine lahko s površinsko ponovno pripravo z rožnatim svedrom izboljšate adhezivnost. Nato odstranite vse ostanke v kaviteti z vodno prho. Kaviteto osušite z zrakom, ki ne vsebuje vode in olj.
4. Zaščita pulpe/podlaganje
Če uporabljate vezivno sredstvo za sklenino/zobovino, ne nanašajte materiala za podlaganje. S kalcijevim hidroksidom prekrijte le zelo globoke predele v bližini pulpe (npr. ApexCal®) in nato uporabite cement, ki je odporen na tlak (npr. steklasti cement, kot je Vivaglass® Liner). Ne prekrivajte ostalih sten kavitete, saj jih lahko uporabite za podporo vezivnemu sredstvu za sklenino/zobovino.
5. Namestitev matrice/medzobne zagozde
Pri kavitetah na proksimalnih delih matrico ovijte ali uporabite lokalno matrico in jo zagozdite.
6. Kondicioniranje/nanos vezivnega sredstva
Kondicionirajte zobno strukturo in nanesite vezivno sredstvo v skladu z navodili za uporabo izdelka, ki ga uporabljate. Priporočamo vam adheziv Syntac® ali ExciTE® F (za jedkanje s fosforno kislino) ali AdheSE® ali samojedkajoči adheziv AdheSE® One F.
7. Nanos kompozita IPS Empress Direct
– Ker je na voljo veliko število odtenkov zobovine in sklenine IPS Empress Direct z različnimi stopnjami prosojnosti, s katerimi lahko oblikujete anatomske podrobnosti, lahko s katero koli tehniko premazovanja na ustvarjalen način poskrbite za zelo lepe restavracije. Za optimalen rezultat kompozit IPS Empress Direct nanesite v plasteh debeline največ 2 mm (ali 1,5 mm pri odtenkih zobovine), in jih prilagodite z npr. ploščico OptraSculpt®/OptraSculpt® Pad.
– Zadostna izpostavljenost svetlobi za strjevanje prepreči nepopolno polimerizacijo. Za priporočila glede časa izpostavljenosti (Exposure Time) in intenzivnosti svetlobe (Light Intensity) glejte tabelo 1. Sevalno odprtino luči držite čim bliže površini restavracijskega materiala.
– Kadar uporabljate kovinsko matrico, dodatno polimerizirajte restavracijo z bukalnega ali lingvalnega/palatalnega vidika, potem ko matrico odstranite, če niste uporabili lučke za strjevanje Bluephase® ali če svetlobne sonde ni bilo mogoče postaviti v optimalni položaj (npr. na večjo razdaljo od materiala ali z divergentnim sevanjem).
– V številnih primerih se zdaj za začetno plast uporablja tekoči kompozit, s katerim lahko oblikujete ravno dno kavitete in poenostavite prilagoditev restavracijskega materiala, ki ga boste uporabili pozneje. Tekoči kompozit (npr. Tetric EvoFlow®) lahko za tanko začetno plast uporabite po želji. Plast je treba strditi s svetlobo v ločenem koraku (treba je upoštevati ustrezna navodila za uporabo).
8. Končna obdelava/preverjanje okluzije/poliranje
Po polimerizaciji odstranite odvečen material z ustreznimi sredstvi za končno obdelavo (npr. Astropol® F) ali finimi diamantnimi svedri. Proksimalne odvečne materiale odstranite z diamantnimi ali volframovimi-karbidnimi finimi svedri, trakovi za končno obdelavo ali fleksibilnimi diski za končno obdelavo. Preverite okluzijo in artikulacijo ter z brušenjem ustrezno popravite, da preprečite prezgodnje stike ali neželene artikulacijske poti na površini restavracij. S silikonskimi polirniki (npr. Astropol® P, Astropol® HP, Astrobrush ali OptraPol® Next Generation) ter polirnimi diski in polirnimi trakovi spolirajte restavracije do trajnega visokega sijaja.

Dodatne informacije
1. Neposredno na polimeriziran material lahko po potrebi nanesete dodaten kompozit IPS Empress Direct. Če ste restavracijo s kompozitom IPS Empress Direct že spolirali, jo morate najprej obdelati tako, da postane hrapava, in namočiti z materialom Heliobond®, preden lahko nanesete novo plast kompozita IPS Empress Direct.
2. Pri uporabi mora biti kompozit IPS Empress Direct na sobni temperaturi. Hladne temperature povzročijo, da je material težko iztisniti.
3. Samo za enkratno uporabo. Če kompozit IPS Empress Direct iz kavifila nanesete neposredno v usta bolnika, je treba kavifil po uporabi zavreči, da ne pride do navzkrižne kontaminacije bolnikov.
4. Brizgalk ali kavifilov ne smete dezinficirati z oksidativnimi sredstvi za dezinfekcijo.

Opozorilo
Pazite, da nepolimeriziran kompozit IPS Empress Direct ne pride v stik s kožo, sluznico ali očmi. Nepolimeriziran kompozit IPS Empress Direct lahko učinkuje rahlo dražljivo in lahko vodi do senzibilizacije na metakrilate. Običajne medicinske rokavice ne nudijo zaščite pred občutljivostnimi reakcijami na metakrilate.

Shranjevanje
– Temperatura za shranjevanje 2–28 °C.
– Brizgalke/kavifile takoj po uporabi zaprite. Izpostavljenost svetlobi povzroči predčasno polimerizacijo.
– Kompozita IPS Empress Direct ne uporabljajte po navedenem roku uporabe.
– Življenjska doba: glejte podatke na kavifilih, brizgalkah in embalažah.

Material shranjujte zunaj dosega otrok!
Samo za dentalno uporabo.

Material je namenjen izključno za uporabo v zobozdravstvu. Uporaba mora potekati izključno v skladu z navodili za uporabo. Zaradi neupoštevanja navodil ali predpisanih območij uporabe ne prevzemamo odgo-

vornosti. Za preizkušanje ustreznosti in uporabe izdelka za kakršen koli namen, ki ni izrecno naveden v navodilih, je odgovoren uporabnik. Opisi in podatki ne predstavljajo garancije za lastnosti in niso zavezujoči.

Hrvatski

Opis
IPS Empress® Direct je svjetlosno polimerizirajući, rendgenski vidljiv, nanohibridni kompozit, utemeljen na najsuvremenijoj tehnologiji i prikladan za izvođenje restauracija koje ispunjavaju najviše zahtjeve. IPS Empress Direct polimerizira se svjetlom valne duljine u rasponu od 400-500 nm (plavo svjetlo).

Boje
IPS Empress Direct je raspoloživ u 32 različite boje i u 5 različitih stupnjeva translucencije. Raspoložive su sljedeće boje:
caklinske boje
A1, A2, A3, A3.5, A4, B1, B2, B3, B4, C1, C2, C3, D2, D3, Bleach-L, Bleach-XL
dentinske boje
A1, A2, A3, A3.5, A4, IVA5, IVA6, B1, B2, C3, D2, Bleach-L, Bleach-XL
translucentne boje
Trans 20, Trans 30, Opal*

*Nije rendgenski vidljiva uslijed visoke transparentnosti.

Sastav
Monomerna matrica sastoji se od dimetakrilata (maseni udio 20-21,5 %, za opalescentnu boju 17 %). Punila sadrže barijevo staklo, iterbij-trifluorid, miješani oksid, silicij-dioksid i kopolimer (maseni udio 77,5-79 %, za opalescentnu boju 83 %). Dodatni sastojci: aditivi, katalizatori, stabilizatori i pigmenti (< 1,0 % masenog udjela). Ukupan udio anorganskih punila iznosi 75-79 % mase ili 52-59 % volumena (opalescentna boja 60,5 % masenog ili 45 % volumenskog udjela). Veličina čestice anorganskih punila iznosi između 40 nm i 3000 nm s prosječnom veličinom čestice od 550 nm.

Indikacije
– anteriorne restauracije (razredi III, IV)
– restauracije u posteriornoj regiji (razredi I i II)
– restauracije zubnog vrata (razred V, npr. u slučaju cervikalnog karijesa, erozije korijena, klinastih defekata)
– korekcije zubnih pozicija i zubnih oblika (npr. zatvaranje dijastema, zatvaranje interdentalnih "crnih trokuta", produženje incizalnih bridova)
– direktne fasete

Kontraindikacije
Stavljanje restauracija IPS Empress Direct kontraindicirano je:
– ako se ne može uspostaviti suho radno područje ili se ne može primijeniti propisana radna tehnika;
– ako je pacijent alergičan na bilo koji sastojak materijala IPS Empress Direct.

Nuspojave
U rijetkim slučajevima komponente materijala IPS Empress Direct mogu uzrokovati senzibilizaciju. IPS Empress Direct se u tim slučajevima ne smije koristiti. Da bi se izbjegla moguća iritacija pulpe, područja u blizini pulpe moraju se zaštititi odgovarajućom zaštitom pulpe/dentina (selektivno nanesite preparat na bazi kalcijeva hidroksida na područja u blizini pulpe i obložite odgovarajućim premazom).

Interakcije
Tvari koje sadrže eugenol/ulje klinčića mogu spriječiti polimerizaciju kompozitnih materijala. Stoga je potrebno izbjegavati primjenu takvih materijala u kombinaciji s proizvodom IPS Empress Direct. U kombinaciji s kationskim vodicama za ispiranje usta, sredstvima za otkrivanje plaka i klorheksidinom može doći do diskoloracije.

Nanošenje
1. Određivanje boje
Prije određivanja boje očistite zube. Caklinska boja odabire se dok je zub još vlažan. Dentinska boja može se odrediti bilo prislanjanjem vrška uz preparirani zub ili uz cervikalno područje očnjaka.
2. Izolacija
Potrebna je odgovarajuća izolacija, najbolje gumenom plahticom (npr. OptraDam® Plus).
3. Priprema kaviteta
Priprema kaviteta provodi se sukladno zahtjevima adhezivne tehnike, tj. zaštite zubne strukture. Rubovi cakline u prednjem području su zakošeni. U stražnjem se području trebaju zaobliti samo oštri rubovi cakline (završna dijamantna brusila, 25-40 µm). Cervikalni defekti bez karijesa se ne prepariraju, već samo čiste abrazivom ili drugim prikladnim pastama za čišćenje uz pomoć gumenih čašica ili rotacionih četkica. U slučaju sklerotičnog dentina ponovno površinsko kondicioniranje pomoću okruglog svrdla može poboljšati adhezivno vezivanje. Zatim se svi ostaci u kavitetu uklone mlazom vode. Kavitet se osuši zrakom bez primjese vode i ulja.
4. Zaštita pulpe / podloga
Nemojte stavljati podlogu ako primjenjujete adheziv za caklinu/dentin. Samo vrlo duboka područja u blizini pulpe prekrijte materijalom od kalcij-hidroksida (npr. ApexCal®) te potom postavite cement otporan na pritiskanje (npr. stakleno-ionomerski cement kao što je Vivaglass® Liner). Ne prekrivajte ostale stijenke kaviteta, jer mogu poslužiti kao potporanj vezivanju adhezivom za caklinu/dentin.
5. Postavljanje matrice / interdentalnog klina
Za kavitete koji sežu u proksimalno područje primijenite cirkularnu matricu ili djelomičnu matricu i klin.
6. Kondicioniranje / nanošenje adheziva
Kondicionirajte zubnu strukturu i primijenite adheziv prema uputama za upotrebu proizvoda koji koristite. Preporučujemo upotrebu proizvoda Syntac® ili ExciTE® F (uz jetkanje fosfornom kiselinom) ili pak samojetkajućeg adheziva AdheSE® ili AdheSE® One F.
7. Nanošenje proizvoda IPS Empress Direct
– Budući da je za stvaranje anatomskog detalja dostupan veliki broj dentinskih i caklinskih boja proizvoda IPS Empress Direct različite translucencije, svaka tehnika slojevanja može se kreativno primijeniti za postizanje visoko estetskih restauracija. Za optimalan rezultat nanesite IPS Empress Direct u slojevima debljine od najviše 2 mm (ili 1,5 mm dentinske boje) i prilagodite pomoću npr. OptraSculpt®/OptraSculpt® Pad.
– Dovoljna izloženost polimerizacijskom svjetlu spriječit će nekompletno stvrdnjavanje. Preporučeno vrijeme izlaganja (Exposure Time) i intenzitet svjetla (Light Intensity) nalazi se u tabeli 1. Otvor za svjetlosnu emisiju držite što bliže površini restorativnog materijala.
– Kada koristite metalnu matricu, nakon njenog uklanjanja dodatno polimerizirajte restauraciju s bukalne ili lingvalne/palatalne strane ako niste

primijenili polimerizacijsko svjetlo Bluephase® ili ako nije bilo moguće optimalno pozicionirati svjetlosnu sondu (tj. dalje od materijala ili s divergentnim zračenjem).
– U mnogim se slučajevima za početni sloj sada primjenjuje tekući kompozit kako bi se stvorilo ravnomjerno dno kaviteta i pojednostavila prilagodba naknadno korištenog restorativnog materijala. Korištenje tekućeg kompozita (tj. Tetric EvoFlow®) kao početnog tankog sloja nije obvezno. Ovaj se sloj u odvojenom koraku mora polimerizirati svjetlom (potrebno je pridržavati se naputaka iz odgovarajućih uputa za upotrebe).

8. Završna obrada / provjera okluzije / poliranje

Višak materijala nakon polimerizacije uklonite odgovarajućim finirerima (npr. Astropol® F) ili finim dijamantima. Uklonite proksimalni suvišak dijamantnim finirerima, finirerima od volfram-karbida, trakama ili fleksibilnim diskovima za završnu obradu. Provjerite okluziju i artikulaciju te provedite odgovarajuće brusne korekcije kako bi se na površinama restauracija spriječili prijevremeni kontakti ili neželjeni artikulacijski putovi. Primjenjujte silikonske polirere (npr. Astropol® P, Astropol® HP, Astrobrush ili OptraPol® Next Generation) te diskove i trake za poliranje kako biste restauracije ispolirali do postojanog visokog sjaja.

Dodatne informacije

1. Ako je potrebno, dodatni materijal IPS Empress Direct može se izravno nanijeti na polimerizirani materijal. Ako je restauracija proizvodom IPS Empress Direct već ispolirana, ona se prije nanošenja svježeg sloja materijala IPS Empress Direct mora ohrapaviti i navlažiti sredstvom Heliobond®.
2. IPS Empress Direct prilikom nanošenja mora biti sobne temperature. Na hladnijim je temperaturama materijal teško istisnuti.
3. Samo za jednokratnu upotrebu. Ako se materijal IPS Empress Direct nanosi iz injektora Cavifil direktno u usnoj šupljini pacijenta, Cavifil se mora baciti kako bi se spriječila unakrsna kontaminacija pacijenata.
4. Štrcaljke ili injektori Cavifil ne smiju se dezinficirati oksidativnim dezinfekcijskim sredstvima.

Upozorenje

Nepolimerizirani materijal IPS Empress Direct ne smije doći u kontakt s kožom, sluznicom ili očima. Nepolimerizirani IPS Empress Direct može izazvati blažu iritaciju te uzrokovati pretjeranu osjetljivost na metakrilate. Komercijalne medicinske rukavice ne štite od osjetljivosti na metakrilate.

Skladištenje

– Temperatura skladištenja: 2-28°C.
– Štrcaljke/injektore Cavifil zatvorite odmah nakon upotrebe. Izloženost svjetlu uzrokuje preranu polimerizaciju.
– Ne primjenjujte IPS Empress Direct nakon isteka datuma valjanosti.
– Rok valjanosti: vidjeti napomenu na injektorima Cavifil, štrcaljkama i ambalaži.

Čuvati izvan dohvata djece!
Samo za stomatološku upotrebu!

Materijal je namijenjen isključivo stomatološkoj primjeni. Obrada se mora provoditi isključivo prema uputama za upotrebu. Proizvođač ne preuzima odgovornost za štete koje su rezultat nepridržavanja uputa ili navedenog područja primjene. Korisnik je odgovoran za ispitivanje prikladnosti i upotrebljivosti proizvoda za svaku svrhu koja nije izričito navedena u uputama. Opisi i podaci nisu jamstvo svojstava i nisu obvezujući.

Česky

Popis

IPS Empress® Direct je světlem tuhnoucí, pro záření neprostupný, nanohybridní kompozit na bázi nejmodernější technologie, který je vhodný pro zhotovení výplní splňujících nejvyšší nároky. IPS Empress Direct se vytvrzuje působením světla o vlnové délce 400–500 nm (modré světlo).

Odstíny

IPS Empress Direct je dostupný ve 32 různých stupních odstínu a 5 různých stupních průsvitnosti.
Dostupné jsou následující odstíny:
Odstíny skloviny
A1, A2, A3, A3.5, A4, B1, B2, B3, B4, C1, C2, C3, D2, D3, Bleach-L, Bleach-XL
Odstíny dentinu
A1, A2, A3, A3.5, A4, IVA5, IVA6, B1, B2, C3, D2, Bleach-L, Bleach-XL
Odstíny průsvitnosti
Trans 20, Trans 30, Opal* *Z důvodu své vysoké průhlednosti není neprostupný pro záření

Složení

Matrice monomeru se skládá z dimetakrylátů (20–21,5 hmot.%, opalizující odstín 17 hmot.%). Plniva se skládají z barnatého skla, trifluoridu yterbia, směsi oxidů, dioxidu křemičitého a kopolymerů (77,5–79 hmot.%, opalizující odstín 83 hmot.%). Kromě toho obsahuje aditiva, katalyzátory, stabilizátory a pigmenty (<1 hmot.%). Celkový obsah anorganických plniv je 75–79 hmot.% nebo 52–59 obj.% (opalizující odstín 60,5 hmot.% nebo 45 obj.%) Velikost částic anorganických plniv se pohybuje mezi 40 nm a 3 000 nm se střední velikostí částic 550 nm.

Indikace

– Výplně přední oblasti (třída III, IV)
– Výplně v zadní oblasti (třídy I a II)
– Opravy krčku zubu (třída V, např. v případě cervikálních kazů, eroze kořene, klínové defekty)
– Opravy zubních pozic a tvaru zubů (např. uzávěr diastemy, uzávěr mezizubních „černých trojúhelníků", prodloužení incizální hrany)
– Fazetování

Kontraindikace

Umístění výplní IPS Empress Direct je kontraindikováno:
– pokud není možné zachovat suché pracovní pole nebo dodržet předepsanou aplikační techniku.
– jestliže má pacient prokázanou alergii na některou ze složek přípravku IPS Empress Direct.

Nežádoucí účinky

Ve vzácných případech mohou složky přípravku IPS Empress Direct vést k senzitizaci. V takových případech musí být od dalšího použití IPS Empress Direct upuštěno. Aby se zabránilo iritaci pulpy, je třeba ošetřit místa v její blízkosti vhodným ochranným prostředkem na ochranu pulpy/dentinu (selektivně aplikujte přípravek na bázi hydroxidu vápenatého na oblasti v blízkosti pulpy a zakryjte adekvátní vrstvou).

Interakce
Substance obsahující eugenol nebo hřebíčkovou silici zabraňují vytvrdnutí kompozitního materiálu. Proto je třeba upustit od použití takových látek společně s přípravkem IPS Empress Direct. Při kontaktu se zásaditými ústními vodami, s indikátory plaku nebo s chlorhexidinem může dojít k nežádoucímu zabarvení.

Použití
1. Určení barvy
Před určováním barvy zuby důkladně vyčistěte. Odstín skloviny se určuje, dokud je zub ještě vlhký. Odstín dentinu může být zjištěn umístěním karty vedle připraveného zubu nebo vedle cervikální oblasti špičáku.
2. Vysušení
Je zapotřebí dostatečného vysušení, nejlépe pomocí koferdamu (např. OptraDam® Plus).
3. Preparace kavity
Při preparování kavity postupujte podle pravidel adhezivní techniky, tj. s ochranou tvrdé zubní tkáně. Kraje skloviny v přední oblasti jsou zkosené. V zadní oblasti by se měly zakulacovat pouze ostré kraje skloviny (diamantovými brousky 25–40 µm). Cervikální defekty bez kazu se nepreparují, pouze se vyčistí pemzou nebo jinými vhodnými čisticími pastami pomocí gumových nebo rotačních kartáčků. V případě sklerotického dentinu, zdrsnění povrchu vrtáčkem může zlepšit přilnutí adheziva. Následně se všechny zbytky v dutině odstraní proudem vody. Potom vysušte vzduchem neobsahujícím vodu ani olej.
4. Ochrana pulpy/podložka
Při použití pojidla na sklovinu a dentin není zapotřebí používat podložky. Pouze u velmi hlubokých kavit a kavit v blízkosti pulpy tuto oblast bodově pokryjte přípravkem obsahujícím hydroxid vápenatý (např. ApexCal®) a následně překryjte vrstvou z cementu odolného vůči tlaku (např. skloionomerní cement, jako je Vivaglass® Liner). Ostatní stěny kavity nezakrývejte, aby byla možná aplikace adheziva na sklovinu a dentin.
5. Umístění matrice/interdentálního klínku
Na kavity zasahující do proximálních oblastí použijte obalové matrice nebo použijte částečnou matrici a zaklínujte ji.
6. Kondicionování/aplikace adhezního prostředku
Při kondicionování zubní tkáně a aplikaci adhezního prostředku postupujte podle návodu k použití k používanému přípravku. Doporučujeme použití adheziva Syntac® nebo ExciTE® F (spolu s leptáním kyselinou fosforečnou) nebo samoleptací lepidlo AdheSE® nebo AdheSE® One F .
7. Použití přípravku IPS Empress Direct
– Protože pro vytvoření anatomického detailu je dostupné velké množství odstínů dentinu a skloviny v různých stupních průsvitnosti přípravku IPS Empress Direct, může se k dosažení vysoce estetické výplně kreativně použít každá technika vrstvení. K dosažení optimálního výsledku použijte IPS Empress Direct ve vrstvách o tloušťce max. 2 mm (nebo 1,5 mm odstíny dentinu) a vytvarujte použitím např. OptraSculpt®/OptraSculpt® Pad.
– Dostatečné působení vytvrzovacího světla zabraňuje neúplné polymerizaci. Doporučení ohledně expoziční doby (Exposure Time) a světelné intenzity (Light Intensity) naleznete v tabulce 1. Světelné zařízení držte co možná nejblíže povrchu výplňového materiálu.
– V případě použití kovové matrice je po odstranění matrice potřeba další osvícení výplně z bukálního nebo lingválního/palatálního pohledu, pokud se nepoužívá polymerizační světlo Bluephase®, nebo pokud lampu nelze dobře nasměrovat (např. nepřístupný kompozit nebo odchylený úhel rozptylu).
– V mnoha případech se pro první vrstvu používá zatékavý kompozit, aby bylo možné vytvořit rovné dno kavity a zjednodušit přizpůsobení následně použitého výplňového materiálu. Použití tekutého kompozitu (např. Tetric EvoFlow®) jako první vrstvy je volitelné. Tato vrstva musí být světelně vytvrzena ve zvláštním kroku (musí se postupovat podle příslušného návodu k použití).
8. Konečná úprava/kontrola okluze/leštění
Po ukončení polymerizace odstraňte přebytečný materiál pomocí finírky (např. Astropol® F) nebo jemnozrnného diamantu. Proximální přebytky odstraňte diamantovými finírkami, wolframokarbidovými finírkami nebo diamantovými brousky nebo disky. Zkontrolujte okluzi a artikulaci a zabruste tak, aby se zamezilo na povrchu náhrady předčasným kontaktům nebo artikulačním drahám. Vysokého lesku náhrady docílíte použitím silikonových lešticů (např. Astropol® P, Astropol® HP, Astrobrush nebo OptraPol® Next Generation), také lešticích disků a pásků.

Další informace
1. Jsou-li potřeba korektury, další materiál IPS Empress Direct se může použít na již polymerizovaný materiál. Jestliže je výplň IPS Empress Direct již vyleštěná, musí se nejprve zdrsnit a navlhčit přípravkem Heliobond®, teprve potom je možné nanést novou vrstvu materiálu IPS Empress Direct.
2. IPS Empress Direct by měl mít při zpracování pokojovou teplotu. Nízké teploty by mohly ztížit vytlačování.
3. Určeno pouze pro jednorázové použití. Jestliže se materiál IPS Empress Direct z aplikační stříkačky Cavifil používá přímo v ústech pacienta, Cavifil se po použití musí zlikvidovat, aby nedošlo ke křížové kontaminace mezi pacienty.
4. Stříkačky ani Cavifil se nesmí dezinfikovat oxidačními dezinfekčními prostředky.

Varování
Vyhněte se kontaktu nevytvrzeného materiálu IPS Empress Direct s pokožkou, sliznicí a očima. Nepolymerizovaný materiál IPS Empress Direct může v nevytvrzeném stavu působit dráždivě a vést k přecitlivělosti vůči metakrylátům. Běžně dostupné lékařské rukavice nechrání před senzibilizujícím účinkem metakrylátů.

Uchovávání
– Teplota uchovávání: 2–28 °C.
– Stříkačky/Cavifil po použití ihned uzavřete. Přístup světla vede k předčasné polymerizaci.
– Po uplynutí data použitelnosti materiál IPS Empress Direct již nepoužívejte.
– Doba použitelnosti: viz údaj na Cavifil, na stříkačkách a obalu.

Skladujte mimo dosahu dětí!
Určeno pouze pro stomatologické účely.

Materiál byl vyvinut výlučně pro použití v zubním lékařství. Zpracování je nutné provádět výhradně podle návodu k použití. Nelze přijmout odpovědnost za škody vzniklé nedodržováním pokynů nebo stanovené oblasti použití. Uživatel odpovídá za testování výrobků z hlediska jeho vhodnosti a použití pro jakékoliv účely výslovně neuvedené v návodu. Popisy a údaje nepředstavují žádnou záruku vlastností a nejsou závazné.

Popis
IPS Empress® Direct je svetlom vytvrdzovaný nano-hybridný kompozit neprepúšťajúci RTG žiarenie, založený na najnovšej technológii, ktorý je vhodný na prípravu náhrad spĺňajúcich najvyššie požiadavky. IPS Empress Direct sa vytvrdzuje svetlom s vlnovou dĺžkou 400 – 500 nm (modré svetlo).

Odtiene
IPS Empress Direct sa dodáva v 32 rôznych odtieňoch a 5 rôznych stupňoch priesvitnosti.
K dispozícii sú nasledujúce odtiene:
Odtiene skloviny
A1, A2, A3, A3.5, A4, B1, B2, B3, B4, C1, C2, C3, D2, D3, Bleach-L, Bleach-XL
Odtiene zuboviny
A1, A2, A3, A3.5, A4, IVA5, IVA6, B1, B2, C3, D2, Bleach-L, Bleach-XL
Priesvitné odtiene
Trans 20, Trans 30, Opal*

* Prepúšťa RTG žiarenie, lebo je vysoko transparentný

Zloženie
Monomérová matrica je zložená z dimetakrylátov (20 – 21,5 hmot. %, opaleskujúci odtieň 17 hmot. %). Plnivá sú zložené z bárnatého skla, fluoridu yterbitého, zo zmesi oxidu, oxidu kremičitého a kopolyméru (77,5 – 79 hmot. %, opaleskujúci odtieň 83 hmot. %). Okrem toho obsahuje prísady, katalyzátory, stabilizátory a pigmenty (< 1,0 hmot. %). Celkový obsah anorganických plnív je 75 – 79 hmot. % alebo 52 – 59 objem. % (opaleskujúci odtieň 60,5 hmot. % alebo 45 objem. %). Veľkosť častíc anorganických plnív je 40 nm až 3000 nm s priemernou veľkosťou častíc 550 nm.

Indikácie
– Predné náhrady (trieda III, IV)
– Náhrady v zadnej časti (triedy I a II)
– Náhrady krčkov zubov (trieda V, napr. pri kaze krčku, erózii koreňa, chybách v tvare klinov)
– Opravy polôh zubov a tvaru zubov (napr. uzavretie medzery medzi zubami, uzavretie medzizubných „čiernych trojuholníkov", predĺženie incíznej hrany)
– Priame fazety

Kontraindikácie
Príprava náhrad IPS Empress Direct je kontraindikovaná,
– ak nie je možné zaistenie suchého poľa alebo dodržanie predpísanej techniky,
– pri preukázanej alergii pacienta na niektoré zo zložiek prípravku IPS Empress Direct.

Vedľajšie účinky
Zložky prípravku IPS Empress Direct môžu v zriedkavých prípadoch spôsobiť precitlivenosť. IPS Empress Direct sa v takých prípadoch nemá použiť. Ak chcete zabrániť možnému podráždeniu zubnej drene, miesta blízke k zubnej dreni sa majú chrániť vhodnou ochranou zubnej drene/zuboviny (selektívne naneste prípravok na báze hydroxidu vápenatého do miest blízkych zubnej dreni a pokryte vhodnou ochrannou vrstvou).

Interakcie
Látky obsahujúce eugenol/klinčekový olej môžu inhibovať polymerizáciu kompozitných materiálov. Takéto materiály sa preto nesmú používať spolu s prípravkom IPS Empress Direct. Pri styku s katiónovými ústnymi vodami, prostriedkami na odhaľovanie zubného plaku a chlórhexidínom môže dôjsť k nežiaducemu sfarbeniu.

Použitie
1. Určenie odtieňa
Pred určením odtieňa zuby vyčistite. Odtieň skloviny sa určuje, kým je zub ešte vlhký. Odtieň zuboviny možno určiť buď priložením plôšky vedľa preparovaného zuba, alebo vedľa oblasti krčka očného zuba.
2. Izolácia
Je potrebná vhodná izolácia, najlepšie pomocou koferdamu (napr. OptraDam® Plus).
3. Príprava kavity
Príprava kavity sa vykonáva podľa požiadaviek adhezívnej techniky, t. j. ochranou štruktúry zuba. Okraje skloviny v prednej časti chrupu sa skosia. V zadnej časti sa zaoblia len ostré okraje skloviny (konečná úprava diamantom, 25 – 40 µm). Chyby krčka bez kazu sa nepreparujú, len očistia pemzou alebo inými vhodnými čistiacimi pastami pomocou gumových kalíškov alebo otáčavých kefiek. V prípade sklerotickej zuboviny môže spojenie lepidla zlepšiť povrchová úprava použitím vrtáka s ružicovou hlavou. Následne sa z kavity postrekom vodou odstránia všetky zvyšky. Kavita sa vysuší vzduchom bez vody a oleja.
4. Ochrana zubnej drene/základný materiál
Pri použití adhezíva na sklovinu a zuboviny neaplikujte základný materiál. Len pri veľmi hlbokých kavitách v blízkosti zubnej drene túto oblasť prekryte preparátom obsahujúcim hydroxid vápenatý (napr. ApexCal®) a následne ju prekryte vrstvou cementu odolného voči tlaku (napr. skloionomérový cement, ako je Vivaglass® Liner). Zvyšné steny kavity neprekrývajte, aby sa dali použiť na podporu spojenia adhézneho prípravku na sklovinu a zuboviny.
5. Nasadenie matrice/medzizubného klinu
V prípade kavít zasahujúcich do proximálneho priestoru použite obvodovú matricu alebo separačnú matricu a klin.
6. Príprava/aplikácia adhézneho prostriedku
Pri príprave štruktúry zuba a aplikovaní adhézneho prostriedku postupujte podľa návodu k používanému prípravku. Odporúčame použitie prostriedku Syntac® alebo ExciTE® F (spolu s leptaním kyselinou fosforečnou) alebo samoleptacieho adhézneho prípravku AdheSE® či AdheSE® One F.
7. Použitie prípravku IPS Empress Direct
– Keďže je k dispozícii veľký počet odtieňov zuboviny a skloviny IPS Empress Direct s rôznymi priepustnosťami svetla na vytváranie anatomických detailov, na dosiahnutie vysoko estetických náhrad sa môže použiť kreatívne každá vrstviaca technika. Optimálny výsledok dosiahnete pri použití prípravku IPS Empress Direct vo vrstvách max. hrúbky 2 mm (alebo 1,5 mm odtiene zuboviny) a úpravou napr. nástrojom OptraSculpt®/ OptraSculpt® Pad.
– Dostatočná expozícia vytvrdzovacím svetlom zabraňuje neúplnej polymerizácii. Odporúčania týkajúce sa času expozície (Exposure Time) a intenzity svetla (Light Intensity) nájdete v tabuľke 1. Okienko emisie svetla držte čo najbližšie k povrchu materiálu náhrady.
– Pri použití kovovej matrice ďalej polymerizujte náhradu z bukálnej alebo linguálno-palatálnej strany po vybratí matrice, ak sa nepoužilo vytvrdzovacie svetlo Bluephase® alebo lampa nemohla byť optimálne umiestnená (napr. ďaleko od materiálu alebo s rozbiehavým vyžarovaním).

– V mnohých prípadoch sa teraz na prvú vrstvu používa tekutý kompozit, aby sa vytvoril rovný základ kavity a uľahčila sa úprava následne použitého materiálu náhrady. Použitie tekutého kompozitu (napr. Tetric EvoFlow®) ako prvej tenkej vrstvy je voliteľné. Táto vrstva sa musí vytvrdiť svetlom v osobitnom kroku (musia sa dodržať pokyny príslušného návodu na použitie).

8. Konečná úprava/kontrola oklúzie/leštenie
Po polymerizácii odstráňte prebytočný materiál pomocou vhodných dokončovacích nástrojov (napr. Astropol® F) alebo jemných diamantov. Proximálny prebytočný materiál odstráňte pomocou diamantových alebo volfrámovo-karbidových dokončovacích nástrojov, dokončovacích prúžkov alebo pružných dokončovacích kotúčov. Skontrolujte oklúziu a artikuláciu a vykonajte vhodné opravy zbrúsením, aby sa zabránilo predčasnému kontaktu alebo nežiaducim artikulačným dráham na povrchu náhrad. Pomocou silikónových leštidiel (napr. Astropol® P, Astropol® HP, Astrobrush alebo OptraPol® Next Generation), ako aj leštiacich kotúčov a leštiacich prúžkov vyleštite náhradu na trvanlivý vysoký lesk.

Ďalšie informácie
1. V prípade potreby sa na polymerizovaný materiál môže priamo naniesť ďalší materiál IPS Empress Direct. Ak bola náhrada z prípravku IPS Empress Direct už vyleštená, najprv sa musí zdrsniť a zvlhčiť prípravkom Heliobond® a potom naniesť čerstvý materiál IPS Empress Direct.
2. IPS Empress Direct má mať pri použití teplotu miestnosti. Pri nižšej teplote môže dôjsť k sťaženiu vytláčania.
3. ⊗ Len na jednorazové použitie. Ak sa IPS Empress Direct používa priamo z plniacej pištole Cavifil v ústach pacienta, Cavifil sa musí zlikvidovať po použití, aby sa zabránilo skríženej kontaminácii medzi pacientmi.
4. Striekačky alebo Cavifil sa nemajú dezinfikovať oxidujúcimi dezinfekčnými látkami.

Upozornenie
Nepolymerizovaný materiál IPS Empress Direct nemá prísť do styku s pokožkou, sliznicou alebo očami. Nepolymerizovaný materiál IPS Empress Direct môže spôsobiť ľahké podráždenie a viesť k precitlivenosti na metakryláty. Komerčne dostupné lekárske rukavice nechránia pred senzibilizujúcim účinkom metakrylátov.

Uchovávanie
– Skladovacia teplota: 2 – 28 °C.
– Injekčné striekačky/plniace pištole (Cavifil) po použití okamžite zatvorte. Prístup svetla spôsobuje predčasnú polymerizáciu.
– Materiál IPS Empress Direct nepoužívajte po vyznačenom dátume exspirácie.
– Čas použitia: pozri informácie na plniacej pištoli, striekačke a obaloch.

Materiál uchovávajte mimo dosahu detí!
Určené výhradne na stomatologické účely.

Tento materiál bol vyvinutý výhradne na stomatologické účely. Spracovanie sa musí vykonávať striktne podľa návodu na použitie. Výrobca nepreberá žiadnu zodpovednosť za škody spôsobené nedodržaním návodu na použitie alebo predpísanej oblasti aplikácie. Používateľ je zodpovedný za testovanie produktov ohľadom ich vhodnosti a použitia na akýkoľvek účel, ktorý nie je výslovne uvedený v návode na použitie. Opis a údaje nepredstavujú žiadnu záruku vlastností a nie sú záväzné.

Magyar

Leírás
Az IPS Empress® Direct a legkorszerűbb technológián alapuló, fényre keményedő, sugárfogó nanohibrid kompozit, amely alkalmas a legmagasabb igényeket is kielégítő restaurációk készítésére. IPS Empress Direct 400–500 nm hullámhossztartományú (kék) fényre keményedik.

Árnyalatok
Az IPS Empress Direct 32 különböző árnyalatban és az áttetszőség öt különböző fokozatában kapható. Az alábbi árnyalatok állnak rendelkezésre:
Zománcárnyalatok
A1, A2, A3, A3.5, A4, B1, B2, B3, B4, C1, C2, C3, D2, D3, Bleach-L, Bleach-XL
Dentinárnyalatok
A1, A2, A3, A3.5, A4, IVA5, IVA6, B1, B2, C3, D2, Bleach-L, Bleach-XL
Áttetsző árnyalatok
Trans 20, Trans 30, Opal*

*Nem sugárfogó a nagy áttetszőség miatt

Összetétel
A monomermátrix dimetil-akrilátokból (20-21,5 súly%, áttetsző árnyalat 17 súly%) áll. A töltőanyagok báriumüveget, itterbium-trifluoridot, elegyoxidot, szilícium-dioxidot és kopolimert (77,5–79 súly%, áttetsző árnyalat 83 súly%) tartalmaznak. További tartalom: adalékanyagok, katalizátorok, stabilizátorok és pigmentek (< 1,0 súly%). A teljes szervetlen töltőanyagtartalom 75-79 súly% vagy 52-59 térfogat% (áttetsző árnyalat 60,5 súly% vagy 45 térfogat%). A szervetlen töltőanyagok részecskemérete 40 nm és 3000 nm között van, 550 nm-es átlagos részecskemérettel.

Javallat
– Elülső restaurációk (III., IV. osztály)
– Restaurációk a hátulsó fogtérségben (I. és II. osztály)
– Fognyaki restaurációk (V. osztály, pl. fognyakszuvasodás, gyökérerózió, ék alakú hibák)
– A foghelyzet és fogalak javításai (pl. diastema lezárása, fogak közötti „fekete háromszögek” lezárása, az incizális él meghosszabbítása)
– Közvetlen lefedés

Ellenjavallat
Az IPS Empress Direct restaurációk elhelyezése ellenjavallt:
– ha száraz munkatérség nem biztosítható, vagy ha az előírt technika nem alkalmazható
– ha ismeretes, hogy a páciens allergiás az IPS Empress Direct valamely összetevőjére

Mellékhatások
Ritka esetben az IPS Empress Direct összetevői érzékenyítést okozhatnak. Ilyen esetekben tilos az IPS Empress Direct használata. A fogbél esetleges irritációjának elkerülése céljából a fogbélhez közeli területeket megfelelő fogbél/dentin védővel kell védeni (pontszerűen alkalmazzon kalcium-hidroxid alapú készítményt a fogbélhez nagyon közeli területeken, majd fedje le megfelelő alátöméssel).

Kölcsönhatások
Eugenolt vagy szegfűszegolajat tartalmazó anyagok megakadályozhatják a kompozitanyagok polimerizációját. Következésképpen ilyen anyagok és az IPS Empress Direct együttes használatát feltétlenül kerülni kell. Elszíneződés

léphet fel az alábbiak egyidejű jelenlétekor: kationos szájvizek, plakkot kimutató szerek és klórhexidin.

Alkalmazás

1. Színmeghatározás

Színmeghatározás előtt tisztítsa meg a fogat. A zománcárnyalatot még nedves fog esetén kell meghatározni. A dentinárnyalatot a fül az előkészített fog vagy a szemfog fognyaki területe mellé helyezésével lehet meghatározni.

2. Izolálás

Megfelelő mértékű izolálásra van szükség, amire legjobb a gumigát (pl. OptraDam® Plus).

3. Üregelőkészítés

Az üregelőkészítés az adhéziós technika követelményei szerint történik, azaz a fogszerkezet védelmével. Az elülső fogtérségben a zománcélek ferdére vannak csiszolva. A hátsó térségben csak az éles zománcéleket kell lekerekíteni (finírozó gyémántokkal, 25-40 µm). A fogszuvasodás-mentes nyaki hibákat nem kell előkészíteni, csak meg kell azokat tisztítani horzskőporral vagy más megfelelő tisztítópasztával, gumikelyheket vagy forgó keféket használva. Szklerotikus dentin esetén gömbfúróval való felszíni helyreállítás javíthatja az adhéziós kötést. Ezután vízpermettel az összes maradékanyag eltávolításra kerül az üregből. Végül az üreg meg lesz szárítva víz- és olajmentes levegővel.

4. Fogbélvédelem/alapozás

Ne alkalmazzon alapozó anyagot, ha zománc/dentin kötőanyagot használ. Csak igen mély, fogbélközeli térségek esetén fedje le a területet kalcium-hidroxid készítménnyel (pl. ApexCal®), majd alkalmazzon nyomásálló cementréteget (pl. üvegionomer-cement, pl. Vivaglass® Liner). Ne fedje le az üreg többi falát, mivel ezek használatosak a kötés elősegítésére zománc/dentin ragasztóanyaggal.

5. Matrica/fogközi ék elhelyezése

Használjon körülfogó matricát a proximális térségre kiható üregeknél, vagy pedig szétszedhető matricát és ékelje be.

6. Előkezelés/kötőanyag alkalmazása

Az előkezelést és a kötőanyag alkalmazását az alkalmazott termék használati utasításai szerint végezze. Mi a Syntac® vagy az ExciTE® F (foszforsavas maratással), illetve az AdheSE® vagy az AdheSE® One F önmarató ragasztó alkalmazását javasoljuk.

7. Az IPS Empress Direct alkalmazása

– Mivel az anatómiai részletek létrehozásához az IPS Empress Direct dentin- és zománcárnyalatai különböző transzlucenciafokozatokban állnak rendelkezésre, mindenfajta rétegezési technika kreatívan használható rendkívül esztétikus restaurációk előállításához. Optimális eredmények érdekében az IPS Empress Direct anyagot maximum 2 mm-es rétegekben (vagy dentinárnyalatok esetén 1,5 mm-es rétegekben) alkalmazza, és adaptálja pl. OptraSculpt®/OptraSculpt® Pad párnával.
– A kikeményítő fénnyel való kielégítő megvilágítás megakadályozza a nem teljes polimerizációt. A megvilágítási időre (Exposure Time) és a fényintenzitásra (Light Intensity) vonatkozó javaslatokat lásd az 1. táblázatban. Tartsa a fénykibocsátó ablakot a lehető legközelebb a restaurációs anyag felületéhez.
– Fémmatrica használata esetén a matrica kivételét követően még polimerizálja tovább a kompozitanyagot bukkális vagy lingvális/palatinális irányból, ha nem Bluephase® polimerizáló fény volt használatban, vagy a fényvezetőt nem lehet ideálisan behelyezni (pl. az anyagtól távolról vagy divergens sugárzással).
– Sok esetben folyóképes kompozit van használatban alaprétegként, egyenletes üregalapzat létrehozása céljából, illetve az azután alkalmazott restaurációs anyag adaptációjának megkönnyítéséhez. Folyóképes kompozit (pl. Tetric EvoFlow®) használata vékony alaprétegként opcionális. Ezt a réteget ki kell keményíteni külön lépésben (a vonatkozó használati utasítás utasításait be kell tartani).

8. Kikészítés/lezárás-ellenőrzés/polírozás

Polimerizáció után távolítsa el a felesleges anyagot megfelelő kikészítőkkel (pl. Astropol® F) vagy finom gyémánttal. Távolítsa el a proximális felesleget gyémánt- vagy volfrámkarbid-kikészítőkkel, kikészítő szalagokkal vagy hajlékony kikészítő korongokkal. Ellenőrizze a lezárást és az illeszkedést, és csiszolással készítsen megfelelő korrekciókat a restauráció felületén, a túl korai érintkezés, illetve a nemkívánatos illeszkedési útvonalak megelőzése céljából. Használjon szilikon polírozókat (pl. Astropol® P, Astropol® HP, Astrobrush vagy OptraPol® Next Generation), illetve polírozó korongokat és polírozó szalagokat a restauráció tartós tükörfényre polírozásához.

További információ

1. Ha szükséges, további IPS Empress Direct anyag alkalmazható közvetlenül a polimerizált anyagra. Ha már megtörtént a IPS Empress Direct restauráció polírozása, azt érdesíteni kell és meg kell nedvesíteni Heliobond® használatával, mielőtt új réteg IPS Empress Direct lenne alkalmazható.
2. Az IPS Empress Direct alkalmazáskor szobahőmérsékletű kell legyen. Hűvös hőmérséklet az anyagot nehezen extrudálhatóvá teszi.
3. Csak egyszeri használatra. Ha az IPS Empress Direct alkalmazása a Cavifil-ból közvetlenül a beteg szájába történik, használat után a Cavifil-t a hulladékba kell helyezni, a betegek közötti keresztfertőzés megelőzése céljából.
4. A fecskendők vagy Cavifil-ek nem fertőtleníthetők oxidáló fertőtlenítő szerekkel.

Figyelmeztetés

A nem polimerizált IPS Empress Direct nem érintkezhet bőrrel, nyálkahártyákkal vagy szemmel. A nem polimerizált IPS Empress Direct enyhén irritáló hatású lehet, és metakrilátokkal szembeni érzékenyítést okozhat. A kereskedelemben kapható orvosi kesztyűk nem nyújtanak védelmet a metakrilátok érzékenyítő hatásával szemben.

Tárolás

– Tárolási hőmérséklet: 2-28 °C.
– Használat után azonnal zárja le a fecskendőket/Cavifil-eket. Fény hatására idő előtti polimerizáció lép fel.
– Az IPS Empress Direct használata tilos a lejárati időn túl.
– Tárolási idő: Lásd a Cavifil-eken, fecskendőkön és csomagokon lévő információt.

Az anyag gyermekektől elzárva tartandó.
Csak fogászati használatra.

Az anyag kizárólag fogászati használatra lett kifejlesztve. A feldolgozás pontosan meg kell feleljen a Használati utasításnak. A használati utasításban leírtaktól eltérő vagy az előírttól eltérő területen történő alkalmazás miatt keletkezett kárra vonatkozóan nem vállalható felelősség. A felhasználó a felelős a termék alkalmasságának ellenőrzéséért, és minden, az ebben a használati utasításban nem kifejezetten említett célra való használatért. A leírások és adatok nem jelentik a tulajdonságok garanciáját.

Opis materiału
IPS Empress® Direct jest światłoutwardzalnym, nanohybrydowym materiałem złożonym, widocznym w promieniach rentgenowskich, stworzonym z wykorzystaniem najnowszych technologii. Przeznaczony jest do wykonywania wypełnień spełniających oczekiwania najbardziej wymagających pacjentów. Materiał IPS Empress Direct polimeryzuje pod wpływem światła zawierającego pasmo o długości fali 400-500 nm (światło niebieskie).

Kolory
Materiał IPS Empress Direct dostępny jest w 32 kolorach i w 5 stopniach translucencji:
Kolory szkliwne
A1, A2. A3, A3.5, A4, B1, B2, B3, B4, C1, C2, C3, D2, D3, Bleach-L, Bleach-XL
Kolory zębinowe
A1, A2, A3, A3.5, A4, IVA5, IVA6, B1, B2, C3, D2, Bleach-L, Bleach-XL
Kolory translucentne
Trans 20, Trans 30, Opal*
*Materiały w tych kolorach nie dają kontrastu na zdjęciach rentgenowskich, ze względu na ich wysoką przezierność

Skład
Matryca monomerowa składa się z dimetakrylanów (20-21.5% wag., w kolorach opalescencyjnych 17% wag.). Wypełniacz nieorganiczny stanowią: szkło barowe, trójfluorek iterbu, mieszanina tlenków, dwutlenek krzemu oraz kopolimery (77,5-79%wag., w kolorach opalescencyjnych 83%wag.) Składniki dodatkowe: katalizatory, stabilizatory i barwniki stanowią poniżej 1% wag. Całkowita zawartość wypełniacza nieorganicznego wynosi:
75-79% wag. lub 52-59% obj., (w materiałach o kolorach opalescencyjnych odpowiednio 60,5% wag. lub 45% obj.). Wielkość ziaren wypełniacza nieorganicznego waha się od 40 nm do 3000 nm. Średnia wielkość ziaren wynosi 550 nm.

Wskazania
– Wypełnianie ubytków w odcinku przednim (klasy III i IV)
– Wypełnianie ubytków w zębach bocznych (klasy I i II)
– Wypełnianie ubytków przyszyjkowych (klasy V, np. ubytków pochodzenia próchnicowego, nadżerek w obrębie korzenia, ubytków klinowych)
– Korekta ustawienia i kształtu zębów (np. zamykanie diastem, zamykanie „czarnych trójkątów", wydłużanie brzegu siecznego
– Licowanie zębów metodą bezpośrednią

Przeciwwskazania
Materiału IPS Empress Direct nie należy stosować w przypadku:
– braku możliwości utrzymania wymaganej suchości pola zabiegowego i przestrzegania zalecanej techniki pracy,
– znanej nadwrażliwości pacjenta na którykolwiek ze składników materiału IPS Empress Direct.

Działania niepożądane
W rzadkich przypadkach, pewne składniki materiału IPS Empress Direct mogą wywołać nieznaczną reakcję nadwrażliwości. Należy wtedy zaniechać stosowania tego materiału.
W celu uniknięcia ewentualnego podrażnienia miazgi, należy miejsca w jej pobliżu przykryć odpowiednim materiałem chroniącym miazgę i zębinę (miejsca w pobliżu miazgi punktowo przykryć materiałem zawierającym wodorotlenek wapnia, a następnie odpowiednim materiałem podkładowym).

Interakcje
Materiały zawierające eugenol lub olejek goździkowy mogą hamować polimeryzację materiałów złożonych. Z tego powodu należy unikać stosowania tego rodzaju materiałów w połączeniu z IPS Empress Direct. Związki kationowe stosowane w płynach do płukania jamy ustnej, środki służące do wybarwiania płytki nazębnej oraz chlorheksydyna, mogą powodować przebarwienia wypełnień z materiału IPS Empress Direct.

Sposób postępowania
1. Dobór koloru materiału
Przed określeniem koloru wypełnienia, zęby należy oczyścić. Podczas dobierania koloru szkliwnego wypełnienia, zęby powinny być wilgotne. Kolor zębinowy można dobrać bezpośrednio do koloru leczonego zęba i porównać z kolorem kła w okolicy przyszyjkowej.
2. Izolacja pola operacyjnego
Pole operacyjne należy odpowiednio zabezpieczyć przed wilgocią, najlepiej za pomocą koferdamu (np. OptraDam® Plus).
3. Opracowanie ubytku
Ubytek opracować według zasad techniki adhezyjnej, tj. w sposób oszczędzający twarde tkanki zęba. W zębach przednich należy ukośnie ściąć brzegi szkliwa. W zębach bocznych należy jedynie zaokrąglić ostre brzegi szkliwa (za pomocą wiertła z drobnoziarnistym nasypem diamentowym 25-40 µm). Ubytki przyszyjkowe pochodzenia niepróchnicowego należy oczyścić pumeksem lub odpowiednią pastą czyszczącą za pomocą gumek lub rotacyjnych szczoteczek. W przypadku, gdy w ubytku mamy zębinę sklerotyczną, ponowne, powierzchniowe kondycjonowanie zębiny, może znacznie poprawić siłę łączenia. Następnie ubytek należy wypłukać wodą, w celu usunięcia wszelkich zanieczyszczeń i wysuszyć powietrzem bez oleju i wody.
4. Ochrona miazgi / Założenie podkładu
W ubytkach płytkich i średniej głębokości nie należy zakładać podkładu, o ile stosowany jest system łączący ze szkliwem i zębiną. Jedynie w ubytkach głębokich, miejsca w pobliżu miazgi, należy punktowo pokryć cienką warstwą materiału zawierającego wodorotlenek wapniowy (np. ApexCal®). Warstwę tę należy przykryć nierozpuszczalnym, mechanicznie wytrzymałym materiałem podkładowym, np. cementem szklano-jonomerowym (np. Vivaglass® Liner). Nie pokrywać pozostałych ścian ubytku materiałem podkładowym. Odsłonięta zębina jest niezbędna do wytworzenia połączenia z systemem łączącym.
5. Zakładanie formówki i klinów międzyzębowych
Aby odbudować ubytek, należy założyć formówkę okrężną, zwracając przy tym szczególną uwagę na powierzchnie styczne: mezjalną i dystalną, lub też zastosować formówkę częściową. Oba rodzaje formówek należy zaklinować przydziąsłowo.
6. Wytrawianie / Nakładanie materiału łączącego
Należy dokładnie zapoznać się z instrukcją stosowania systemu łączącego i zaleceniami producenta. W technice całkowitego wytrawiania szkliwa i zębiny, firma Ivoclar Vivadent zaleca systemy łączące Syntac® lub ExciTE® F. Można zastosować także samowytrawiające systemy łączące AdheSE® lub AdheSE® One F.
7. Nakładanie materiału IPS Empress Direct

– Materiał IPS Empress Direct dostępny jest w szerokiej gamie kolorów szkliwnych i zębinowych o różnej translucencji. W celu wykonania wypełnień o wysokiej estetyce można stosować różne techniki odbudowy warstwowej. Aby uzyskać optymalny efekt, należy nałożyć materiał IPS Empress Direct Enamel w warstwie o maksymalnej grubości 2 mm, lub 1,5 mm.- w przypadku kolorów zębinowych – Dentin. Powierzchnię wypełnienia należy ukształtować i wymodelować przy użyciu OptraSculpt®/ OptraSculpt® Pad.
– Odpowiednia ekspozycja na światło pozwala na całkowitą polimeryzację materiału. Zalecenia dotyczące czasu naświetlania (Exposure Time) oraz intensywności światła (Light Intensity) zostały przedstawione w tabeli 1. Należy utrzymywać źródło światła jak najbliżej powierzchni polimeryzowanego materiału.
– Jeśli używana jest formówka metalowa, to dodatkowo po jej usunięciu należy spolimeryzować uzupełnienie od strony policzkowej lub językowo-podniebiennej. Taką procedurę należy przeprowadzić również wtedy, gdy nie jest używana lampa Bluephase® lub jeśli światłowód użytej lampy nie jest odpowiednio ustawiony, ewentualnie w przypadku, gdy znajduje się on zbyt daleko od utwardzanego materiału lub lampa emituje światło o odmiennej długości fali, w stosunku do długości niezbędnej do polimeryzacji materiału.
– W wielu przypadkach płynny kompozyt jest obecnie stosowany jako pierwsza warstwa wypełnienia, co pozwala uzyskać dokładne pokrycie dna ubytku i upraszcza adaptację materiału o regularnej gęstości. Stosowanie płynnego kompozytu (Tetric EvoFlow®) jako pierwszej warstwy, jest opcjonalne. Polimeryzacja tej warstwy musi być jednak przeprowadzona oddzielnie, ściśle według instrukcji stosowania materiału.

8. Końcowe opracowanie / Kontrola okluzji i artykulacji / Polerowanie wypełnienia

Po polimeryzacji należy usunąć nadmiary materiału wypełniającego wiertłem z drobnoziarnistym nasypem diamentowym lub odpowiednimi gumkami (np. Astropol F®). Z przestrzeni międzyzębowych materiał usunąć wiertłami z nasypem diamentowym, ewentualnie przy użyciu wierteł, wykonanych z wolframu lub z węglika spiekanego. Następnie należy dokładnie skontrolować kontakty zębów w okluzji podczas artykulacji i dokonać niezbędnych korekt przez szlifowanie, w celu uniknięcia przedwczesnych kontaktów i nieprawidłowego prowadzenia zgryzu.
Wypełnienie wypolerować do uzyskania wysokiego połysku gumkami silikonowymi (np. Astropol® P, Astropol® HP), szczoteczkami polerującymi Astrobrush lub OptraPol® Next Generation) krążkami i paskami ściernymi.

Uwagi dodatkowe

1. O ile to konieczne, materiał IPS Empress Direct może być nakładany na spolimeryzowane wypełnienie. Jeśli jego powierzchnia została wypolerowana, przed nałożeniem nowej warstwy materiału, należy ją schropowacić i pokryć niewielką ilością materiału Heliobond.
2. Podczas nakładania, materiał IPS Empress Direct powinien mieć temperaturę pokojową. Niska temperatura sprawia, że jest on trudny do wyciśnięcia z opakowania.
3. Tylko do jednorazowego użytku. W przypadku nakładania materiału IPS Empress Direct bezpośrednio z pojemnika cavifil w ustach pacjenta, ze względów higienicznych, (w celu uniknięcia zakażeń krzyżowych), pojemniki Cavifil powinny być stosowane jednorazowo dla każdego pacjenta.
4. W celu dezynfekcji strzykawek i jednorazowych pojemników cavifil nie należy stosować środków dezynfekcyjnych o właściwościach utleniających.

Ostrzeżenie

Należy unikać kontaktu niespolimeryzowanego materiału IPS Empress Direct ze skórą, błoną śluzową i oczami, ponieważ może on spowodować niewielkie podrażnienie i wywołać reakcję nadwrażliwości na metakrylany.
Rękawiczki medyczne będące w powszechnym użyciu nie chronią przed uczulającym działaniem metakrylanów.

Warunki przechowywania

– Przechowywać w temperaturze 2-28 °C.
– Materiału IPS Empress Direct nie należy stosować po upływie terminu ważności.
– Pojemniki Cavifil lub strzykawki należy zamykać natychmiast po ich użyciu. Kontakt ze światłem powoduje przedwczesną polimeryzację materiału złożonego.
– Data ważności: podana jest na strzykawkach, jednorazowych pojemnikach cavifil i opakowaniu.

Materiał przechowywać w miejscu niedostępnym dla dzieci.
Materiał przeznaczony tylko do użytku w stomatologii.

Materiały są przeznaczone wyłącznie do stosowania w stomatologii. Przy ich użyciu należy ściśle przestrzegać instrukcji stosowania. Nie ponosi się odpowiedzialności za szkody powstałe w wyniku nieprzestrzegania instrukcji, lub użycia materiałów niezgodnie ze wskazaniami. Użytkownik jest odpowiedzialny za testowanie materiałów dla swoich własnych celów i za ich użycie w każdym innym przypadku nie wyszczególnionym w instrukcji. Opisy materiałów i ich skład nie stanowią gwarancji i nie są wiążące.

Српски

Опис

IPS Empress® Direct je рендгенски видљив нанохибридни композит који полимеризује на светлу. Заснива се на најсавременијој технологији и погодан је за рестаурације према највишим стандардима. IPS Empress Direct полимеризује на светлости таласне дужине од 400 до 500 nm (плаво светло).

Боје

IPS Empress Direct доступан је у 32 различите боје и 5 различитих нивоа транслуцентности. На располагању су следеће боје:
Глеђне боје
A1, A2, A3, A3.5, A4, B1, B2, B3, B4, C1, C2, C3, D2, D3, Bleach-L, Bleach-XL
Дентинске боје
A1, A2, A3, A3.5, A4, IVA5, IVA6, B1, B2, C3, D2, Bleach-L, Bleach-XL
Транслуцентне боје
Trans 20, Trans 30, Opal*

*Није рендгенски видљива због високе транспарентности

Састав

Мономерну матрицу чине диметакрилати (тежински удео 20-21,5 %, код прозрачних боја тежински удео 17 %). Пунилац садржи баријумско стакло, итербијум трифлуорид, мешани оксид, силикон диоксид и кополимер (тежински удео 77,5-79 %, код прозрачних боја тежински удео 83 %). Додатни састојци: адитиви, катализатори, стабилизатори и пигменти (тежински удео < 1,0). Укупан садржај неогранских пунилаца износи 75-79 % тежинског или 52-59 % запреминског удела (код

прозрачних боја тежински удео је 60,5 %, а запремински 45 %). Величина честица неорганских пунилаца је од 40 nm до 3000 nm, при чему је средња вредност величине честица 550 nm.

Индикације
– Рестаурације предњих зуба (класе III, IV)
– Рестаурације у бочним регијама (класе I и II)
– Рестаурације врата зуба (класе V, нпр. код цервикалног каријеса, ерозије корена, клинастих дефеката)
– Корекције положаја и облика зуба (нпр. затварање дијастеме, затварањегцрних троуглова" између зуба, продужење инцизалне ивице)
– Директне навлаке

Контраиндикације
Рестаурација помоћу IPS Empress Direct контраиндикована је
– када није могуће обезбедити суво радно поље или применити прописану технику;
– ако се зна да је пацијент алергичан на било који од састојака IPS Empress Direct-а.

Нежељена дејства
У ретким случајевима, састојци IPS Empress Direct-а могу да изазову преосетљивост. У тим случајевима не треба користити IPS Empress Direct. Да бисте избегли могућу иритацију, подручја у близини пулпе прекрийте одговарајућим средством за заштиту пулпе/дентина (препарат на бази калцијум хидроксида селективно нанесите на подручја око пулпе и обложите одговарајућим лајнером).

Интеракције
Материјали који садрже еугенол/уље каранфилића могу да спрече полимеризацију композитних материјала. Због тога треба избегавати примену таквих материјала заједно са IPS Empress Direct-ом. У додиру са катјонским течностима за испирање уста, средствима за откривање плака и хлорхексидином може да дође до промене боје.

Наношење
1. Одређивање боје
Пре одређивања боје очистите зубе. Глеђна боја се одређује на још влажном зубу. Дентинска боја може се одредити постављањем уметка поред припремљеног зуба или поред цервикалног подручја очњака.
2. Изолација
Потребно је осигурати одговарајућу изолацију, по могућству кофердамом (нпр. OptraDam® Plus).
3. Препарација кавитета
Препарација кавитета врши се према захтевима адхезивне технике, тј. уз чување зубне масе. Глеђне ивице у предњој регији треба искосити. У бочним регијама треба заоблити само оштре глеђне ивице (дијамантским финишерима од 25-40 µm). Цервикални дефекти без каријеса се не припремају, већ само очисте плавцем или другом подесном пастом за чишћење помоћу гумених наставака или обртних четкица. Ако је присутна склероза дентина, површинска додатна обрада округлом бургијом може да поспеши адхезију. На крају се сви остаци уклоне из кавитета ваздушним спрејом. Потом се кавитет исуши ваздушним млазом без примеса воде и уља.
4. Заштита пулпе/подлога
Немојте наносити подлогу ако користите средство за везивање глеђи/дентина. Само веома дубока подручја у близини пулпе селективно прекријте материјалом на бази калцијум хидроксида (нпр. ApexCal®) па нанесите цемент отпоран на притисак (нпр. стаклени јономерни цемент као што је Vivaglass® Liner). Остали зидови кавитета се не покривају да би могли да подупру везу са адхезивом за глеђ/дентин.
5. Постављање матрице/интерденталног клина
Код кавитета у апроксималном подручју примените циркуларну матрицу или делимичну матрицу и клин.
6. Кондиционирање/наношење средства за везивање
Кондиционирање зубне масе и наношење средства за везивање врше се према упутству за употребу производа који употребљавате. Препоручујемо Syntac® или ExciTE® F (уз нагризање фосфорном киселином) или самонагризајући адхезив AdheSE® или AdheSE® One F.
7. Наношење IPS Empress Direct-а
– Пошто је ради добијања различитих анатомских детаља IPS Empress Direct доступан у великом броју дентинских и глеђних боја различите транслуцентности, све технике за слојеве могу се користити на креативан начин како би се постигле рестаурације изузетне естетске вредности. Оптимални резултати постижу се наношењем IPS Empress Direct-а у слојевима не дебљим од 2 mm (или 1,5 mm код дентинских боја) и адаптацијом помоћу нпр. OptraSculpt®/OptraSculpt® Pad.
– Довољна изложеност светлу спречава некомплетну полимеризацију. Пратите препоруке у табели 1 у вези времена осветљивања (Exposure Time) и интензитета светла (Light Intensity). Отвор који емитује светло држите што ближе површини рестауративног материјала.
– Ако користите металну матрицу, након њеног уклањања рестаурацију додатно полимеризујте букално или лингвално/палатално ако не користите полимеризациону лампу Bluephase® или ако светлосну сонду није било могуће поставити у оптималан положај (нпр. велика удаљеност од материјала или дивергентно зрачење).
– У многим случајевима, сада се као први слој користи течни композит како би се уједначило дно кавитета и поједноставила каснија адаптација рестауративног материјала. Употреба течног композита (нпр. Tetric EvoFlow®) као првог слоја није обавезна. Овај слој се мора полимеризовати у засебном кораку (морају се следити инструкције из одговарајућег упутства за употребу).
8. Завршна обрада/провера оклузије/полирање
Након полимеризације, вишак материјала одстраните одговарајућим финишерима (нпр. Astropol® F) или ситнозрним дијамантима. Проксималне вишкове одстраните дијамантским или тунгстен карбидним финишерима, брусним тракама или флексибилним дисковима за полирање. Проверите оклузију и артикулацију па избрусите тако да на површини рестаурација не настану рани контакти или нежељене артикулационе тачке. Исполирајте до трајног високог сјаја помоћу силиконских средстава за полирање (нпр. Astropol® P, Astropol® HP, Astrobrush или OptraPol® Next Generation) и дискова и трака за полирање.

Додатне информације
1. По потреби се директно на полимеризовани материјал може нанети додатни материјал IPS Empress Direct. Уколико је рестаурација IPS Empress Direct-ом већ полирана, пре наношења свежег IPS Empress

Direct-а мора се охрапавити и навлажити Heliobond®-ом.
2. IPS Empress Direct приликом наношења треба да буде на собној температури. Нижа температура отежава истискивање материјала.
3. Само за једнократну употребу. Ако се IPS Empress Direct наноси из Cavifil-а директно у пацијентова уста, након употребе Cavifil се мора бацити како би се спречила унакрсна контаминација пацијената.
4. Шприцеве и инјекторе Cavifil не треба дезинфиковати оксидишућим дезинфекционим средствима.

Упозорење
Неполимеризовани IPS Empress Direct не сме да дође у контакт са кожом, слузокожом и очима. Неполимеризовани IPS Empress Direct може деловати слабо надражујуће и проузроковати преосетљивост на метакрилате. Комерцијалне медицинске рукавице не пружају заштиту од сензибилизације на метакрилате.

Упутство за чување и складиштење
– Чувати на температури од 2 до 28°C.
– Шприцеве/инјекторе Cavifil затворите одмах након употребе. Изложеност светлу доводи до превремене полимеризације.
– IPS Empress Direct се не сме користити по истеку назначеног рока трајања.
– Рок трајања: види натпис на инјекторима Cavifil, шприцевима и паковањима.

Чувати ван домашаја деце.
Само за употребу у стоматологији.

Овај производ је развијен само за употребу у стоматологији. Мора се користити строго према упутству за употребу. Произвођач не преузима одговорност за штете које могу настати због непоштовања упутства за употребу или због неодговарајуће области наношења. Корисник је дужан да испита подесност производа и сноси одговорност за употребу производа у било коју сврху која није изричито наведена у Упутству за употребу. Опис производа и подаци не представљају гаранцију својстава и нису обавезујући.

Македонски

Опис
IPS Empress® Direct е нанохибриден композит според најсовремената технологија, полимеризира на светло и непропустлив е за рентгенски зраци. Соодветен е за изработка на реставрации кои ги исполнуваат највисоките очекувања. IPS Empress Direct полимеризира со светло на бранова должина помеѓу 400–500 nm (сино светло).

Бои
IPS Empress Direct е достапен во 32 различни бои и 5 различни степени на транслуценција. Достапни се следните бои:
Бои на глеѓ
A1, A2, A3, A3.5, A4, B1, B2, B3, B4, C1, C2, C3, D2, D3, Bleach-L, Bleach-XL
Бои на дентин
A1, A2, A3, A3.5, A4, IVA5, IVA6, B1, B2, C3, D2, Bleach-L, Bleach-XL
Транслуцентни бои
Trans 20, Trans 30, Opal*
*пропустлив за рентгенски зраци поради неговата висока проѕирност

Состав
Мономерната матрица се состои од диметакрилати (20-21,5% тежински удел, опалесцентна боја 17% тежински удел). Полначите содржат бариумово стакло, итербиум трифлуорид, мешан оксид, силициум диоксид и кополимер (77,5-79% тежински удел, опалесцентна боја 83% тежински удел). Дополнително содржи адитиви, катализатори, стабилизатори и пигменти (< 1,0% тежински удел). Вкупната содржина на неоргански полначи е 75-79% односно 52-59% волуменски удел (опалесцентна боја 60,5% односно 45% тежински удел). Големината на честичките на неорганските полначи е помеѓу 40 nm и 3000 nm со просечна големина на честичката од 550 nm.

Индикации
– Реставрации на предните заби (класа III, IV)
– Реставрации во задниот дел (класа I и II)
– Реставрации на забниот врат (класа V, нпр. во случај на кариес на забниот врат, ерозија на коренот, клиновидни дефекти)
– Корекција на позицијата и обликот на забот (нпр. затворање на дијастема, затворање на црни триаглести интердентални простори, продолжување на инцизалниот раб)
– Директни винири

Контраиндикации
Поставувањето на реставрации со IPS Empress Direct е контраиндицирано
– ако не може да се обезбеди суво работно поле или не може да се примени пропишаната работна техника
– ако е познато дека пациентот е алергичен на некоја од состојките на IPS Empress Direct

Несакани дејства
Во ретки случаи, составните делови на IPS Empress Direct можат да предизвикаат пречувствителност. Во тој случај IPS Empress Direct не треба да се користи. За да се избегне можна иритација на пулпата, заштитете ги местата блиску до пулпата со соодветно средство за заштита на пулпата/дентинот (на деловите блиску до пулпата селективно нанесете препарат врз база на калциум хидроксид и покријте со соодветен лајнер за кавитетот).

Интеракции
Супстанциите што содржат еугенол/масло од каранфилче ја инхибираат полимеризацијата на композитните материјали. Поради тоа мора да се избегнува примената на тие материјали заедно со IPS Empress Direct. Може да се јави менување на бојата во комбинација со катјонски води за уста, ревелатори на плак и хлорхексидин.

Апликација
1. Одредување на бојата
Пред да ја одредите бојата исчистете ги забите. Одредете ја бојата за глеѓта додека забите се сè уште влажни. Бојата на дентинот може да се одреди директно на препарираниот заб или во пределот на забниот врат кај очникот.
2. Изолација
Потребна е соодветна изолација, најдобро со кофердам (нпр. OptraDam® Plus).

3. Препарација на кавитетот
Препарацијата на кавитетот се врши според правилата на адхезивната техника, т.е. со заштита на што е можно поголем дел од забната структура. Рабовите на глеѓта во предниот дел треба да се накосат. Во задниот дел треба да се заоблат само острите рабови на глеѓта (дијамантски финишери, 25-40 μm). Да не се препарираат дефекти на забниот врат кои се без кариес. Тие само треба да се исчистат со пемза или друга соодветна паста за чистење со помош на гумена капа или ротирачка четкичка. Во случај на склеротичен дентин, адхезивната врска може да се подобри со површинско рекондиционирање со помош на округол борер. Потоа, прекумерните остатоци во кавитетот се плакнат со воден млаз. Кавитетот се суши со воздух без примеси на вода и масло.
4. Заштита на пулпата/подлога
Не користете материјал за подлога кога користите средство за бондирање на глеѓта/дентинот. Покријте ги само многу длабоките кавитети блиску до пулпата со калциум хидроксиден материјал (нпр. ApexCal®), а потоа покријте со цемент стабилен на притисок (нпр. гласјономерен цемент како Vivaglass® Liner). Другите ѕидови на кавитетот да не се покриваат зашто може да помогнат при бондирањето со адхезив за глеѓта/дентинот.
5. Поставување на матрица/интердентално колче
Кај кавитети во проксималниот предел употребете или циркуларна матрица или делумна матрица и поставете колче.
6. Кондиционирање/апликација на средство за бондирање
Кондиционирајте ја забната структура и нанесете го средството за бондирање според упатството за употреба на производот што го користите. Ivoclar Vivadent ја препорачува употребата на Syntac® или ExciTE® F (со нагризување со помош на фосфорна киселина) или самонагризувачкиот адхезив AdheSE® или AdheSE® One F.
7. Апликација на IPS Empress Direct
- Со оглед на тоа што се достапни голем број на бои за дентин и глеѓ на IPS Empress Direct Dentin со различна транслуценција за обликување на анатомските детали, може да се применува било која техника за поставување на слоевите на креативен начин и со тоа да се постигнат високоестетски реставрации. За да се постигнете оптимални резултати, нанесете го IPS Empress Direct во слоеви со максимална дебелина од 2 mm (или 1,5 mm кај боите на дентин) и адаптирајте со соодветен инструмент како нпр. OptraSculpt®/ OptraSculpt® Pad.
- Со доволно изложување на светлото за стврднување се спречува нецелосна полимеризација. Препораките за времето на осветлување (Exposure Time) и интензитет на светлото (Light Intensity) се дадени во табела 1. Држете го прозорчето со светлосниот зрак што е можно поблиску до реставративниот материјал.
- Доколку користите метална матрица, по отстранувањето на матрицата дополнително осветлете го композитниот материјал од букално или лингвално/палатално, ако не се користи Bluephase® светло за полимеризација или сондата со светло не може идеално да се позиционира (нпр. кога е далеку од материјалот или во случај на дивергенција на аголот на распрскување на зракот).
- Во многу случаи сега се користи флуиден композит за првиот слој за да се постигне рамен кавитетен под и за да се олесни адаптацијата на реставративниот материјал што потоа ќе се употребува. Употребата на флуиден композит (нпр. Tetric EvoFlow®) како прв тенок слој е опционална. Овој слој мора да се полимеризира во посебен чекор (да се почитува упатството за употреба за производот што го се користи).

8. Завршна обработка/контрола на оклузијата/полирање
По полимеризацијата, отстранете ги остатоците со соодветни финишери (нпр. Astropol® F) или фино зрнести дијаманти. Отстранете ги проксималните остатоци со дијамантски финишери, тунгстен карбидни финишери, ленти за финирање односно еластични дискови за финирање. Проверете ја оклузијата и артикулацијата, истружете за корекција за да не се создадат рани контакти или несакани артикулациски патеки на површината на реставрацијата. За траен и висок сјај на реставрацијата се врши полирање со силиконски полирачи (нпр. Astropol® P, Astropol® HP, Astrobrush или OptraPol® Next Generation) како и дискови за полирање и ленти за полирање.

Дополнителни информации
1. Ако е потребно, дополнителен IPS Empress Direct може да се нанесе директно на веќе полимеризиран материјал. Ако реставрацијата со IPS Empress Direct е веќе полирана, мора прво да се иструга за да стане рапав и да се навлажни со Heliobond® пред да се нанесе нов свеж слој на IPS Empress Direct.
2. IPS Empress Direct треба да има собна температура додека се нанесува. Студените температури го отежнуваат истиснувањето.
3. Само за еднократна употреба. Доколку IPS Empress Direct се нанесува од Cavifil инјектор директно во устата на пациентот, по употребата Cavifil инјекторот мора да се отстрани во отпад за да се избегне вкрстена контаминација помеѓу пациентите.
4. Да не се дезинфицираат шприцовите или Cavifil инјекторите со оксидирачки средства за дезинфекција.

Предупредување
Неполимеризираниот IPS Empress Direct не смее да дојде во контакт со кожата, слузокожата или очите. IPS Empress Direct во нестврднатата состојба може да делува слабо надразнувачки и да предизвика пречувствителност кон метакрилати. Комерцијалните медицински ракавици не пружаат заштита против сензибилирачкото дејство на метакрилатите.

Чување и складирање
- Да се чува на температура од 2 до 28 °C.
- Затворете ги шприцовите/Cavifil инјекторите веднаш по употребата. Изложувањето на светло предизвикува преранa полимеризација.
- Да не се користи IPS Empress Direct по истекот на наведениот рок на траење.
- Рок на траење: види ги информциите на Cavifil инјекторите, шприцовите и пакувањето.

Материјалот да се чува вон дофат на децата!
Само за употреба во стоматологијата.

Овој производ е развиен само за употреба во стоматологијата. Треба да се користи строго според упатството за употреба. Производителот не презема одговорност за штети што можат да настанат поради непочитување на упатството за употреба или поинакво користење од пропишаното поле на примена. Корисникот е одговорен да го тестира производот во однос на неговата погодност и употреба за било која цел што не е експлицитно наведена во упатството. Описот и податоците не претставуваат гаранција на својствата и не се обврзувачки.

Описание
IPS Empress® Direct е фотополимеризиращ, рентген положителен, нанохибриден материал (композит) на базата на съвременна технология, който е подходящ за реставрации, които отговарят на най-високите изисквания. IPS Empress Direct се полимеризира при светлина с дължина на вълната 400–500 nm (синя светлина).

Разцветки
IPS Empress Direct се предлага в 32 различни разцветки и 5 различни степени на прозрачност.
Предлагат се следните разцветки:
Емайлни разцветки
A1, A2, A3, A3.5, A4, B1, B2, B3, B4, C1, C2, C3, D2, D3, Bleach-L, Bleach-XL
Дентинови разцветки
A1, A2, A3, A3.5, A4, IVA5, IVA6, B1, B2, C3, D2, Bleach-L, Bleach-XL
Прозрачни разцветки
Trans 20, Trans 30, Opal* *Не е рентген положителен поради високата си прозрачност.

Състав
Мономерният матрикс се състои от диметакрилати (20-21,5 тегл. %, опалесценция 17 тегл. %). Пълнежните материали съдържат бариево стъкло, итербиев трифлуорид, смес от окиси, силиконов диоксид и кополимери (77,5-79 тегл. %, опалесценция 83 тегл. %). Допълнително съдържание: добавки, катализатори, стабилизатори и пигменти (<1 тегл. %). Общото съдържание на неорганични пълнители е 75-79 тегл. % или 52-59 обемни % (опалесценция 60,5 тегл. % или 45 обемни %). Големината на частиците на неорганичните пълнители е между 40 nm и 3000 nm, при среден размер на частиците 550 nm.

Показания
- Предни реставрации (клас III, IV)
- Реставрации в задната област (класове I и II)
- Реставрации в зъбната шийка (клас V, напр. в случай на шиечни кариеси, коренна ерозия, клиновидни дефекти)
- Корекции на позицията и формата на зъба (напр. закриване на диастема, закриване на „черни триъгълници", удължаване на инцизния ръб)
- Директни фасети

Противопоказания
Поставянето на IPS Empress Direct реставрации е противопоказано
- ако няма възможност за достатъчно подсушаване или ако предписаната техника не може да бъде приложена
- ако пациентът е с доказана алергия към някой от съставките на IPS Empress Direct

Странични ефекти
В редки случаи, съставки на IPS Empress Direct може да доведат до сенсибилизиране. В тези случаи IPS Empress Direct не трябва да се използва. С цел избягване на евентуално дразнене на пулпата, областите в близост до нея трябва да бъдат предпазени с подходящ протектор за пулпа/дентин (селективно нанесете калциево-хидроксиден препарат в зоните с непосредствена близост с пулпата и покрийте с подходящ лайнер).

Взаимодействия
Вещества, съдържащи еугенол/карамфилово масло, забавят полимеризацията на композитни материали. Следователно, приложението на такива материали заедно с IPS Empress Direct трябва да се избягва. При комбинация с катионни води за освежаване на устата, оцветители на зъбна плака и хлорхексидин може да настъпят цветови промени.

Приложение
1. Определяне на цвета
Преди да се определи цветът, зъбите се почистват. Емайленият цвят се определя докато зъбите са още влажни. Дентиновата разцветка може да се определи или с поставяне на мострата до приготвения зъб или до цервикалната област на кучешките зъби.
2. Подсушаване
Нужно е подходящо подсушаване, най-добре с кофердам (напр. OptraDam® Plus).
3. Подготовка на кавитет
Кавитетът се подготвя съгласно правилата на адхезивната техника, т.е. защита на зъбната структура. Емайлените ръбове в предната област се скосяват. В задната област само острите емайлени ръбове трябва да се заоблят (финиширащи диаманти 25-40 µm). Цервикалните дефекти без кариес не се подготвят, само се почистват с пемза или други подходящи почистващи пасти с помощта на гумени чашки или ротационни четки. При наличие на склеротичен дентин, повърхностно възстановяване с розов борер може да подобри адхезивното свързване. След това отстранете всички остатъци в кавитета с водна струя. Кавитетът се подсушава с обезводнен и обезмаслен въздух.
4. Защита на пулпата / Основа
При използване на свързващо вещество за емайл/дентин да не се прави подложка. Само при много дълбоки кавитети в близост до пулпата покрийте зоната с калциево-хидроксиден препарат (напр. ApexCal®), а след това използвайте устойчив на натиск цимент (напр. глас-йономерен цимент като Vivaglass® Liner). Останалите кухини на стената не се покриват, за да могат да се използват като база за сцепление посредством адхезив за емайл/дентин.
5. Поставяне на матрица / междудентален клин
Използвайте обгръщаща матрица за кавитети, които засягат проксималната област, или секторна матрица и я заклинете.
6. Кондициониране / нанасяне на свързващото вещество
Кондиционирайте зъбната структура и приложете свързващото вещество според ръководството за употреба на продукта, който се използва. Препоръчваме използването на свързващото вещество Syntac® или ExciTE® F (в комбинация с ецване с фосфорна киселина) или самоецващия адхезив AdheSE® или AdheSE® One F.
7. Приложение на IPS Empress Direct
- Тъй като се предлагат голям брой IPS Empress Direct дентинови и емайлови разцветки с различна прозрачност за създаване на анатомичен детайл, всякаква техника на полагане може да бъде креативно използвана за получаване на високо естетични реставрации. За оптимален резултат, положете IPS Empress Direct на слоеве с дебелина макс. 2 mm (или 1,5 mm дентинови разцветки) и адаптирайте, например с OptraSculpt®/OptraSculpt® Pad подложка.

– Достатъчното експониране с полимеризационната светлина предпазва от непълно полимеризиране. За препоръки относно времето на експонация (Exposure Time) и интензитета на светлината (Light Intensity) вижте Таблица 1. Лампата за фотополимеризация трябва да се държи максимално близо до повърхността на реставрационния материал.
– При използване на метална матрица, след отстраняването ѝ е необходимо допълнително полимеризиране на реставрацията от букалната или лингвалната/палатиналната страна, ако не се използва полимеризационна светлина Bluephase® или ако не е възможно оптимално позициониране на светловода (напр. твърде далече от материала или при дивергиращ ъгъл на облъчване).
– В много случаи, за начален слой вече се използва течен композит, за да се създаде равен под на кавитета и да се улесни адаптирането на използвания след това материал за реставрация. Използването на течен композит (напр. Tetric EvoFlow®) като тънък начален слой, е по желание. Този слой трябва да бъде фотополимеризиран в отделна стъпка (трябва да се съблюдават указанията от съответните Инструкции за употреба).

8. Последна обработка / оклузионен контрол / полиране
След полимеризиране отстранете излишните количества материал с подходящи полиращи инструменти (напр. Astropol® F) или фини диаманти. Проксималните излишъци се отстраняват с финиширащи инструменти от диамант, волфрамов карбид, с финиширащи лентички, или с гъвкави финиширащи дискове. Проверете оклузията и артикулацията и нанесете подходящи абразивни корекции, за да се избегнат преждевременни контакти или нежелани артикулационни следи по повърхностите на реставрациите. Използвайте силиконови полиращи дискове (напр. Astropol® P, Astropol® HP, Astrobrush или OptraPol® Next Generation), както и полиращи дискове и полиращи лентички, за да полирате реставрациите до получаване на траен висок гланц.

Допълнителна информация
1. Ако е необходимо, допълнително количество материал IPS Empress Direct може да се приложи направо върху полимеризирания материал. Ако реставрацията с IPS Empress Direct вече е полирана, тя трябва да се надраска и намокри с Heliobond® преди нанасяне на нов слой IPS Empress Direct.
2. Когато се прилага, IPS Empress Direct трябва да бъде на стайна температура. Ниските температури затрудняват екструдирането на материала.
3. Само за еднократна употреба. Ако IPS Empress Direct се прилага директно от Cavifil в устата на пациента, Cavifil трябва да се изхвърли след употреба, за да се предотврати кръстосано замърсяване между пациенти.
4. Спринцовки или Cavifils не трябва да се дезинфектират с окисляващи дезинфектанти.

Внимание
Неполимеризираният IPS Empress Direct не трябва да влиза в контакт с кожа, лигавица или очите. Неполимеризираният IPS Empress Direct може да предизвика леко дразнене и да доведе до сенсибилизация към метакрилати. Предлаганите на пазара медицински ръкавици не осигуряват защита срещу сенсибилизиращия ефект към метакрилати.

Съхранение
– Температура на съхраняване: 2–28 °C.
– Затваряйте спринцовките/Cavafil веднага след употреба. Излагането на светлина може да предизвика преждевременна полимеризация.
– Не използвайте IPS Empress Direct след посочения срок на годност.
– Срок на годност: вижте указаното върху Cavifil, спринцовките и опаковките.

Да се съхранява на недостъпни за деца места!
За употреба само от зъболекари.

Препаратът е разработен само за стоматологична употреба. Да се прилага при стриктно спазване на Инструкциите за употреба. За щети, възникнали от неспазването на инструкциите или приложение различно от указаното, производителят не носи отговорност. Потребителят е длъжен на своя отговорност да провери продуктите за тяхната годност и възможност за използване за предвидените цели, особено ако тези цели не са изрично посочени в информацията за употреба. Описанията и данните не представляват гаранция за свойствата на препарата и не са задължаващи.

Shqip

Përshkrimi
IPS Empress® Direct është një kompozit nano-hibrid, radio-opak, i fotopolimerizueshëm i bazuar në teknologjinë bashkëkohore, i përshtatshëm për përgatitje restaurimesh të standardeve më të larta. IPS Empress Direct polimerizohet me dritë në një interval gjatësie vale prej 400–500 nm (dritë e kaltër).

Ngjyrat
IPS Empress Direct ofrohet në 32 ngjyra të ndryshme dhe në 5 shkallë të ndryshme kthjelltësie.
Ekzistojnë në gjendje ngjyrat vijuese:
Ngjyrat e smaltit
A1, A2, A3, A3.5, A4, B1, B2, B3, B4, C1, C2, C3, D2, D3, Bleach-L, Bleach-XL
Ngjyrat e dentinës
A1, A2, A3, A3.5, A4, IVA5, IVA6, B1, B2, C3, D2, Bleach-L, Bleach-XL
Ngjyrat e kthjellta
Trans 20, Trans 30, Opal*
*Jo radio-opak për shkak të tejdukshmërisë së tij të madhe

Përbërja
Matrica monomerike përbëhet nga dimetakrilate (20–21,5 % e peshës, në ngjyrën opaleshente 17% e peshës). Mbushësit përmbajnë qelq bariumi, trifluorid iterbiumi, oksid të përzier, dioksid silici dhe kopolimer (77,5-79% të peshës, në ngjyrën opaleshente 83% të peshës). Përmban gjithashtu aditivë, katalizatorë, stabilizues dhe ngjyrues (< 1% të peshës). Në tërësi, mbushësit inorganikë përfaqësojnë 75-79% të peshës ose 52-59% të volumit (në ngjyrën opaleshente 60,5% të peshës ose 45% të volumit). Grimcat e mbushësve inorganikë kanë përmasa nga 40 nm deri në 3000 nm, me një madhësi mesatare prej 550 nm.

Indikimi
– Restaurime të përparme (Klasat III, IV)
– Restaurime në rajonin posterior (Klasat I dhe II)
– Restaurime cervikale (Klasa V, p.sh. në rast kariesi cervikal, erozioni radikular, anomalie në formë konoide)
– Korrigjime të pozicionit të dhëmbëve apo të formës së dhëmbëve

(p.sh. mbyllja e diastemës, mbyllja e trekëndëshave të zinj interdentalë, zgjatja e buzës prerëse)
– Fasheta me metodë të drejtpërdrejtë

Kundërindikime
Realizimi i restaurimeve me IPS Empress Direct kundërindikohet
– nëse nuk mund të krijohet një fushë pune e thatë ose nuk mund të zbatohet teknika e parashikuar e aplikimit
– nëse është e njohur një alergji e pacientit ndaj ndonjë prej përbërësve të IPS Empress Direct

Efekte anësore
Në raste të rralla, përbërësit e IPS Empress Direct mund të shkaktojnë mbindjeshmëri. Në të tilla raste, produkti nuk duhet përdorur. Për të shmangur një irritim të mundshëm të pulpës, zonat afër saj duhen ruajtur me anë të një mbrojtësi të përshtatshëm për pulpën/dentinën (zonat në afërsi të pulpës të lyhen sipas nevojës me një preparat me bazë hidroksid kalciumi dhe të mbulohen me një veshje të përshtatshme).

Ndërveprime
Substancat me përbërje eugenoli/vaji karafili mund të pengojnë polimerizimin e materialeve kompozite. Si pasojë, duhet shmangur përdorimi i këtyre materialeve së bashku me IPS Empress Direct. Mund të hasen çngjyrime në rast shoqërimi me losione kationike për shpëlarje goje, me agjentë zbulues për pllakën dhe me klorheksidinë.

Aplikimi
1. Përcaktimi i ngjyrës
Të pastrohen dhëmbët para se të përcaktohet ngjyra. Ngjyra e smaltit duhet përzgjedhur kur dhëmbët janë ende të lagur. Ngjyra e dentinës mund të përcaktohet duke e vendosur çelësin e ngjyrave qoftë në afërsi të dhëmbit të përgatitur qoftë ngjitur me zonën cervikale të kaninit.
2. Izolimi
Nevojitet izolim i përshtatshëm, mundësisht me një ndarëse gome (p.sh. OptraDam® Plus).
3. Përgatitja e kavitetit
Kaviteti përgatitet sipas parimeve të teknikës adezive, d.m.th. duke ruajtur strukturën e dhëmbit. Bisotohen buzët e smaltit në rajonin e përparmë. Në rajonin posterior, duhen rrumbullakosur vetëm buzët e smaltit që janë të mprehta (me freza diamanti prej 25-40 μm). Anomalitë cervikale pa karies nuk përgatiten, vetëm pastrohen me pomiçe ose pastë tjetër pastruese të përshtatshme, me ndihmën e gomave në formë hinke apo të furçave rrotulluese. Në rast se dentina është sklerotike, rikushtëzimi sipërfaqësor me anë të një freze të posaçme do ta përmirësojë lidhjen adezive. Më pas, kaviteti shpëlahet me ujë për të eliminuar të gjitha mbeturinat. Në fund, kaviteti thahet me ajër pa përmbajtje uji apo vaji.
4. Mbrojtja e pulpës / Baza
Të mos vendoset bazë në qoftë se po përdoret një agjent lidhës smalt-dentinë. Vetëm zonat shumë të thella afër pulpës duhet të vishen me një material me hidroksid kalciumi (p.sh. ApexCal®) dhe, më pas, të përdoret cement rezistues ndaj trysnisë (p.sh. cement qelqi jonomerik si p.sh. Vivaglass® Liner). Të mos mbulohet pjesa e mbetur e mureve të kavitetit, në mënyrë që të përdoret për përftimin e lidhjes me një adeziv smalt-dentinë.
5.Vendosja e një matrice / pyke interdentale
Përdorni një matricë mbështjellëse për zgavra që prekin zonën proksimale ose një matricë seksionale dhe pykojeni.
6. Kondicionimi / Aplikimi i agjentit lidhës
Të kondicionohet siç duhet struktura e dhëmbit e të vendoset agjenti lidhës sipas udhëzimeve të përdorimit të produktit. Këshillojmë të përdoret Syntac® ose ExciTE® F (pas ashpërsimit me acid fosforik) ose adezivi vetë-ashpërsues AdheSE® ose AdheSE® One F.
7. Aplikimi i IPS Empress Direct
– Duke qenë që kihen në dispozicion një numër i madh ngjyrash smalti dhe dentine IPS Empress me kthjelltësi të ndryshme, mund të përdoret me origjinalitet çdo lloj teknike shtresimi për krijimin e detajeve anatomike duke krijuar restaurime tepër estetike. Për një rezultat ideal, të aplikohet IPS Empress Direct në shtresa me trashësi maksimumi 2 mm (ose 1,5 mm për ngjyrat dentinal) dhe të modelohet materiali me një instrument të përshtatshëm si p.sh. OptraSculpt®/OptraSculpt® Pad.
– Duke siguruar ekspozim të mjaftueshëm ndaj dritës forcuese, do të parandaloni polimerizimin e paplotë. Për rekomandimet në lidhje me kohën e ekspozimit (Exposure Time) dhe intensitetin e dritës (Light Intensity), shihni Tabelën 1. Gypi emetues i dritës duhet mbajtur sa më afër sipërfaqes së materialit restaurues që të jetë e mundur.
– Nëse përdoret matricë metalike, të kryhet, pas heqjes së matricës, një polimerizim i mëtejshëm nga ana bukale ose nga ana linguale/palatinale, nëse, më parë, nuk është përdorur dritë polimerizuese Bluephase®, ose në qoftë se sonda e dritës nuk mund të pozicionohet në mënyrë ideale (p.sh. kur mbetet larg materialit ose paraqet rrezatim divergjent).
– Në shumë raste, përdoret tashmë një kompozit i rrjedhshëm për shtresën fillestare në mënyrë që të krijohet një dysheme e zgavrës e njëtrajtshme si dhe për të thjeshtuar përshtatjen e materialit restaurues që përdoret më pas. Përdorimi i një kompoziti të rrjedhshëm (si p.sh. Tetric EvoFlow®) si një shtresë fillestare e hollë është opsional. Kjo shtresë duhet të fotopolimerizohet në një fazë tjetër (të ndiqen udhëzimet përkatëse të përdorimit).
8. Lëmimi / Kontrolli i okluzionit / Lustrimi
Pas polimerizimit, të hiqet materiali i tepërt me anë lëmuesish të përshtatshëm (si p.sh. Astropol® F) ose me frezë diamanti të imët. Të hiqen tepricat proksimale me freza lëmuese diamanti, lëmues me karbid tungsteni, shirita lëmues ose disqe lëmuese të përkulshme. Të kontrollohet okluzioni dhe artikulimi dhe të kryhen korrigjimet e duhura me lëmim dhe lustrim për të parandaluar kontakte të parakohshme ose artikulime të gabuara në sipërfaqen e restaurimeve. Të përdoren lustrues silikoni (si Astropol® P, Astropol® HP, Astrobrush ose OptraPol® Next Generation) si dhe disqe lustruese ose shirita lustrues deri sa të arrihet një shkëlqim i fortë dhe i qëndrueshëm.

Informacione të mëtejshme
1. Nëse nevojitet, mund të aplikohet material shtesë IPS Empress Direct drejtpërsëdrejti mbi materialin e polimerizuar. Në qoftë se restaurimi me IPS Empress Direct është lustruar tashmë, ai duhet ashpërsuar dhe lagur me Heliobond® para se të vendoset aty kjo shtresë e re me IPS Empress Direct.
2. Gjatë aplikimit, IPS Empress Direct duhet të ketë temperaturën e ambientit. Temperaturat e ftohta e vështirësojnë nxjerrjen e materialit.
3. Për një përdorim të vetëm. Nëse IPS Empress Direct vendoset me Cavifil drejtpërdrejt në gojën e pacientit, Cavifil-i duhet hedhur

poshtë pas përdorimit për të shmangur kontaminimet e ndërsjella mes pacientëve.
4. Shiringat apo Cavifil-et nuk duhen dezinfektuar me agjentë dezinfenktimi oksidues.

Paralajmërim
Materiali IPS Empress Direct i papolimerizuar nuk duhet të bjerë në kontakt me lëkurën, membranat mukoze ose sytë. Materiali IPS Empress Direct i papolimerizuar mund të shkaktojë një irritim të lehtë dhe të sjellë një mbindjeshmëri ndaj metakrilateve. Dorashkat sanitare në treg nuk ofrojnë mbrojtje ndaj efektit mbindjeshmues të metakrilateve.

Ruajtja
– Temperatura e ruajtjes: 2–28 °C.
– Menjëherë pas përdorimit, shiringat/injektuesit Cavifil duhen mbyllur. Ekspozimi ndaj dritës shkakton polimerizim të parakohshëm.
– Materiali IPS Empress Direct nuk duhet përdorur pas datës së parashikuar të skadimit.
– Afati i skadimit: shih shënimin mbi injektuesit Cavifil, tek shiringat dhe në ambalazhe.

Mbajeni materialin larg fëmijëve.
Vetëm për përdorim stomatologjik.

Ky material është zhvilluar për t'u përdorur vetëm në praktikën dentare. Përpunimi duhet kryer duke ndjekur me rreptësi Udhëzimet e Përdorimit. Nuk do të pranohet asnjë përgjegjshmëri për dëme të shkaktuara nga mosrespektimi i Udhëzimeve ose i zonës së indikuar për aplikim. Është përgjegjësi e përdoruesit që t'i testojë produktet për përshtatshmëri dhe përdorim për çfarëdo qëllimi tjetër të paspecifikuar në mënyrë të hapur në Udhëzimet. Përshkrimet dhe të dhënat nuk ofrojnë ndonjë garanci për atributet dhe nuk janë detyruese.

Română

Descriere
IPS Empress® Direct este un compozit fotopolimerizabil, radioopac, nano-hibrid, bazat pe tehnologie modernă, care este potrivit pentru realizarea de restaurări ce răspund celor mai înalte cerinţe. IPS Empress Direct se solidifică la lumină cu lungime de undă cuprinsă în intervalul 400–500 nm (lumină albastră).

Nuanţe
IPS Empress Direct este disponibil în 32 de nuanţe diferite şi 5 grade diferite de transparenţă.
Sunt disponibile următoarele nuanţe:
Nuanţe de smalţ
A1, A2, A3, A3.5, A4, B1, B2, B3, B4, C1, C2, C3, D2, D3, Bleach-L, Bleach-XL
Nuanţe de dentină
A1, A2, A3, A3.5, A4, IVA5, IVA6, B1, B2, C3, D2, Bleach-L, Bleach-XL
Nuanţe transparente
Trans 20, Trans 30, Opal* *Nu este radioopacă din cauza gradului său ridicat de transparenţă

Compoziţie
Matricea monomerică este compusă din dimetacrilaţi (20-21,5 procente de masă, nuanţă opalescentă 17 procente de masă). Agenţii de umplere sunt reprezentaţi de sticlă de bariu, trifluorură de yterbiu, oxid mixt, dioxid de siliciu şi copolimeri (77,5-79 procente de masă, nuanţă opalescentă 83 procente de masă). Conţinut suplimentar: aditivi, catalizatori, stabilizatori şi pigmenţi (<1,0 procente de masă). Conţinutul total de agenţi de umplere anorganici este de 75-79 procente de masă sau 52-59 procente de volum (nuanţă opalescentă 60,5 procente de masă sau 45 procente de volum). Dimensiunea particulelor agenţilor de umplere anorganici variază de la 40 nm până la 3000 nm, cu o dimensiune medie a particulelor de 550 nm.

Indicaţii
– Restaurări anterioare (clasa III, IV)
– Restaurări în zona posterioară (clasele I şi II)
– Restaurări ale coletului dentar (clasa V, de exemplu în cazul cariilor cervicale, eroziunii rădăcinii, defectelor în formă de pană)
– Corecţii ale poziţiei dinţilor şi ale formei dinţilor (de exemplu închiderea diastemei, închiderea „triunghiurilor negre" interdentare, lungirea marginii incizale)
– Faţetări directe

Contraindicaţii
Aplicarea restaurărilor IPS Empress Direct este contraindicată
– dacă nu se poate asigura un spaţiu de lucru uscat sau dacă nu se poate aplica tehnica de lucru prescrisă
– dacă un pacient este alergic la oricare dintre constituenţii IPS Empress Direct

Efecte secundare
În cazuri izolate, substanţele din compoziţia produsului IPS Empress Direct pot să inducă sensibilizare. IPS Empress Direct nu trebuie utilizat în astfel de cazuri. Pentru a evita posibilele iritaţii pulpare se vor trata zonele apropiate de pulpa dentară cu un material de protecţie pulpo-dentinară adecvat (se va aplica selectiv un preparat pe bază de hidroxid de calciu în zonele apropiate de pulpă şi se va acoperi cu un material adecvat de protecţie).

Interacţiuni
Substanţele care conţin eugenol/ulei de cuişoare pot inhiba procesul de polimerizare al materialelor compozite. Prin urmare, se va evita utilizarea unor astfel de materiale împreună cu IPS Empress Direct. La contactul cu apele de gură cationice, cu indicatorii de placă şi cu clorhexidina pot surveni modificări cromatice.

Tehnica de lucru
1. Determinarea nuanţei
Înainte de a determina nuanţa, curăţaţi dinţii. Selectarea nuanţei de smalţ se va efectua pe dintele încă umed. Nuanţa de dentină poate fi determinată aşezând modelul fie lângă dintele preparat, fie lângă zona cervicală a caninului.
2. Izolarea
Este necesară o izolare corespunzătoare, de preferat cu o digă de cauciuc (de ex., OptraDam® Plus).
3. Prepararea cavităţii
Prepararea cavităţii se efectuează conform cerinţelor impuse de tehnica adezivă, mai exact protejând structura dintelui. Marginile smalţului din regiunea anterioară sunt oblice. În regiunea posterioară, numai marginile de smalţ tăioase trebuie rotunjite (instrumente diamantate de finisare, 25-40 µm). Defectele cervicale care nu prezintă carii nu se prepară, ci doar se curăţă cu piatră ponce sau alte paste de curăţare adecvate, cu ajutorul unor cupe de cauciuc sau al unor perii rotative. În cazul unei dentine sclerotice, recondiţionarea superficială cu o freză globulară poate îmbunătăţi adeziunea. La final se va curăţa cavitatea de resturi cu un jet de apă. Cavitatea se usucă cu aer

degresat şi deshidratat.

4. Protecţia pulpară/obturaţia de bază

În cazul utilizării unui agent de legătură amelo-dentinar, nu efectuaţi obturaţie de bază. Numai în cazul cavităţilor foarte adânci, apropiate de pulpă, această zonă se va acoperi punctual cu un material pe bază de hidroxid de calciu (de ex., ApexCal®), adăugând apoi un strat de ciment rezistent la compresiune (de ex., un ciment ionomer de sticlă, precum Vivaglass® Liner). Nu acoperiţi ceilalţi pereţi intracavitari, pentru a putea fi folosiţi ulterior ca agent de legătură cu un adeziv amelo-dentinar.

5. Aplicarea matricei / penei interdentare

Utilizaţi o matrice circulară pentru cavităţile care afectează zona proximală sau o matrice secţională, apoi fixaţi-o cu pana interdentară.

6. Condiţionarea/aplicarea agentului de legătură

Condiţionarea structurii dentare şi aplicarea agentului de legătură se vor efectua conform instrucţiunilor de utilizare pentru produsul folosit. Recomandăm utilizarea Syntac® sau ExciTE® F (gravaj cu acid fosforic) sau a adezivului cu autogravaj AdheSE® ori AdheSE® One F.

7. Aplicarea IPS Empress Direct

– Întrucât pentru crearea detaliilor anatomice există un mare număr de nuanţe de dentină şi de smalţ ale IPS Empress Direct, cu grade diferite de transparenţă, poate fi utilizată în mod creativ orice tehnică de stratificare pentru a obţine restaurări foarte estetice. Pentru un rezultat optim, aplicaţi IPS Empress Direct în straturi cu grosimea de max. 2 mm (sau de 1,5 mm pentru nuanţele de dentină) şi adaptaţi cu OptraSculpt®/ OptraSculpt® Pad.
– Expunerea suficientă la lumina de polimerizare previne polimerizarea incompletă. Pentru recomandările privind timpul de expunere (Exposure Time) şi intensitatea luminii (Light Intensity) a se vedea tabelul 1. Menţineţi fereastra de emisie a luminii cât mai aproape posibil de suprafaţa materialului restauraţiei.
– Când se utilizează o matrice din metal, se va efectua polimerizarea suplimentară a restaurării din direcţia bucală sau linguală/palatală, după îndepărtarea matricei, dacă nu este utilizată fotopolimerizarea Bluephase® sau dacă emiţătorul de lumină nu poate fi poziţionat optim (de exemplu, la distanţă de materialul compozit sau într-un unghi divergent de radiaţie).
– În multe cazuri, este utilizat în prezent un compozit lichid pentru stratul iniţial, pentru a crea o platformă uniformă a cavităţii şi pentru a simplifica adaptarea materialului de restaurare care este utilizat ulterior. Folosirea unui material compozit lichid (de ex., Tetric EvoFlow®) într-un prim strat subţire este facultativă. Acest strat trebuie fotopolimerizat într-o etapă separată (trebuie respectate instrucţiunile respective din manualul de instrucţiuni).

8. Finisarea/controlul ocluziv/lustruirea

După polimerizare se vor elimina excesele de material cu un finisor (de ex., Astropol® F) sau cu un instrument diamantat cu granulaţie fină. Îndepărtaţi excesul proximal folosind un finisor diamantat, finisoare pe bază de carbură de tungsten, benzi de finisare sau discuri de finisare flexibile. Verificaţi ocluzia şi articulaţia şi efectuaţi corecţiile necesare, pentru a preveni contactul prematur sau striaţii articulare nedorite pe suprafaţa restaurărilor. Utilizaţi pietre siliconice (de ex., Astropol® P, Astropol® HP, Astrobrush sau OptraPol® Next Generation) precum şi cu discuri de finisare şi benzi de finisare, pentru a lustrui restaurările.

Informaţii suplimentare

1. Dacă este necesar, o cantitate suplimentară de material IPS Empress Direct poate fi aplicată direct pe materialul polimerizat. Dacă suprafaţa restaurată cu IPS Empress Direct a fost deja lustruită, ea trebuie să fie sablată şi umezită cu Heliobond® înainte de a aplica un nou strat de IPS Empress Direct.
2. IPS Empress Direct trebuie să aibă temperatura camerei atunci când este utilizat. Temperaturile scăzute fac dificilă extrudarea materialului.
3. Material de unică folosinţă. Dacă IPS Empress Direct este aplicat din injectorul Cavifil direct în cavitatea bucală a pacientului, injectorul Cavifil trebuie eliminat după utilizare pentru a preveni contaminarea încrucişată a pacienţilor.
4. Seringile sau injectoarele Cavifil nu trebuie dezinfectate cu agenţi oxidanţi de dezinfectare.

Avertizare

Evitaţi contactul IPS Empress Direct nepolimerizat cu pielea, mucoasele sau ochii. IPS Empress Direct nepolimerizat poate avea un efect uşor iritant şi poate duce la sensibilizare faţă de metacrilaţi. Mănuşile medicale din comerţ nu protejează împotriva efectului de sensibilizare indus de metacrilaţi.

Mod de păstrare

– Temperatură de păstrare 2-28°C.
– Închideţi seringile/injectoarele Cavifil imediat după utilizare. Expunerea la lumină determină polimerizarea prematură.
– Nu utilizaţi IPS Empress Direct după data de expirare indicată.
– Data de valabilitate: consultaţi informaţiile de pe injectoarele Cavifil, seringi şi ambalaje.

A nu se lăsa materialul la îndemâna copiilor!
Numai pentru uz stomatologic.

Materialul este destinat numai pentru uz stomatologic. Materialul va fi utilizat strict în conformitate cu prezentele instrucţiuni. Producătorul nu îşi asumă responsabilitatea pentru daunele rezultate în urma nerespectării instrucţiunilor sau domeniului de aplicare indicat. Utilizatorul este responsabil pentru testarea compatibilităţii produselor precum şi pentru folosirea acestora în orice alt scop decât cel stipulat în mod explicit în instrucţiuni. Descrierile şi informaţiile nu pot fi invocate drept clauze de garanţie în privinţa caracteristicilor produsului şi nu au caracter de obligativitate.

Русский

Описание

IPS Empress® Direct – это светоотверждаемый рентгеноконтрастный наногибридный композит, базирующийся на современных технологиях, для создания реставраций, соответствующих самым высоким требованиям к эстетике. IPS Empress Direct отверждается светом с диапазоном волны 400–500 нм (синий свет).

Цвета

Мы предлагаем 32 цвета и 5 различных степеней прозрачности.
IPS Empress Direct выпускается в следующих цветах:

Цвета эмали

A1, A2, A3, A3.5, A4, B1, B2, B3, B4, C1, C2, C3, D2, D3, Bleach-L, Bleach-XL

Цвета дентина

A1, A2, A3, A3.5, A4, IVA5, IVA6, B1, B2, C3, D2, Bleach-L, Bleach-XL

Прозрачные цвета
Trans 20 (прозрачный 20), Trans 30 (прозрачный 30),
Opal (опаловый)* * Не является рентгеноконтрастным из-за высокой прозрачности.

Состав
Мономерная матрица состоит из диметакрилатов (20 - 21.5% по весу, опалесцирующий цвет 17% по весу). Наполнитель состоит из бариевого стекла, трифторида иттербия, смешанного оксида, диоксида кремния и сополимера (77.5 - 79% по весу, опалесцирующий цвет 83% по весу). Дополнительно в материале присутствуют присадки, катализаторы, стабилизаторы и пигменты (< 1.0% по весу). Общее содержание неорганического наполнителя составляет 75 - 79% по весу или 52 - 59% по объему (опалесцирующий цвет 60.5% или 45% по объему). Размер первичных частиц неорганического наполнителя составляет от 40 нм до 3000 нм, средняя величина частицы составляет 550 нм.

Показания
– Пломбирование передних зубов (классы III, IV)
– Пломбирование боковых зубов (классы I и II)
– Пломбы в пришеечной области (классы V, например, при кариесе в пришеечной области, эрозии корней, клиновидных дефектах)
– Корректировки положения и формы зубов (например, закрытие диастемы, закрытие межзубных треугольников, продление режущего края)
– Изготовление виниров прямым методом

Противопоказания
Применение IPS Empress Direct противопоказано:
– если невозможно обеспечение необходимой сухости рабочего поля или соблюдение предписанной технологии работы
– при известной аллергии на компоненты IPS Empress Direct

Побочное действие
Компоненты материала IPS Empress Direct в редких случаях могут приводить к возникновению чувствительности. В подобных случаях от дальнейшего применения материала следует отказаться.
Чтобы исключить вероятность воспаления пульпы, близкие к пульпе области защитить соответствующим образом (точечно нанести препарат, содержащий гидроксид кальция, а затем закрыть прокладочным материалом). Материалы, содержащие эвгенол или гвоздичное масло, могут препятствовать отверждению композитов. Следует избегать применения таких материалов в сочетании с IPS Empress Direct. При применении катионной воды для полоскания рта, препаратов, способствующих образованию зубного налета, а также хлоргексидина могут появиться цветоизменения.

Применение
1. Определение цвета
Перед определением цвета зубы почистить. Цвет эмали определяется на еще влажных зубах. Цвет дентина может определяться прямо на препарированном зубе или на пришеечной области клыков.
2. Обеспечение сухости
Необходимо обеспечить достаточную сухость рабочего поля, лучше всего с помощью коффердама (например, OptraDam® Plus).
3. Препарирование полости
Препарирование полости проводится по правилам работы адгезивной техникой, т.е. в максимально щадящем режиме для тканей зуба. На фронтальных зубах край эмали выполнять со скосом, в области боковых зубов только слегка сточить острые края эмали или скруглить их (алмазными финирами, 25–40 мкм). Дефекты в пришеечной области, не пораженной кариесом, не препарируются, а только очищаются с помощью пемзы или другой соответствующей чистящей пасты, резиновой чашечкой или вращающейся щеточкой. В случае некротического дентина поверхностная обработка шаровидным бором может улучшить адгезивную связь. Затем все остатки удаляются из полости водяным спреем, а полость высушивается воздухом, очищенным от воды и масла.
4. Защита пульпы / Прокладка
При использовании адгезива эмаль-дентин от использования прокладки следует отказаться. Только в случае очень глубоких, близких к пульпе полостей, точечно закрыть эту область препаратом, содержащим гидроксид кальция (например, ApexCal®), а затем нанести слой цемента, устойчивого к нагрузкам (например, стеклоиономерный цемент, как Vivaglass® Liner). Оставшиеся стенки полости не закрывать, поскольку они будут нужны для создания связи адгезивом эмаль-дентин.
5. Установка матриц / межзубных клинышков
При работе с полостями с аппроксимальной частью использовать либо циркулярную матрицу либо частичную матрицу, и клинышки.
6. Подготовка / Нанесение адгезива
Подготовку и нанесение адгезива проводить в соответствии с инструкцией к используемым материалам. Ivoclar Vivadent рекомендует использовать адгезив Syntac® или ExciTE® F (в сочетании с протравкой фосфорной кислотой) или самопротравливающие адгезивы AdheSE® или AdheSE® One F.
7. Нанесение IPS Empress Direct
– Поскольку материал IPS Empress Direct предлагает большое количество цветов дентина и эмали, с различной степенью прозрачности, а также транслюцентные цвета, существует масса возможностей для создания тончайших нюансов и особенностей. Для достижения оптимального результата наносите IPS Empress Direct слоями макс. толщиной 2 мм (или оттенки Dentin 1,5 мм) и выравнивайте при помощи, например OptraSculpt®/OptraSculpt® Pad.
– Достаточная экспозиция светополимеризационным приборам предотвращает неполное твердение материала.Рекомендации относительно времени полимеризации (Exposure Time) и интенсивности света (Light Intensity) см. Таблица 1. Держите окошко излучения света как можно ближе к поверхности реставрационного материала.
– При использовании металлической матрицы дополнительно полимеризуйте реставрационный материал с буккальной или лингвальной/палатинальной стороны после удаления матрицы, если не применялся полимеризатор Bluephase® или если было невозможно оптимально расположить световой зонд (например, далеко от материала или рассеивающееся излучение).
– В настоящее время во многих случаях используется текучий композит для первого слоя, чтобы создать ровное дно полости для упрощения адаптации реставрационного материала, используемого далее. Применение текучего композита (напр., Tetric EvoFlow®) в качестве первого слоя не является обязательным. Этот слой должен отверждаться светом отдельным этапом (необходимо соблюдать соответствующие инструкции по применению).

8. Обработка, контроль окклюзии, полировка
После полимеризации излишки удалить соответствующим финиром (например, Astropol® F) или мелкозернистым алмазом. Излишки с аппроксимальной стороны удалить с помощью алмазных финиров, твердосплавных полировочных боров, финирующих полосок либо гибких финирующих дисков. Проверить окклюзию и артикуляцию, при необходимости корректировать до исчезновения преждевременных контактов и иных нежелательных препятствий артикуляции на поверхности пломбы. Окончательная полировка производится силиконовыми полирами (например, Astropol® P, Astropol® HP, Astrobrush или OptraPol® Next Generation), а также полировочными дисками и штрипсами.

Особые рекомендации
1. При выполнении корректировок свежую порцию IPS Empress Direct можно наносить на уже сполимеризованный материал. Если пломба из материала IPS Empress Direct уже отполирована, то ей сначала нужно придать шероховатость, увлажнить Heliobond®, а затем наносить новый слой IPS Empress Direct.
2. IPS Empress Direct при работе должен иметь комнатную температуру. Извлечение материала из шприца после хранения в холодильнике может быть затруднено.
3. Только для одноразового применения. Если материал IPS Empress Direct наносят из кавифила непосредственно в полость зуба, из соображений гигиены рекомендуется использовать этот кавифил только для одного пациента (во избежание перекрестной инфекции среди пациентов).
4. Не проводить дезинфекцию шприцов или кавифилов оксидирующими дезинфекционными средствами.

Меры предосторожности:
Избегать контакта несполимеризованного IPS Empress Direct с кожей/слизистой оболочкой и глазами.
IPS Empress Direct в неотвержденном состоянии может оказывать легкое раздражающее действие и может привести к чувствительности на метакрилаты. Обычные медицинские перчатки не обеспечивают защиты от чувствительности к метакрилатам.
Условия хранения:
– Температура хранения 2–28 °C.
– шприцы/кавифилы закрывать сразу же после использования. Проникновение света ведет к преждевременной полимеризации
– Не используйте IPS Empress Direct после указанного на упаковке срока годности.
– срок годности: см. на кавифиле, шприце/упаковке

Хранить в месте, недоступном для детей!
Только для применения в стоматологии!

Этот материал разработан исключительно для применения в стоматологии и должен использоваться строго в соответствии с инструкцией по применению. Производитель не несет ответственности в случае использования материала с нарушением инструкции или в непредусмотренной области применения. Потребитель несет ответственность за тестирование материала на пригодность его применения для любых целей, прямо не указанных в инструкции. Описания и приведенные данные не являются гарантией свойств материала и не являются обязательными.

Українською

Опис
IPS Empress® Direct - це фотополімерний рентгенконтрастний наногібридний високотехнологічний композит для виготовлення пломб найвищої якості. Композит IPS Empress Direct полімеризується світлом із довжиною хвилі 400-500 нм (синє світло).

Кольори
Композит IPS Empress Direct доступний у 32 відтінках кольору із 5 ступенями прозорості. Доступні такі відтінки кольору:
Відтінки кольору емалі
A1, A2, A3, A3.5, A4, B1, B2, B3, B4, C1, C2, C3, D2, D3, Bleach-L, Bleach-XL
Відтінки кольору дентину
A1, A2, A3, A3.5, A4, IVA5, IVA6, B1, B2, C3, D2, Bleach-L, Bleach-XL
Прозорі відтінки кольору
Trans 20, Trans 30, Opal* 	*Рентген неконтрастний через високий ступінь прозорості

Склад
Мономірна матриця складається з діметилакрилатів (20-21,5% ваги, опалесцуючий відтінок кольору становить 17% ваги). Наповнювач складається з барієвого скла, трифториду ітербію, змішаного оксиду, діоксиду кремнію та сополімеру (77,5-79% ваги, опалесцуючий відтінок кольору становить 83% ваги). Також до складу входять додаткові компоненти: добавки, каталізатори, стабілізатори та пігменти (< 1,0% ваги). Загальний вміст неорганічних наповнювачів становить 75-79% ваги або 52-59% від об'єму (опалесцуючий відтінок кольору становить 60,5% ваги або 45% від об'єму). Розмір часток неорганічного наповнювача становить від 40 нм до 3000 нм із середнім розміром частки в 550 нм.

Показання
– Реставрація зубів у передньому відділі (клас III, IV)
– Реставрація зубів у задньому відділі (клас I, II)
– Реставрація шийки зуба (клас V, наприклад, за наявності пришийкового карієсу, ерозії кореня, клиноподібних дефектів)
– Корекція положення та форми зуба (наприклад, за наявності діастеми, рецесії або інтердентальних "чорних трикутників", подовження ріжучого краю)
– Установлення прямих вінірів

Протипоказання
Застосування композиту IPS Empress Direct для відновлення має певні протипоказання
– За неможливості осушення робочої поверхні або неможливості використання зазначеної методики
– Якщо відомо, що у пацієнта алергія на якісь компоненти IPS Empress Direct

Побічні явища
Компоненти IPS Empress Direct можуть лише в окремих випадках призводити до підвищення чутливості. За таких ситуацій композит IPS Empress Direct не слід використовувати. З метою уникнення можливого подразнення пульпи, місця, розташовані поблизу неї, слід захищати, накривати за допомогою відповідного матеріалу (наприклад, на розташовані близько від пульпи місця можна вибірково нанести препарат із гідроксидом кальцію та встановити підходящу прокладку).

Взаємодія з іншими препаратами
Речовини, що містять евгенол або гвоздичну олію, можуть перешкоджати полімеризації композитних матеріалів. Тому слід уникати одночасного застосування таких матеріалів із композитом IPS Empress Direct. Під час використання препарату з катіонактивними рідинами для полоскання рота, із засобами для візуалізації зубного нальоту або хлоргексидину може змінитися колір.

Застосування
1. Визначення кольору
Перед визначенням кольору слід почистити зуби. Відтінок кольору емалі визначається на вологих зубах. Відтінок кольору дентину визначають накладанням таблички поруч із підготовленим зубом або біля пришийкової області ікла.
2. Ізоляція
Необхідно добре висушити робочу поверхню. Це найкраще зробити за допомогою коффердама (наприклад, OptraDam® Plus).
3. Препарування каріозної порожнини
Препарування каріозної порожнини виконується відповідно до вимог адгезивної техніки, тобто із забезпеченням захисту структури зуба. Краї емалі в передній області стинаються. У задній області гострі краї емалі тільки закругляються (фінішним алмазним бором 25-40 нм). Некаріозні дефекти пришийкової області не препаруються, а тільки очищаються пемзовою або іншою підходящою очищувальною пастою за допомогою гумових чашечок або обертальних щіток. За наявності склеротизованого дентину поверхневе шліфування з використанням буру типу "rose" може поліпшити зчеплення з адгезивом. Після цього порожнину слід очистити від залишків за допомогою зрошувача. Порожнину слід осушити за допомогою повітря без домішок води або олії.
4. Захист пульпи / Основа
Від прокладок можна відмовитися, якщо на емаль і дентин наносити адгезивний матеріал. У дуже глибоких порожнинах або на ділянках, близьких до пульпи, необхідно матеріал на основі гідроксиду кальцію (наприклад, ApexCal®), а потім - стійкий до навантажень цемент (склоіономірний, наприклад, Vivaglass® Liner). Інші стінки порожнини закривати не потрібно, тому що пізніше потрібно буде наносити адгезійний матеріал на емаль та дентин.
5. Нанесення матриці / міжзубної розпірки
Під час роботи з каріозними порожнинами у проксимальній частині, потрібно використовувати циркулярну або часткову матрицю, закріпляючи її розпірками.
6. Підготовка / нанесення адгезиву
Підготовку поверхні зуба й нанесення адгезиву потрібно здійснювати відповідно до інструкцій з використання цього продукту. Рекомендовано використовувати адгезиви Syntac® чи ExciTE® F (протравлення фосфорною кислотою) або AdheSE® чи AdheSE® One F (самостійне протравлення).
7. Застосування композиту IPS Empress Direct
– Через доступність великої кількості відтінків кольору та ступенів прозорості композиту IPS Empress Direct для емалі та дентину, що використовується для відтворення анатомічної структури, для забезпечення високоестетичних реставрацій можна широко варіювати техніки пошарового накладання. Для забезпечення оптимального результату композит IPS Empress Direct слід наносити шарами, товщиною не більше 2 мм (для дентину не більше 1,5 мм), вирівнюючи його за допомогою відповідного інструменту (наприклад, OptraSculpt®/ OptraSculpt® Pad).
– Достатня експозиція до світлу для отвердження попереджує неповну полімеризацію. Дивіться Таблицю 1 для інформації щодо часу експозиції (Exposure Time) та інтенсивності світла (Light intensity). Утримуйте вікно емісії світла якнайближче до реставраційного матеріалу.
– Якщо використовується металева матриця, після її вилучення слід додатково полімеризувати реставрацію із щічної або язикової/палатальної проекції, якщо не застосовувалася програма полімеризації Bluephase®, або якщо лампу світлової полімеризації не вдається розмістити належним чином (наприклад, вона віддалена від матеріалу або її промінь дивергентний).
– Для створення рівного дна порожнини часто використовується рідкотекучий композит. Такий початковий шар значно спрощує вирівнювання шарів реставраційного матеріалу, що наноситься зверху. Використання рідкотекучого композиту (наприклад, Tetric EvoFlow®) як початкового шару опціональне. Цей шар слід полімеризувати окремо (докладніше див. відповідні інструкції з використання).
8. Обробка / Перевірка прикусу / Полірування
Видаліть надлишки за допомогою відповідних фінірів (наприклад, Astropol® F) або дрібнозернистих алмазних полірів. Надлишки з проксимального боку також видаляються. Це можна зробити за допомогою алмазних, вольфрамо-карбідних фінірів або фінірних смужок чи гнучких фінірних дисків. Перевірте прикус і артикуляцію, а потім виконайте відповідну корекцію шліфуванням для запобігання небажаних контактів з поверхнею реставрації. Фінішне полірування реставрації здійснюється за допомогою силіконових полірів (наприклад, Astropol® P, Astropol® HP, Astrobrush або OptraPol® Next Generation), а також поліруючих дисків і смужок.

Додаткова інформація
1. За потреби додатковий матеріал IPS Empress Direct можна застосувати безпосередньо на полімеризуючий матеріал. Якщо реставрацію IPS Empress Direct вже відполіровано, перед нанесенням свіжої порції матеріалу IPS Empress Direct її слід зробити шершавою та зволожити за допомогою Heliobond®.
2. Застосовуваний матеріал IPS Empress Direct має бути кімнатної температури. Низькі температури призводять до утруднення вивільнення матеріалу.
3. Лише для одноразового використання.Якщо композит IPS Empress Direct наноситься за допомогою кавіфіла безпосередньо в ротову порожнину пацієнта, після використання кавіфіл слід утилізувати для запобігання можливої перехресної контамінації пацієнтів.
4. Шприци й кавіфіли не можна дезінфікувати з використанням окислюючих агентів.

Запобіжні заходи
Слід уникати контакту неполірмеризованого композиту IPS Empress Direct зі шкірою, слизовою оболонкою та очами. Незатверділий композит IPS Empress Direct може мати дещо подразливу дію і викликати чутливість до метакрилатів. Звичайні медичні рукавички не забезпечують захисту від сенсибілізуючої дії метакрилату.

Вказівки щодо зберігання
– Температура зберігання: 2–28 °C
– Шприци/кавіфіли слід закривати одразу після використання. Проникнення світла може призвести до передчасного затвердіння.
– Не використовуйте композит IPS Empress Direct після завершення зазначеного терміну придатності
– Строк зберігання без використання: див. відповідну інформацію на кавіфілах, шприцах і упаковках.

Зберігати в недоступному для дітей місці!
Використовувати тільки в зуболікувальних цілях!

Цей продукт розроблений для використання в зуболікувальній галузі й має використовуватися тільки відповідно до інформації щодо його використання. Виробник не несе відповідальності за збитки, спричинені використанням з іншою метою та такі, що спричинилися внаслідок невідповідного використання. Користувач повинен під свою відповідальність перевірити продукцію перед використанням на предмет її придатності та можливостей використання в цілях, які не були чітко вказані в інструкції з використання. Описи й наведені дані не містять гарантій властивостей матеріалу.

Eesti keel

Kirjeldus
IPS Empress® Direct on valguskõvastuv röntgenkontrastne nanohübriidkomposiit, mis põhineb tipptehnoloogial, mis sobib keerukaimate restoratsioonide tegemiseks. IPS Empress Direct kõvastub valguse käes, mille lainepikkus on 400–500 nm (sinine valgus).

Värvitoonid
IPS Empress Direct on saadaval 32 värvitoonis ja viies läbipaistvuse astmes. Saadaval on järgmised värvitoonid.
Emaili värvitoonid:
A1, A2, A3, A3.5, A4, B1, B2, B3, B4, C1, C2, C3, D2, D3, Bleach-L, Bleach-XL
Dentiini värvitoonid:
A1, A2, A3, A3.5, A4, IVA5, IVA6, B1, B2, C3, D2, Bleach-L, Bleach-XL
Läbipaistvad varjundid
Trans 20, Trans 30, Opal*

* Suure läbipaistvuse tõttu pole röntgenkontrastne

Koostis
Monomeermaatriks koosneb dimetakrülaatidest (20–21,5 massiprotsenti, röntgenkontrastne varjund 17 massiprotsenti). Täidised sisaldavad baariumklaasi, üterbiumtrifluoriidi, segatud oksiidi, ränidioksiidi ja kopolümeeri (77,5–79 massiprotsenti, röntgenkontrastne varjund 83 massiprotsenti). Lisakoostisained: lisaained, katalüsaatorid, stabilisaatorid ja pigmendid (< 1,0 massiprotsenti). Anorgaaniliste täidiste kogusisaldus on 75–79 massiprotsenti või 52–59 mahuprotsenti (röntgenkontrastne varjund 60,5 massiprotsenti või 45 mahuprotsenti). Anorgaaniliste täidisosakeste suurus on 40–3000 nm. Keskmine osakese suurus on 550 nm.

Näidustused
– Esihammaste täidised (III, IV klass)
– Täidised molaaridel ja premolaaridel (I ja II klass)
– Hambakaela restoratsioonid (V klass, nt kaelaosa kaariese, uure erosiooni, kiilukujuliste defektide puhul)
– Hammaste asendi ja kuju korrektsioonid (nt diasteemia sulgemine, hammaste vahelise nn musta kolmnurga sulgemine, lõikeserva pikendamine)
– Otsesed katmised

Vastunäidustused
IPS Empress Directi restoratsioonide paigaldamine on vastunäidustatud järgmistel juhtudel:
– kui pole võimalik hoida tööpiirkonda kuivana või kasutada ettekirjutatud metoodikat
– kui patsiendil on teadaolev allergia materjali IPS Empress Direct suhtes.

Kõrvaltoimed
IPS Empress Directi koostisosad võivad harvadel juhtudel esile kutsuda ülitundlikkust. Sellistel juhtudel tuleb IPS Empress Directi edasisest kasutamisest kohe loobuda. Et pulbi ärritust vältida, tuleb pulbilähedasi alasid sobiva pulbi- või dentiinikaitsematerjaliga katta (aplitseerida pulbi lähedale kaltsiumhüdroksiidi alusel materjali).

Koostoimed
Eugenooli/nelgiõli sisaldavad ained võivad põhjustada komposiitmaterjalide polümerisatsiooni häireid. Seepärast tuleks selliste materjalide kasutamist koos IPS Empress Directiga vältida. Katioone sisaldavate suuloputusvee, hambakatu relevaatorite ja kloorheksidiiniga kokku puutudes võivad tekkida värvimuutused.

Materjaliga töötamine
1. Värvi valik
Enne värvi valimist tuleb hambad puhastada. Emaili värvitoon määratakse veel niiskel hambal. Dentiini varjundi saate kas lipiku asetamisel prepareeritud hamba või silmahamba kaelapiirkonna kõrvale.
2. Isoleerimine
Vajalik on piisav isoleerimine, soovitatavalt kofferdami kasutades (nt OptraDam® Plus).
3. Kaviteetide prepareerimine
Põhimõtteliselt peaks kaviteete prepareerima minimaalselt, järgides adhesiivse täitmise tehnikat ja säilitades tervet hambakude. Esihammaste piirkonnas prepareeritakse emaili ääred nurga all. Tagahammastel ümardatakse emaili servad (viimistlusteemandid, 25–40 µm). Kaariesest vabu hambakaela defekte ei prepareerita, vaid ainult puhastatakse pimsskivi pastaga või mõne sobiva puhastuspastaga, kummiotsaku või pöördharjakesega. Sklerootilise dentiini puhul võib seondumist parandada pinna töötlemine ümara otsaga puuri abil. Seejärel eemaldatakse kõik jäägid kaviteedis veepihusti abil. Kaviteet kuivatatakse vee- ja õlivaba õhuga.
4. Pulbikaitse/alustäidis
Adhesiivsüsteemi järgimisel pole alustäidist vaja teha. Ainult väga sügavate kaviteetide puhul kaetakse pulbilähedane ala kaltsiumhüdroksiidi preparaadiga (nt ApexCal®) ja lisatakse õhuke survekindla tsemendi kiht (nt klaasionomeert-sement, nagu Vivaglass®Liner). Ülejäänud kaviteedi seinu ei kaeta, et neid saaks töödelda emaili ja dentiini adhesiividega.
5. Maatriksi / hammastevahelise kiilu asetamine
Proksimaalset piirkonda mõjutavate kaviteetide puhul kasutage ringmatriitsi või osalist matriitsi ja kiilu.
6. Konditsioneerimine / sideaine aplitseerimine
Hamba struktuuri konditsioneerimine ja sideaine aplitseerimine toimub iga kasutatava toote kasutamisjuhendi järgi. Me soovitame kasutada isesöövitavaid adhesiive Syntac® või ExciTE® F (fosforhappega söövitav) või AdheSE® või AdheSE® One F.

7. IPS Empress Directi aplitseerimine

– Kuna IPS Empress Directi on saada mitmesuguste dentiini ja emaili varjunditega ja mitmesuguse läbipaistvusega anatoomiliste detailide loomiseks, saab igasugust katmistehnikat kasutada loominguliselt esteetilise restoratsiooni saavutamiseks. Optimaalse tulemuse saamiseks aplitseerige IPS Empress Directi maksimaalselt 2 mm kihtidena (dentiini varjundite puhul 1,5 mm kihtidena) ja adapteerige nt padjaga OptraSculpt®/OptraSculpt® Pad.
– Küllaldane kõvendav valgus väldib mittetäielikku polümerisatsiooni. Soovitusi toimeaja (exposure time) ja valguse võimsuse (light intensity) kohta vaadake Tabel 1. Hoidke valguse emissiooni aken restoratsioonimaterjalile võimalikult lähedal.
– Kui Bluephase®-i valguskõvastamist ei kasutatud või kui valgusandurit ei saanud optimaalselt paigutada (nt see oli materjalist liiga kaugel või langes kiirgus halva nurga all), polümeriseerige metallmatriitsi kasutamisel restoratsioon pärast matriitsi eemaldamist ka bukaalsel ja lingvaalsel/palataalsel küljel.
– Paljudel juhtudel kasutatakse voolavat komposiiti nüüd esmase kihi puhul, et saada ühtlane kaviteedipõhi ja lihtsustada järgmisena kasutatava restoratiivse materjali kohanemist. Võib kasutada ka voolavat komposiiti (nt Tetric EvoFlow®) esmase õhukese kihina. Seda kihti tuleb valgusega töödelda eraldi etapis (järgida tuleb vastava toote kasutusjuhendit).

8. Viimistlemine / oklusiooni kontroll / poleerimine

Pärast polümeriseerimist eemaldatakse ülejäägid sobivate viimistlejatega (nt Astropol® F) või sametteemantidega. Eemaldage proksimaalselt liigne täidis viimistlemiseks mõeldud teemantpuuriga, volframkarbiidpuuriga, viimistlusribade või painduvate viimistluskettastega. Oklusioon ja ühendused kontrollitakse ja lihvitakse, et ei tekiks varajasi kokkupuuteid või soovimatuid ühendusjooni täidise pealispinnal. Poleerige restoratsioonid säravaks, kasutades silikoonpoleerijaid (nt Astropol® P, Astropol® HP, Astrobrush või OptraPol® Next Generation), samuti poleerimiskettaid ja -ribasid.

Lisateave

1. Vajaduse korral saab materjali IPS Empress Direct asetada otse polümeriseeritud materjalile. Kui IPS Empress Directi restoratsiooni on juba poleeritud, tuleb seda kõigepealt karestada ja niisutada materjaliga Heliobond® ja alles siis aplitseerida IPS Empress Direct.
2. IPS Empress Direct peab aplitseerimise ajal olema toatemperatuuril. Kui materjal on jahe, on seda raske välja pressida.
3. Ainult ühekordseks kasutamiseks. Kui IPS Empress Direct aplitseeritakse Cavifilist otse patsiendi suus, tuleb Cavifil pärast kasutamist hävitada, et vältida ristsaastumist patsientide vahel.
4. Süstlaid või Cavifile ei tohiks töödelda oksüdeerivate desinfektsioonivahenditega.

Ettevaatusabinõud

Mittekõvastunud IPS Empress Directi sattumist silma, nahale ja limaskestadele tuleb vältida. Vedelas olekus võib IPS Empress Direct mõjuda ärritavalt ja tekitada ülitundlikkust metakrülaatide suhtes. Kaubandusvõrgus olevad meditsiinilised kindad ei paku piisavat kaitset tundlikkuse korral metakrülaatide suhtes.

Hoidmine

– Hoida temperatuuril: 2–28 °C.
– Sulgege süstlad/Cavifilid kohe pärast kasutamist. Valguse mõju põhjustab enneaegse polümerisatsiooni.
– IPS Empress Directi ei tohi kasutada pärast kõlblikkusaja lõppu.
– Kõlblikkusaeg: vt Cavifilidel, süstaldel ja pakendil olevat teavet.

Hoida lastele kättesaamatus kohas!
Kasutamiseks ainult hambaravis!

Materjal on mõeldud ainult hambaravis kasutamiseks. Toodet tuleb käsitseda rangelt toote kasutusjuhendi järgi. Garantii ei kehti, kui kahjustus on tulenenud kasutusjuhendi või kasutusala eiramisest. Kui tooteid ei kasutata juhendi kohaselt, vastutab nende sobivuse ja kasutamise eest kasutaja. Kirjeldus ja andmed ei tähenda mingit garantiid omadustele ega ole siduvad.

Latviski

Apraksts

IPS Empress® Direct ir gaismā cietējošs, rentgenstarojumu necaurlaidīgs nano hibrīda kompozītmateriāls, kurā izmantotas modernākās tehnoloģijas, un šis materiāls ir piemērots, lai izveidotu augstākajām prasībām atbilstošas restaurācijas. Kompozītmateriāls IPS Empress Direct sacietē gaismā ar viļņa garuma diapazonu 400–500 nm (zilā gaisma).

Toņi

IPS Empress Direct ir pieejams 32 dažādos toņos un 5 dažādos caurspīdīguma līmeņos. Ir pieejami šādi toņi:

Emaljas toņi
A1, A2, A3, A3.5, A4, B1, B2, B3, B4, C1, C2, C3, D2, D3, Bleach-L, Bleach-XL

Dentīna toņi
A1, A2, A3, A3.5, A4, IVA5, IVA6, B1, B2, C3, D2, Bleach-L, Bleach-XL

Caurspīdīguma toņi
Trans 20, Trans 30, opāls* *Nav starojumu necaurlaidīgs, jo ir augsts caurspīdīguma līmenis.

Sastāvs

Monomēra matrica sastāv no dimetilakrilātiem (20-21,5% mitruma satura, opalescējošs tonis 17% mitruma satura). Pildvielu sastāvā ir bārija stikls, iterbija trifluorīds, jauktais oksīds, silikona dioksīds un kopolimērs (77,5-79% mitruma satura, opalescējošs tonis 83% mitruma satura). Papildu sastāvs: piedevas, katalizatori, stabilizatori un krāsvielas (< 1 mitruma procentu). Neorganiskās pildvielas veido 75–79% mitruma satura vai 52–59% tilpuma no kopējā satura (opalescējošs tonis 60,5% mitruma satura vai 45%). Neorganisko pildvielu daļiņu lielums ir no 40 nm līdz 3000 nm ar vidējo graudiņu lielumu 550 nm.

Indikācija

– Zobu priekšējās daļas restaurācijas (III, IV klase)
– Zobu aizmugurējās daļas restaurācijas (I un II klase)
– Zobu kakliņa restaurācijas (V klase, piemēram, cervikālais kariess, saknes erozija, ķīļveida bojājumi)
– Zobu stāvokļa un zobu formas korekcijas (piemēram, diastēmas aizpildīšana, starpzobu "melno trīsstūru" aizpildīšana, zobu augšmalas pagarināšana)
– Direct venīri

Kontrindicēts

IPS Empress Direct restaurāciju novietošana ir kontrindicēta

– ja nevar nodrošināt sausu operācijas lauku vai nevar izmantot noteiktus darba paņēmienus;
– ja pacientam ir zināmas alerģiskas reakcijas pret kādu no IPS Empress Direct sastāvdaļām.

Blakusiedarbība
Retos gadījumos IPS Empress Direct sastāvdaļas var izraisīt jutīgumu. Šādos gadījumos IPS Empress Direct nedrīkst lietot. Lai novērstu iespējamu pulpas kairinājumu, pulpas tuvumā esošās zonas jāaizsargā ar atbilstošu pulpas/dentīna aizsargmateriālu (selektīvi uzklājiet pulpas tuvumā esošajām zonām sagatavi uz kalcija hidroksīda bāzes un nosedziet ar atbilstošu pārklājumu).

Mijiedarbība ar citām vielām
Vielas, kas satur eigenolu/krustnagliņu eļļu, var kavēt kompozītmateriālu polimerizāciju. Tādēļ jāizvairās no šādu materiālu izmantošanas kopā ar kompozītmateriālu IPS Empress Direct. Lietojot kopā ar katjonu mutes dobuma skalošanas līdzekļiem, aplikuma likvidēšanas līdzekļiem un hlorheksidīnu, var būt novērojama krāsas maiņa.

Lietošana
1. Toņa noteikšana
Pirms toņa noteikšanas iztīriet zobus. Emaljas tonis tiek noteikts, kamēr zobs vēl ir mitrs. Dentīna toni var noteikt vai nu novietojot krāsu skalu blakus zoba sagatavei, vai blakus acu zoba cervikālai zonai.
2. Izolēšana
Nepieciešama atbilstoša izolēšana, vēlams ar koferdamu (piem., OptraDam® Plus).
3. Kavitātes sagatavošana
Kavitāte tiek sagatavota atbilstoši adhezīva uzklāšanas tehnikas prasībām, t. i., aizsargājot zoba struktūru. Emaljas malas priekšējā zonā ir jānolīdzina. Zobu aizmugures zonā jānolīdzina tikai asās emaljas malas (apstrādes dimanti, 25-40 µm). Cervikāli bojājumi bez kariesa netiek sagatavoti; tie ir jāiztīra ar pumeku vai kādu citu atbilstošu pastu, izmantojot gumijas trauciņus vai rotējošas birstītes. Ja ir apkaļķots dentīns, adhezīva sasaisti var uzlabot viegla atkārtota kondicionēšana ar urbi. Pēc tam visi pārpalikumi no kavitātes ir jāizskalo ar ūdens strūklu. Kavitāte ir jānožāvē ar gaisu, kurā nav ūdens vai eļļas piemaisījumu.
4. Pulpas aizsardzība/bāze
Neizmantojiet bāzes masu, ja tiek lietota emaljas/dentīna saistviela. Ļoti dziļu kavitāšu gadījumā pulpas apkārtne jāapstrādā tikai ar kalcija hidroksīda oderi (piem., ApexCal®) un noslēgumā jāpārklāj ar spiedienizturīgu cementu (proti, stikla joncementu, piem., Vivaglass® Liner). Nepārklājiet kavitātes sieniņas, lai tās varētu izmantot saistījuma veidošanā ar emaljas/dentīna adhezīvu.
5. Matricas/starpzobu ķīļa lietošana
Izmantojiet aptinamo matricu kavitātēm, kas ietekmē proksimālo zonu, vai izmantojiet sekciju matricu un ķīli.
6. Kondicionēšana/saistvielas uzklāšana
Kondicionējiet zoba struktūru un uzklājiet saistvielu saskaņā ar izmantotā izstrādājuma lietošanas instrukcijām. Ieteicams izmantot Syntac® vai ExciTE® F (ar fosforskābes kodināšanu) vai AdheSE®, vai AdheSE® One F paškodinošo adhezīvu.
7. IPS Empress Direct lietošana
– Tā kā anatomiskās precizitātes radīšanai ir pieejami dažādi IPS Empress Direct dentīna un emaljas toņi dažādos caurspīdīguma līmeņos, lai izveidotu augstas kvalitātes restaurācijas, var izmantot jebkādu pārklāšanas metodi. Lai iegūtu optimālu rezultātu, lietojiet IPS Empress Direct maks. 2 mm (vai 1,5 mm dentīna toņi) biezumā un pielāgojiet ar OptraSculpt®/OptraSculpt® Pad instrumentu.
– Pietiekams apgaismojums novērsīs nepilnīgu polimerizāciju. Ieteicamais apgaismošanas ilgums (Exposure Time) un gaismas intensitāte (Light Intensity) ir parādīta 1. tabulā. Turiet gaismas izstarošanas logu iespējami tuvu restaurējošā materiāla virsmai.
– Izmantojot metāla matricu, pēc matricas izņemšanas papildus polimerizējiet restaurāciju no vaigu vai mēles/palatālās daļas, ja netika izmantota Bluephase® gaisma vai ja gaismas zondi nevarēja optimāli pozicionēt (piem., pārāk tālu no materiāla vai ar dažādu starojumu).
– Daudzos gadījumos pirmā slāņa uzklāšanai mūsdienās tiek lietots šķidrs kompozītmateriāls, lai izveidotu līdzenu kavitātes pamatni un vienkāršotu pēc tam izmantotā restaurējošā materiāla pielāgošanu. Šķidra kompozītmateriāla (piem., Tetric EvoFlow®) ieklāšana pirmajā slānī nav obligāta. Šis slānis jācietina gaismā atsevišķā darbībā (jāievēro norādījumi attiecīgajās lietošanas instrukcijās).
8. Procedūras pabeigšana/oklūzijas pārbaude/pulēšana
Pēc polimerizācijas noņemiet lieko materiālu, izmantojot piemērotu frēzi (piemēram, Astropol® F) vai dimanta urbi. Noņemiet lieko proksimālo materiālu ar dimanta vai volframa karbīda frēzi, pabeigšanas sloksnītēm vai elastīgiem diskiem. Pārbaudiet oklūziju un artikulāciju un veiciet atbilstošos slīpēšanas koriģējumus, lai novērstu priekšlaicīgu kontaktu vai nevēlamus artikulācijas ceļus uz restaurācijas virsmas. Izmantojiet silikona pulēšanas materiālus (piem., Astropol® P, Astropol® HP, Astrobrush vai OptraPol® Next Generation), kā arī pulēšanas diskus un pulēšanas sloksnītes, lai nopulētu restaurāciju līdz izturīgam augstas kvalitātes spīdumam.

Papildu informācija
1. Ja nepieciešams, iespējams uzklāt papildu IPS Empress Direct slāni tieši uz polimerizētā materiāla. Ja IPS Empress Direct restaurācija jau ir nopulēta, tā ir jānocietina un jāsamitrina ar Heliobond®, pirms uzklāt jaunu IPS Empress Direct slāni.
2. IPS Empress Direct kompozītmateriālam lietošanas laikā jābūt istabas temperatūrā. Ja šis materiāls tiek lietots zemā temperatūrā, to ir sarežģīti izņemt.
3. ⊗ Tikai vienreizējai lietošanai. Ja IPS Empress Direct pacienta mutē tiek uzklāts tieši no Cavifil, pēc lietošanas Cavifil jāizmet, lai novērstu krusteniskā piesārņojuma risku starp pacientiem.
4. Šļirces vai Cavifil materiālus nedrīkst dezinficēt ar oksidējošiem dezinfekcijas līdzekļiem.

Brīdinājumi
Nepolimerizēts kompozītmateriāls IPS Empress Direct nedrīkst nokļūt saskarē ar ādu, gļotādu vai acīm. Nepolimerizētam kompozītmateriālam IPS Empress Direct var būt viegli kairinoša iedarbība, un tas var izraisīt jutīgumu pret metakrilātiem. Tirdzniecībā esošie medicīniskie cimdi neaizsargā pret metakrilātu izraisītu jutīgumu.

Uzglabāšana
– Uzglabāšanas temperatūra: 2–28 °C.
– Noslēdziet šļirces/Cavifil izstrādājumus tūlīt pēc lietošanas. Gaismas iedarbība izraisa priekšlaicīgu polimerizāciju.

– Nelietojiet IPS Empress Direct pēc norādītā derīguma termiņa beigām.
– Derīguma termiņš: skatiet piezīmi uz Cavifil izstrādājumiem, šļircēm un iepakojumiem.

Uzglabāt bērniem nepieejamā vietā!
Izmantot tikai zobārstniecībā!

Materiāls paredzēts izmantošanai tikai zobārstniecībā. Materiāls ir jāizmanto tikai saskaņā ar lietošanas instrukciju. Ražotājs neuzņemas nekādu atbildību par bojājumiem, kas radušies, izmantojot materiālu neatbilstoši lietošanas instrukcijā sniegtajiem norādījumiem vai paredzētajam nolūkam. Produkta izmantotāja pienākums ir pārbaudīt produkta piemērotību un lietot produktu atbilstoši instrukcijā paredzētajam mērķim. Apraksti un norādītie dati nav uzskatāmi par jebkāda veida izstrādājuma īpašību garantiju un nav saistoši.

Lietuviškai

Aprašas
„IPS Empress® Direct" yra šviesa kietinamas, rentgeno spinduliams nepralaidus, nanohibridinis kompozitas, paremtas šiuolaikiškiausia technologija, tinkantis griežčiausius poreikius tenkinančioms restauracijoms gaminti. „IPS Empress Direct" kietėja šviesoje, 400–500 nm bangos intervale (mėlyna šviesa).

Atspalviai
„IPS Empress Direct" tiekiamas 32 skirtingų atspalvių ir 5 skirtingų skaidrumo lygių.
Galimi šie atspalviai:
Emalio atspalviai
A1, A2, A3, A3.5, A4, B1, B2, B3, B4, C1, C2, C3, D2, D3, „Bleach-L", „Bleach-XL"
Dentino atspalviai
A1, A2, A3, A3.5, A4, IVA5, IVA6, B1, B2, C3, D2, „Bleach-L", „Bleach-XL"
Permatomi atspalviai
„Trans 20", „Trans 30", „Opal"* *Dėl didelio skaidrumo rentgeno spinduliams laidus

Sudėtis
Monomero matricą sudaro dimetakrilatai (20–21,5 % svorio, opalinis atspalvis 17 % svorio). Užpildų sudėtyje yra bario stiklo, iterbio trifluorido, maišyto oksido, silikono dioksido ir kopolimero (77,5–79 % svorio, opalinio atspalvio 83 % svorio). Papildomos sudėtinės dalys: priedai, katalizatoriai, stabilizatoriai ir pigmentai (<1,0 % svorio). Bendras neorganinių užpildų kiekis yra 75–79 % svorio arba 52–59 % tūrio (opalinis atspalvis 60,5 % svorio arba 45 % tūrio). Neorganinių užpildų dalelės dydis yra nuo 40 iki 3000 nm, vidutinis dalelės dydis yra 550 nm.

Indikacija
– Priekinės restauracijos (III, IV klasės)
– Restauracijos vidinėje srityje (I ir II klasės)
– Danties kaklelio restauracijos (V klasės, pvz., kaklelio karieso, šaknies erozijos, pleišto formos defektų atveju)
– Danties padėties ir formos korekcijos (pvz., dantų tarpo uždarymas, tarpdantinių „juodųjų trikampių" uždarymas, kandamojo krašto pailginimas)
– Tiesioginiai venyrai

Kontraindikacija
„IPS Empress Direct" restauracijas taikyti kontraindikuotina
– jei negalima užtikrinti, kad darbo sritis būtų sausa arba negali būti taikoma nustatyta technika;
– jei žinoma, kad pacientas yra alergiškas bet kuriai „IPS Empress Direct" sudėtyje esančiai medžiagai.

Šalutinis poveikis
Retais atvejais gali pasireikšti jautrumas sudedamosioms „IPS Empress Direct" dalims. Tokiais atvejais „IPS Empress Direct" naudoti nereikėtų.
Siekiant išvengti galimo pulpos sudirginimo, šalia jos esančios sritys turi būti apsaugotos tinkama pulpos / dentino apsauga (pasirinktinai ant šalia pulpos esančių sričių užtepkite preparato kalcio hidroksido pagrindu ir padenkite tinkamu užpildu).

Sąveika
Medžiagos, kurių sudėtyje yra eugenolio / gvazdikėlių aliejaus, gali slopinti kompozicinių medžiagų polimerizaciją. Todėl tokių medžiagų nereikėtų naudoti su „IPS Empress Direct". Derinant su katijoniniais burnos skalavimo skysčiais, dantų akmenų atidengimo medžiagomis arba chlorheksidinu galimas spalvos išblukimas.

Taikymas
1. Atspalvio nustatymas
Prieš atlikdami atspalvio nustatymą nuvalykite dantis. Emalio atspalvis parenkamas kai dantys yra dar drėgni. Dentino atspalvį galima nustatyti pridedant kortelę prie paruošto danties arba šalia iltinio danties kaklelio srities.
2. Izoliavimas
Būtina užtikrinti tinkamą izoliavimą, pageidautina – guminiu koferdamu (pvz., „OptraDam® Plus").
3. Ertmės paruošimas
Ertmė ruošiama laikantis adhezyvo technikos reikalavimų, t. y. apsaugant danties struktūrą. Emalio kraštai priekinėje srityje nusklembiami. Vidinėje srityje užapvalinami tik aštrūs emalio kraštai (galutinio apdorojimo deimantais, 25–40 µm). Karieso nepažeistų kaklelio defektų ruošti nereikia, jie tik nuvalomi pemza ar kitomis tinkamomis valymo pastomis, naudojant gumines taureles arba sukamuosius šepetėlius. Jei dentinas yra sklerotinis, paviršinis apdorojimas rožiniu galąstuvu gali pagerinti adhezyvo sukibimą. Po to visi ertmėje esantys likučiai pašalinami vandens srove. Ertmė išdžiovinama oru be vandens ir aliejaus priemaišų.
4. Pulpos apsauga / bazė
Netepkite bazinės medžiagos, kai naudojate emalio / dentino rišamąją medžiagą. Tik itin gilias šalia pulpos esančias sritis padenkite kalcio hidroksido medžiaga (pvz., „ApexCal®"), po to – spaudimui atspariu cementu (pvz., stiklo jonomerų cementu, tokiu kaip „Vivaglass® Liner"). Nedenkite kitų ertmės sienelių, nes jos gali būti naudojamos surišti su emalio / dentino adhezyvu.
5. Matricos / tarpdantinio pleišto įdėjimas
Jei ertmės liečia proksimalinę sritį, naudokite matricos apvyniojimą arba sekcinę matricą ir ją sutvirtinkite pleištu.
6. Rišamosios medžiagos paruošimas / uždėjimas
Apdorokite danties struktūrą ir užtepkite rišamosios medžiagos laikydamiesi taikomo produkto naudojimo instrukcijų. Rekomenduojame naudoti „Syntac®" arba „ExciTE®F" (su ėsdinimu fosforine rūgštimi) arba „AdheSE®" ar „AdheSE® One F" savaiminio ėsdinimo adhezyvu.
7. „IPS Empress Direct" uždėjimas
– Kadangi yra daug įvairaus skaidrumo „IPS Empress Direct" dentino ir

emalio atspalvių, skirtų atkurti detalią anatomiją, gali būti kūrybingai naudojama bet kuri sluoksnio dengimo technika, kad restauracijos būtų itin estetiškos. Norint pasiekti optimalų rezultatą denkite „IPS Empress Direct“ ne storesniais kaip 2 mm (arba 1,5 mm dentino atspalvio) sluoksniais ir pritaikykite, pvz., „OptraSculpt®“ / „OptraSculpt® Pad“.
- Pakankamai ilga ekspozicija polimerizuojančioje šviesoje apsaugo nuo nevisiškos polimerizacijos. Rekomendacijas dėl ekspozicijos laiko (Exposure Time) ir šviesos intensyvumo (Light Intensity) žr. 1 lentelėje. Laikykite šviesos spinduliavimo langelį kiek galima arčiau restauracinės medžiagos paviršiaus.
- Kai naudojama metalinė matrica, ją nuėmę papildomai polimerizuokite restauraciją iš bukalinės ar lingvalinės / palatalinės pusės, jeigu buvo naudojamas kietinimas ne „Bluephase®“ šviesa arba šviesos zondo nepavyksta nustatyti optimalioje padėtyje (pvz., jis yra per toli nuo medžiagos arba taikant sklaidomąjį spinduliavimą).
- Daugeliu atveju pradiniam sluoksniui dabar naudojamas takusis kompozitas, taip siekiama sukurti lygų ertmės pagrindą ir supaprastinti vėliau naudojamos restauracinės medžiagos pritaikymą. Takųjį kompozitą (pvz., „Tetric EvoFlow®“) naudoti pradiniam plonam sluoksniui nebūtina. Šis sluoksnis turi būti kietinamas šviesa atskirame etape (būtina laikytis atitinkamoje naudojimo instrukcijoje pateikiamų nurodymų).

8. Apdaila / sąkandžio tikrinimas / poliravimas

Po polimerizavimo pašalinkite medžiagos perteklių tinkamais apdailinimo instrumentais (pvz., „Astropol® F“) arba smulkiais deimantais. Pašalinkite proksimalinį perteklių deimantu, šlifavimo antgaliais arba apdailos juostelėmis arba lanksčiais šlifavimo diskais. Patikrinkite sukandimą ir sujungimą, tinkamomis korekcinėmis šlifavimo priemonespriemonėmis panaikinkite per ankstyvą restauracijų paviršiaus kontaktą ar nepageidaujamus sujungimus. Poliruokite restauracijas silikoniniais poliruokliais (pvz., „Astropol® P“, „Astropol® HP“, „Astrobrush“ arba „OptraPol® Next Generation“), o taip pat poliravimo diskais ir juostelėmis, kol restauracijos taps tvirtos ir itin blizgios.

Papildoma informacija

1. Jei reikia, papildomas „IPS Empress Direct“ medžiagos kiekis gali būti tiesiogiai dedamas ant polimerizuotos medžiagos. Jeigu „IPS Empress Direct“ restauracija jau buvo nupoliruota, iš pradžių ją reikia pašiurkštinti ir sudrėkinti naudojant „Heliobond®“, tik po to dėti naują „IPS Empress Direct“ sluoksnį.
2. Dedant „IPS Empress Direct“ turi būti kambario temperatūros. Esant žemai temperatūrai medžiagą sunku išstumti.
3. Skirta tik vienkartiniam naudojimui. Jeigu „IPS Empress Direct“ dedama iš „Cavifil“ tiesiai paciento burnoje, panaudotą „Cavifil“ reikia išmesti, kad būtų išvengta pacientų tarpusavio užkrėtimo.
4. Švirkštų arba „Cavifils“ negalima dezinfekuoti oksiduojančiomis dezinfekavimo medžiagomis.

Įspėjimas

Saugokite, kad nepolimerizuotos „IPS Empress Direct“ nepatektų ant odos, gleivinės membranos ar į akis. Nepolimerizuota „IPS Empress Direct“ gali šiek tiek dirginti ir sukelti jautrumą metakrilatams. Įprastos parduodamos medicininės pirštinės neužtikrina apsaugos nuo jautrumo metakrilatams.

Saugojimas

- Saugojimo temperatūra 2–28 °C.
- Uždarykite švirkštus / „Cavifils“ iš karto po naudojimo. Poveikis šviesa sukelia per ankstyvą polimerizaciją.
- Nenaudokite „IPS Empress Direct“ po nurodytos galiojimo datos.
- Tinkamumo naudoti laikas: žr. informaciją ant „Cavifil“, švirkštų ir pakuotės.

Laikykite medžiagą vaikams nepasiekiamoje vietoje.
Skirta naudoti tik odontologams.

Medžiaga buvo sukurta naudoti tik odontologijoje. Apdorojimas turi būti atliekamas tiksliai laikantis naudojimo informacijos. Mes neatsakome už žalą, atsiradusią nesilaikant instrukcijos arba nustatytos naudojimo paskirties. Naudotojas atsako už produktų tinkamumo patikrinimą ir naudojimą pagal paskirtį, kuri aiškiai nėra nurodyta instrukcijose. Aprašai ir duomenys nesuteikia garantijos priedams ir nėra įpareigojantys.

Table 1

Unit / Program	Bluephase® C8	Bluephase® Style	Bluephase®	Bluephase® 20i
**Turbo**	–	–	–	5 s
**High Power**	15 s	10 s	10 s	10 s
**Soft Start**	20 s	–	15 s	15 s

Light Intensity	Exposure Time
≥ 500 mW/cm^2	20 s
≥ 1‘000 mW/cm^2	10 s

Ivoclar Vivadent AG
Bendererstrasse 2, 9494 Schaan, Liechtenstein,
Tel. +423 235 35 35, Fax +423 235 33 60
www.ivoclarvivadent.com

Ivoclar Vivadent Pty. Ltd.
1 – 5 Overseas Drive, P.O. Box 367, Noble Park, Vic. 3174, Australia
Tel. +61 3 9795 9599, Fax +61 3 9795 9645
www.ivoclarvivadent.com.au

Ivoclar Vivadent Ltda.
Alameda Caiapós, 723, Centro Empresarial Tamboré, CEP 06460-110 Barueri – SP, Brazil
Tel. +55 11 2424 7400, Fax +55 11 3466 0840
www.ivoclarvivadent.com.br

Ivoclar Vivadent Inc.
1-6600 Dixie Road, Mississauga, Ontario, L5T 2Y2, Canada
Tel. +1 905 670 8499, Fax +1 905 670 3102
www.ivoclarvivadent.us

Ivoclar Vivadent Shanghai
Trading Co., Ltd., 2/F Building 1, 881 Wuding Road, Jing An District
200040 Shanghai, China
Tel. +86 21 6032 1657, Fax +86 21 6176 0968
www.ivoclarvivadent.com

Ivoclar Vivadent Marketing Ltd.
Calle 134 No. 7-B-83, Of. 520, Bogotá, Colombia
Tel. +57 1 627 3399, Fax +57 1 633 1663
www.ivoclarvivadent.co

Ivoclar Vivadent SAS
B.P. 118, F-74410 Saint-Jorioz, France
Tel. +33 4 50 88 64 00, Fax +33 4 50 68 91 52
www.ivoclarvivadent.fr

Ivoclar Vivadent GmbH
Dr. Adolf-Schneider-Str. 2, D-73479 Ellwangen, Jagst, Germany
Tel. +49 7961 889 0, Fax +49 7961 6326
www.ivoclarvivadent.de

Wieland Dental + Technik GmbH & Co. KG
Schwenninger Strasse 13, D-75179 Pforzheim, Germany
Tel. +49 7231 3705 0, Fax +49 7231 3579 59
www.wieland-dental.com

Ivoclar Vivadent Marketing (India) Pvt. Ltd.
503/504 Raheja Plaza, 15 B Shah Industrial Estate, Veera Desai Road, Andheri (West)
Mumbai, 400 053, India
Tel. +91 22 2673 0302, Fax +91 22 2673 0301
www.ivoclarvivadent.in

Ivoclar Vivadent s.r.l.
Via Isonzo 67/69, 40033 Casalecchio di Reno (BO), Italy
Tel. +39 051 6113555, Fax +39 051 6113565
www.ivoclarvivadent.it

Ivoclar Vivadent K.K.
1-28-24-4F Hongo, Bunkyo-ku, Tokyo 113-0033, Japan
Tel. +81 3 6903 3535, Fax +81 3 5844 3657
www.ivoclarvivadent.jp

Ivoclar Vivadent Ltd.
12F W-Tower, 1303-37, Seocho-dong, Seocho-gu, Seoul 137-855, Republic of Korea
Tel. +82 2 536 0714, Fax +82 2 596 0155
www.ivoclarvivadent.co.kr

Ivoclar Vivadent S.A. de C.V.
Av. Insurgentes Sur No. 863, Piso 14, Col. Napoles, 03810 México, D.F., México
Tel. +52 55 5062 1000, Fax +52 55 5062 1029
www.ivoclarvivadent.com.mx

Ivoclar Vivadent BV
De Fruittuinen 32, 2132 NZ Hoofddorp, Netherlands
Tel. +31 23 529 3791, Fax +31 23 555 4504
www.ivoclarvivadent.com

Ivoclar Vivadent Ltd.
12 Omega St, Rosedale, PO Box 303011 North Harbour, Auckland 0751, New Zealand
Tel. +64 9 914 9999, Fax +64 9 914 9990
www.ivoclarvivadent.co.nz

Ivoclar Vivadent Polska Sp. z o.o.
Al. Jana Pawla II 78, 00-175 Warszawa, Poland
Tel. +48 22 635 5496, Fax +48 22 635 5469
www.ivoclarvivadent.pl

Ivoclar Vivadent Marketing Ltd.
Prospekt Andropova 18 korp. 6/ , office 10-06, 115432 Moscow, Russia
Tel. +7 499 418 0300, Fax +7 499 418 0310
www.ivoclarvivadent.ru

Ivoclar Vivadent Marketing Ltd.
Qlaya Main St., Siricon Building No.14, 2nd Floor, Office No. 204
P.O. Box 300146, Riyadh 11372, Saudi Arabia
Tel. +966 11 293 8345, Fax +966 11 293 8344
www.ivoclarvivadent.com

Ivoclar Vivadent Pte. Ltd.
171 Chin Swee Road, #02-01 San Centre, Singapore 169877
Tel. +65 6535 6775, Fax +65 6535 4991
www.ivoclarvivadent.com

Ivoclar Vivadent S.L.U.
C/ Ribera del Loira nº 46, 5ª planta, 28042 Madrid, Spain
Tel. + 34 913 757 820, Fax + 34 913 757 838
www.ivoclarvivadent.es

Ivoclar Vivadent AB
Dalvägen 14, S-169 56 Solna, Sweden
Tel. +46 8 514 939 30, Fax +46 8 514 939 40
www.ivoclarvivadent.se

Ivoclar Vivadent Liaison Office
: Tesvikiye Mahallesi, Sakayik Sokak, Nisantas' Plaza No:38/2, Kat:5 Daire:24
34021 Sisli – Istanbul, Turkey
Tel. +90 212 343 0802, Fax +90 212 343 0842
www.ivoclarvivadent.com

Ivoclar Vivadent Limited
Ground Floor Compass Building, Feldspar Close, Warrens Business Park, Enderby
Leicester LE19 4SE, United Kingdom
Tel. +44 116 284 7880, Fax +44 116 284 7881
www.ivoclarvivadent.co.uk

Ivoclar Vivadent, Inc.
175 Pineview Drive, Amherst, N.Y. 14228, USA
Tel. +1 800 533 6825, Fax +1 716 691 2285
www.ivoclarvivadent.us
