# Supplementary material for: Effect of different scaling instruments on the roughness of esthetic resin composite restorations: an in vitro study
Source: Front Dent Med. 2026 Aug 28;7:1894177. doi: 10.3389/fdmed.2026.1894177 (PMC13561887; doi:10.3389/fdmed.2026.1894177)
Supplement: Supplementary file 1 [file Datasheet1.zip › Palfique.pdf]

■PRODUCT DESCRIPTION AND GENERAL INFORMATION

1) PALFIQUE LX5 is a light-cured, radiopaque, submicron filled composite resin for use in anterior and posterior restorations and is indicated for all carious classes. PALFIQUE LX5 contains 82% by weight (71% by volume) of silica-zirconia filler and composite filler. A high filler load offers decreased polymerization shrinkage. All inorganic filler contained in PALFIQUE LX5 is a spherical submicron filler (mean particle size: 0.2 μm, particle size range: 0.1 to 0.3 μm) that is designed to deliver excellent gloss retention and wear resistance. The monomer matrix contains Bis-GMA and Triethylene glycol dimethacrylate.
2) PALFIQUE LX5 implements Radical-Amplified Photopolymerization initiator technology (RAP technology). Curing time for PALFIQUE LX5 can be shortened by one-third using a light curing unit with a wavelength range of 400 to 500nm (peak: 470nm) compared to our conventional resin composite. Please see the table depicting the relationship between curing time and increment depth (■INDICATIONS FOR FILLING AND CURING).
3) RAP technology facilitates a longer working time for PALFIQUE LX5.
4) PALFIQUE LX5 is provided either in SYRINGE or Pre Loaded Tip(PLT).

■SHADE

A1, A2, A3, A3.5, A4, A5, B1, B2, B3, B4, C1, C2, C3, OA1, OA2, OA3, OPA2, BW (Bleach White), WE (White Enamel), CE (Clear Enamel/Incisal) and SW (Snow White).

- Opalescent shades (OA1, OA2, OA3) have adequate opacity designed for blocking out the dark shine that comes through the oral cavity (Class III and Class IV restoration). The opalescent shades also serve as a dentin layer for the multi-shade layering technique. Shades OA1, OA2 and BW closely match those of primary teeth. However, they are not designed for masking metal or a darkly stained area such as tetracycline stained.
- OPA2 is designed for masking slight stain or reconstructing a highly opaque tooth (use as a thin layer at the lingual cavity wall of class III and IV).
- Please check with your local distributor for available shades.

■INDICATIONS

- Direct anterior and posterior restorations including the occlusal surface and all carious classes
- Direct bonded composite veneer
- Diastema closure
- Repair of porcelain/composite

■CONTRAINDICATIONS

PALFIQUE LX5 contains methacrylic monomers. DO not use PALFIQUE LX5 for patients allergic to or hypersensitive to methacrylic and related monomers.

■PRECAUTIONS

1) DO NOT use PALFIQUE LX5 for any purpose other than those listed in these instructions. Use PALFIQUE LX5 only as directed herein.
2) PALFIQUE LX5 is designed for sale and use by licensed dental care professionals only. It is not designed for sale nor is it suitable for use by non-dental care professionals.
3) DO NOT use PALFIQUE LX5 if the safety seals are broken or appear to have been tampered with.
4) If PALFIQUE LX5 causes an allergic reaction or oversensitivity, discontinue its use immediately.
5) Use examination gloves (plastic, vinyl, or latex) at all times when handling PALFIQUE LX5 to avoid the possibility of allergic reactions from methacrylic monomers. Note: Certain substances/materials may penetrate through examination gloves. If PALFIQUE LX5 comes in contact with the examination gloves, remove and dispose of the gloves, and wash hands thoroughly with water as soon as possible.
6) Avoid contact of PALFIQUE LX5 with eyes, mucosal membrane, skin and clothing.
   - If PALFIQUE LX5 comes in contact with the eyes, thoroughly flush eyes with water

Scanned with CamScanner
