# Supplementary material for: Effect of different scaling instruments on the roughness of esthetic resin composite restorations: an in vitro study
Source: Front Dent Med. 2026 Aug 28;7:1894177. doi: 10.3389/fdmed.2026.1894177 (PMC13561887; doi:10.3389/fdmed.2026.1894177)
Supplement: Supplementary file 1 [file Datasheet1.zip › Vittra.pdf]

the product and your last patient.

Product description

VITTRA APS is a highly esthetic light-cure composite resin recommended for restorations of all classes in anterior and posterior teeth. The composite is radiopaque, its loading particles are composed by spheres of a zirconia complex with average load particle size of 0.2 micron, with total inorganic load of 72% to 82% in weight (52% to 60% in volume). These features contribute to the mechanical and esthetic properties, especially evidenced by how easy it is to obtain proper polishing and by the long-lasting surface shine.

VITTRA APS' formula is free of Bis-GMA and Bis-EMA, following the present trend of products free of Bisphenol A (BPA). APS is the acronym for Advanced Polymerization System, and it consists of a combination of different photoinitiators that interact among each other, amplifying the curing capacity of light emitted from light-curing units. Added to different materials, the system provides different advantages.

In the case of VITTRA APS, APS adds, as a main benefit, the reduction or elimination of visual shade and opacity alteration* before-after light curing, which enhances the predictability of the result. Another factor is that, although there is very little concentration of camphorquinone, APS has great power of polymerization, allowing greater degree of conversion. The APS also allows the product to obtain superior stability to ambient light, providing longer working time to light-sensitive materials.

* Color change imperceptible to the naked human eye.

Basic composition

Active ingredients: methacrylate monomers mixture, photoinitiating composition (APS), coinitiators, stabilizer and silane.

Inactive ingredients: load particles of zirconia, silica and pigments.

Presentation

Check product availability in your country.

Refill:

Syringe with 4 g: DA1, DA2, DA3, DA3.5, DB1, EA1, EA2, EA3, EA3.5, EB1, E-Bleach, DBL1, EBL1, DBL2, EBL2, DBL3 and EBL3.

Syringe with 2 g: DA0, DA4, DA5, VM, VH, Trans OPL and Trans N.

Essential Kit: DA1, DA2, DA3, EA1, EA2, Trans N + Ambar APS + Condac 37.

Kit Bleach: DA0, DA1, VH, E-Bleach, Trans OPL + Ambar APS + Condac 37 + Diamond Excel.

Capsules: package containing 20 capsules of 0.2 g each.

Product indication

The composite is suitable for use in permanent and primary teeth:

- Direct restorations in anterior and posterior teeth (I, II, III, IV, V and VI);
- Direct veneers with composites;
- Cementation of tooth fragments;
- Core build-ups;
- Teeth splinting;

Scanned with CamScanner
